# Comparative physiological, metabolomic, and transcriptomic analyses reveal developmental stage-dependent effects of cluster bagging on phenolic metabolism in Cabernet Sauvignon grape berries

**DOI:** 10.1186/s12870-019-2186-z

**Published:** 2019-12-26

**Authors:** Run-Ze Sun, Guo Cheng, Qiang Li, Yan-Rong Zhu, Xue Zhang, Yu Wang, Yan-Nan He, Si-Yu Li, Lei He, Wu Chen, Qiu-Hong Pan, Chang-Qing Duan, Jun Wang

**Affiliations:** 1 Center for Viticulture and Enology, College of Food Science and Nutritional Engineering, China Agricultural University, Beijing, 100083 China; 2 Key Laboratory of Viticulture and Enology, Ministry of Agriculture and Rural Affairs, Beijing, 100083 China; 3 Key Laboratory of Plant Resources, Institute of Botany, Chinese Academy of Science, Beijing, 100093 China; 4 CITIC Guoan Wine Co. Ltd., Xinjiang, 832200 Manasi China; 5 Grape and Wine Research Institute, Guangxi Academy of Agricultural Sciences, Nanning, 530007 China; 6 Institute of Vegetables and Flowers, Chinese Academy of Agricultural Sciences, Beijing, 100081 China; 7 Nongfu Spring Co. Ltd., Hangzhou, 310000 China; 8 Ruifeng Oseis (Yantai) Wine Manor Co. Ltd., Yantai, 264010 China; 9 College of Bioscience & Bioengineering, Hebei University of Science and Technology, Shijiazhuang, 050018 China

**Keywords:** Cluster bagging, Grape berry, Phenolic compounds, Transcriptome, *Vitis vinifera* cv. Cabernet sauvignon, *VviFLS4*

## Abstract

**Background:**

Light conditions significantly influence grape berry ripening and the accumulation of phenolic compounds, but the underlying molecular basis remains partially understood. Here, we applied integrated transcriptomics and pathway-level metabolomics analyses to investigate the effect of cluster bagging during various developmental stages on phenolic metabolism in Cabernet Sauvignon grapes.

**Results:**

Bagging treatments had limited effects on berry quality attributes at harvest and did not consistently affect phenolic acid biosynthesis between seasons. Significantly elevated flavan-3-ol and flavonol contents were detected in re-exposed berries after bagging during early-developmental stages, while bagging after véraison markedly inhibited skin anthocyanin accumulation. Several anthocyanin derivatives and flavonol glycosides were identified as marker phenolic metabolites for distinguishing bagged and non-bagged grapes. Coordinated transcriptional changes in the light signaling components CRY2 and HY5/HYHs, transcription regulator MYBA1, and enzymes LAR, ANR, UFGT and FLS4, coincided well with light-responsive biosynthesis of the corresponding flavonoids. The activation of multiple hormone signaling pathways after both light exclusion and re-exposure treatments was inconsistent with the changes in phenolic accumulation, indicating a limited role of plant hormones in mediating light/darkness-regulated phenolic biosynthesis processes. Furthermore, gene-gene and gene-metabolite network analyses discovered that the light-responsive expression of genes encoding bHLH, MYB, WRKY, NAC, and MADS-box transcription factors, and proteins involved in genetic information processing and epigenetic regulation such as nucleosome assembly and histone acetylation, showed a high positive correlation with grape berry phenolic accumulation in response to different light regimes.

**Conclusions:**

Altogether, our findings provide novel insights into the understanding of berry phenolic biosynthesis under light/darkness and practical guidance for improving grape features.

## Background

Environmental and viticultural practices have long been known to influence grape and wine quality. Sunlight is a major factor associated with the development and composition of grape berries under natural solar radiation. The exposure of grape bunches to sunlight generally improves the content of berry total soluble solids (TSS) and phenolics, but it leads to a reduced berry weight, malic acid, amino acids, titratable acidity (TA), and juice pH [1–7]. However, the precise responses of grapevine to sunlight are heavily dependent upon the genotypes [8–10], climatic conditions [6, 11, 12], and berry developmental stages and seasonal variations [13–15].

Phenolic acids [mainly hydroxybenzoic acids (HBAs) and hydroxycinnamic acids (HCAs)], flavonoids (mainly flavan-3-ols, anthocyanins, and flavonols), and non-flavonoid polyphenols (mainly resveratrols) are three major classes of phenolic compounds detected in grapes and wine [16, 17]. These compounds are generally considered to act as free radical scavengers and UV radiation protectants and to play important roles in pigmentation/co-pigmentation and defense against fungal and bacterial pathogen infections in grape berries [17, 18]. Moreover, grape phenolics contribute to the color, bitterness, astringency, and antioxidant properties of wine and its potential benefits to human health [19, 20]. The impact of sunlight on phenolic biosynthesis in grape berries has been extensively investigated through a range of approaches, mostly involving the viticultural practices of artificial light excluding via bunch shading or exposing via leaf removal and cluster thinning [18]. HCAs in grape berries have been observed to be slightly affected by leaf removal at the beginning of the maturation period [21, 22]. However, significantly increased HCA levels at both véraison and harvest associated with enhanced solar exposure have also been reported [13]. Exposure to solar UV-B radiation leads to increased individual (quercetin, kaempferol, and myricetin) and total flavonol concentrations in grape skins, while shading and UV light barriers significantly reduce them [6, 21, 23–27]. The accumulation of skin proanthocyanidins (PAs), the oligomeric and polymeric flavan-3-ol units, is reduced in shaded fruit during development, but inconsistent results at harvest have been observed among studies [23, 28, 29]. In contrast, sunlight exclusion induces changes in the composition of PAs, such as a decrease in the proportion of trihydroxylated subunits and the mean degree of polymerization (mDP) within PAs, while improved light exposure induces the biosynthesis of PAs and increases its proportion of hydroxylated subunits in grape skins [24, 30]. Generally, shading treatment inhibits the accumulation of skin anthocyanins, accompanied by decreases in the proportion of trihydroxylated anthocyanins [23, 30–34]. However, the response of skin anthocyanins to sunlight exposure treatments is variable and heavily dependent on the timing and degree of severity [14, 35]. It appears that early leaf removal or cluster thinning induces a substantial acceleration of anthocyanin biosynthesis and a higher anthocyanin concentration [10, 36–39], whereas excessive solar radiation/irradiance causes sunburn damage and is not conducive to optimal anthocyanin accumulation in berries [12, 15, 21, 33, 40, 41].

Phenolic compounds are biosynthesized along general metabolic pathways of shikimate and phenylpropanoid by the activity of multi-enzyme complexes in the cytosol. It is well established that the transcriptional regulation of flavonoid biosynthetic pathway structural genes is orchestrated by a ternary complex involving transcription factors from the R2R3-MYB, basic helix-loop-helix (bHLH), and WD40-repeat proteins in several plant species [42, 43]. Previous studies have demonstrated the light-induced expression of an array of R2R3-MYB transcription factors that are known to regulate the general flavonoid pathway (VviMYB5a), as well as those specifically associated with flavonols (VviMYBF1/VviMYB12), flavan-3-ols (VviMYBPA1 and VviMYBPA2), and anthocyanins (VviMYBA1 and VviMYBA2) biosynthesis in grapevine [24, 27, 32, 35, 44–47]. Additional potential regulators belonging to the grape basic-leucine zipper (bZIP) transcription factor family, VvibZIPC22, ELONGATED HYPOCOTYL 5 (VviHY5) and its close homolog VviHYH, have also been linked to the activation of key structural genes of the flavonoid pathway as well as flavonoid biosynthesis under UV radiation [48–50]. More recently, integrated transcriptomic and metabolomic analyses have revealed the putative involvement of multiple hormone signals, ubiquitin-dependent protein degradation, and a large number of coordinately expressed transcription factors in the process of phenolic biosynthesis in response to different light (including UV radiation) exposure conditions [5, 9, 13, 50–53]. Limited data are available regarding the effect of different sunlight exclusion regimes on dynamic changes in the transcriptome and the detailed pathway-level metabolome in grape berries during different developmental stages, although many studies have been conducted to determine the impact of multiple environmental conditions on berry phenolic accumulation.

The present study assessed the impact of shading on ripening and phenolic accumulation in grape berries (*Vitis vinifera* L. cv. Cabernet Sauvignon) grown under field conditions. The contents and compositions of phenolic acids (HCAs and HBAs) and flavonoids (flavonols, flavan-3-ols, and anthocyanins) together with the transcriptome dynamics were monitored in grape berries subjected to different cluster bagging treatments during various phenological stages. To broaden our understanding of the regulatory mechanism of phenolic metabolism in grape berries grown under shade conditions, the light-responsiveness of a *flavonol synthase* gene promoter was characterized, and the genome-wide co-expression network of phenolic biosynthetic related structural genes and their putative upstream signals and regulators were further analyzed.

## Results

### Effect of cluster bagging on canopy microclimate and berry ripening

**Fig. 1:**
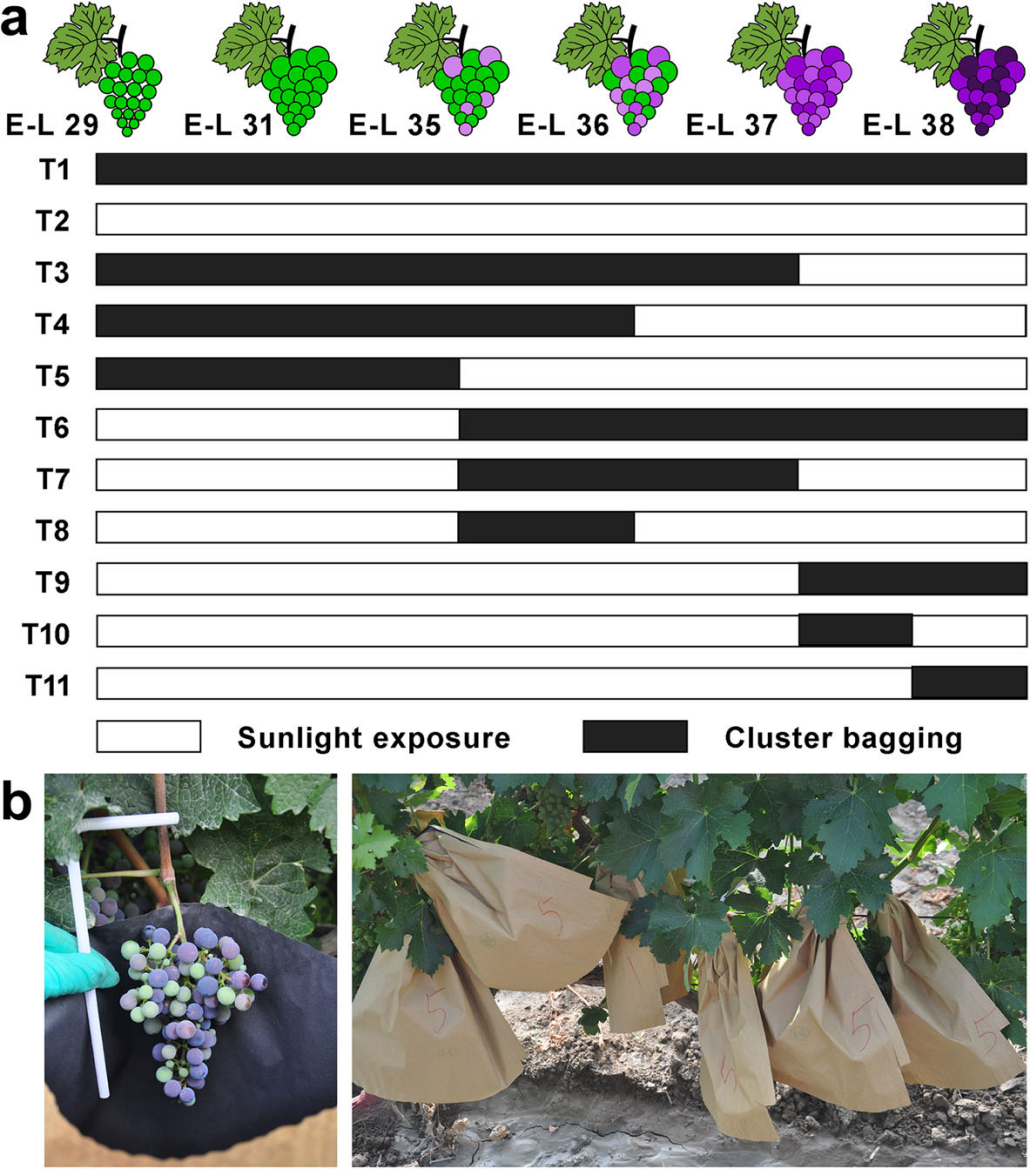
Experimental design for cluster bagging treatments during different developmental stages (**a**) and field photographs of bagging-treated Cabernet Sauvignon grapes (**b**). T1, cluster bagging from 3 WAF until harvest; T2, control group; T3, cluster bagging at E-L 29 stage and bag removal at E-L 37 stage; T4, cluster bagging at E-L 29 stage and bag removal at E-L 36 stage; T5, cluster bagging at E-L 29 stage and bag removal at E-L 35 stage; T6, cluster bagging from E-L 35 to E-L 38 stages; T7, cluster bagging at E-L 35 stage and bag removal at E-L 37 stage; T8, cluster bagging at E-L 35 stage and bag removal at E-L 36 stage; T9, cluster bagging from E-L 37 to E-L 38 stages; T10, cluster bagging at E-L 37 stage and bag removal at E-L 37.5 stage; T11, cluster bagging from E-L 37.5 to E-L 38 stages

In two consecutive years (2012 and 2013), ten cluster sunlight excluding treatments were established in the vines through cluster bagging and bag removal at different developmental stages as follows (Fig. 1a): T1, cluster bagging from 3 weeks after flowering (WAF) (berry pepper-corn size; E-L 29) until harvest (complete ripening, E-L 38); T3, cluster bagging at E-L 29 stage and bag removal at the end of véraison (berries not quite ripe, E-L 37); T4, cluster bagging at E-L 29 stage and bag removal at mid-ripening stage (berries with intermediate Brix values, E-L 36); T5, cluster bagging at E-L 29 stage and bag removal at early-véraison (berries begin to color, E-L 35); T6, cluster bagging from E-L 35 to E-L 38 stages; T7, cluster bagging at E-L 35 stage and bag removal at E-L 37 stage; T8, cluster bagging at E-L 35 stage and bag removal at E-L 36 stage; T9, cluster bagging from E-L 37 to E-L 38 stages; T10, cluster bagging at E-L 37 stage and bag removal at one week before complete ripening stage (E-L 37.5); T11, cluster bagging from E-L 37.5 to E-L 38 stages. Clusters exposed to sunlight during the entire developmental stages (T2) served as the control group. Similar local meteorological conditions were detected during the field experiments of two consecutive growing seasons, except for a higher temperature at the green and ripening stages of berry development in 2012 (Additional file 1: Table S1). Monitoring of the microclimate showed that the level of photosynthetically active radiation (PAR) and solar radiation was markedly reduced by cluster bagging treatment during each developmental stage of grape berries in both experimental years. Temperature and relative humidity in the bags were only slightly elevated and decreased, respectively, compared with the canopy environment during the two growing seasons (Additional file 2: Table S2).

**Fig. 2:**
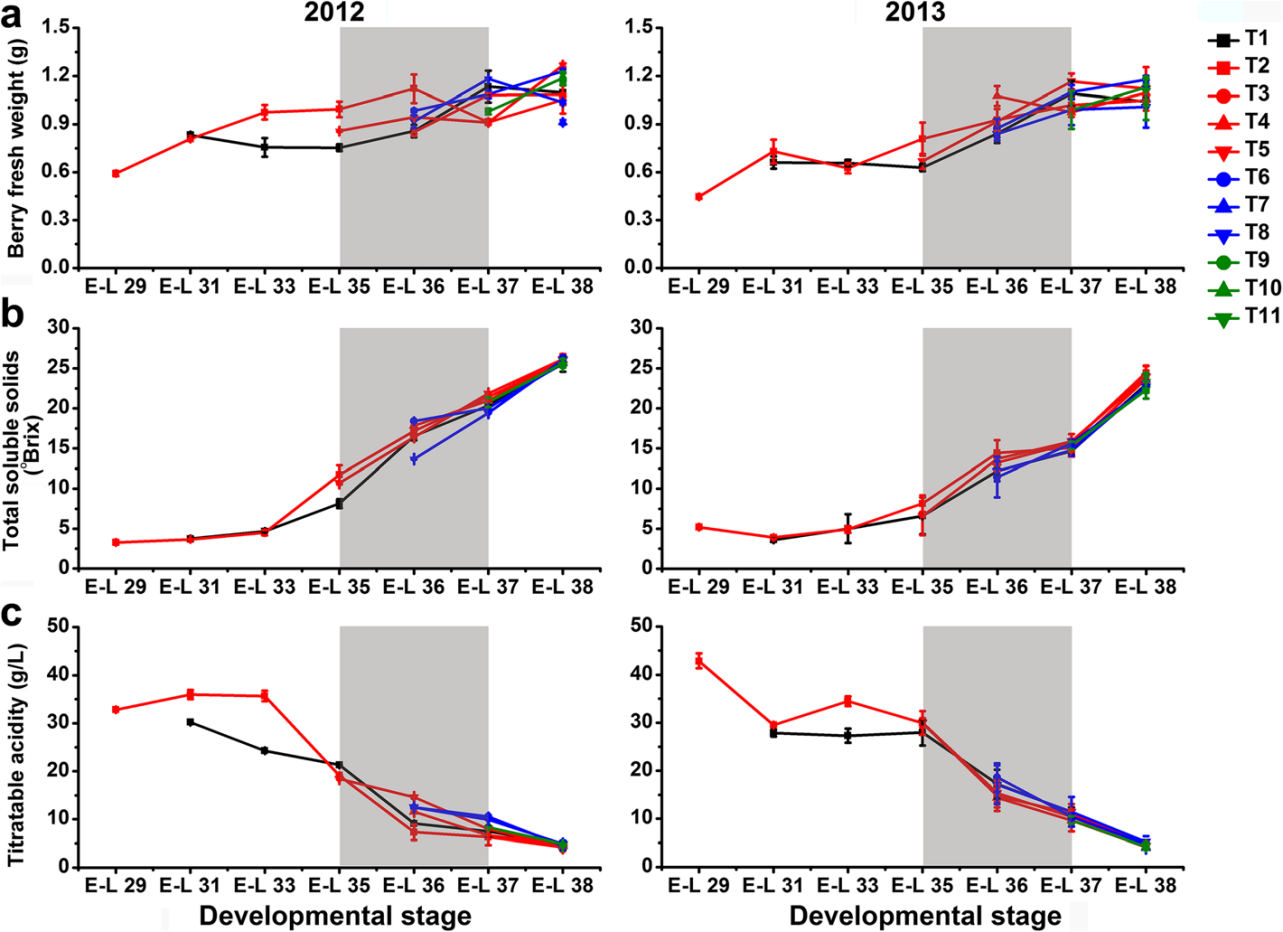
Influence of cluster bagging treatments on the fresh weight (**a**), total soluble solids (**b**) and titratable acidity contents (**c**) of Cabernet Sauvignon grape berries during development over two seasons. Data are the mean ± SD of three biological replicates. The light grey background represents the phenological phase of véraison from 5 to 100% of colored berries. T1, cluster bagging from 3 WAF until harvest; T2, control group; T3, cluster bagging at E-L 29 stage and bag removal at E-L 37 stage; T4, cluster bagging at E-L 29 stage and bag removal at E-L 36 stage; T5, cluster bagging at E-L 29 stage and bag removal at E-L 35 stage; T6, cluster bagging from E-L 35 to E-L 38 stages; T7, cluster bagging at E-L 35 stage and bag removal at E-L 37 stage; T8, cluster bagging at E-L 35 stage and bag removal at E-L 36 stage; T9, cluster bagging from E-L 37 to E-L 38 stages; T10, cluster bagging at E-L 37 stage and bag removal at E-L 37.5 stage; T11, cluster bagging from E-L 37.5 to E-L 38 stages

The development of grape berries from the control group was faster in 2012 than those in 2013 based on the changes of berry weight and TSS content (Fig. 2a, b), partially because of the relatively higher solar radiation and temperature during berry development in the first growing season (Additional file 1: Table S1 and Additional file 2: Table S2). Differences in the time-course of development and ripening were observed between berries from bagged and non-bagged clusters in both 2012 and 2013. The weight of T1-treated berries was statistically significantly (*p* ≤ 0.01, the same below) lower during véraison but higher after véraison than those of the exposed berries in both years (Fig. 2a). At harvest, no significant differences in berry weight were observed from not only T1 but also T3, T4, and T8 treatments, in comparison with the control in both years. However, the influence of cluster bagging treatments during other developmental stages on berry weight at harvest was inconsistent between years. There was no significant difference in the content of TSS in berries from each developmental stage among treatments during the two years (Fig. 2b). Reduction of titratable acidity during berry ripening was delayed by T1, T4, T5, T6, and T8 treatments in 2012, but no significant difference was observed at harvest (Fig. 2c).

### Influence of cluster bagging on phenolic accumulation

**Fig. 3:**
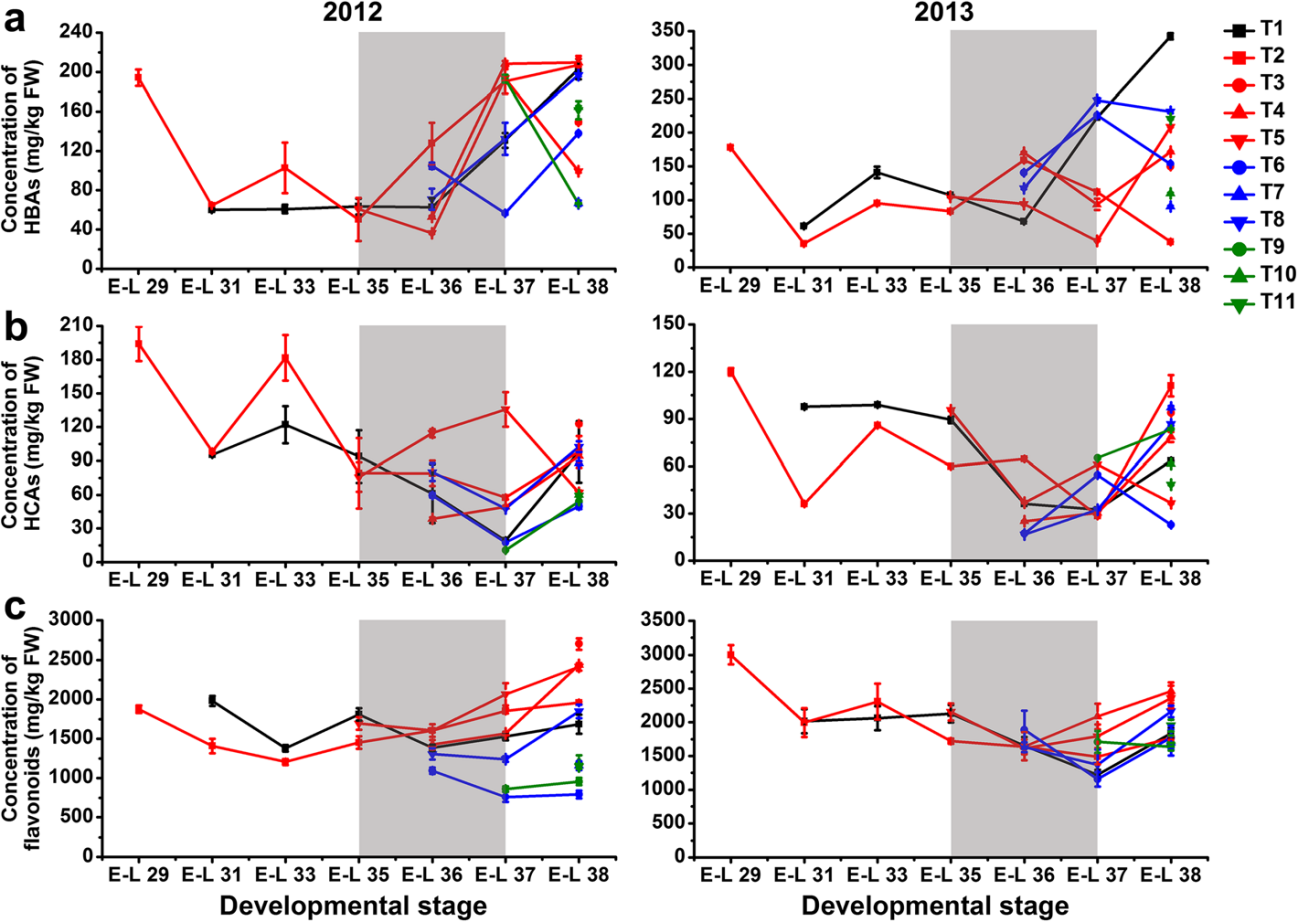
Changes in the accumulation of total hydroxybenzoic acids (HBAs) (**a**), hydroxycinnamic acids (HCAs) (**b**), and flavonoids (**c**) in different cluster bagging-treated grape berries during development over two seasons. Data are the mean ± SD of three biological replicates. The light grey background represents the phenological phase of véraison from 5 to 100% of colored berries. T1, cluster bagging from 3 WAF until harvest; T2, control group; T3, cluster bagging at E-L 29 stage and bag removal at E-L 37 stage; T4, cluster bagging at E-L 29 stage and bag removal at E-L 36 stage; T5, cluster bagging at E-L 29 stage and bag removal at E-L 35 stage; T6, cluster bagging from E-L 35 to E-L 38 stages; T7, cluster bagging at E-L 35 stage and bag removal at E-L 37 stage; T8, cluster bagging at E-L 35 stage and bag removal at E-L 36 stage; T9, cluster bagging from E-L 37 to E-L 38 stages; T10, cluster bagging at E-L 37 stage and bag removal at E-L 37.5 stage; T11, cluster bagging from E-L 37.5 to E-L 38 stages

**Fig. 4:**
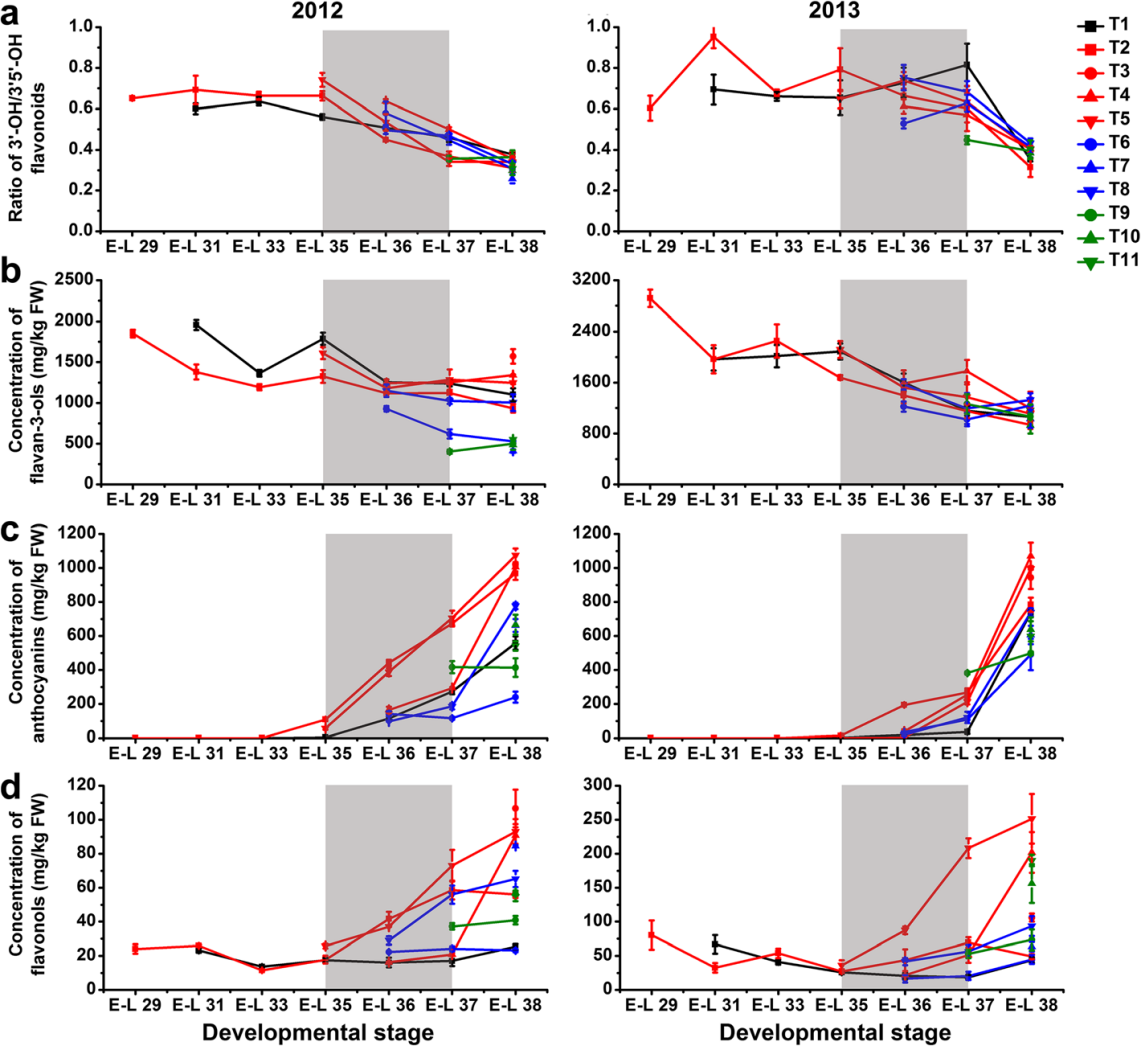
Changes in the ratio of dihydroxylated/trihydroxylated flavonoids (**a**) and the accumulation of flavan-3-ols (**b**), anthocyanins (**c**) and flavonols (**d**) in different cluster bagging-treated grape berries during development over two seasons. Data are the mean ± SD of three biological replicates. The light grey background represents the phenological phase of véraison from 5 to 100% of colored berries. T1, cluster bagging from 3 WAF until harvest; T2, control group; T3, cluster bagging at E-L 29 stage and bag removal at E-L 37 stage; T4, cluster bagging at E-L 29 stage and bag removal at E-L 36 stage; T5, cluster bagging at E-L 29 stage and bag removal at E-L 35 stage; T6, cluster bagging from E-L 35 to E-L 38 stages; T7, cluster bagging at E-L 35 stage and bag removal at E-L 37 stage; T8, cluster bagging at E-L 35 stage and bag removal at E-L 36 stage; T9, cluster bagging from E-L 37 to E-L 38 stages; T10, cluster bagging at E-L 37 stage and bag removal at E-L 37.5 stage; T11, cluster bagging from E-L 37.5 to E-L 38 stages

A statistically significantly (*p* ≤ 0.01) decreased concentration of both HBAs and HCAs was observed in T1-treated berries during development, but there was no significant difference at harvest in comparison to the control berries in 2012. In contrast, the content of HBAs in grapes was significantly increased during berry development and ripening, while the HCA content was significant increased before véraison and then significantly decreased from véraison to harvest by T1 treatment in the next growing season (Fig. 3a, b; Additional file 3: Table S3). The accumulation of HCAs in T5-treated berries was greatly induced from véraison to pre-harvest and thereafter significantly inhibited at harvest in both the 2012 and 2013 growing seasons. Other treatments, including T6, T10, and T11, also decreased the berry HCA content at harvest compared to the control in both years. In the first growing season, the accumulation of flavonoids in T1-treated berries was induced during expansion (from the E-L 31 to E-L 35 stages) and then inhibited during maturation (from the E-L 36 to E-L 38 stages) in comparison to the control berries, whereas opposite trends were observed in the next growing season (Fig. 3c). At harvest, the total flavonoid concentration was significantly increased in T3, T4, and T5-treated grapes, while it was decreased by T9 treatment in both years. However, the effect of other cluster bagging treatments on the accumulation of HBAs, HCAs and flavonoids was inconsistent between different seasons. In addition, the dihydroxylated/trihydroxylated flavonoid ratio in berry skin was significantly or moderately (0.01 < *p* ≤ 0.05) increased by T1, T3, T4, T5, T8, and T9 treatments in comparison to the control during the two growing seasons, but it was differentially affected by T6, T8, T10, and T11 between years (Fig. 4a).

The level of total flavan-3-ols was significantly increased in T1, T3, T4, and T5-treated grapes at harvest, whereas the effects of other cluster bagging treatments were variable during the two developmental seasons (Fig. 4b). Cluster bagging during véraison and/or the late stages of ripening (T1, T6, T7, T8, T9, T10, and T11) showed substantially inhibitory effects on the accumulation of skin anthocyanins from the beginning of each treatment until harvest over two seasons (Fig. 4c). With the exception of T1 and T8-treated berries in the second season, there was no change in terminal anthocyanin content in comparison to the control, although its biosynthesis was retarded during véraison. In contrast, cluster bagging treatments from 3 WAF and bag removal before post-véraison (T3, T4, and T5) did not markedly affect the final skin anthocyanin concentration in 2012, whereas in the next season, exposure of early-stage shaded berries to sunlight led to a significantly increased skin anthocyanin concentration at harvest. Cluster bagging at different phenological stages of berry development also showed an inconsistent impact on the biosynthesis of skin flavonols over the two seasons (Fig. 4d). T1 treatment was found to have no significant effect on the accumulation of flavonols during the early stage of véraison but significantly inhibited it at harvest compared with the control. Total flavonol content was significantly decreased in T4, T6, and T9-treated berries, while it was significantly increased by T5 treatment during véraison. Bag removal after véraison (T3, T4, T5, T7, and T8) resulted in a much higher flavonol level compared with control berries at harvest, whereas the effects of cluster bagging during ripening (T6, T9, T10 and, T11) on the accumulation of flavonols at harvest were inconsistent over the two seasons.

### Metabolic profiling of phenolics and identification of putative biomarkers

**Fig. 5:**
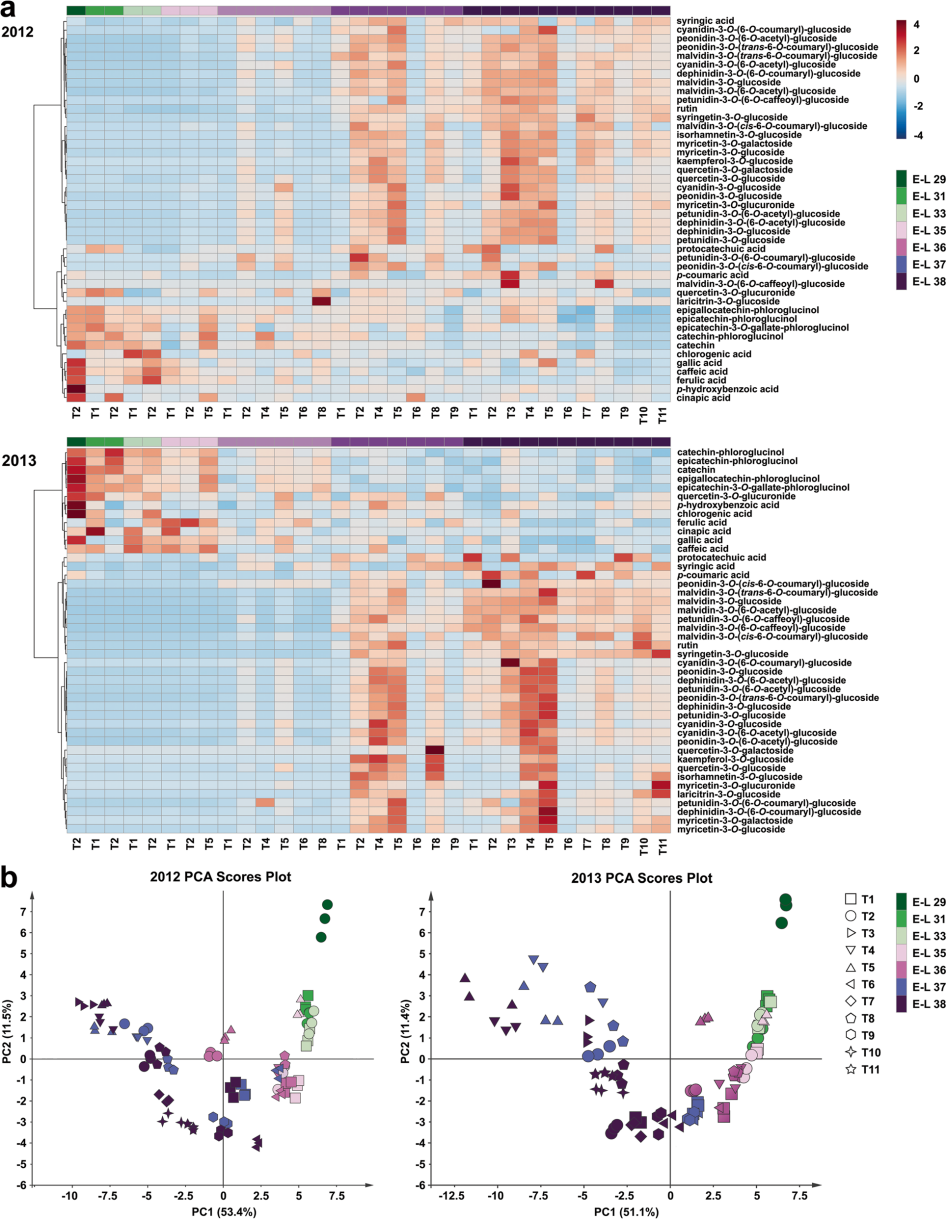
Heatmap visualization of hierarchical clustering analysis (**b**) and score scatter plot of principal component analysis (**a**) based on the phenolic profiles detected in different cluster bagging-treated grape berries during development in 2012 and 2013, respectively. T1, cluster bagging from 3 WAF until harvest; T2, control group; T3, cluster bagging at E-L 29 stage and bag removal at E-L 37 stage; T4, cluster bagging at E-L 29 stage and bag removal at E-L 36 stage; T5, cluster bagging at E-L 29 stage and bag removal at E-L 35 stage; T6, cluster bagging from E-L 35 to E-L 38 stages; T7, cluster bagging at E-L 35 stage and bag removal at E-L 37 stage; T8, cluster bagging at E-L 35 stage and bag removal at E-L 36 stage; T9, cluster bagging from E-L 37 to E-L 38 stages; T10, cluster bagging at E-L 37 stage and bag removal at E-L 37.5 stage; T11, cluster bagging from E-L 37.5 to E-L 38 stages

We reproducibly detected a total of 44 different phenolic compounds within the present grape berry samples, including four HBAs, five HCAs, five flavan-3-ols, 19 anthocyanin derivatives and 11 flavonol glycosides. Hierarchical clustering analysis showed that most of the phenolic acids and flavan-3-ols were clustered together, whereas the accumulation of anthocyanin derivatives and flavonol glycosides in berries during development and different cluster bagging treatments exhibited similar patterns in both growing seasons (Fig. 5a). To investigate the influence of cluster bagging during different developmental stages on detailed phenolic accumulation, the data set for all phenolic compounds detected from each grape sample were pooled together to construct the principal component analysis (PCA). Score scatter plots of the PCA models demonstrated that the effects of different cluster bagging treatments on phenolic profiles were similar in both growing seasons (Fig. 5b). The first principal component (PC1) explained 53.4 and 51.1% of the variances in the initial data sets of all identified phenolics in the 2012 and 2013 growing seasons, respectively, while PC2 explained 11.5 and 11.4%. Grape samples could be roughly distinguished based on their developmental stages in both seasons, and a distinct separation between the control and bagging-treated groups was also detected within each stage of development. Moreover, the areas of several grape samples from different developmental stages were overlapped by PCA, indicating the effect of bagging treatment on the phenolic accumulation and ripening process of grape berries.

**Fig. 6:**
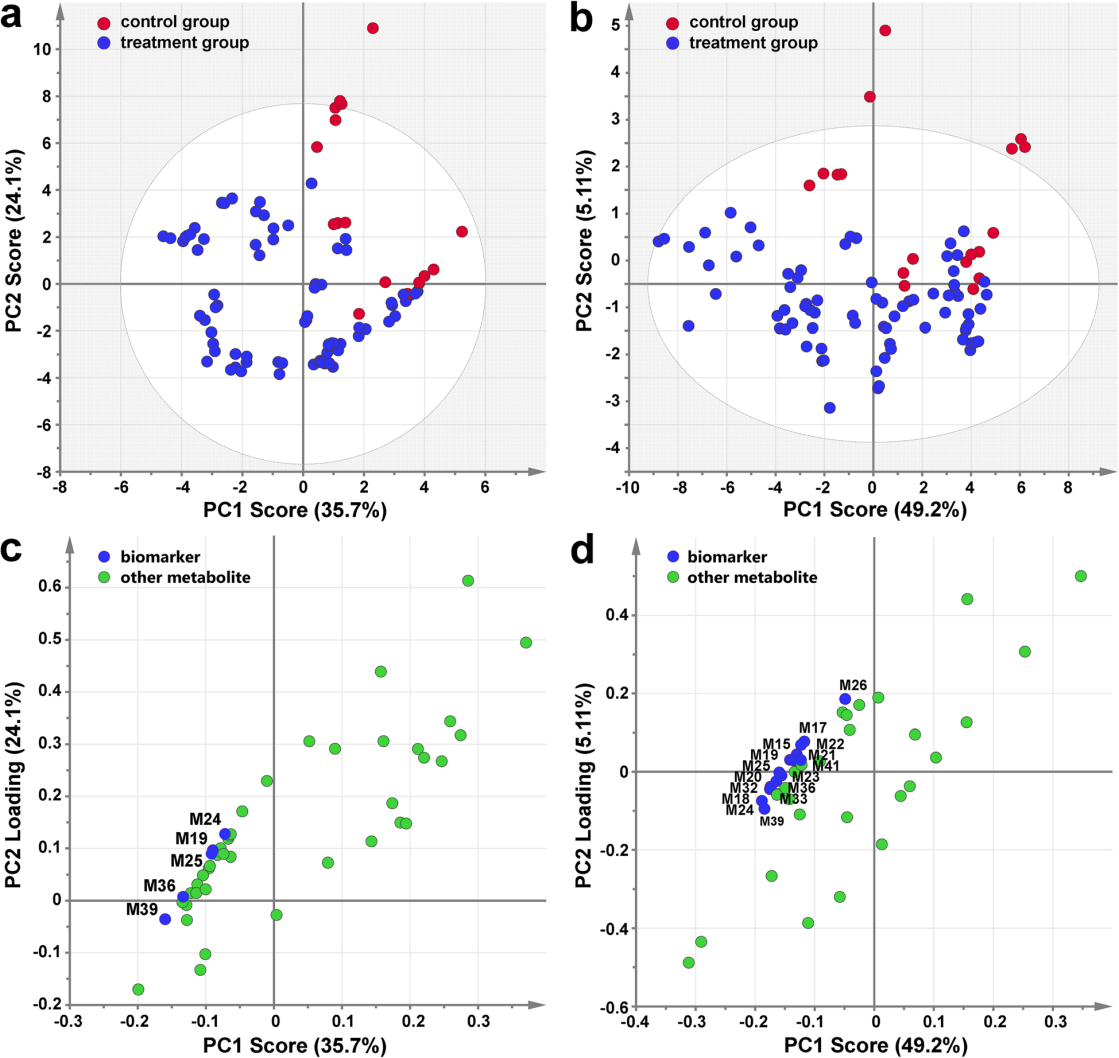
Identification of potential phenolic biomarkers via partial least squares-discriminant analysis (PLS-DA). Score scatter plot of the two-component PLS-DA model based on the concentration of detected phenolic compounds during the 2012 (**a**) and 2013 (**b**) seasons showing the discrimination between the control and cluster bagging-treated groups. Loading scatter plot of PLS-DA showing the important phenolic compounds (|Pcorr| value ≥0.8 and VIP value ≥1) during the 2012 (**c**) and 2013 (**d**) seasons that contributed significantly to the distinction between the control and cluster bagging-treated groups. M15, dephinidin-3-*O*-glucoside; M17, petunidin-3-*O*-glucoside; M18, peonidin-3-*O*-glucoside; M19, malvidin-3-*O*-glucoside; M20, dephinidin-3-*O*-(6-*O*-acetyl)-glucoside; M21, cyanidin-3-*O*-(6-*O*-acetyl)-glucoside; M22, petunidin-3-*O*-(6-*O*-acetyl)-glucoside; M23, dephinidin-3-*O*-(6-*O*-coumaryl)-glucoside; M24, peonidin-3-*O*-(6-*O*-acetyl)-glucoside; M25, malvidin-3-*O*-(6-*O*-acetyl)-glucoside; M26, petunidin-3-*O*-(6-*O*-caffeoyl)-glucoside; M32, peonidin-3-*O*-(*trans*-6-*O*-coumaryl)-glucoside; M33, malvidin-3-*O*-(trans-6-*O*-coumaryl)-glucoside; M36, myricetin-3-*O*-glucoside; M39, rutin; M41, laricitrin-3-*O*-glucoside

Partial least squares-discriminant analysis (PLS-DA) was performed to discriminate between cluster bagging treatments (T1, T3 to T11) and the control (T2) groups in the 2012 (Fig. 6a) and 2013 (Fig. 6b) growing seasons, respectively. Metabolites that were considered to be crucial were filtered by setting the absolute correlation coefficient (|Pcorr|) value ≥0.8 and the variable importance for the projection (VIP) value ≥1. Statistically, five important phenolic compounds, including three anthocyanin derivatives [malvidin-3-*O*-glucoside, malvidin-3-*O*-(6-*O*-acetyl)-glucoside, and peonidin-3-*O*-(6-*O*-acetyl)-glucoside] and two flavonol glycosides (myricetin-3-*O*-glucoside and rutin), were found to be competent as putative biomarkers with a conspicuous contribution to the discrimination between the control and treatment classes in the 2012 growing season (Fig. 6c). The PLS-DA model constructed from the phenolic profiles of grape berries in the next growing season confirmed the contribution of five biomarkers detected from the data for 2012 and several additional putative biomarkers in discriminating the two classes, most of which were anthocyanin derivatives and flavonol glycosides (Fig. 6d).

### Sunlight exclusion-induced transcriptome changes in the phenolic biosynthetic pathway

Transcriptome dynamics in berries from T1, T2, and T8-treated grapes during development were characterized to elucidate the molecular basis for light exclusion-affected phenolic biosynthesis. Approximately 571 Mb of clean reads were obtained from each sample using high-throughput RNA sequencing (RNA-Seq), with an average unique genome mapping ratio of 76.41% (Additional file 4: Table S4). DEGs were identified through comparisons of the FPKM values for each gene (fold-change ≥2 and divergence probability ≥0.8) between each developmental stage of the three groups, respectively, or between the control and bagging-treated samples at each developmental stage. Large-scale transcriptome reprogramming had occurred during the developmental processes of berries from both the control and treated groups. For cluster bagging-responsive DEGs, a total of 782 and 687 genes were differentially expressed in grape berries under T1 treatment from E-L 31 to E-L 38 stages and T8 treatment from E-L 36 to E-L 38 stages, respectively (Additional file 5: Figure S1). The Kyoto Encyclopedia of Genes and Genomes (KEGG) pathway enrichment analysis showed that ‘flavonoid biosynthesis’, ‘flavone and flavonol biosynthesis’, and ‘phenylpropanoid biosynthesis’ were significantly enriched by DEGs between T1 and T2-treated grape berries at E-L 35 stage, as well as by DEGs between T8 and T1/T2 treatments at E-L 36 stage (Additional file 6: Figure S2).

**Fig. 7:**
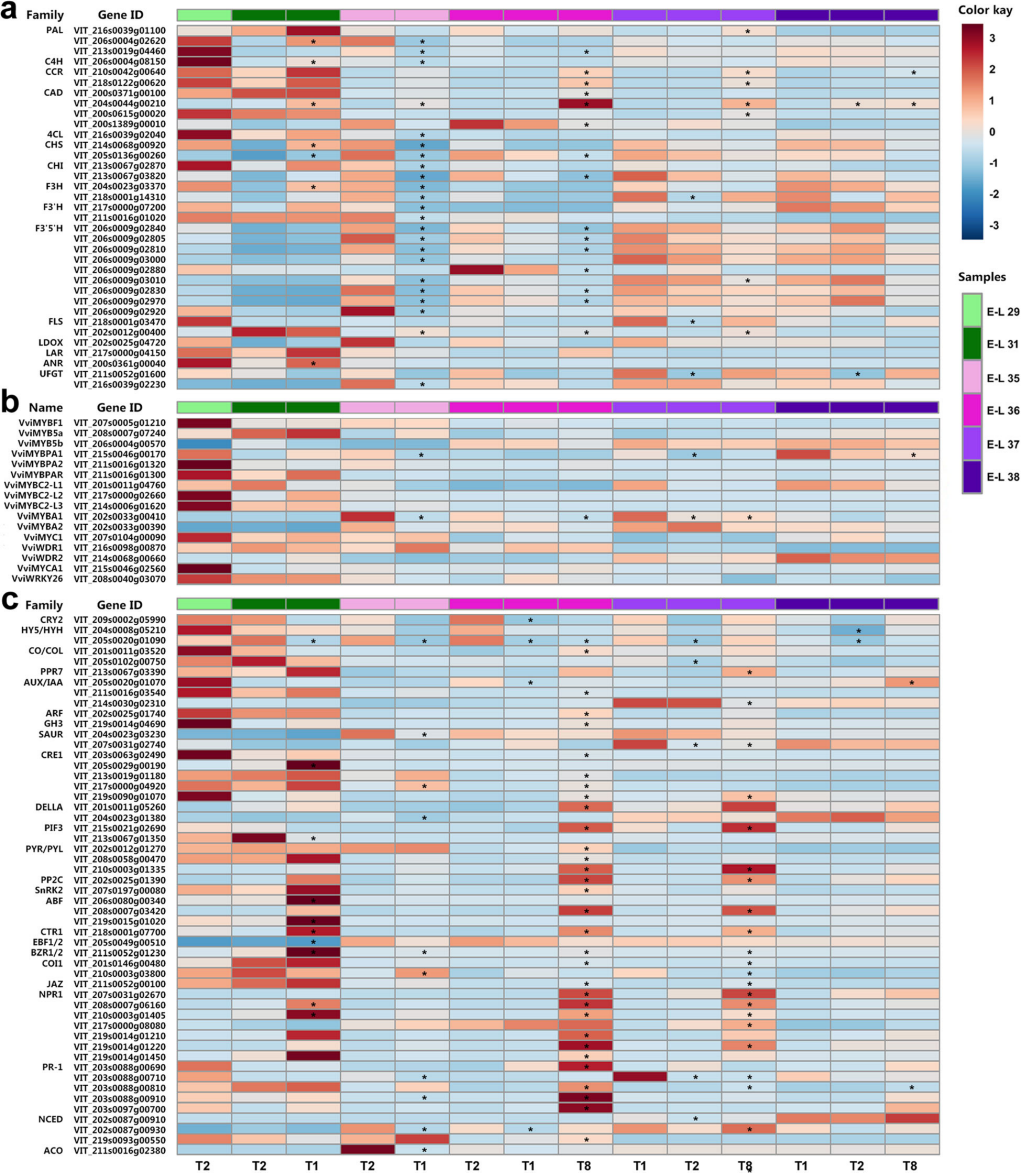
Transcription profile of DEGs encoding enzymes required for phenolic biosynthesis (**a**), genes encoding transcription factors involved in the regulation of phenolic metabolism (**b**) and DEGs encoding components of light and hormone signal transductions (**c**) in grape berries under cluster bagging treatments. T1: cluster bagging from 3 WAF until harvest; T2: control group; T8: cluster bagging at E-L 35 stage and bag removal at E-L 36 stage. PAL, phenylalanine ammonia-lyase; C4H, trans-cinnamate 4-monooxygenase; CCR, cinnamoyl-CoA reductase; CAD, cinnamyl-alcohol dehydrogenase; 4CL, 4-coumarate: CoA ligase; CHS: chalcone synthase; CHI, chalcone isomerase; F3H, flavanone 3-hydroxylase; F3’H, flavonoid 3′-hydroxylase; F3’5’H, flavonoid 3′,5′-hydroxylase; FLS, flavonol synthase; LDOX, leucoanthocyanidin dioxygenase; LAR, leucoanthocyanidin reductase; ANR, anthocyanidin reductase; UFGT, UDP-glucose: flavonoid 3-*O*-glucosyltransferase; CRY2, cryptochrome 2; HY5/HYH, ELONGATED HYPOCOTYL 5 (HY5)/HY5-homolog; CO/COL, CONSTANS/CONSTANS-like; PPR7, PSEUDO-RESPONSE REGULATOR 7; AUX/IAA, auxin-responsive protein IAA; ARF, auxin response factor; GH3, indole-3-acetic acid-amido synthetase; SAUR, small auxin-up RNA; CRE1, cytokinin receptor 1; DELLA, DELLA protein gibberellin-insensitive (GAI)-like; PIF3, phytochrome-interacting factor 3; PYR/PYL, pyrabactin resistance 1/PYR1-like; PP2C, protein serine/threonine phosphatase 2C; SnRK2, serine/threonine-protein kinase SRK2; ABF, ABA-responsive element binding factor; CTR1, serine/threonine protein kinase; EBF1/2, ethylene-insensitive protein 3 (EIN3)-binding F-box protein 1/2; BZR1/2, brassinosteroid resistant 1/2; COI1, coronatine-insensitive protein 1; JAZ, jasmonate ZIM domain-containing protein; NPR1, nonexpressor of pathogenesis-related genes 1; PR-1, pathogenesis-related protein 1; NCED, 9-*cis*-epoxycarotenoid dioxygenase; ACO, 1-aminocyclopropane-1-carboxylic acid oxidase. Each square in the heatmap located beside the gene names corresponds to the average FPKM value of the gene in each sample as illustrated in the legend. Genes with significant expression changes compared with the control group (fold change ≥2 and divergence probability ≥0.8) in each development stage are indicated by asterisks (*) in the squares

Pathway-specific transcriptome analysis indicated that the transcription of genes encoding enzymes in the general phenylpropanoid and central flavonoid biosynthetic pathways, including phenylalanine ammonia-lyase (PAL; VIT_206s0004g02620), *trans*-cinnamate 4-monooxygenase (C4H; VIT_206s0004g08150), chalcone synthases (CHSs; VIT_214s0068g00920 and VIT_205s0136g00260), flavanone 3-hydroxylase (F3H; VIT_204s0023g03370), and leucoanthocyanidin reductase (LAR; VIT_217s0000g04150) specifically involved in the biosynthesis of flavan-3-ols, was significantly upregulated in berries under T1 treatments compared with the control group at E-L 31 stage. By contrast, the transcription level of genes encoding PALs (VIT_206s0004g02620 and VIT_213s0019g04460), C4H (VIT_206s0004g08150), 4-coumarate: CoA ligase (4CL; VIT_216s0039g02040), CHSs (VIT_214s0068g00920 and VIT_205s0136g00260), chalcone isomerases (CHIs; VIT_213s0067g02870 and VIT_213s0067g03820), F3Hs (VIT_204s0023g03370 and VIT_218s0001g14310), and leucoanthocyanidin dioxygenase/anthocyanidin synthase (LODX/ANS; VIT_202s0025g04720) required for flavan-3-ol/anthocyanin biosynthesis and several flavonoid 3′-hydroxylase (F3’H) and flavonoid 3′,5′-hydroxylase (F3’5’H) members determining the hydroxylation pattern of the B-ring of flavonoids, was significantly downregulated in T1-treated grape berries at E-L 35 stage. In addition, a significant upregulation of the gene encoding cinnamyl-alcohol dehydrogenase (CAD; VIT_204s0044g00210) specifically involved in HCA biosynthesis at E-L 31, 35 and 38 stages, while a significant downregulation of the genes encoding flavonol synthase (FLS; VIT_218s0001g03470) specifically required for flavonol biosynthesis at E-L 37 and UDP-glucose: flavonoid 3-*O*-glucosyltransferases (UFGTs; VIT_211s0052g01600 and VIT_216s0039g02230) required for anthocyanin biosynthesis at E-L 35, 37, and 38 stages, was detected in T1-treated berries. In T8-treated grape berries, the transcript abundance of genes encoding PAL (VIT_213s0019g04460), CHS (VIT_205s0136g00260) and CHI (VIT_213s0067g03820) was significantly downregulated at E-L 36 stage, while cinnamoyl-CoA reductases (CCRs; VIT_210s0042g00640 and VIT_218s0122g00620) and CADs (VIT_200s0371g00100 and VIT_204s0044g00210) specifically required for HCA biosynthesis were significantly or moderately (fold-change ≥2 while divergence probability ≤0.8) upregulated during ripening (E-L 36, 37, and 38 stages). Moreover, the significantly downregulated expression of several members of the gene family encoding F3’5’H in grape berries at both E-L 36 and 37 stages after T8 treatment was in fair agreement with the higher ratio of 3′-OH/3′,5′-OH flavonoids (Fig. 7a; Additional file 7: Table S5).

The transcription profiles of genes encoding known grapevine phenolic biosynthesis-related positive/negative regulators belonging to the MYB (VviMYBF1, VviMYB5a, VviMYB5b, VviMYBPA1, VviMYBPA2, VviMYBPAR, VviMYBC2-L1, VviMYBC2-L2, VviMYBC2-L3, VviMYBA1, and VviMYBA2), bHLH (VviMYC1 and VviMYCA1), WDR (VviWDR1 and VviWDR2), and WRKY (VviWRKY26) transcription factor families were also analyzed in grape berries under sunlight exclusion. Consistent with the decreased accumulation of anthocyanins in light exclusion-treated grape berries, the expression level of the anthocyanin biosynthesis regulator VviMYBA1 (VIT_202s0033g00410) was significantly lower in berries from T1-treated grapes at E-L 35 and 37 stages and T8-treated grapes at E-L 36 and 37 stages compared with the control group. A significant downregulation of transcript abundance for the flavan-3-ol biosynthesis regulator VviMYBPA1 (VIT_215s0046g00170) was also detected in berries from T1-treated grapes at E-L 35 and 37 stages and T8-treated grapes at E-L 38 stage (Fig. 7b).

Investigation of relationships between transcriptome and metabolome data allowed us to identify additional structural genes that might contribute to the altered accumulation of phenolic compounds in grapes under different sunlight conditions (Additional file 8: Table S6). Correlation analysis based on Pearson’s coefficient revealed that the transcription of genes encoding four CCR members (VIT_206s0004g02380, VIT_206s0004g02370, VIT_213s0067g00620 and VIT_203s0038g04220) and two CAD members (VIT_200s0615g00030 and VIT_200s0346g00080) was significantly positively correlated (r ≥ 0.7 and *p*-value ≤0.001) with the concentration of corresponding HCAs among samples. Moreover, significant positive correlations were also observed between the transcript changes in genes encoding LAR (VIT_201s0011g02960) and anthocyanidin reductase (ANR; VIT_215s0046g01170), two enzymes required for the last step of the synthesis of flavan-3-ols, as well as FLS (VIT_218s0001g03470) and the bifunctional UDP-glucose/UDP-galactose: flavonoid-3-*O*-glucosyltransferase/galactosyltransferase (GT6; VIT_202s0025g02920) involved in the initial synthesis and glycosylation of flavonols, with an altered accumulation of the corresponding flavonoids in sunlight exclusion-treated grape berries, respectively.

To validate the RNA-Seq results, the relative expression levels of 18 genes encoding enzymes from phenolic biosynthetic pathways (VviPAL1, VviPAL2, VviPAL3, VviPAL4, VviCHS1, VviCHS2, VviCHI1, VviF3H1, VviF3H2, VviF3’H, VviF3’5’H, VviFLS1, VviFLS2, VviFLS3, and VviFLS4), as well as three pathway-related transcription factors (VviMYBF1, VviMYBPA1, and VviMYBA1), were determined in each sample using qPCR. Transcripts of two housekeeping genes *VviUbiquitin1* (VIT_216s0098g01190) and *Vviβ-actin* (VIT_204s0044g00580) were used as the endogenous control for normalization due to their relatively constant expression in grape berries throughout development and/or under various stress conditions [54, 55]. The expression profile of these genes in berries from light exclusion-treated and control grapes during development obtained from an independent qPCR evaluation were significantly positively correlated [Pearson correlation coefficient (r) = 0.71, *p* = 0.001] with those from the transcriptomes (Additional file 9: Figure S3), demonstrating the high reproducibility of the results from RNA-Seq.

### Involvement of light and hormone signaling pathways

To understand the responses of grape berries to excluded light irradiation directly affected by cluster bagging treatments, we identified all possible genes encoding receptors, downstream components and functional proteins associated with light and hormone signal perception and transduction which were differentially expressed in grape berries after sunlight exclusion treatment (Fig. 7c; Additional file 12: Table S7). No significant transcriptional changes in the members of three classes of photoreceptors, phytochrome (PHY), phototropin (PHOT), and UV resistance locus 8 (UVR8), were detected in grape berries under dark conditions. By contrast, the transcript abundances of a blue light receptor cryptochrome 2 (CRY2; VIT_209s0002g05990) and two positive regulators located downstream of the photoreceptors, HY5 (VIT_204s0008g05210) and HY5-homolog (HYH; VIT_205s0020g01090), were significantly or moderately downregulated in grape berries during light exclusion and retuned to a normal level after bag removal.

Among those genes associated with plant hormone signal transduction pathways, a total of 46 genes encoding signaling components of different hormones, including auxin, cytokinin (CTK), gibberellin (GA), abscisic acid (ABA), ethylene (ETH), brassinosteroid (BR), jasmonic acid (JA), and salicylic acid (SA), were significantly differentially expressed between cluster bagging-treated and untreated grape berries (Fig. 7b). Notably, the transcription of genes encoding auxin-responsive protein IAA (AUX/IAA; VIT_211s0016g03540) and auxin response factor (ARF; VIT_202s0025g01740) involved in auxin signal transduction was significantly enhanced in T8-treated berries at E-L 36 stage. Transcript abundances of some CTK receptor CRE1 (VIT_203s0063g02490, VIT_205s0029g00190, VIT_213s0019g01180, VIT_217s0000g04920 and VIT_219s0090g01070) and brassinosteroid resistant 1/2 (BZR1/2; VIT_211s0052g01230) members involved in BR signal transduction were significantly upregulated in T1-treated berries at E-L 31 and 35 stages and in T8-treated berries at E-L 36 and 37 stages. Significantly upregulated transcription of genes encoding pyrabactin resistance 1/PYR1-like (PYR/PYL; VIT_202s0012g01270, VIT_208s0058g00470 and VIT_210s0003g01335) receptor proteins and ABA-responsive element binding factor (ABF; VIT_206s0080g00340, VIT_208s0007g03420 and VIT_219s0015g01020) involved in ABA signal transduction, nonexpressor of pathogenesis-related genes 1 (NPR1; VIT_207s0031g02670, VIT_208s0007g06160, VIT_210s0003g01405, VIT_217s0000g08080, VIT_219s0014g01210, VIT_219s0014g01220 and VIT_219s0014g01450) involved in SA signal transduction, and serine/threonine protein kinase CTR1 (VIT_218s0001g07700) involved in ETH signal transduction, was also detected in T1-treated berries at E-L 31 stage and in T8-treated berries at E-L 36 and 37 stages.

### Activity analysis of *VviFLS4* and its light-responsive promoter

**Fig. 8:**
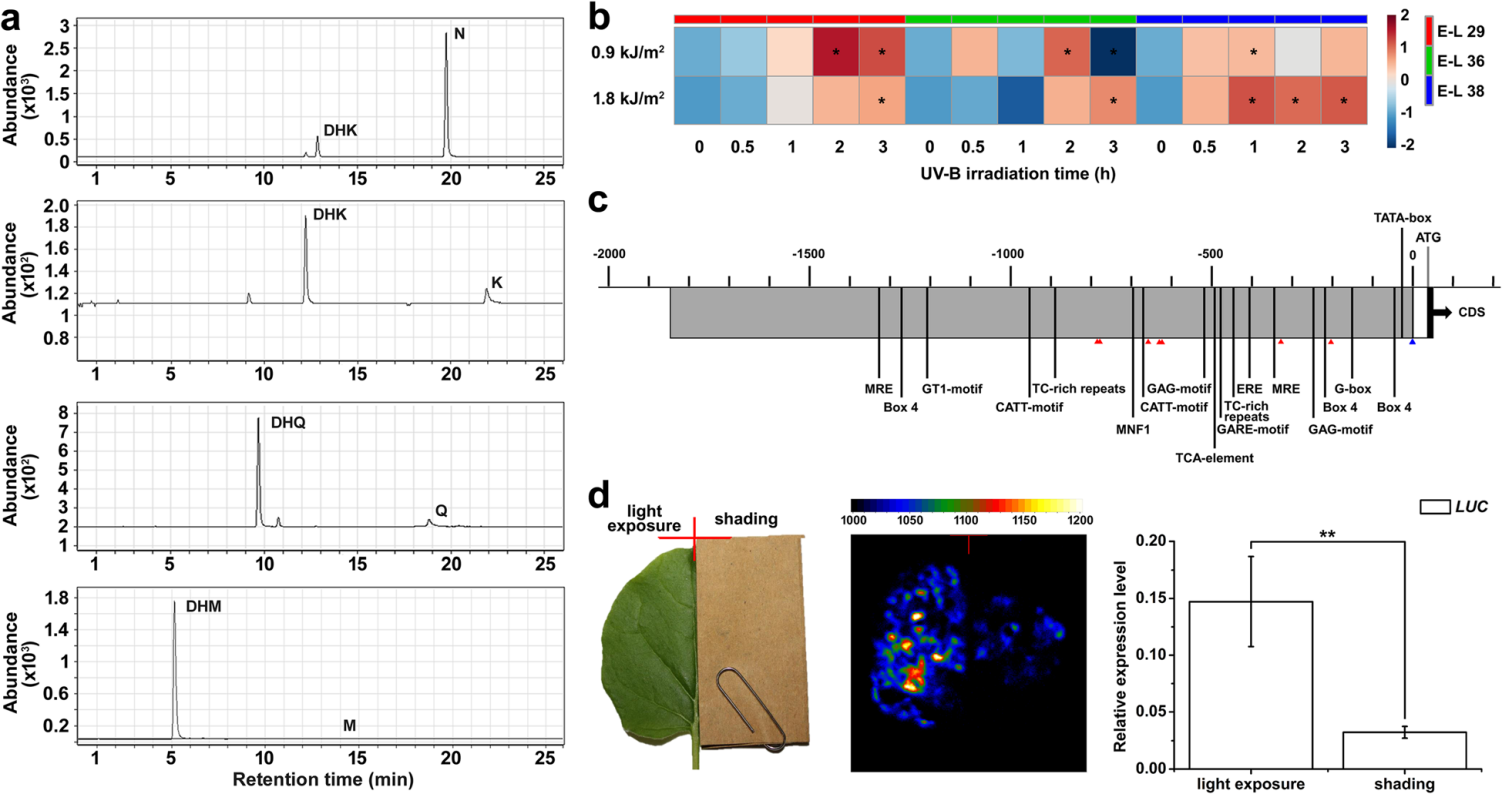
Molecular characterization and functional analysis of VviFLS4. (**a**) HPLC-MS analysis of reaction products generated from the incubation of VviFLS4-His recombinant protein with the substrates naringenin (N), dihydrokaempferol (DHK), dihydroquercetin (DHQ), and dihydromyricetin (DHM). K: kaempferol; Q: quercetin; M: myricetin. (**b**) Quantitative analysis of *VviFLS4* gene expression in the skin of detached grape berries under UV-B radiation treatments. Each square in the heatmap corresponds to the relative expression of the gene as illustrated in the legend. Significant expression changes of *VviFLS4* gene after treatment compared are indicated by asterisks (*) in the squares. (**c**) Schematic representation of the *cis*-acting elements predicted in the promoter region of the *VviFLS4* gene. The promoter region and 5*′*-untranslated region are differentiated by grey and white boxes, respectively. The CAAT-boxes are indicated by red triangles. Values above boxes represent the number of nucleotides, starting from the putative transcription start site indicated by the blue triangle. CDS, coding sequence. (**d**) Transient expression analysis of *VviFLS4* gene promoter activity in tobacco leaves. An equal amount of p*VviFLS4-LUC* construct was transformed into the left and right sides of tobacco leaves. The right sides of the infiltrated leaves were shaded with masking for dark shading treatments. Left panel: Schematic diagram of the leaf shading treatment. Middle panel: Fluorescence imaging. Right panel: Relative transcription level of the *LUC* gene in the experimental and control groups (^**^ indicates significance at the 0.01 level)

The observation that light exclusion-regulated transcriptional changes in *VviFLS4* (VIT_218s0001g03470) appear to be significantly positively correlated with the drastically decreased flavonol biosynthesis in grape berries grown in darkness raised the hypothesis that VviFLS4 might be a key enzyme required for the light-responsive biosynthesis of flavonols in grape berries. A full-length coding sequence of *VviFLS4* was obtained from the cDNA library of Cabernet Sauvignon berries constructed previously [56]. This gene encodes a predicted polypeptide of 335 amino acids with a calculated molecular weight of 37.94 kDa and a theoretical isoelectric point (pI) of 6.0. The deduced VviFLS4 protein contains a typical α-subunit of prolyl-4-hydroxylase belonging to the 2OG-Fe (II) dioxygenase superfamily at the C-terminal end (Additional file 10: Figure S4). HPLC-MS analysis demonstrated that the recombinant purified VviFLS4-His protein (Additional file 11: Figure S5) could enzymatically transform naringenin into dihydrokaempferol (DHK), DHK into kaempferol, and dihydroquercetin (DHQ) into quercetin, respectively, but it was unable to catalyze the conversion of dihydromyricetin (DHM) to myricetin in vitro (Fig. 8a). Moreover, the transcriptional activity of *VviFLS4* in detached grape berries at three different developmental stages (E-L 29, 36 and 38) was significantly increased after both high (1.8 kJ/m^2^) and low doses (0.9 kJ/m^2^) of supplemental UV-B irradiation treatments (Fig. 8b).

To better understand the light-regulated transcriptional activity of the *VviFLS4* gene, a 1878-bp fragment upstream of the initiation codon (ATG) of *VviFLS4* was isolated from the genomic DNA of Cabernet Sauvignon grapes. Sequence analysis of the 5′-regulatory region revealed the existence of several putative light-responsive *cis*-acting elements upstream of the predicted TATA-box and transcription start site, which includes CATT-motifs (− 674 bp and − 958 bp), G-box-related element (− 150 bp), GAG-motif (− 514 bp), MNF1 (− 706 bp), GT1-motif (− 1207 bp), three Box 4 (− 49 bp, − 227 bp, and − 1257 bp), and two MYB response elements (MRE, − 347 bp and − 1334 bp) (Fig. 8c). *Agrobacterium*-mediated transient expression of a 1350-bp promoter fragment fused to the promoterless *luciferase* (*LUC*) reporter gene was examined in tobacco (*Nicotiana benthamiana* L.) leaves. The results showed that the LUC signal was markedly suppressed (*p* ≤ 0.01) in the infiltrated leaves by shading treatment (Fig. 8d), suggesting that light exclusion could reduce the activity of the *VviFLS4* promoter in vivo.

### Identification of co-expressed transcriptional regulators and modules associated with sunlight-responsive phenolic accumulation

A genome-wide co-expression analysis between metabolic pathway-related DEGs and genes encoding transcription factors was employed to systemically explore additional putative regulators that control phenolic biosynthesis in grape berries under sunlight exclusion conditions (Additional file 13: Table S8). Transcriptional changes in a total of 158 transcription factor genes belonging to 38 families among samples showed a significant positive correlation (Pearson Correlation Coefficient ≥ 0.9 and *p*-value ≤0.01) with those of 9 key structural genes encoding PAL, CADs, CCRs, ANR, FLS, and UFGT. Remarkably, members of bHLH, MYB, WRKY, NAC (no apical meristem, ATAF 1,2, and cup-shaped cotyledon 2), ethylene-responsive factor (ERF), homeodomain-leucine zipper (HD-Zip), and MADS (MCM1, agamous, deficiens, and serum response factor)-box TF families were found to be the most abundant significantly positively co-expressed transcription factors.

**Fig. 9:**
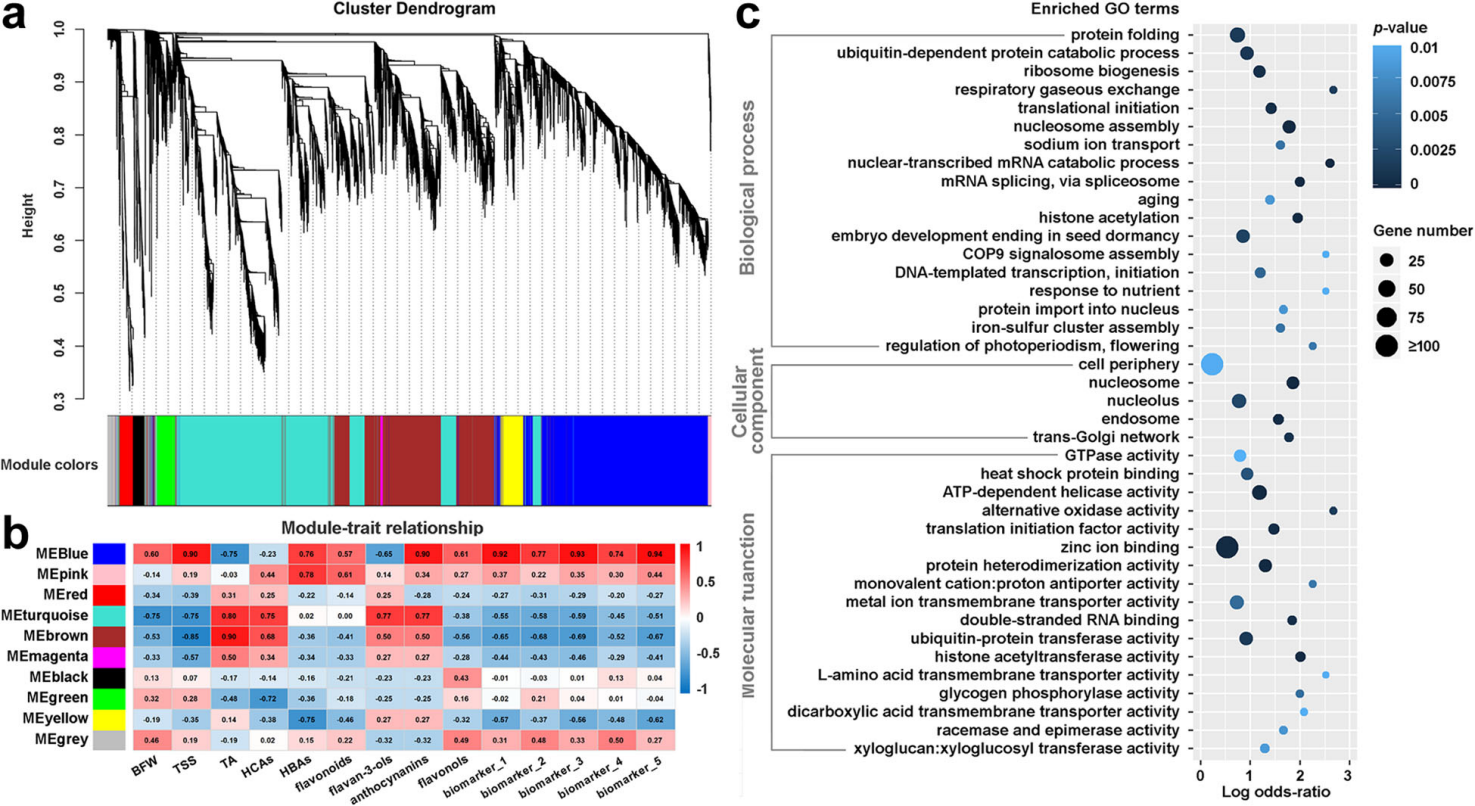
Weighted gene co-expression network analysis (WGCNA) of DEGs induced by cluster bagging treatments and functional analysis of DEGs associated with the accumulation of phenolic compounds. (**a**) Hierarchical cluster tree showing co-expression modules identified by WGCNA. Each leaf in the tree is one gene. The major tree branches constitute 10 modules labeled by different colors. (**b**) Module–trait association. Each row corresponds to a module. Each column corresponds to a physiological/metabolic parameter. The color of each cell at the row–column intersection indicates the correlation coefficient between the module and the physiological/metabolic parameter. A high degree of correlation between a specific module and the physiological/metabolic parameter is indicated by red (positive) or blue (negative). BFW, berry fresh weight; TSS, total soluble solids; TA, titratable acidity; HCAs, hydroxycinnamic acids; HBAs, hydroxybenzoic acids. Biomarkers 1 to 5 represent malvidin-3-*O*-glucoside, peonidin-3-*O*-(6-*O*-acetyl)-glucoside, malvidin-3-*O*-(6-*O*-acetyl)-glucoside, myricetin-3-*O*-glucoside, and rutin, respectively. (**c**) Gene Ontology enrichment analysis of DEGs in the blue module

Weighted gene co-expression network analysis (WGCNA) of the DEGs between cluster bagging treatments and the control groups during the same developmental stages was performed to cluster gene modules and identify functional pathways potentially associated with grape berry development and phenolic accumulation under sunlight exclusion. Co-expression modules are defined as clusters of highly interconnected genes with high correlation coefficients. After the test of topology for the hierarchical clustering of samples and analysis of network topology for various soft-thresholding powers (setting as 14; Additional file 14: Figure S6), we ultimately identified 10 distinct modules with gene numbers ranging from 38 (magenta) to 3153 (turquoise) (Fig. 9a). Notably, the blue co-expression module comprised DEGs that were significantly (*p*-value ≤0.01) positively corrected with the berry fresh weight, the content of TSS, HBAs, total flavonoids, anthocyanins, and flavonols, as well as the phenolic biomarkers (Fig. 9b), indicating putatively important roles for this module in sunlight-affected berry development and phenolic biosynthesis. Gene Ontology (GO) enrichment analysis revealed that the genes in the blue module were significantly (*p*-value ≤0.01 and FDR ≤ 0.05) enriched in the biological processes of ‘nucleosome assembly’, ‘nuclear-transcribed mRNA catabolic process’, ‘mRNA splicing via spliceosome’, and ‘histone acetylation’, the cellular component of ‘nucleosome’, and the molecular functions of ‘ATP-dependent helicase activity’, ‘zinc ion binding’, ‘protein heterodimerization activity’, and ‘histone acetyltransferase activity’ (Fig. 9c). In addition, some genes in this module were largely related to the initiation of transcription/translation, protein folding and import into the nucleus, and the ubiquitin-dependent protein catabolic process.

## Discussion

As a physically protective field practice, fruit bagging for a specific period prior to the harvest has been extensively used in several fruit crops to improve fruit color and quality, reduce mechanical fruit damage, sunburn of the fruit peel, and presence of pesticide residues, and also protect fruit from pathogens, pests, and birds [57–61]. Numerous efforts have focused on the influence of cluster bagging or artificial shading with light exclusion boxes during different developmental stages concerning berry ripening, phenolic accumulation and the expression of phenolic biosynthesis genes in grapes [7, 14, 15, 23, 24, 30, 34, 62]. A recent genome-wide transcriptome analysis of two red grape cultivars also advanced our knowledge regarding the mechanisms of light-controlled anthocyanin synthesis [9]. However, many gaps remain in the molecular events of the transduction pathway underlying the process of grape phenolic biosynthesis in response to sunlight [44]. To further elucidate the transcriptional regulatory circuits controlling phenolic biosynthesis under sunlight exclusion, we performed a comprehensive integrative analysis of physiological, metabolic, and transcriptomic responses of grape berries to cluster bagging treatments. Our results showed that bagging treatment induced solar radiation interception and that temperature increases had limited effects on the quality attributes (weight, TSS, and TA) of grape berries at harvest (Fig. 2), thus minimizing the possible impacts of shading on grape production and improving the ability to focus on breeding programs aiming to modify berry phenolic pattern [23]. Similar changes in berry ripening progression and phenolic compounds accumulation under cluster bagging treatments were observed between the two consecutive growing seasons, which might be due to the similar meteorological conditions during berry development between years (Additional file 1: Table S1). Particularly, cluster bagging during early developmental stages and re-exposure of bagged fruits to sunlight after véraison resulted in significantly elevated flavan-3-ol and flavonol concentrations in shaded berries, while bagging after véraison significantly inhibited skin anthocyanin accumulation in both years (Fig. 4). These phenomena were associated with light-responsive transcriptional changes in enzymes and regulators of the phenolic biosynthetic pathway (Fig. 7), as well as genes involved in mRNA processing and protein modification (Fig. 9c). The expression of several components of light, temperature and hormone signal transductions were also affected by sunlight exclusion (Fig. 7c), which may play a critical role in mediating light-responsive phenolic biosynthesis in grapes [17, 63].

Sunlight has been demonstrated, in extensive studies, to be one of the most relevant factors controlling phenolic metabolism in plants, particularly the biosynthesis of flavonoids [44]. Sunlight exclusion was less effective for phenolic acids than for flavonoids, although differences in HCA contents and the transcription of corresponding structural genes in grape berries during ripening were found between treatments (Figs. 3and 7b). These results are in accordance with previous findings in both red and white grape cultivars showing that HCAs respond only slightly to leaf removal before maturity [21, 22], suggesting a limited role of solar radiation in regulating phenolic acid biosynthesis. Oligomers and polymers of flavan-3-ols are the most abundant flavonoids in grape berries during early developmental stages, accumulating mainly before véraison and then declining with increasing maturity. Previous studies have shown that shaded grapes reach a much lower maximum of skin flavan-3-ol content than exposed grapes; however, the decrease in flavan-3-ol during berry ripening is greater in exposed grapes such that the levels are virtually the same in shaded and exposed berries at harvest [14, 18, 28]. In the present study, the peak content of skin flavan-3-ols in the control group occurred around 3 WAF and thereafter, gradual decreased until harvest (Fig. 4b). A similar pattern for skin flavan-3-ol accumulation in grape berries during development was observed in our previous experiments carried out in another field under the same climatic conditions, which was mainly due to the excessive solar radiation of these regions [11, 64]. Thus, the enhanced biosynthesis of flavan-3-ol mediated by the upregulated transcription of genes encoding key the synthetic enzymes LAR and ANR (Fig. 7a), accompanied with a reduced decrease in flavan-3-ols in shaded grapes during the early development stage, contributed to the improvement in skin flavan-3-ol levels in early shaded berries at harvest.

Anthocyanins and flavonols have been shown, in numerous studies, to be the phenolic compounds in grape berries and many other fruit species that are most drastically affected by changes in light conditions [18, 44], as confirmed by the light-responsive anthocyanin and flavonol biomarkers identified in our experiments (Fig. 6c, d). It is well known that anthocyanin biosynthesis in grape berry skin starts at véraison and continues during the ripening phase. Both light intensity and qualities, especially UV-B and blue light, have been associated with the regulation of anthocyanin biosynthesis [65]. HY5 protein acts downstream of the blue light receptor CRY and mediates blue-light induced anthocyanin accumulation in plants [66, 67], and its homologous protein, HYH, has also been proposed to be the transcriptional regulator of several *MYBA* genes controlling anthocyanin synthesis in grapes [68]. Regarding the accumulation of skin anthocyanins under shading at different stages (Fig. 4c), sunlight exclusion retarded and suppressed their biosynthesis around the time of véraison and at late stages of ripening, in accordance with previous findings in Cabernet Sauvignon and several other red grape cultivars [23, 30–34]. The severely suppressed expression of genes encoding the positive transcriptional regulator VviMYBA1 and one of its direct downstream target gene encoding the UFGT enzyme catalyzing the last biosynthetic step in anthocyanin synthesis [69, 70] in response to light exclusion, was consistent with the downregulated transcript abundance of the blue light receptor *CRY2* and signaling components *HY5/HYHs*, which are responsible for shading-inhibited anthocyanin biosynthesis.

Flavonols are reported to be highly effective scavengers of free radicals, such as reactive oxygen species (ROS), during sunlight-induced photo-oxidation, additionally possessing the ability to act as UV-B protectants despite the negligible low content of these compounds found in developing grape berries [44, 53, 71]. Previous studies have demonstrated that light-induced flavonol accumulation in grapes appears to be strongly correlated with enhanced transcription of genes encoding VviMYBF1 (also known as VviMYB12) and the key enzyme VviFLS4 (also known as VviFLS1) for flavonol biosynthesis [35, 45, 54, 72]. In the present study, we confirmed the FLS activity of VviFLS4 protein using an in vitro enzymatic assay, and we also observed UV-B radiation induction of VviFLS4 gene expression in detached grape berries and an inhibitory effect of shading on its promoter activity (Fig. 8). A substantial decrease in the content of flavonols along with rapid light responsiveness of transcripts encoding HY5/HYHs in the UV-B signaling cascades and key enzyme VviFLS4 in shading berries [48] demonstrated UV-B radiation-responsive flavonol biosynthesis in grapes in a development-independent manner (Figs. 4, 7 and 9d, b). The expression of the gene encoding VviMYBF1 specifically responsible for flavonol biosynthesis in grape berries [35, 45], however, showed no significant response to shading treatments throughout berry development (Fig. 7b), although *VviMYBF1* was found to be directly targeted by HY5/HYH and responsive to solar UV-B and visible light radiations in previous studies [13, 27, 48, 73]. Thus, further studies on the identification of novel transcriptional regulators of light-modulated *VviFLS4* gene expression, will be required to fully clarify the transcriptional regulatory mechanism.

The signaling of endogenous plant hormones, such as auxin, CTK, ABA, GA and BR, interact with light/darkness signaling pathways controlling diverse growth and developmental processes in plants [74–76]. Exogenous application of ABA, ETH and BR significantly promotes grape berry ripening and enhances the accumulation of specific flavonoid compounds, mainly anthocyanins, flavonols and HCAs [32, 77–79]. In contrast, auxin (2,4-dichlorohenoxyacetic acid and naphthaleneacetic acid) treatments delay the ripening progression and inhibit the expression of anthocyanin biosynthetic genes and accumulation of anthocyanins in grape berries [32, 80]. More recently, synergistic effects of exogenous EBR and light treatments on the induction of PAs and anthocyanin synthesis have been found [62]. Enhanced signal transductions of auxin, CTK, ABA, BR, SA and ETH have been observed in response to both cluster bagging (T1-treated berries at E-L 31 stage and T8-treated berries at E-L 36 stage) and bag removal (T8-treated berries at E-L 37 stage) treatments (Fig. 7c), suggesting the putative involvement of multiple hormone signaling pathways in the response of grape berries to light changes. Several hormone signal transduction components, particularly those involved in auxin, ethylene, and ABA signaling, have been previously observed among the positive and negative molecular biomarkers of the onset of ripening in grapevine [81]. Our data showed that the delayed ripening process of T8-treated grape berries, as indicated by lower TSS content and higher TA during véraison (Fig. 2b, c), coincided well with sunlight exclusion-induced up-regulation of auxin-related negative biomarkers (*AUX/IAA* and *ARF*) compared with those in the control group during berry development (Fig. 7c). However, the sunlight exclusion/exposure-triggered activation of hormone signals seems to have little causal relationship with the changes in phenolic accumulation, indicating that hormone signaling may play a limited role in mediating the light/darkness-regulated grape phenolic biosynthesis process.

To better understand the regulatory process of grape phenolic metabolism in the dark, we performed genome-wide co-expression network analysis and identified a significant number of candidate TFs that were directly or indirectly involved in the transcriptional activity of light-responsive phenolic synthesis-related genes (Additional file 13: Table S8), as well as unique gene sets/modules that were highly associated with the dynamic accumulation of phenolics (Fig. 9). It is interesting to note that the most abundant significantly positively correlated TF families, including bHLH, MYB, WRKY, NAC, and MADS-box, have also been identified as potential regulators associated with the phenolic metabolic process in sunlight-exposed grape berries [13]. The involvement of MYB-bHLH-WD40 (MBW) ternary protein complexes in the initiation and fine-tuned transcriptional regulation of flavonoid synthesis has been commonly demonstrated in a wide range of higher plants, including grapevine (Fig. 7b) [42, 82, 83]. Additional regulators of plant phenolic metabolism belonging to the WRKY [*Arabidopsis* TTG2 and grapevine VviWRKY26 (VIT_208s0040g03070.2)], NAC [*Arabidopsis* ANAC078, peach BLOOD and grapevine VviNAC29 (VIT_201s0026g02710)], HD-ZIP [grapevine VviATHB6 (VIT_207s0191g00180) and VviATHB13 (VIT_201s0026g01950)], and MADS-box (*Arabidopsis* TT16) TF families have also been functionally characterized, some of which can act directly or indirectly on MBW complex activity [83–88]. However, the molecular functions and regulatory roles of these TFs on co-expressed phenolic biosynthetic genes in grape berries remain to be determined. Genome-wide epigenetic changes in plants, such as DNA methylation, histone modifications and nuclear architecture, have been widely reported to be involved in gene regulation during developmental processes, tissue differentiation and stress responses [89–91]. GO enrichment analysis of phenolic-associated gene modules highlight the putative correlation between phenolic metabolism in response to different light regimes and the expression of genes involved in epigenetic regulation (nucleosome assembly and histone acetylation) and genetic information (mRNA and protein) processing in grape berries, which might provide new insight into molecular mechanisms of berry phenolic metabolism under light/darkness.

## Conclusions

We performed an integrated transcriptomics and pathway-level metabolomics analysis to investigate the effect of cluster bagging during various developmental stages on grape phenolic metabolism. The results indicate that cluster bagging during early-developmental stages significantly elevated the content of flavan-3-ols and flavonols in grape berries at harvest, whereas bagging after véraison inhibited anthocyanin accumulation, which are coincided with transcriptional changes in several members of light signaling components, transcription regulators, and enzymes. Moreover, gene-gene/metabolic network analyses suggest a high positive correlation of genetic information processing and epigenetic regulation with phenolic metabolism in grapes. In summary, our findings present a first step into understanding of the transcriptional regulatory cascades of grape phenolic biosynthesis under light/darkness, and also provide practical guidance for improving grape features. Upcoming work will aim to characterize the identified individual key genes and biological processes to understand their functions in light-regulated phenolic biosynthesis in grapes.

## Methods

### Plant materials and treatments

The field experiments were conducted on Cabernet Sauvignon vines grown in a commercial vineyard in Manas Country, China (44°17′ North, 86°12′ East) during two consecutive growing seasons (2012 and 2013), with the permission of the CITIC Guoan Wine Co. Ltd. (China). The own-rooted vines in this vineyard were planted in 2000, arranged in north–south rows with a 2.5 m × 1 m vine spacing and equipped with a furrow irrigated system, spur-pruned (15 nodes per linear meter) and trained using a modified Vertical-Shoot-Positioned (M-VSP) trellis system [13]. Nutrition and pest managements were carried out according to industry standards for this cultivar and the region as described previously [92]. For the cluster bagging treatment, an individual cluster was placed into two-layer Kraft paper bags (36 × 36 cm), yellow exterior layer with the black interior layer coated with wax), with a bent straw to maintain ventilation inside the bag (Fig. 1b). Each treatment was applied to 15 vines randomly selected from both south and north sites of the vineyard, with three biological replicates. Canopy microclimatic conditions for both bagged and un-bagged grapes were monitored as described previously [13]. The meteorological data were gathered from the local meteorological administration (Additional file 1: Table S1).

Berries from each group were collected at E-L 29, 31, 33, 35, 36, 37, and 38 stages during two consecutive seasons. The data of harvest (E-L 38 stage) was set at approximately 16 WAF according to the commercial harvest time of the vineyard in both years. Berries samples for physiological measurements, UV-B irradiation treatments, metabolite determination and transcriptional analysis were prepared according to the method described by Sun et al. [13]. Physiological indexes such as berry weight, TSS and TA were determined as described previously [13]. One-way ANOVA followed by the Duncan’s new multiple range test was calculated using SPSS 20.0 for windows (SPSS Inc., Chicago, IL, USA) to determine the physiological differences among samples. UV-B irradiation treatments of detached grape berries at E-L 29, 36 and 38 stages were performed as described previously [93].

### Extraction and high performance liquid chromatography-mass spectrometry analysis

Extraction of grape berry phenolic acids and skin flavan-3-ols, anthocyanins and flavonols was based on the method described by Sun et al. [13]. Qualitative and quantitative analyses of phenolic compounds were carried out on an Agilent 1100 series high performance liquid chromatography-mass spectrometry detector (HPLC-MSD) trap VL (Agilent, Santa Clara, CA, USA), equipped with a diode array detector (DAD) for phenolic acids, flavan-3-ols and anthocyanins, or a variable wavelength detector for flavonols as described previously [13]. The statistical significance of differences in the concentrations of phenolics among samples was determined by one-way ANOVA as mentioned above. PCA and PLS-DA of the metabolic profiles were performed using SIMCA 13.0 software (Umetrics, Umeå, Sweden).

### RNA isolation, sequencing and data analysis

Grape Berries from the T1 treatment at E-L 31, 35, 36, 37, and 38 stages, T8 treatment at E-L 36, 37, and 38 stages, and control group at E-L 29, 31, 35, 36, 37, and 38 stages during the 2012 growing season were selected to conduct the transcriptome profiling analysis based on the physiological and metabolic results. Total RNAs for RNA-Seq analysis were isolated and purified from deseeded berries according to Sun et al. [13]. The concentration and purity of the extracted RNA samples were established using a Nanodrop 2000 spectrophotometer (Thermo Fisher Scientific Inc., Wilmington, DE, USA), and the integrity of the RNA was analyzed with an Agilent 2100 Bioanalyzer (Agilent, Santa Clara, CA, USA). After quality assessment, poly(A) mRNA was isolated for each of the RNA samples and sequenced using an Illumina Hiseq™2000 sequencer (Illumina Inc., San Diego, CA, USA) with a 50-bp single read module. The sequencing reads were aligned against the V2 version of the *V. vinifera* (PN40024) genome using the alignment software Bowtie [94], allowing a maximum of two nucleotide mismatches. The RNA-Seq data were deposited into the NCBI Gene Expression Omnibus (GEO, accession no. GSE129916). The transcript abundance of each gene was calculated using the FPKM (expected fragments per kilobase of transcript per million fragments mapped) method. The differentially expressed genes [DEGs, log2(−fold change) ≥ 1 and divergence probability ≥0.8] between samples were identified using functions of the R package NOISeq [95]. KEGG pathway enrichment analysis of DEGs was performed as described previously [11]. Pathways with a corrected *p*-value and Q-value ≤0.05 were defined as significantly enriched. Pearson correlation evaluation was conducted with the R package Hmisc using the rcorr function. Gene co-expression and module analyses were performed using the R package WGCNA [96]. GO functional enrichment analysis of each module was performed using the online software GOEAST (http://omicslab.genetics.ac.cn/GOEAST/). The GO terms with a corrected *p*-value ≤0.05 were defined as significantly enriched.

### Quantitative real-time PCR

Preparation of cDNA, quantitative real-time PCR (qPCR), and the relative expression calculation were performed according to Sun et al. [11]. Two housekeeping genes encoding ubiquitin1 and β-actin in grapevine were selected as endogenous controls. The gene-specific primers are listed in Additional file 15: Table S9. Three biological replicates and three technical replicates were run per sample. Significance of differences in gene expression among samples was determined by one-way ANOVA as mentioned above.

### Amplification and analysis of the coding sequence and promoter region

The complete coding sequence and 5*′*-untranslated region (5*′*-UTR) of the *VviFLS4* gene was amplified from the cDNA library of Cabernet Sauvignon berries previously constructed by our lab [56]. Gene-specific primers designed according to our de novo assembled reference transcriptome of Cabernet Sauvignon [11] are listed in Additional file 15: Table S9. The PCR products were cloned into the pGM-T vector (TIANGEN, Beijing, China), transformed into *Escherichia coli* DH5α and sequenced. The gene structure was predicted using the FGENESH program from SoftBerry (http://linux1.softberry.com/berry.phtml) and ExPASy-ProtParam tools (https://web.expasy.org/). Multiple alignment of amino acid sequence was performed using online software Clustal Omega (https://www.ebi.ac.uk/Tools/msa/clustalo/). Genomic DNA of Cabernet Sauvignon grapes used for the amplification of *VviFLS4* promoter was isolated with a Plant Genomic DNA kit (TIANGEN, Beijing, China). Gene-specific primers (Additional file 15: Table S9) were designed according to the genome sequence of Pinot Noir (PN40024). The amplicons were cloned and sequenced as described above. The promoter sequence was analyzed using the Softberry-TSSP program, PlantCARE (http://bioinformatics.psb.ugent.be/webtools/plantcare/html/), JASPAR (http://jaspar.genereg.net/), and PLACE database (https://www.dna.affrc.go.jp/database/).

### Prokaryotic expression and enzymatic assay

The coding sequence of *VviFLS4* was amplified using the forward primer containing the 5′ *Sma* I site and the reverse primer containing the 5′ *Eco*R I site (Additional file 15: Table S9). It was then cloned into the pET-48b(+) vector (Novagen, Madison, WI, USA) as a translational fusion to the C-terminal 6 × His-tag peptide and transformed into *E. coli* BL21 (DE3) (TIANGEN, Beijing, China) following induction with 1 mM isopropyl-*β*-D-thiogalactopyranoside (IPTG). The recombinant protein was extracted and purified on an Ni^2+^ −chelating chromatography column as described in the pET system manual. The concentration of the purified recombinant His-tag VviFLS4 protein was determined using the Bradford protein assay (Bio-Rad). The enzymatic activity assay was performed according to the method described by Falcone Ferreyra et al. [97] with some modifications. Each 200-μL reaction mixture containing 10 mM *α*-ketoglutaric acid, 0.25 mM FeSO_4_, 10 mM ascorbic acid, 100 mM NaH_2_PO_4_ (pH 6.0), 0.5 μM substrate (Naringenin, DHK, DHQ or DHM), and 50 μg of purified VviFLS4-His protein was incubated at 25 °C for 1 h with gentle shaking. The mixtures were vigorously extracted twice with the same volume of ethyl acetate, dried under nitrogen gas, and then resuspended in 400 μL of 80% methanol. The crude protein extract from the induced BL21 containing the empty pET-48b(+) vector was used as the control. Qualitative and quantitative analyses of the enzymatic products were performed by HPLC-MS under the conditions mentioned above.

### Transient expression in tobacco leaves

Generation of the *VviFLS4* promoter-driven *luciferase* (*LUC*) construct, agrobacterium-mediated transient expression of the promoter::*LUC* construct in tobacco leaves and shading treatment of the infiltrated plants were conducted according to Sun et al. [86]. The primers used for *VviFLS4* promoter::*LUC* construction are listed in Additional file 15: Table S9. Fluorescence signals were recorded with an Andor iXon charge-coupled device (CCD) imaging apparatus (Andor Technology, Belfast, UK). The transcriptional activity of the *LUC* gene was detected by qPCR using previously described conditions [86]. Two housekeeping genes in tobacco, *NbUbi3* and *NbEF-1α*, were selected as endogenous controls [98, 99]. The primers used for qPCR analysis are listed in Additional file 15: Table S9. Three biological replicates and three technical replicates were run per sample. For each group of assays, at least nine leaves from three individual plants were used for CCD imaging and qPCR analysis. Significance of differences in *LUC* gene expression between the control and treatment groups was determined by an independent sample t-test using SPSS 20.0 for windows (SPSS Inc., Chicago, IL, USA).

## Supplementary information

**Additional file 1: Table S1.** Meteorological data of the study area during berry development in 2012 and 2013.

**Additional file 2: Table S2.** Microclimate conditions of the fruiting zone of the bagging-treated and control grapes.

**Additional file 3: Table S3.** Statistical analysis of the physiological and metabolomic data in different bagging-treated berries during each developmental stage in 2012 and 2013.

**Additional file 4: Table S4.** Alignment statistics result with the reference gene for all samples.

**Additional file 5: Figure S1.** Number statistics of DEGs between the cluster bagging-treated and control grapes.

**Additional file 6: Figure S2.** KEGG pathway enrichment analysis of DEGs between the cluster bagging-treated and control grapes.

**Additional file 7: Table S5.** Transcription profile of the phenolic metabolic pathway genes among samples.

**Additional file 8: Table S6.** Correlation analysis between the accumulation of phenolic compounds and the transcription of phenolic biosynthesis-related genes among samples.

**Additional file 9: Figure S3.** Validation of the RNA-Seq results by quantitative real-time PCR. The average FPKM values of the RNA-Seq analysis and the average relative expression values from three independent real-time PCR experiments are shown in the left-hand and right-hand heatmap, respectively. The correlation is significant at the 0.01 level. T1: cluster bagging from 3 WAF until harvest; T2: control group; T8: cluster bagging at E-L 35 stage and bag removal at E-L 36 stage.

**Additional file 10: Figure S4.** Multiple alignment of the deduced amino acid sequences of VviFLS4 from Cabernet Sauvignon, Pinot Noir, and Shiraz grape cultivars.

**Additional file 11: Figure S5.** Purification of the prokaryotic expressed recombinant FLS4-His protein from *Escherichia coli* BL21(DE3) extracts induced with 1 mM isopropyl-β-D-thiogalactopyranoside. kDa, kilo Dalton; M, protein marker; Lanes 1 and 3, total proteins extracted from the empty vector-carrying (lane 1) and VviFLS4-His recombinant (lane 3) strain of *E. coli*; Lanes 2 and 4, the purified empty His-tag (lane 2) and VviFLS4-His recombinant (lane 4) protein fractions used for the enzymatic assay.

**Additional file 12: Table S7.** Transcription profile of light and plant hormone signal transduction related genes among samples.

**Additional file 13: Table S8.** Co-expression analysis of genes encoding transcription factors and key enzymes required for sunlight-regulated phenolic biosynthesis.

**Additional file 14: Figure S6.** Pre-analysis for WGCNA. (a) Clustering dendrogram of samples based on their Euclidean distance. (b) Analysis of network topology for various soft-thresholding powers.

**Additional file 15: Table S9.** List of the primers used in this study.

**Additional file 16: Table S10.** The promoter and cDNA sequences of *VviFLS4* gene.

## Data Availability

The RNA-Seq data are accessible in the NCBI GEO database with the accession number GSE129916 (https://www.ncbi.nlm.nih.gov/geo/). The promoter and cDNA sequences of *VviFLS4* gene are available in Additional file 16: Table S10 and in the NCBI GenBank database with accession numbers MK792349 and MK792350, respectively.

## Abbreviations

ABA: Abscisic acid
BR: Brassinosteroid
CTK: Cytokinin
DEG: Differentially expressed gene
ETH: Ethylene
FLS: Flavonol synthase
GA: Gibberellin
GO: Gene Ontology
HBA: Hydroxybenzoic acid
HCA: Hydroxycinnamic acid
HPLC-MS: High performance liquid chromatography-mass spectrometry
JA: Jasmonic acid
KEGG: Kyoto Encyclopedia of Genes and Genomes
PAs: Proanthocyanidins
PCA: Principal component analysis
PLS-DA: Partial least squares-discriminant analysis
qPCR: Quantitative real-time polymerase chain reaction
RNA-Seq: High-throughput mRNA sequencing
SA: Salicylic acid
TF: Transcription factor
TSS: Total soluble solids
WAF: Weeks after flowering
WGCNA: Weighted gene co-expression network analysis

## References

[1] Rojas-Lara B, Morrison J (1989). Differential effects of shading fruit or foliage on the development and composition of grape berries. Vitis..

[2] Dokoozlian NK, Kliewer WM (1996). Influence of light on grape berry growth and composition varies during fruit development. J Am Soc Hort Sci..

[3] Poni S, Bernizzon F, Civard S, Libelli N (2009). Effects of pre-bloom leaf removal on growth of berry tissues and must composition in two red *Vitis vinifera* L. cultivars. Aust J Grape Wine Res..

[4] Intrieri C, Filippetti I, Allegro G, Centinari M, Poni S (2008). Early defoliation (hand *vs* mechanical) for improved crop control and grape composition in Sangiovese (*Vitis vinifera* L.). Aust J Grape Wine Res..

[5] Pastore C, Zenoni S, Fasoli M, Pezzotti M, Tornielli GB, Filippetti I (2013). Selective defoliation affects plant growth, fruit transcriptional ripening program and flavonoid metabolism in grapevine. BMC Plant Biol..

[6] Spayd SE, Tarara JM, Mee DL, Ferguson JC (2002). Separation of sunlight and temperature effects on the composition of *Vitis vinifera* cv. Merlot Berries. Am J Enol Vitic..

[7] Friedel M, Stoll M, Patz CD, Will F, Dietrich H. Impact of light exposure on fruit composition of white ‘Riesling’ grape berries (*Vitis vinifera* L.). Vitis. 2015;54:107–16.

[8] Kotseridis Y, Georgiadou A, Tikos P, Kallithraka S, Koundouras S (2012). Effects of severity of post-flowering leaf removal on berry growth and composition of three red *Vitis vinifera* L. cultivars grown under semiarid conditions. J Agric Food Chem..

[9] Wu B-H, Cao Y-G, Guan L, Xin H-P, Li J-H, Li S-H (2014). Genome-wide transcriptional profiles of the berry skin of two red grape cultivars (*Vitis vinifera*) in which anthocyanin synthesis is sunlight-dependent or -independent. PLoS One..

[10] Tardaguila J, de Toda FM, Poni S, Diago MP (2010). Impact of early leaf removal on yield and fruit and wine composition of *Vitis vinifera* L. Graciano and Carignan. Am J Enol Vitic..

[11] Sun R, He F, Lan Y, Xing R, Liu R, Pan Q, Wang J, Duan C (2015). Transcriptome comparison of Cabernet Sauvignon grape berries from two regions with distinct climate. J Plant Physiol..

[12] Bergqvist J, Dokoozlian N, Ebisuda N (2001). Sunlight Exposure and Temperature Effects on Berry Growth and Composition of Cabernet Sauvignon and Grenache in the Central San Joaquin Valley of California. Am J Enol Vitic..

[13] Sun R-Z, Cheng G, Li Q, He Y-N, Wang Y, Lan Y-B, Li S-Y, Zhu Y-R, Song W-F, Zhang X, et al. Light-induced variation in phenolic compounds in Cabernet Sauvignon grapes (*Vitis vinifera* L.) involves extensive transcriptome reprogramming of biosynthetic enzymes, transcription factors, and phytohormonal regulators. Front Plant Sci. 2017;8:547. 10.3389/fpls.2017.00547 PMC5395571 28469625

[14] Downey MO, Harvey JS, Robinson SP (2004). The effect of bunch shading on berry development and flavonoid accumulation in Shiraz grapes. Aust J Grape Wine Res..

[15] Li J-H, Guan L, Fan P-G, Li S-H, Wu B-H (2013). Effect of sunlight exclusion at different phenological stages on anthocyanin accumulation in red grape clusters. Am J Enol Vitic..

[16] Adams DO (2006). Phenolics and ripening in grape berries. Am J Enol Vitic..

[17] Teixeira A, Eiras-Dias J, Castellarin S, Gerós H (2013). Berry phenolics of grapevine under challenging environments. Int J Mol Sci..

[18] Downey MO, Dokoozlian NK, Krstic MP (2006). Cultural practice and environmental impacts on the flavonoid composition of grapes and wine: a review of recent research. Am J Enol Vitic..

[19] Waterhouse AL (2002). Wine Phenolics. Ann N Y Acad Sci..

[20] Conde C, Silva P, Fontes N, Dias ACP, Tavares RM, Sousa MJ, Agasse A, Delrot S, Gerós H (2007). Biochemical changes throughout grape berry development and fruit and wine quality. Food..

[21] Sternad Lemut M, Trost K, Sivilotti P, Vrhovsek U (2011). Pinot Noir grape colour related phenolics as affected by leaf removal treatments in the Vipava Valley. J Food Compost Anal..

[22] Šuklje K, Lisjak K, Baša Česnik H, Janeš L, Du Toit W, Coetzee Z, Vanzo A, Deloire A (2012). Classification of grape berries according to diameter and total soluble solids to study the effect of light and temperature on methoxypyrazine, glutathione, and hydroxycinnamate evolution during ripening of Sauvignon blanc (*Vitis vinifera* L.). J Agric Food Chem..

[23] Cortell JM, Kennedy JA (2006). Effect of shading on accumulation of flavonoid compounds in (*Vitis vinifera* L.) Pinot Noir fruit and extraction in a model system. J Agric Food Chem..

[24] Koyama K, Ikeda H, Poudel PR, Goto-Yamamoto N (2012). Light quality affects flavonoid biosynthesis in young berries of Cabernet Sauvignon grape. Phytochemistry..

[25] Reshef N, Agam N, Fait A (2018). Grape berry acclimation to excessive solar irradiance leads to repartitioning between major flavonoid groups. J Agric Food Chem..

[26] Reshef N, Walbaum N, Agam N, Fait A (2017). Sunlight modulates fruit metabolic profile and shapes the spatial pattern of compound accumulation within the grape cluster. Front Plant Sci..

[27] Liu L, Gregan S, Winefield C, Jordan B (2015). From UVR8 to flavonol synthase: UV-B-induced gene expression in Sauvignon blanc grape berry. Plant Cell Environ..

[28] Fujita A, Soma N, Goto-Yamamoto N, Mizuno A, Kiso K, Hashizum K (2007). Effectof shading on proanthocyanidin biosynthesis in the grape berry. J Jpn Soc Hortic Sci..

[29] Ristic R, Downey MO, Iland PG, Bindon K, Francis IL, Herderich M, Robinson SP (2007). Exclusion of sunlight from Shiraz grapes alters wine colour, tannin and sensory properties. Aust J Grape Wine Res..

[30] Koyama K, Goto-Yamamoto N (2008). Bunch shading during different developmental stages affects the phenolic biosynthesis in berry skins of ‘Cabernet Sauvignon’ grapes. J Am Soc Hort Sci..

[31] Ban T, Shiozaki S, Ogata T, Horiuchi S (2000). Effects of abscisic acid and shading treatments on the levels of anthocyanin and resveratrol in skin of Kyoho grape berry. Acta Hortic..

[32] Jeong ST, Goto-Yamamoto N, Kobayashi S, Esaka M (2004). Effects of plant hormones and shading on the accumulation of anthocyanins and the expression of anthocyanin biosynthetic genes in grape berry skins. Plant Sci..

[33] Chorti E, Guidoni S, Ferrandino A, Novello V (2010). Effect of different cluster sunlight exposure levels on ripening and anthocyanin accumulation in Nebbiolo grapes. Am J Enol Vitic..

[34] Guan L, Dai Z, Wu B-H, Wu J, Merlin I, Hilbert G, Renaud C, Gomès E, Edwards E, Li S-H (2016). Anthocyanin biosynthesis is differentially regulated by light in the skin and flesh of white-fleshed and teinturier grape berries. Planta..

[35] Matus JT, Loyola R, Vega A, Peña-Neira A, Bordeu E, Arce-Johnson P, Alcalde JA (2009). Post-veraison sunlight exposure induces MYB-mediated transcriptional regulation of anthocyanin and flavonol synthesis in berry skins of *Vitis vinifera*. J Exp Bot..

[36] Matsuyama S, Tanzawa F, Kobayashi H, Suzuki S, Takata R, Saito H (2014). Leaf removal accelerated accumulation of delphinidin-based anthocyanins in ‘Muscat Bailey A’ [*Vitis × labruscana* (Bailey) and *Vitis vinifera* (Muscat Hamburg)] grape skin. J Jpn Soc Hortic Sci..

[37] Wang Y, He Y-N, Chen W-K, He F, Chen W, Cai X-D, Duan C-Q, Wang J (2018). Effects of cluster thinning on vine photosynthesis, berry ripeness and flavonoid composition of Cabernet Sauvignon. Food Chem..

[38] Rustioni L, Rossoni M, Cola G, Mariani L, Failla O (2011). Bunch exposure to direct solar radiation increases ortho-diphenol anthocyanins in Northern Italy climatic condition. J Int Sci Vine Vin..

[39] Lee J, Skinkis PA (2013). Oregon ‘Pinot noir’ grape anthocyanin enhancement by early leaf removal. Food Chem..

[40] Haselgrove L, Botting D, van Heeswijck R, Høj PB, Dry PR, Ford C, Land PGI (2000). Canopy microclimate and berry composition: the effect of bunch exposure on the phenolic composition of *Vitis vinifera* L cv. Shiraz grape berries. Aust J Grape Wine Res..

[41] Tarara JM, Lee J, Spayd SE, Scagel CF (2008). Berry temperature and solar radiation alter acylation, proportion, and concentration of anthocyanin in Merlot grapes. Am J Enol Vitic..

[42] Hichri I, Barrieu F, Bogs J, Kappel C, Delrot S, Lauvergeat V (2011). Recent advances in the transcriptional regulation of the flavonoid biosynthetic pathway. J Exp Bot..

[43] Petrussa E, Braidot E, Zancani M, Peresson C, Bertolini A, Patui S, Vianello A (2013). Plant flavonoids—biosynthesis, transport and involvement in stress responses. Int J Mol Sci..

[44] Zoratti L, Karppinen K, Luengo Escobar A, Häggman H, Jaakola L (2014). Light-controlled flavonoid biosynthesis in fruits. Front Plant Sci..

[45] Czemmel S, Stracke R, Weisshaar B, Cordon N, Harris NN, Walker AR, Robinson SP, Bogs J (2009). The grapevine R2R3-MYB transcription factor VvMYBF1 regulates flavonol synthesis in developing grape berries. Plant Physiol..

[46] Azuma A, Yakushiji H, Koshita Y, Kobayashi S (2012). Flavonoid biosynthesis-related genes in grape skin are differentially regulated by temperature and light conditions. Planta..

[47] Matus JT, Poupin MJ, Cañón P, Bordeu E, Alcalde JA, Arce-Johnson P (2010). Isolation of WDR and bHLH genes related to flavonoid synthesis in grapevine (*Vitis vinifera* L.). Plant Mol Biol..

[48] Loyola R, Herrera D, Mas A, Wong DCJ, Höll J, Cavallini E, Amato A, Azuma A, Ziegler T, Aquea F (2016). The photomorphogenic factors UV-B RECEPTOR 1, ELONGATED HYPOCOTYL 5, and HY5 HOMOLOGUE are part of the UV-B signalling pathway in grapevine and mediate flavonol accumulation in response to the environment. J Exp Bot..

[49] Malacarne G, Coller E, Czemmel S, Vrhovsek U, Engelen K, Goremykin V, Bogs J, Moser C (2016). The grapevine VvibZIPC22 transcription factor is involved in the regulation of flavonoid biosynthesis. J Exp Bot..

[50] Matus JT (2016). Transcriptomic and metabolomic networks in the grape berry illustrate that it takes more than flavonoids to fight against ultraviolet radiation. Front Plant Sci..

[51] Carbonell-Bejerano P, Diago M-P, Martínez-Abaigar J, Martínez-Zapater JM, Tardáguila J, Núñez-Olivera E (2014). Solar ultraviolet radiation is necessary to enhance grapevine fruit ripening transcriptional and phenolic responses. BMC Plant Biol..

[52] Suzuki M, Nakabayashi R, Ogata Y, Sakurai N, Tokimatsu T, Goto S, Suzuki M, Jasinski M, Martinoia E, Otagaki S (2015). Multiomics in grape berry skin revealed specific induction of the stilbene synthetic pathway by ultraviolet-C irradiation. Plant Physiol..

[53] Zenoni S, Dal Santo S, Tornielli GB, D'Incà E, Filippetti I, Pastore C, Allegro G, Silvestroni O, Lanari V, Pisciotta A (2017). Transcriptional responses to pre-flowering leaf defoliation in grapevine berry from different growing sites, years, and genotypes. Front Plant Sci..

[54] Downey MO, Harvey JS, Robinson SP (2003). Synthesis of flavonols and expression of flavonol synthase genes in the developing grape berries of Shiraz and Chardonnay (*Vitis vinifera* L.). Aust J Grape Wine Res..

[55] Reid KE, Olsson N, Schlosser J, Peng F, Lund ST (2006). An optimized grapevine RNA isolation procedure and statistical determination of reference genes for real-time RT-PCR during berry development. BMC Plant Biol..

[56] Mu L, He F, Pan Q-H, Zhou L, Duan C-Q (2014). Screening and verification of late embryogenesis abundant protein interacting with anthocyanidin reductase in grape berries. Vitis..

[57] Griñán I, Morales D, Galindo A, Torrecillas A, Pérez-López D, Moriana A, Collado-González J, Carbonell-Barrachina ÁA, Hernández F (2019). Effect of preharvest fruit bagging on fruit quality characteristics and incidence of fruit physiopathies in fully irrigated and water stressed pomegranate trees. J Sci Food Agric..

[58] Karajeh MR (2018). Pre-harvest bagging of grape clusters as a non-chemical physical control measure against certain pests and diseases of grapevines. Org Agric..

[59] Sharma R, Pal R, Asrey R, Sagar V, Dhiman M, Rana M (2013). Pre-harvest fruit bagging influences fruit color and quality of apple cv. Delicious. Agric Sci..

[60] Sharma RR, Reddy SVR, Jhalegar MJ (2014). Pre-harvest fruit bagging: a useful approach for plant protection and improved post-harvest fruit quality – a review. J Hortic Sci Biotechnol..

[61] Hudina M, Stampar F (2011). Effect of fruit bagging on quality of ‘Conference’ pear (*Pyrus communis* L.). Eur J Hortic Sci..

[62] Zhou Y, Yuan C, Ruan S, Zhang Z, Meng J, Xi Z (2018). Exogenous 24-epibrassinolide interacts with light to regulate anthocyanin and proanthocyanidin biosynthesis in Cabernet Sauvignon (*Vitis vinifera* L.). Molecules.

[63] Cheynier V, Comte G, Davies KM, Lattanzio V, Martens S (2013). Plant phenolics: Recent advances on their biosynthesis, genetics, and ecophysiology. Plant Physiol Biochem..

[64] Li Q, He F, Zhu B-Q, Liu B, Sun R-Z, Duan C-Q, Reeves MJ, Wang J (2014). Comparison of distinct transcriptional expression patterns of flavonoid biosynthesis in Cabernet Sauvignon grapes from east and west China. Plant Physiol Biochem..

[65] Jaakola L (2013). New insights into the regulation of anthocyanin biosynthesis in fruits. Trends Plant Sci..

[66] Liu C-C, Chi C, Jin L-J, Zhu J, Yu J-Q, Zhou Y-H (2018). The bZip transcription factor HY5 mediates CRY1a-induced anthocyanin biosynthesis in tomato. Plant Cell Environ..

[67] Tao R, Bai S, Ni J, Yang Q, Zhao Y, Teng Y (2018). The blue light signal transduction pathway is involved in anthocyanin accumulation in ‘Red Zaosu’ pear. Planta..

[68] Matus JT, Cavallini E, Loyola R, Höll J, Finezzo L, Dal Santo S, Vialet S, Commisso M, Roman F, Schubert A (2017). A group of grapevine MYBA transcription factors located in chromosome 14 control anthocyanin synthesis in vegetative organs with different specificities compared with the berry color locus. Plant J..

[69] Boss PK, Davies C, Robinson SP (1996). Analysis of the expression of anthocyanin pathway genes in developing *Vitis vinifera* L. cv Shiraz grape berries and the implications for pathway regulation. Plant Physiol..

[70] Kobayashi S, Ishimaru M, Hiraoka K, Honda C (2002). Myb-related genes of the Kyoho grape (*Vitis labruscana*) regulate anthocyanin biosynthesis. Planta..

[71] Falcone Ferreyra ML, Rius SP, Casati P (2012). Flavonoids: biosynthesis, biological functions, and biotechnological applications. Front Plant Sci..

[72] Fujita A, Goto-Yamamoto N, Aramaki I, Hashizume K (2006). Organ-specific transcription of putative flavonol synthase genes of grapevine and effects of plant hormones and shading on flavonol biosynthesis in grape berry skins. Biosci Biotechnol Biochem..

[73] Czemmel S, Höll J, Loyola R, Arce-Johnson P, Alcalde JA, Matus JT, Bogs J (2017). Transcriptome-wide identification of novel UV-B- and light modulated flavonol pathway genes controlled by VviMYBF1. Front Plant Sci..

[74] Lau OS, Deng XW (2010). Plant hormone signaling lightens up: integrators of light and hormones. Curr Opin Plant Biol..

[75] Seo M, Nambara E, Choi G, Yamaguchi S (2009). Interaction of light and hormone signals in germinating seeds. Plant Mol Biol..

[76] Li Q-F, He J-X (2016). BZR1 interacts with HY5 to mediate brassinosteroid- and light-regulated cotyledon opening in *Arabidopsis* in darkness. Mol Plant..

[77] He F, Mu L, Yan G-L, Liang N-N, Pan Q-H, Wang J, Reeves MJ, Duan C-Q (2000). Biosynthesis of anthocyanins and their regulation in colored grapes. Molecules.

[78] Berli FJ, Fanzone M, Piccoli P, Bottini R (2011). Solar UV-B and ABA are involved in phenol metabolism of *Vitis vinifera* L. increasing biosynthesis of berry skin polyphenols. J Agric Food Chem..

[79] Luan L-Y, Zhang Z-W, Xi Z-M, Huo S-S, Ma L-N (2013). Brassinosteroids regulate anthocyanin biosynthesis in the ripening of grape berries. S Afr J Enol Vitic..

[80] Ban T, Ishimaru M, Kobayashi S, Goto-Yamamoto N, Horiuchi S (2003). Abscisic acid and 2,4-dichlorophenoxyacetic acid affect the expression of anthocyanin biosynthetic pathway genes in ‘Kyoho’ grape berries. J Hortic Sci Biotechnol..

[81] Fasoli M, Richter CL, Zenoni S, Bertini E, Vitulo N, Santo SD, Dokoozlian N, Pezzotti M, Tornielli GB (2018). Timing and order of the molecular events marking the onset of berry ripening in grapevine. Plant Physiol..

[82] Li S (2014). Transcriptional control of flavonoid biosynthesis: fine-tuning of the MYB-bHLH-WD40 (MBW) complex. Plant Signal Behav..

[83] Xu W, Dubos C, Lepiniec L (2015). Transcriptional control of flavonoid biosynthesis by MYB–bHLH–WDR complexes. Trends Plant Sci..

[84] Nesi N, Debeaujon I, Jond C, Stewart AJ, Jenkins GI, Caboche M, Lepiniec L (2002). The *TRANSPARENT TESTA16* locus encodes the ARABIDOPSIS BSISTER MADS domain protein and is required for proper development and pigmentation of the seed coat. Plant Cell..

[85] Morishita T, Kojima Y, Maruta T, Nishizawa-Yokoi A, Yabuta Y, Shigeoka S (2009). Arabidopsis NAC transcription factor, ANAC078, regulates flavonoid biosynthesis under high-light. Plant Cell Physiol..

[86] Sun R-Z, Pan Q-H, Wang J, Duan C-Q (2015). Light response and potential interacting proteins of a grape *flavonoid 3′-hydroxylase* gene promoter. Plant Physiol Biochem..

[87] Zhou H, Lin-Wang K, Wang H, Gu C, Dare AP, Espley RV, He H, Allan AC, Han Y (2015). Molecular genetics of blood-fleshed peach reveals activation of anthocyanin biosynthesis by NAC transcription factors. Plant J..

[88] Amato A, Cavallini E, Zenoni S, Finezzo L, Begheldo M, Ruperti B, Tornielli GB (2017). A grapevine TTG2-like WRKY transcription factor is involved in regulating vacuolar transport and flavonoid biosynthesis. Front Plant Sci..

[89] Hilker M, Schmülling T (2019). Stress priming, memory, and signalling in plants. Plant Cell Environ..

[90] Kumar S (2018). Epigenomics of plant responses to environmental stress. Epigenomes..

[91] Sun R-Z, Zuo E-H, Qi J-F, Liu Y, Lin C-T, Deng X. A role of age-dependent DNA methylation reprogramming in regulating the regeneration capacity of *Boea hygrometrica* leaves. Funct Integr Genomic. 2019. 10.1007/s10142-019-00701-3. 10.1007/s10142-019-00701-3 31414312

[92] Cheng G, He Y-N, Yue T-X, Wang J, Zhang Z-W (2014). Effects of climatic conditions and soil properties on Cabernet Sauvignon berry growth and anthocyanin profiles. Molecules..

[93] Zhang Z-Z, Li X-X, Chu Y-N, Zhang M-X, Wen Y-Q, Duan C-Q, Pan Q-H (2012). Three types of ultraviolet irradiation differentially promote expression of shikimate pathway genes and production of anthocyanins in grape berries. Plant Physiol Biochem..

[94] Langmead B, Trapnell C, Pop M, Salzberg SL (2009). Ultrafast and memory-efficient alignment of short DNA sequences to the human genome. Genome Biol..

[95] Tarazona S, Garcia-Alcalde F, Dopazo J, Ferrer A, Conesa A (2011). Differential expression in RNA-seq: a matter of depth. Genome Res..

[96] Langfelder P, Horvath S (2008). WGCNA: an R package for weighted correlation network analysis. BMC Bioinformatics..

[97] Falcone Ferreyra ML, Rius S, Emiliani J, Pourcel L, Feller A, Morohashi K, Casati P, Grotewold E (2010). Cloning and characterization of a UV-B-inducible maize flavonol synthase. Plant J..

[98] Rotenberg D, Thompson TS, German TL, Willis DK (2006). Methods for effective real-time RT-PCR analysis of virus-induced gene silencing. J Virol Methods..

[99] Ishihama N, Yamada R, Yoshioka M, Katou S, Yoshioka H (2011). Phosphorylation of the *Nicotiana benthamiana* WRKY8 transcription factor by MAPK functions in the defense response. Plant Cell..

