# Supplementary material for: Comparative physiological, metabolomic, and transcriptomic analyses reveal developmental stage-dependent effects of cluster bagging on phenolic metabolism in Cabernet Sauvignon grape berries
Source: BMC Plant Biol. 2019 Dec 26;19:583. doi: 10.1186/s12870-019-2186-z (PMC6933938; doi:10.1186/s12870-019-2186-z)
Supplement: Supplementary file 1 — Additional file 1: Table S1. Meteorological data of the study area during berry development in 2012 and 2013. [file 12870_2019_2186_MOESM1_ESM.docx]

**Table S1.** Meteorological data of the study area during berry development in 2012 and 2013.

| Developmental  stage | Number of days | | Growing degree days (°C) | | Sunshine duration (h) | | Temperature (°C) | | | | | | Rainfall (mm) | | Relative humidity (%) | |
| --- | --- | --- | --- | --- | --- | --- | --- | --- | --- | --- | --- | --- | --- | --- | --- | --- |
|  |  |  |  |  |  |  | Average | | Maximum | | Minimum | |  |  |  |  |
|  | 2012 | 2013 | 2012 | 2013 | 2012 | 2013 | 2012 | 2013 | 2012 | 2013 | 2012 | 2013 | 2012 | 2013 | 2012 | 2013 |
| flowering | 7 | 7 | 113.5 | 96.7 | 70.8 | 82.4 | 26.2 | 23.8 | 31.2 | 30.9 | 20.9 | 18.5 | 0.1 | 1.1 | 30.7 | 32.1 |
| green | 47 | 42 | 785.6 | 624.1 | 497.7 | 451.7 | 26.7 | 24.9 | 32.6 | 30.8 | 21.4 | 19.5 | 35.9 | 38.7 | 40.7 | 34.6 |
| véraison | 21 | 23 | 347.4 | 373.2 | 240.0 | 237.4 | 26.5 | 26.2 | 32.8 | 32.6 | 20.3 | 19.5 | 10.2 | 11.3 | 41.6 | 38.6 |
| ripening | 39 | 41 | 480.8 | 415.8 | 399.8 | 415.7 | 22.3 | 20.1 | 28.8 | 27.8 | 15.7 | 13.0 | 20.1 | 21.9 | 42.4 | 40.9 |
