# Supplementary material for: Comparative physiological, metabolomic, and transcriptomic analyses reveal developmental stage-dependent effects of cluster bagging on phenolic metabolism in Cabernet Sauvignon grape berries
Source: BMC Plant Biol. 2019 Dec 26;19:583. doi: 10.1186/s12870-019-2186-z (PMC6933938; doi:10.1186/s12870-019-2186-z)
Supplement: Supplementary file 2 — Additional file 2: Table S2. Microclimate conditions of the fruiting zone of the bagging-treated and control grapes. [file 12870_2019_2186_MOESM2_ESM.docx]

**Table S2.** Microclimate conditions of the fruiting zone of the bagging treated and control grapes.

| Developmental stage | PAR (µmol·m^-2^·s^-1^) | | | | SR (µmol·m^-2^·s^-1^) | | | | Average temperature (°C) | | | | Relative humidity (%) | | | |
| --- | --- | --- | --- | --- | --- | --- | --- | --- | --- | --- | --- | --- | --- | --- | --- | --- |
|  | 2012 | | 2013 | | 2012 | | 2013 | | 2012 | | 2013 | | 2012 | | 2013 | |
|  | CK | CB | CK | CB | CK | CB | CK | CB | CK | CB | CK | CB | CK | CB | CK | CB |
| Stage I | 14.24 | 1.20 | 33.14 | 1.20 | 35.46 | 0.60 | 30.90 | 0.60 | 24.89 | 25.89 | 24.33 | 25.41 | 60.33 | 58.21 | 59.49 | 54.77 |
| Stage II | 16.55 | 1.20 | 31.45 | 1.20 | 52.08 | 0.60 | 35.67 | 0.60 | 26.05 | 27.60 | 24.89 | 25.86 | 56.68 | 52.54 | 68.84 | 66.18 |
| Stage III | 21.49 | 1.20 | 8.71 | 1.20 | 55.83 | 0.60 | 33.80 | 0.60 | 24.57 | 26.16 | 23.00 | 23.59 | 55.66 | 51.97 | 69.00 | 66.24 |
| Stage IV | 15.51 | 1.20 | 9.84 | 1.20 | 46.32 | 0.60 | 35.26 | 0.60 | 22.92 | 24.36 | 21.00 | 21.82 | 50.83 | 47.86 | 66.20 | 63.30 |
| Stage V | 7.70 | 1.20 | 11.49 | 1.20 | 29.59 | 0.60 | 26.80 | 0.60 | 19.66 | 20.69 | 17.63 | 18.16 | 61.31 | 58.35 | 61.45 | 58.98 |

Stage I, from fruit-set to pre-véraison; stage II, from the beginning of véraison to middle véraison; stage III, from middle véraison to the end of véraison; stage IV, from the end of véraison to pre-harvest; stage V, from pre-harvest to harvest. PAR, photosynthetically active radiation; SR, solar radiation. CK, control group; CB, cluster bagging treated group.
