# Supplementary material for: Comparative physiological, metabolomic, and transcriptomic analyses reveal developmental stage-dependent effects of cluster bagging on phenolic metabolism in Cabernet Sauvignon grape berries
Source: BMC Plant Biol. 2019 Dec 26;19:583. doi: 10.1186/s12870-019-2186-z (PMC6933938; doi:10.1186/s12870-019-2186-z)
Supplement: Supplementary file 3 — Additional file 3: Table S3. Statistical analysis of the physiological and metabolomic data in different bagging-treated berries during each developmental stage in 2012 and 2013. [file 12870_2019_2186_MOESM3_ESM.docx]

**Table S3.** Statistical analysis of the physiological and metabolomic data in different bagging-treated berries during each developmental stage in 2012 and 2013.

| Data | Year | Developmental stage | Treatment | | | | | | | | | | |
| --- | --- | --- | --- | --- | --- | --- | --- | --- | --- | --- | --- | --- | --- |
|  |  |  | T1 | T2 | T3 | T4 | T5 | T6 | T7 | T8 | T9 | T10 | T11 |
| Berry fresh weight | 2012 | E-L 29 | a | a | a | a | a | a | a | a | a | a | a |
|  |  | E-L 31 | a | a | a | a | a | a | a | a | a | a | a |
|  |  | E-L 33 | b | a | b | b | b | a | a | a | a | a | a |
|  |  | E-L 35 | c | a | c | c | b | a | a | a | a | a | a |
|  |  | E-L 36 | b | a | ab | b | b | ab | b | b | a | a | a |
|  |  | E-L 37 | bc | d | d | c | d | c | d | ab | d | a | d |
|  |  | E-L 38 | bc | c | bc | bc | a | a | d | c | ab | ab | ab |
|  | 2013 | E-L 29 | a | a | a | a | a | a | a | a | a | a | a |
|  |  | E-L 31 | a | a | a | a | a | a | a | a | a | a | a |
|  |  | E-L 33 | a | a | a | a | a | a | a | a | a | a | a |
|  |  | E-L 35 | a | a | a | a | a | a | a | a | a | a | a |
|  |  | E-L 36 | b | b | b | a | b | b | b | b | b | b | b |
|  |  | E-L 37 | ab | ab | b | b | a | ab | ab | b | b | ab | ab |
|  |  | E-L 38 | a | a | a | a | a | a | a | a | a | a | a |
| Total soluble solids | 2012 | E-L 29 | a | a | a | a | a | a | a | a | a | a | a |
|  |  | E-L 31 | a | a | a | a | a | a | a | a | a | a | a |
|  |  | E-L 33 | a | a | a | a | a | a | a | a | a | a | a |
|  |  | E-L 35 | b | a | b | b | a | a | a | a | a | a | a |
|  |  | E-L 36 | e | d | cd | b | e | a | bc | f | d | d | d |
|  |  | E-L 37 | cd | a | a | b | a | d | d | e | bc | cd | a |
|  |  | E-L 38 | a | a | a | a | a | a | a | a | a | a | a |
|  | 2013 | E-L 29 | a | a | a | a | a | a | a | a | a | a | a |
|  |  | E-L 31 | a | a | a | a | a | a | a | a | a | a | a |
|  |  | E-L 33 | a | a | a | a | a | a | a | a | a | a | a |
|  |  | E-L 35 | a | a | a | a | a | a | a | a | a | a | a |
|  |  | E-L 36 | a | a | a | a | a | a | a | a | a | a | a |
|  |  | E-L 37 | a | a | a | a | a | a | a | a | a | a | a |
|  |  | E-L 38 | a | a | a | a | a | a | a | a | a | a | a |
| Titratable acidity | 2012 | E-L 29 | a | a | a | a | a | a | a | a | a | a | a |
|  |  | E-L 31 | b | a | b | b | b | a | a | a | a | a | a |
|  |  | E-L 33 | b | a | b | b | b | a | a | a | a | a | a |
|  |  | E-L 35 | a | c | a | a | b | c | c | c | c | c | c |
|  |  | E-L 36 | de | e | bc | bc | a | ab | cd | ab | e | e | ea |
|  |  | E-L 37 | cde | de | e | de | bcd | a | ab | ab | bcd | abc | de |
|  |  | E-L 38 | a | ab | b | a | ab | ab | ab | a | a | ab | ab |
|  | 2013 | E-L 29 | a | a | a | a | a | a | a | a | a | a | a |
|  |  | E-L 31 | a | a | a | a | a | a | a | a | a | a | a |
|  |  | E-L 33 | b | a | b | b | b | a | a | a | a | a | a |
|  |  | E-L 35 | a | a | a | a | a | a | a | a | a | a | a |
|  |  | E-L 36 | a | a | a | a | a | a | a | a | a | a | a |
|  |  | E-L 37 | a | a | a | a | a | a | a | a | a | a | a |
|  |  | E-L 38 | a | a | a | a | a | a | a | a | a | a | a |
| Hydroxybenzoic acids | 2012 | E-L 29 | a | a | a | a | a | a | a | a | a | a | a |
|  |  | E-L 31 | b | a | b | b | b | a | a | a | a | a | a |
|  |  | E-L 33 | a | a | a | a | a | a | a | a | a | a | a |
|  |  | E-L 35 | a | a | a | a | a | a | a | a | a | a | a |
|  |  | E-L 36 | c | a | c | c | c | ab | a | bc | a | a | a |
|  |  | E-L 37 | b | a | b | a | a | c | a | b | a | a | a |
|  |  | E-L 38 | ab | ab | d | a | e | d | f | b | f | c | c |
|  | 2013 | E-L 29 | a | a | a | a | a | a | a | a | a | a | a |
|  |  | E-L 31 | a | b | a | a | a | b | b | b | b | b | b |
|  |  | E-L 33 | a | b | a | a | a | b | b | b | b | b | b |
|  |  | E-L 35 | a | c | a | a | b | c | c | c | c | c | c |
|  |  | E-L 36 | f | b | f | a | e | c | b | d | b | b | b |
|  |  | E-L 37 | b | d | b | e | f | b | d | a | c | d | d |
|  |  | E-L 38 | a | f | bcde | bcde | abcd | bcde | ef | abc | ab | cde | abcd |
| Hydroxycinnamic acids | 2012 | E-L 29 | a | a | a | a | a | a | a | a | a | a | a |
|  |  | E-L 31 | a | a | a | a | a | a | a | a | a | a | a |
|  |  | E-L 33 | b | a | b | b | b | a | a | a | a | a | a |
|  |  | E-L 35 | a | a | a | a | a | a | a | a | a | a | a |
|  |  | E-L 36 | ab | a | ab | b | a | ab | a | a | a | a | a |
|  |  | E-L 37 | c | a | c | c | d | d | a | b | c | a | a |
|  |  | E-L 38 | b | b | a | bc | cd | d | b | ab | d | cd | cd |
|  | 2013 | E-L 29 | a | a | a | a | a | a | a | a | a | a | a |
|  |  | E-L 31 | a | b | a | a | a | b | b | b | b | b | b |
|  |  | E-L 33 | a | b | a | a | a | b | b | b | b | b | b |
|  |  | E-L 35 | b | c | b | b | a | c | c | c | c | c | c |
|  |  | E-L 36 | c | e | c | d | a | b | e | c | e | e | e |
|  |  | E-L 37 | d | b | d | a | b | d | b | c | e | b | b |
|  |  | E-L 38 | d | a | b | c | f | b | g | f | c | d | e |
| Flavonoids | 2012 | E-L 29 | a | a | a | a | a | a | a | a | a | a | a |
|  |  | E-L 31 | a | b | a | a | a | b | b | b | b | b | b |
|  |  | E-L 33 | a | a | a | a | a | a | a | a | a | a | a |
|  |  | E-L 35 | c | b | c | c | a | b | b | b | b | b | b |
|  |  | E-L 36 | cd | a | cd | b | a | d | a | bc | a | a | a |
|  |  | E-L 37 | b | a | b | a | a | c | a | b | d | a | a |
|  |  | E-L 38 | d | c | a | b | b | g | e | c | f | e | e |
|  | 2013 | E-L 29 | a | a | a | a | a | a | a | a | a | a | a |
|  |  | E-L 31 | a | a | a | a | a | a | a | a | a | a | a |
|  |  | E-L 33 | a | a | a | a | a | a | a | a | a | a | a |
|  |  | E-L 35 | b | b | b | b | a | b | b | b | b | b | b |
|  |  | E-L 36 | c | bc | c | ab | a | ab | bc | ab | bc | bc | bc |
|  |  | E-L 37 | c | b | c | a | a | b | c | b | c | b | b |
|  |  | E-L 38 | de | de | ab | a | a | de | de | bc | e | de | cd |
| 3’-OH/3’,5’-OH | 2012 | E-L 29 | a | a | a | a | a | a | a | a | a | a | a |
|  |  | E-L 31 | a | a | a | a | a | a | a | a | a | a | a |
|  |  | E-L 33 | a | a | a | a | a | a | a | a | a | a | a |
|  |  | E-L 35 | c | b | c | c | a | b | b | b | b | b | b |
|  |  | E-L 36 | b | c | b | a | b | ab | ab | bc | c | c | c |
|  |  | E-L 37 | a | b | a | a | b | a | a | a | b | b | b |
|  |  | E-L 38 | a | ab | ab | ab | b | ab | c | ab | ab | bc | bc |
|  | 2013 | E-L 29 | a | a | a | a | a | a | a | a | a | a | a |
|  |  | E-L 31 | b | a | b | b | b | a | a | a | a | a | a |
|  |  | E-L 33 | a | a | a | a | a | a | a | a | a | a | a |
|  |  | E-L 35 | a | a | a | a | a | a | a | a | a | a | a |
|  |  | E-L 36 | ab | ab | ab | bc | a | d | d | a | ab | ab | ab |
|  |  | E-L 37 | a | bc | a | c | a | bc | bc | ab | d | bc | bc |
|  |  | E-L 38 | bc | c | a | a | ab | ab | a | ab | ab | ab | ab |
| Flavan-3-ols | 2012 | E-L 29 | a | a | a | a | a | a | a | a | a | a | a |
|  |  | E-L 31 | a | a | a | a | a | a | a | a | a | a | a |
|  |  | E-L 33 | a | a | a | a | a | a | a | a | a | a | a |
|  |  | E-L 35 | b | b | b | b | a | b | b | b | b | b | b |
|  |  | E-L 36 | ab | ab | ab | a | a | b | ab | ab | ab | ab | ab |
|  |  | E-L 37 | a | a | a | a | a | a | a | b | c | a | a |
|  |  | E-L 38 | c | d | a | b | b | ef | f | cd | ef | ef | e |
|  | 2013 | E-L 29 | a | a | a | a | a | a | a | a | a | a | a |
|  |  | E-L 31 | a | a | a | a | a | a | a | a | a | a | a |
|  |  | E-L 33 | a | b | b | b | b | b | b | b | b | b | b |
|  |  | E-L 35 | b | b | b | b | a | b | b | b | b | b | b |
|  |  | E-L 36 | c | bc | bc | a | ab | ab | bc | a | bc | bc | bc |
|  |  | E-L 37 | a | a | a | a | a | a | a | a | a | a | a |
|  |  | E-L 38 | abc | c | a | abc | abc | ab | bc | a | abc | c | abc |
| Anthocyanins | 2012 | E-L 29 | a | a | a | a | a | a | a | a | a | a | a |
|  |  | E-L 31 | a | a | a | a | a | a | a | a | a | a | a |
|  |  | E-L 33 | a | a | a | a | a | a | a | a | a | a | a |
|  |  | E-L 35 | c | a | c | c | b | a | a | a | a | a | a |
|  |  | E-L 36 | d | a | d | c | b | cd | a | d | a | a | a |
|  |  | E-L 37 | d | ab | d | bc | a | e | ab | c | d | ab | ab |
|  |  | E-L 38 | e | b | ab | ab | a | g | d | c | f | d | e |
|  | 2013 | E-L 29 | a | a | a | a | a | a | a | a | a | a | a |
|  |  | E-L 31 | a | a | a | a | a | a | a | a | a | a | a |
|  |  | E-L 33 | a | a | a | a | a | a | a | a | a | a | a |
|  |  | E-L 35 | b | a | b | b | b | a | a | a | a | a | a |
|  |  | E-L 36 | bc | a | bc | b | a | b | a | c | a | a | a |
|  |  | E-L 37 | d | b | d | b | a | d | b | c | d | b | b |
|  |  | E-L 38 | cd | c | b | b | a | e | de | cd | e | cde | de |
| Flavonols | 2012 | E-L 29 | a | a | a | a | a | a | a | a | a | a | a |
|  |  | E-L 31 | a | a | a | a | a | a | a | a | a | a | a |
|  |  | E-L 33 | a | a | a | a | a | a | a | a | a | a | a |
|  |  | E-L 35 | b | b | b | b | a | b | b | b | b | b | b |
|  |  | E-L 36 | c | b | c | c | b | c | b | a | b | b | b |
|  |  | E-L 37 | d | b | d | a | ab | d | b | b | c | b | b |
|  |  | E-L 38 | e | c | a | b | b | e | b | c | d | c | c |
|  | 2013 | E-L 29 | a | a | a | a | a | a | a | a | a | a | a |
|  |  | E-L 31 | a | b | a | a | a | b | b | b | b | b | b |
|  |  | E-L 33 | a | a | a | a | a | a | a | a | a | a | a |
|  |  | E-L 35 | b | ab | b | b | a | ab | ab | ab | ab | ab | ab |
|  |  | E-L 36 | b | b | b | b | a | b | b | b | b | b | b |
|  |  | E-L 37 | b | a | b | a | a | b | a | a | b | a | a |
|  |  | E-L 38 | f | ef | d | b | a | f | def | de | def | c | bc |

Different letters represent significant difference at the 0.01 level among treatments in each sampling time point. T1, cluster bagging from 3 WAF until harvest; T2, control group; T3, cluster bagging at E-L 29 stage and bag removal at E-L 37 stage; T4, cluster bagging at E-L 29 stage and bag removal at E-L 36 stage; T5, cluster bagging at E-L 29 stage and bag removal at E-L 35 stage; T6, cluster bagging from E-L 35 to E-L 38 stages; T7, cluster bagging at E-L 35 stage and bag removal at E-L 37 stage; T8, cluster bagging at E-L 35 stage and bag removal at E-L 36 stage; T9, cluster bagging from E-L 37 to E-L 38 stages; T10, cluster bagging at E-L 37 stage and bag removal at E-L 37.5 stage; T11, cluster bagging from E-L 37.5 to E-L 38 stages.
