# Supplementary material for: Comparative physiological, metabolomic, and transcriptomic analyses reveal developmental stage-dependent effects of cluster bagging on phenolic metabolism in Cabernet Sauvignon grape berries
Source: BMC Plant Biol. 2019 Dec 26;19:583. doi: 10.1186/s12870-019-2186-z (PMC6933938; doi:10.1186/s12870-019-2186-z)
Supplement: Supplementary file 4 — Additional file 4: Table S4. Alignment statistics result with the reference gene for all samples. [file 12870_2019_2186_MOESM4_ESM.docx]

**Table S4.** Alignment statistics result with the reference gene for all samples.

| Sample | Total clean reads | Total base-pair (Mb) | Gene map ratio | Unique matching ratio | Expressed genes | Expressed transcripts |
| --- | --- | --- | --- | --- | --- | --- |
| T1_EL-31_A | 12,431,594 | 580.93 | 84.98% | 74.80% | 22,982 | 32,557 |
| T1_EL-31_B | 12,370,818 | 578.09 | 82.95% | 72.89% | 22,769 | 32,499 |
| T1_EL-31_C | 12,225,920 | 571.32 | 87.04% | 76.80% | 23,122 | 32,641 |
| T1_EL-35_A | 12,355,183 | 577.36 | 87.87% | 78.27% | 21,304 | 29,941 |
| T1_EL-35_B | 11,941,481 | 558.03 | 86.30% | 76.69% | 21,262 | 29,504 |
| T1_EL-35_C | 12,370,254 | 578.06 | 86.41% | 77.18% | 21,508 | 30,280 |
| T1_EL-36_A | 11,875,447 | 554.94 | 85.94% | 77.15% | 21,588 | 31,083 |
| T1_EL-36_B | 12,104,184 | 565.63 | 87.20% | 78.34% | 21,194 | 30,232 |
| T1_EL-36_C | 12,625,058 | 589.97 | 86.16% | 77.44% | 21,834 | 31,191 |
| T1_EL-37_A | 12,459,394 | 582.23 | 84.84% | 76.58% | 21,459 | 31,499 |
| T1_EL-37_B | 12,307,919 | 575.15 | 83.73% | 75.60% | 21,498 | 31,659 |
| T1_EL-37_C | 12,304,724 | 575.00 | 85.63% | 77.01% | 21,853 | 31,867 |
| T1_EL-38_A | 12,509,663 | 584.58 | 82.74% | 75.01% | 21,090 | 31,301 |
| T1_EL-38_B | 12,402,869 | 579.59 | 83.65% | 75.77% | 21,259 | 31,423 |
| T1_EL-38_C | 12,275,935 | 573.65 | 84.49% | 76.52% | 21,154 | 31,347 |
| T2-EL-29_A | 11,831,500 | 552.89 | 89.18% | 79.40% | 23,537 | 33,355 |
| T2-EL-29_B | 12,567,883 | 587.30 | 87.24% | 77.37% | 23,287 | 33,191 |
| T2-EL-29_C | 11,703,832 | 546.92 | 86.33% | 76.55% | 23,049 | 32,709 |
| T2-EL-31_A | 11,838,848 | 553.23 | 87.76% | 77.48% | 22,434 | 31,315 |
| T2-EL-31_B | 12,327,520 | 576.07 | 83.16% | 73.52% | 22,630 | 31,975 |
| T2-EL-31_C | 12,139,580 | 567.28 | 87.12% | 76.92% | 22,721 | 31,888 |
| T2-EL-35_A | 12,534,482 | 585.74 | 85.42% | 76.53% | 21,532 | 30,324 |
| T2-EL-35_B | 11,902,501 | 556.20 | 85.58% | 76.42% | 21,530 | 30,428 |
| T2-EL-35_C | 11,747,441 | 548.96 | 82.89% | 73.93% | 21,901 | 30,816 |
| T2-EL-36_A | 12,167,990 | 568.61 | 87.12% | 78.03% | 21,458 | 30,964 |
| T2-EL-36_B | 12,106,436 | 565.73 | 87.03% | 78.25% | 21,134 | 30,622 |
| T2-EL-36_C | 12,329,007 | 576.14 | 86.44% | 77.72% | 21,440 | 30,954 |
| T2-EL-37_A | 12,391,213 | 579.04 | 85.28% | 76.57% | 22,146 | 32,695 |
| T2-EL-37_B | 11,747,023 | 548.94 | 84.18% | 75.59% | 21,983 | 32,372 |
| T2-EL-37_C | 12,353,616 | 577.28 | 85.99% | 77.32% | 21,512 | 31,889 |
| T2-EL-38_A | 12,468,993 | 582.68 | 84.50% | 76.46% | 21,478 | 31,964 |
| T2-EL-38_B | 12,888,956 | 602.30 | 84.20% | 76.12% | 21,465 | 31,979 |
| T2-EL-38_C | 11,701,650 | 546.82 | 84.49% | 76.59% | 21,169 | 31,231 |
| T8-EL-36_A | 11,930,700 | 557.52 | 85.13% | 76.32% | 22,354 | 31,935 |
| T8-EL-36_B | 11,955,144 | 558.66 | 86.70% | 77.84% | 22,175 | 31,584 |
| T8-EL-36_C | 12,587,578 | 588.22 | 85.65% | 76.76% | 22,428 | 32,304 |
| T8-EL-37_A | 12,633,206 | 590.35 | 84.10% | 75.75% | 22,014 | 32,251 |
| T8-EL-37_B | 11,829,939 | 552.81 | 83.28% | 74.91% | 22,059 | 32,088 |
| T8-EL-37_C | 12,528,158 | 585.44 | 83.92% | 75.46% | 22,359 | 32,678 |
| T8-EL-38_A | 12,228,083 | 571.42 | 82.77% | 75.05% | 21,417 | 31,710 |
| T8-EL-38_B | 12,374,849 | 578.28 | 82.86% | 75.13% | 21,801 | 32,251 |
| T8-EL-38_C | 11,926,651 | 557.33 | 82.85% | 75.13% | 21,309 | 31,401 |

T1: cluster bagging from 3 WAF until harvest; T2: control group; T8: cluster bagging at E-L 35 stage and bag removal at E-L 36 stage.
