# Supplementary figures and images for: Comparative physiological, metabolomic, and transcriptomic analyses reveal developmental stage-dependent effects of cluster bagging on phenolic metabolism in Cabernet Sauvignon grape berries
Source: BMC Plant Biol. 2019 Dec 26;19:583. doi: 10.1186/s12870-019-2186-z (PMC6933938; doi:10.1186/s12870-019-2186-z)

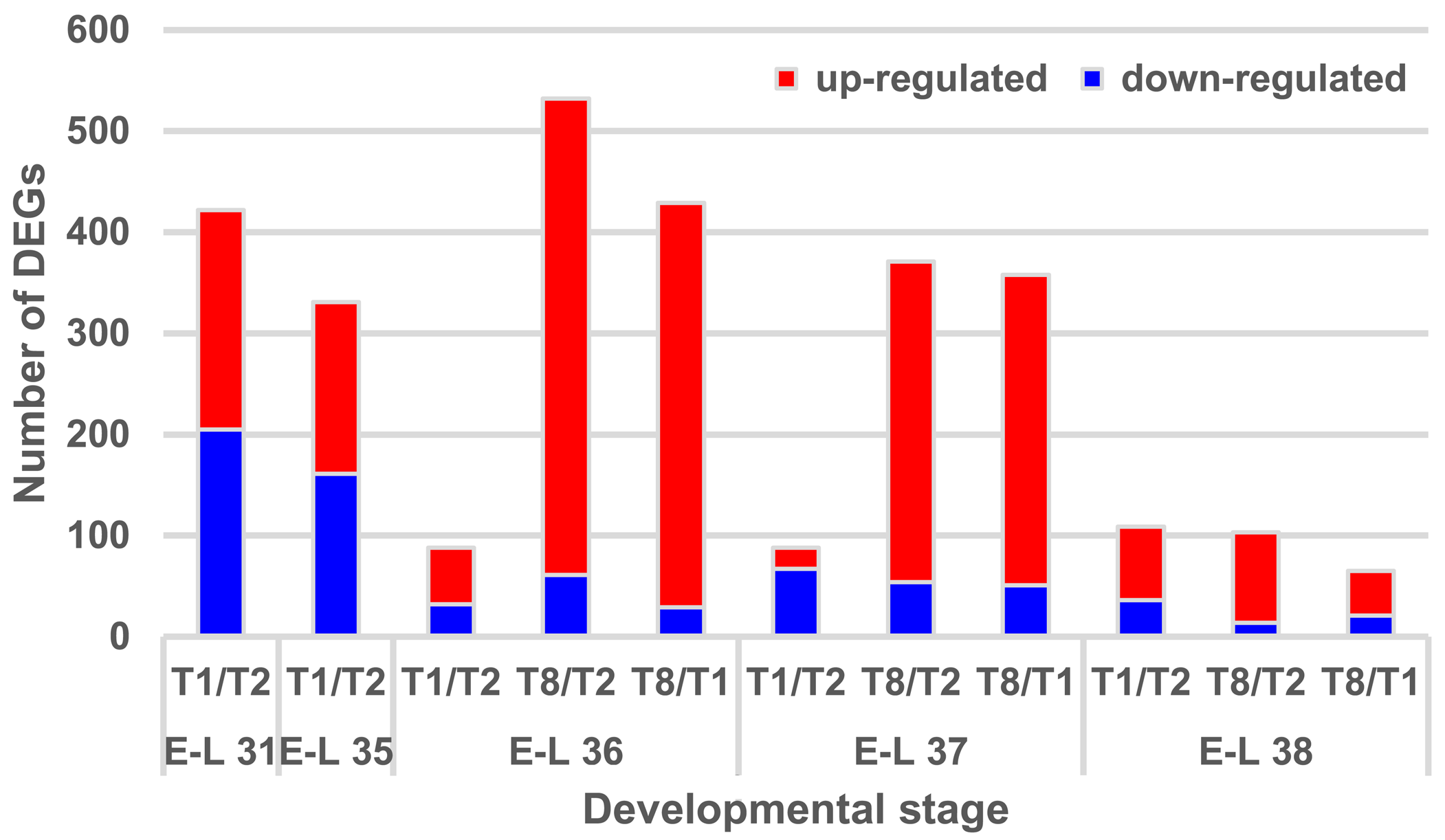

Supplement: Supplementary file 5 — Additional file 5: Figure S1. Number statistics of DEGs between the cluster bagging-treated and control grapes. [file 12870_2019_2186_MOESM5_ESM.tif]

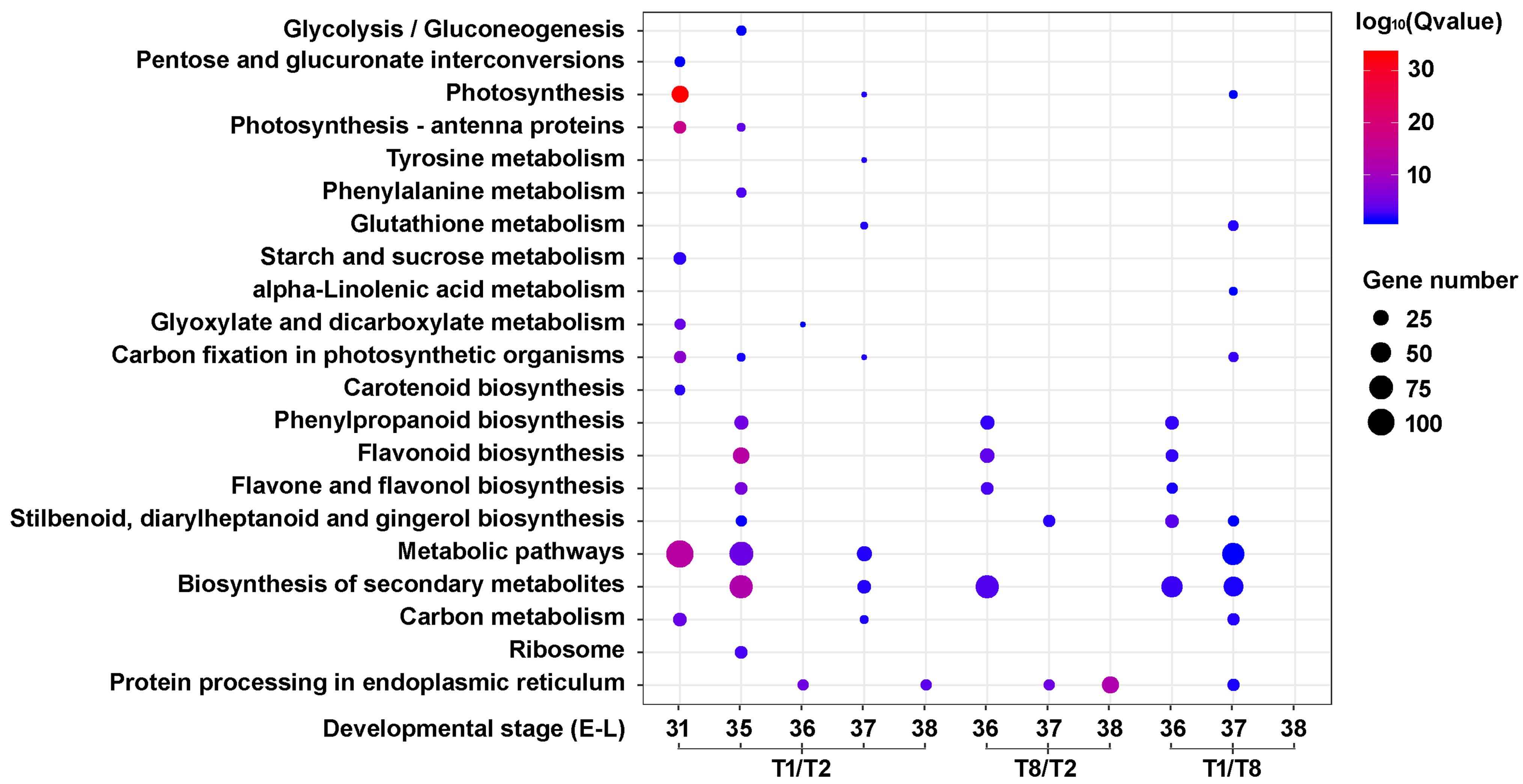

Supplement: Supplementary file 6 — Additional file 6: Figure S2. KEGG pathway enrichment analysis of DEGs between the cluster bagging-treated and control grapes. [file 12870_2019_2186_MOESM6_ESM.tif]

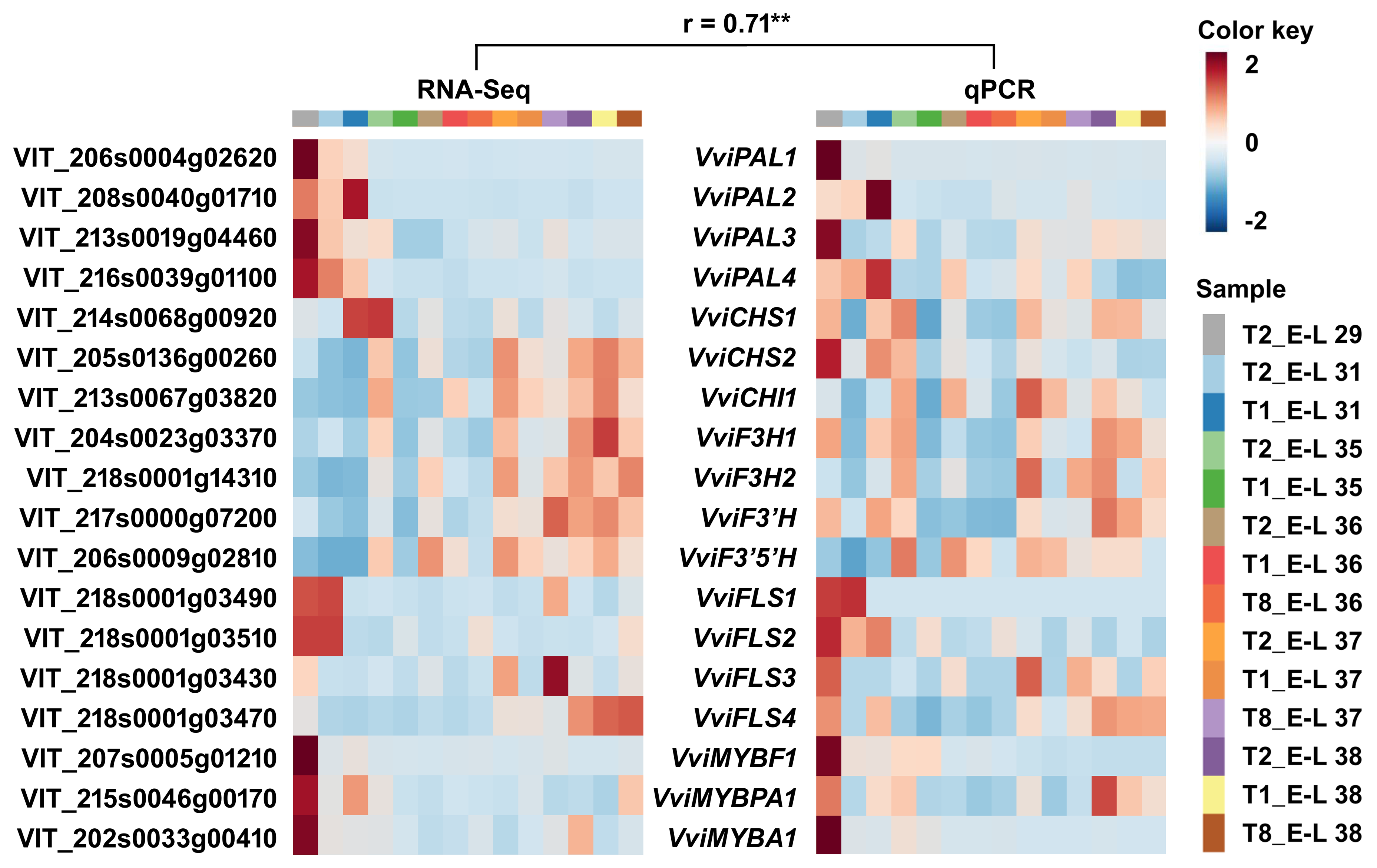

Supplement: Supplementary file 9 — Additional file 9: Figure S3. Validation of the RNA-Seq results by quantitative real-time PCR. The average FPKM values of the RNA-Seq analysis and the average relative expression values from three independent real-time PCR experiments are shown in the left-hand and right-hand heatmap, respectively. The correlation is significant at the 0.01 level. T1: cluster bagging from 3 WAF until harvest; T2: control group; T8: cluster bagging at E-L 35 stage and bag removal at E-L 36 stage. [file 12870_2019_2186_MOESM9_ESM.tif]

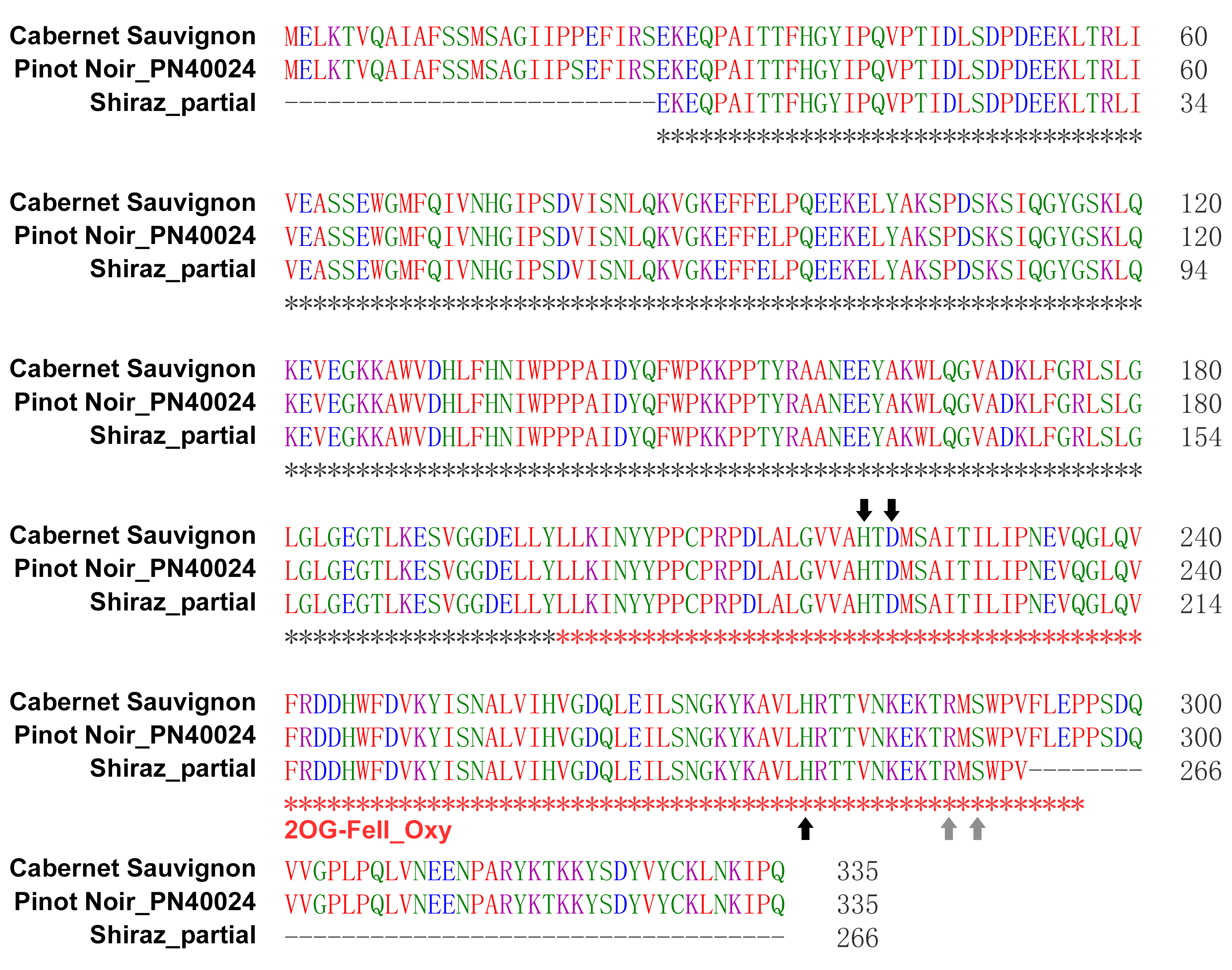

Supplement: Supplementary file 10 — Additional file 10: Figure S4. Multiple alignment of the deduced amino acid sequences of VviFLS4 from Cabernet Sauvignon, Pinot Noir, and Shiraz grape cultivars. [file 12870_2019_2186_MOESM10_ESM.tif]

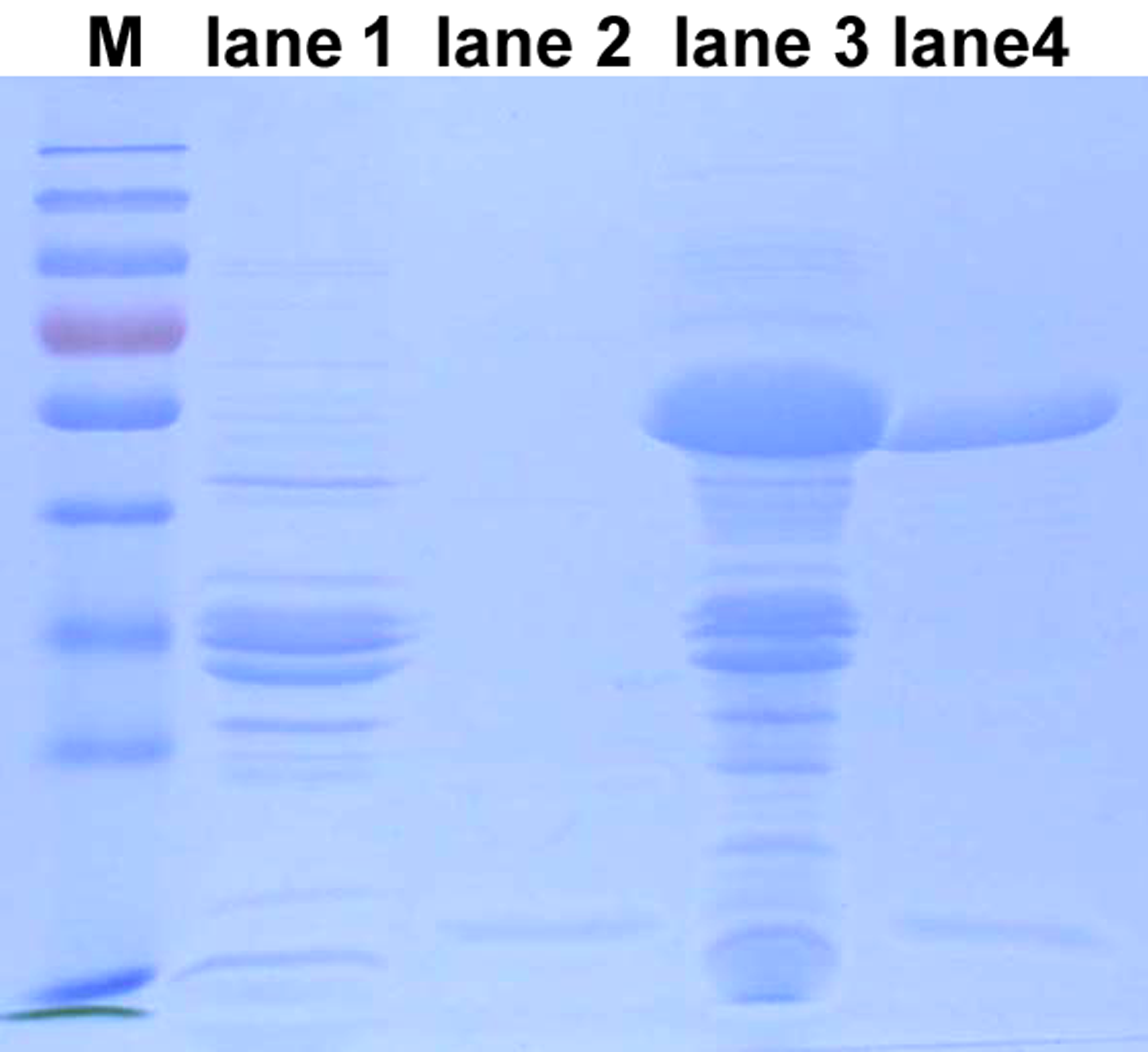

Supplement: Supplementary file 11 — Additional file 11: Figure S5. Purification of the prokaryotic expressed recombinant FLS4-His protein from Escherichia coli BL21(DE3) extracts induced with 1 mM isopropyl-β-D-thiogalactopyranoside. kDa, kilo Dalton; M, protein marker; Lanes 1 and 3, total proteins extracted from the empty vector-carrying (lane 1) and VviFLS4-His recombinant (lane 3) strain of E. coli; Lanes 2 and 4, the purified empty His-tag (lane 2) and VviFLS4-His recombinant (lane 4) protein fractions used for the enzymatic assay. [file 12870_2019_2186_MOESM11_ESM.tif]

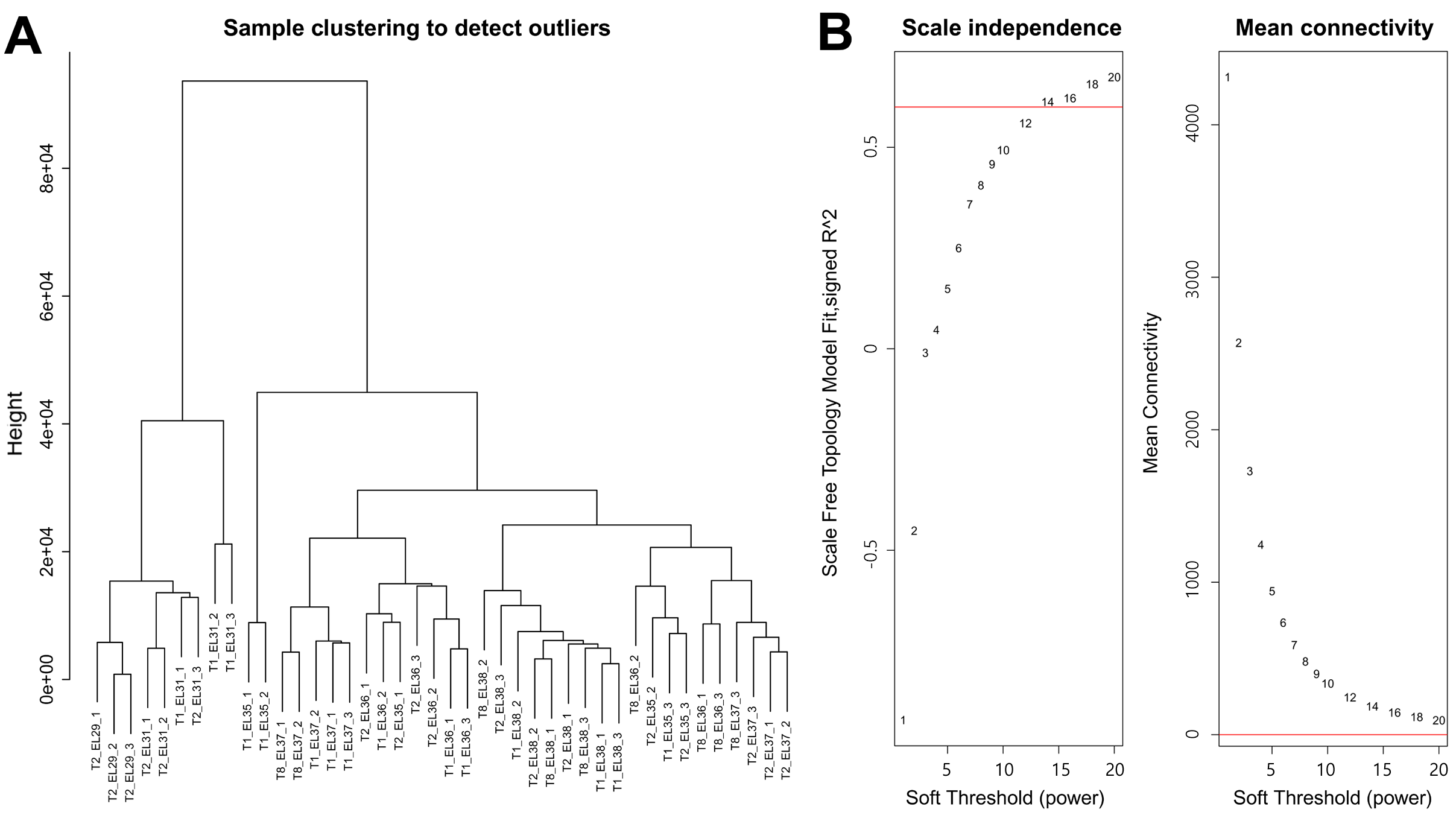

Supplement: Supplementary file 14 — Additional file 14: Figure S6. Pre-analysis for WGCNA. (a) Clustering dendrogram of samples based on their Euclidean distance. (b) Analysis of network topology for various soft-thresholding powers. [file 12870_2019_2186_MOESM14_ESM.tif]
