# Supplementary material for: Comparative physiological, metabolomic, and transcriptomic analyses reveal developmental stage-dependent effects of cluster bagging on phenolic metabolism in Cabernet Sauvignon grape berries
Source: BMC Plant Biol. 2019 Dec 26;19:583. doi: 10.1186/s12870-019-2186-z (PMC6933938; doi:10.1186/s12870-019-2186-z)
Supplement: Supplementary file 7 — Additional file 7: Table S5. Transcription profile of the phenolic metabolic pathway genes among samples. [file 12870_2019_2186_MOESM7_ESM.docx]

**Table S5.** Transcription profile of the phenolic metabolic pathway genes among samples.

| Family | Gene ID | Average expression level (FPKM) | | | | | | | | | | | | | |
| --- | --- | --- | --- | --- | --- | --- | --- | --- | --- | --- | --- | --- | --- | --- | --- |
|  |  | E-L 29 | E-L 29 | | E-L 29 | | E-L 29 | | | E-L 29 | | | E-L 29 | | |
|  |  | T2 | T2 | T1 | T2 | T1 | T2 | T1 | T8 | T2 | T1 | T8 | T2 | T1 | T8 |
| PAL | VIT_216s0039g01100 | 15.22 | 27.83 | 51.23 | 14.69 | 7.56 | 18.72 | 9.16 | 14.97 | 6.85 | 8.50 | 17.02 | 2.86 | 1.39 | 3.47 |
|  | VIT_200s2849g00010 | 19.30 | 19.50 | 28.56 | 5.91 | 3.42 | 8.89 | 3.91 | 5.23 | 4.87 | 5.54 | 5.46 | 3.28 | 1.65 | 3.72 |
|  | VIT_208s0040g01710 | 555.27 | 16.29 | 31.20 | 5.54 | 3.39 | 4.31 | 1.27 | 5.09 | 7.81 | 3.37 | 2.82 | 5.96 | 1.53 | 3.66 |
|  | VIT_216s0039g01120 | 7.81 | 11.84 | 21.41 | 5.06 | 2.36 | 6.58 | 3.84 | 8.27 | 3.21 | 3.60 | 9.13 | 1.44 | 0.47 | 1.58 |
|  | VIT_216s0039g01170 | 6.64 | 10.73 | 16.56 | 2.54 | 1.26 | 7.64 | 2.54 | 3.84 | 4.95 | 4.94 | 5.61 | 2.80 | 0.54 | 2.36 |
|  | VIT_206s0004g02620 | 62.51 | 11.23 | 46.52 | 51.78 | 6.51 | 18.38 | 8.88 | 10.28 | 23.14 | 17.37 | 15.50 | 22.83 | 22.46 | 16.53 |
|  | VIT_216s0039g01130 | 15.04 | 8.00 | 13.90 | 3.46 | 1.68 | 3.46 | 2.09 | 4.32 | 0.83 | 1.12 | 4.52 | 0.66 | 0.06 | 0.52 |
|  | VIT_216s0039g01110 | 5.82 | 3.08 | 7.62 | 1.23 | 0.92 | 1.26 | 0.72 | 1.08 | 0.45 | 0.53 | 1.58 | 0.10 | 0.00 | 0.00 |
|  | VIT_213s0019g04460 | 173.57 | 8.17 | 17.03 | 56.71 | 9.00 | 30.44 | 14.50 | 13.22 | 50.60 | 35.74 | 35.23 | 53.43 | 49.02 | 43.00 |
|  | VIT_216s0039g01320 | 0.07 | 1.22 | 2.90 | 0.01 | 0.00 | 1.22 | 0.25 | 0.17 | 1.72 | 1.29 | 1.70 | 2.16 | 1.48 | 1.53 |
|  | VIT_216s0039g01360 | 4.33 | 2.16 | 4.26 | 0.15 | 0.35 | 1.14 | 0.64 | 2.29 | 0.76 | 1.04 | 0.89 | 0.77 | 0.26 | 0.18 |
|  | VIT_211s0016g01520 | 2.31 | 1.76 | 6.99 | 0.33 | 0.30 | 0.10 | 0.13 | 0.56 | 0.51 | 0.44 | 0.69 | 0.63 | 0.39 | 0.33 |
|  | VIT_211s0016g01640 | 0.52 | 0.52 | 3.59 | 0.00 | 0.10 | 0.03 | 0.07 | 0.30 | 0.14 | 0.06 | 0.32 | 0.13 | 0.00 | 0.00 |
|  | VIT_211s0016g01660 | 1.07 | 1.16 | 3.51 | 0.03 | 0.31 | 0.00 | 0.00 | 0.40 | 0.19 | 0.15 | 0.14 | 0.00 | 0.00 | 0.22 |
|  | VIT_216s0039g01300 | 1.03 | 0.27 | 1.64 | 0.77 | 0.23 | 0.14 | 0.08 | 0.19 | 0.94 | 0.45 | 0.18 | 0.83 | 0.35 | 0.51 |
|  | VIT_216s0039g01280 | 1.89 | 1.09 | 1.70 | 1.06 | 0.47 | 1.08 | 0.00 | 0.53 | 1.57 | 0.48 | 0.24 | 0.20 | 0.09 | 0.63 |
|  | VIT_216s0039g01240 | 0.49 | 0.92 | 0.68 | 0.45 | 0.09 | 0.21 | 0.19 | 0.00 | 1.16 | 0.30 | 0.19 | 0.32 | 0.16 | 0.00 |
|  | VIT_211s0016g01535 | 0.00 | 0.00 | 0.00 | 0.00 | 0.00 | 0.00 | 0.00 | 0.00 | 0.00 | 0.00 | 0.00 | 0.00 | 0.06 | 0.00 |
|  | VIT_211s0016g01510 | 0.20 | 0.09 | 0.08 | 0.06 | 0.06 | 0.06 | 0.00 | 0.07 | 0.00 | 0.06 | 0.00 | 0.22 | 0.00 | 0.06 |
|  | VIT_200s2508g00010 | 0.00 | 0.00 | 0.00 | 0.00 | 0.00 | 0.00 | 0.00 | 0.00 | 0.47 | 0.47 | 0.00 | 0.14 | 0.02 | 0.63 |
|  | VIT_218s0111g00036 | 0.14 | 0.05 | 0.00 | 0.00 | 0.00 | 0.05 | 0.05 | 0.10 | 0.24 | 0.27 | 0.40 | 0.19 | 0.05 | 0.10 |
| C4H | VIT_206s0004g08150 | 369.40 | 33.94 | 86.04 | 62.78 | 19.78 | 44.40 | 30.30 | 27.06 | 69.37 | 59.42 | 45.22 | 94.89 | 86.81 | 58.44 |
|  | VIT_211s0065g00350 | 36.45 | 14.06 | 16.27 | 4.62 | 3.37 | 4.50 | 3.43 | 3.69 | 6.53 | 5.58 | 5.77 | 8.96 | 5.28 | 6.10 |
|  | VIT_211s0078g00290 | 8.33 | 1.14 | 1.73 | 0.21 | 0.19 | 0.19 | 0.10 | 0.15 | 0.31 | 0.46 | 0.31 | 0.55 | 0.23 | 0.46 |
| CCR | VIT_206s0004g07130 | 95.82 | 70.86 | 67.27 | 73.19 | 74.68 | 62.94 | 62.34 | 61.52 | 49.00 | 52.58 | 51.17 | 49.73 | 61.74 | 56.04 |
|  | VIT_210s0042g00640 | 17.61 | 8.67 | 21.06 | 1.25 | 4.61 | 1.01 | 1.03 | 8.81 | 1.04 | 1.01 | 7.27 | 0.40 | 1.24 | 3.37 |
|  | VIT_209s0070g00240 | 120.18 | 5.97 | 12.21 | 2.66 | 1.73 | 0.33 | 0.49 | 3.65 | 0.79 | 0.37 | 1.39 | 0.74 | 0.34 | 0.72 |
|  | VIT_218s0122g00640 | 0.12 | 0.23 | 0.18 | 0.00 | 0.00 | 0.11 | 0.06 | 0.06 | 0.00 | 0.17 | 0.12 | 0.12 | 0.12 | 0.18 |
|  | VIT_206s0004g02380 | 254.82 | 152.77 | 148.64 | 70.98 | 97.90 | 54.17 | 62.83 | 60.78 | 52.82 | 46.33 | 47.47 | 46.85 | 54.34 | 49.30 |
|  | VIT_214s0066g01150 | 12.90 | 31.22 | 40.19 | 5.72 | 7.91 | 5.57 | 4.07 | 7.82 | 7.56 | 7.85 | 12.17 | 4.33 | 3.87 | 4.25 |
|  | VIT_216s0039g01670 | 53.36 | 20.67 | 30.72 | 15.03 | 6.44 | 19.66 | 15.52 | 21.88 | 52.91 | 37.34 | 35.51 | 28.95 | 24.79 | 19.46 |
|  | VIT_206s0004g02370 | 36.54 | 24.30 | 29.32 | 9.47 | 12.77 | 3.93 | 5.36 | 4.80 | 6.73 | 3.83 | 4.46 | 2.17 | 2.71 | 3.06 |
|  | VIT_218s0122g00620 | 30.07 | 12.73 | 27.47 | 2.45 | 6.39 | 0.97 | 1.99 | 14.57 | 1.45 | 1.97 | 8.86 | 0.68 | 2.27 | 1.73 |
|  | VIT_202s0012g01570 | 21.18 | 10.22 | 27.99 | 34.92 | 22.30 | 22.31 | 18.02 | 16.64 | 29.91 | 33.29 | 23.26 | 38.63 | 37.23 | 26.24 |
|  | VIT_212s0035g02070 | 17.80 | 17.16 | 14.50 | 14.75 | 17.61 | 13.38 | 12.05 | 17.31 | 16.23 | 13.73 | 20.72 | 14.06 | 14.62 | 11.88 |
|  | VIT_218s0122g00630 | 3.83 | 12.13 | 11.41 | 8.18 | 9.40 | 8.07 | 10.24 | 9.33 | 8.49 | 7.21 | 8.06 | 3.21 | 2.80 | 3.63 |
|  | VIT_214s0083g00320 | 24.99 | 5.86 | 19.87 | 12.21 | 9.63 | 42.17 | 35.35 | 26.55 | 63.52 | 64.51 | 52.75 | 44.65 | 45.19 | 50.72 |
|  | VIT_213s0067g00620 | 9.46 | 10.34 | 7.47 | 4.22 | 3.99 | 2.56 | 2.87 | 2.58 | 2.52 | 1.90 | 1.66 | 1.47 | 0.98 | 2.13 |
|  | VIT_213s0047g00540 | 13.37 | 8.19 | 11.07 | 11.18 | 12.53 | 11.31 | 12.86 | 13.12 | 12.12 | 10.97 | 10.49 | 8.01 | 8.11 | 7.07 |
|  | VIT_213s0101g00240 | 8.57 | 8.63 | 7.12 | 8.93 | 10.48 | 9.13 | 9.01 | 8.32 | 11.12 | 8.93 | 7.46 | 5.03 | 4.47 | 5.73 |
|  | VIT_213s0064g00270 | 2.49 | 12.14 | 4.80 | 7.24 | 4.22 | 3.26 | 3.77 | 2.93 | 4.19 | 3.59 | 3.72 | 2.25 | 1.41 | 1.93 |
|  | VIT_213s0047g00940 | 7.57 | 4.76 | 5.64 | 5.41 | 4.16 | 4.84 | 4.73 | 4.95 | 6.54 | 5.41 | 5.12 | 2.86 | 4.00 | 5.14 |
|  | VIT_213s0064g00280 | 3.37 | 2.38 | 5.33 | 0.65 | 0.91 | 0.30 | 0.64 | 0.93 | 0.61 | 0.62 | 1.60 | 1.20 | 0.94 | 0.32 |
|  | VIT_203s0110g00350 | 4.83 | 6.00 | 4.48 | 3.85 | 3.55 | 2.84 | 2.74 | 3.05 | 3.10 | 2.89 | 2.41 | 1.90 | 1.40 | 0.96 |
|  | VIT_213s0064g00290 | 1.60 | 3.06 | 3.81 | 1.35 | 1.38 | 0.82 | 0.87 | 1.12 | 1.14 | 1.21 | 0.93 | 0.42 | 0.32 | 0.68 |
|  | VIT_201s0011g03480 | 0.15 | 2.63 | 3.91 | 2.78 | 1.83 | 1.10 | 2.86 | 2.37 | 1.20 | 0.97 | 1.48 | 0.22 | 0.72 | 0.74 |
|  | VIT_213s0047g00770 | 2.11 | 1.94 | 1.21 | 1.32 | 0.85 | 0.58 | 0.53 | 0.87 | 0.70 | 0.53 | 0.30 | 0.46 | 0.07 | 0.38 |
|  | VIT_215s0107g00210 | 0.94 | 2.87 | 1.15 | 1.82 | 0.98 | 0.45 | 0.58 | 0.80 | 0.71 | 0.66 | 1.17 | 0.59 | 0.26 | 0.66 |
|  | VIT_203s0038g04220 | 2.85 | 0.84 | 1.15 | 0.26 | 0.49 | 0.06 | 0.00 | 0.06 | 0.00 | 0.00 | 0.00 | 0.06 | 0.06 | 0.00 |
|  | VIT_203s0110g00310 | 0.21 | 0.60 | 0.38 | 0.22 | 0.22 | 0.15 | 0.07 | 0.07 | 0.24 | 0.07 | 0.14 | 0.15 | 0.07 | 0.16 |
|  | VIT_213s0019g01160 | 0.18 | 0.00 | 1.14 | 0.00 | 0.00 | 0.00 | 0.00 | 0.12 | 0.00 | 0.00 | 0.13 | 0.00 | 0.00 | 0.00 |
|  | VIT_213s0067g00530 | 0.00 | 0.06 | 0.19 | 0.00 | 0.12 | 0.00 | 0.06 | 0.12 | 0.00 | 0.00 | 0.06 | 0.00 | 0.00 | 0.00 |
|  | VIT_212s0142g00710 | 0.18 | 0.00 | 0.06 | 0.00 | 0.00 | 0.00 | 0.06 | 0.00 | 0.00 | 0.06 | 0.00 | 0.07 | 0.00 | 0.07 |
|  | VIT_213s0047g00550 | 0.32 | 0.17 | 0.18 | 0.23 | 0.05 | 0.12 | 0.11 | 0.06 | 0.28 | 0.11 | 0.10 | 0.06 | 0.07 | 0.00 |
|  | VIT_212s0142g00510 | 0.39 | 0.00 | 0.00 | 0.00 | 0.00 | 0.00 | 0.00 | 0.00 | 0.00 | 0.00 | 0.00 | 0.00 | 0.00 | 0.00 |
|  | VIT_213s0067g00580 | 0.00 | 0.00 | 0.26 | 0.00 | 0.13 | 0.00 | 0.00 | 0.13 | 0.00 | 0.13 | 0.00 | 0.00 | 0.00 | 0.00 |
|  | VIT_213s0067g00690 | 0.00 | 0.00 | 0.06 | 0.00 | 0.00 | 0.00 | 0.00 | 0.00 | 0.00 | 0.00 | 0.00 | 0.00 | 0.00 | 0.00 |
|  | VIT_213s0101g00250 | 0.00 | 0.55 | 0.35 | 0.08 | 0.07 | 0.14 | 0.00 | 0.00 | 0.00 | 0.10 | 0.08 | 0.07 | 0.00 | 0.11 |
|  | VIT_203s0110g00330 | 0.00 | 0.00 | 0.00 | 0.00 | 0.00 | 0.00 | 0.00 | 0.00 | 0.00 | 0.00 | 0.00 | 0.00 | 0.21 | 0.00 |
|  | VIT_213s0067g00460 | 0.00 | 0.22 | 0.00 | 0.00 | 0.00 | 0.00 | 0.06 | 0.17 | 0.00 | 0.11 | 0.18 | 0.16 | 0.00 | 0.00 |
|  | VIT_213s0067g00590 | 0.06 | 0.18 | 0.06 | 0.00 | 0.00 | 0.00 | 0.00 | 0.00 | 0.00 | 0.00 | 0.00 | 0.12 | 0.00 | 0.00 |
|  | VIT_213s0047g00990 | 0.07 | 0.14 | 0.00 | 0.00 | 0.09 | 0.09 | 0.28 | 0.06 | 0.00 | 0.10 | 0.00 | 0.00 | 0.00 | 0.05 |
|  | VIT_211s0037g01030 | 0.00 | 0.00 | 0.00 | 0.00 | 0.07 | 0.00 | 0.00 | 0.00 | 0.00 | 0.00 | 0.00 | 0.00 | 0.00 | 0.00 |
|  | VIT_213s0101g00330 | 0.00 | 0.16 | 0.00 | 0.16 | 0.00 | 0.00 | 0.00 | 0.00 | 0.00 | 0.00 | 0.00 | 0.00 | 0.00 | 0.00 |
|  | VIT_203s0110g00320 | 0.00 | 0.00 | 0.00 | 0.00 | 0.00 | 0.00 | 0.00 | 0.00 | 0.00 | 0.00 | 0.00 | 0.15 | 0.00 | 0.00 |
|  | VIT_213s0067g00560 | 0.00 | 0.00 | 0.00 | 0.00 | 0.00 | 0.00 | 0.21 | 0.00 | 0.00 | 0.00 | 0.00 | 0.00 | 0.00 | 0.00 |
|  | VIT_213s0047g00760 | 0.37 | 0.00 | 0.48 | 0.44 | 0.55 | 0.67 | 0.94 | 0.00 | 0.42 | 0.41 | 0.00 | 0.18 | 0.00 | 0.17 |
|  | VIT_218s0122g00650 | 0.28 | 0.00 | 0.27 | 0.00 | 0.00 | 0.00 | 0.00 | 0.28 | 0.00 | 0.00 | 0.00 | 0.00 | 0.00 | 0.00 |
|  | VIT_213s0067g00680 | 0.00 | 0.07 | 0.00 | 0.00 | 0.00 | 0.00 | 0.00 | 0.07 | 0.00 | 0.00 | 0.15 | 0.00 | 0.00 | 0.08 |
|  | VIT_213s0047g00735 | 0.00 | 0.00 | 0.00 | 0.00 | 0.00 | 0.00 | 0.00 | 0.00 | 0.00 | 0.00 | 0.00 | 0.00 | 0.00 | 0.00 |
| CAD | VIT_200s0615g00030 | 124.54 | 139.11 | 100.81 | 20.31 | 18.16 | 17.63 | 15.89 | 13.40 | 5.00 | 4.37 | 7.06 | 2.57 | 1.25 | 2.56 |
|  | VIT_200s0371g00050 | 50.01 | 129.66 | 78.10 | 155.55 | 135.27 | 143.73 | 176.40 | 162.62 | 61.12 | 102.83 | 40.19 | 33.68 | 36.57 | 30.61 |
|  | VIT_200s0371g00100 | 36.23 | 54.54 | 55.43 | 6.73 | 10.96 | 3.26 | 4.31 | 12.24 | 1.71 | 1.29 | 6.13 | 0.93 | 1.17 | 4.74 |
|  | VIT_203s0180g00260 | 126.00 | 48.45 | 54.55 | 48.36 | 44.36 | 28.79 | 33.42 | 36.44 | 55.31 | 39.58 | 43.29 | 54.97 | 45.94 | 46.94 |
|  | VIT_202s0025g03100 | 29.49 | 24.19 | 20.10 | 15.02 | 12.41 | 14.83 | 11.67 | 11.19 | 29.82 | 26.16 | 23.13 | 27.35 | 26.52 | 27.41 |
|  | VIT_204s0044g00210 | 2.08 | 5.16 | 27.54 | 0.16 | 11.68 | 1.18 | 1.84 | 66.19 | 0.56 | 2.25 | 29.80 | 1.73 | 14.99 | 16.87 |
|  | VIT_200s0615g00020 | 11.60 | 8.63 | 7.87 | 1.36 | 0.71 | 0.63 | 0.69 | 1.76 | 0.00 | 0.26 | 2.40 | 0.66 | 0.35 | 0.66 |
|  | VIT_200s0346g00080 | 2.86 | 3.43 | 3.64 | 0.25 | 0.46 | 0.17 | 0.00 | 0.85 | 0.00 | 0.41 | 0.00 | 0.24 | 0.13 | 0.00 |
|  | VIT_204s0044g00190 | 157.96 | 2.89 | 7.87 | 1.26 | 1.66 | 1.55 | 1.24 | 1.41 | 1.28 | 1.12 | 1.04 | 0.86 | 0.62 | 0.59 |
|  | VIT_218s0001g01160 | 0.91 | 6.01 | 0.98 | 2.43 | 1.81 | 0.13 | 0.26 | 0.54 | 0.52 | 0.13 | 0.27 | 0.00 | 0.00 | 0.00 |
|  | VIT_200s0346g00110 | 6.89 | 4.73 | 3.18 | 21.76 | 11.56 | 21.65 | 26.69 | 12.28 | 7.58 | 14.84 | 8.86 | 3.68 | 5.77 | 2.12 |
|  | VIT_200s0218g00010 | 2.82 | 3.04 | 1.51 | 0.07 | 0.11 | 0.48 | 0.41 | 0.34 | 0.18 | 0.00 | 0.08 | 0.07 | 0.16 | 0.17 |
|  | VIT_218s0001g14910 | 15.50 | 2.44 | 1.31 | 6.23 | 2.20 | 8.25 | 5.78 | 2.71 | 6.33 | 4.29 | 3.56 | 1.27 | 0.79 | 0.66 |
|  | VIT_200s1389g00010 | 3.26 | 0.41 | 1.69 | 7.56 | 2.57 | 10.83 | 7.48 | 3.37 | 3.05 | 4.39 | 3.35 | 0.60 | 1.17 | 1.54 |
|  | VIT_207s0129g01030 | 0.08 | 0.06 | 0.37 | 0.06 | 0.22 | 0.11 | 0.05 | 0.29 | 0.12 | 0.34 | 0.11 | 0.29 | 0.23 | 0.33 |
|  | VIT_203s0180g00250 | 1.17 | 0.30 | 0.43 | 0.72 | 1.41 | 0.41 | 0.89 | 2.74 | 0.25 | 0.42 | 0.00 | 0.24 | 0.42 | 0.12 |
|  | VIT_210s0003g04910 | 0.00 | 0.00 | 0.06 | 0.00 | 0.00 | 0.00 | 0.00 | 0.00 | 0.00 | 0.06 | 0.00 | 0.00 | 0.00 | 0.00 |
|  | VIT_215s0048g01710 | 0.00 | 0.08 | 0.08 | 0.00 | 0.00 | 0.00 | 0.00 | 0.08 | 0.04 | 0.00 | 0.04 | 0.00 | 0.00 | 0.00 |
|  | VIT_200s0346g00100 | 0.27 | 0.28 | 0.04 | 0.00 | 0.00 | 0.00 | 0.00 | 0.00 | 0.00 | 0.00 | 0.00 | 0.00 | 0.00 | 0.00 |
|  | VIT_218s0122g00450 | 0.00 | 0.00 | 0.04 | 0.00 | 0.05 | 0.00 | 0.00 | 2.21 | 0.05 | 0.15 | 1.51 | 0.27 | 0.24 | 0.20 |
|  | VIT_200s0174g00270 | 0.08 | 0.04 | 0.04 | 0.00 | 0.00 | 0.00 | 0.00 | 0.04 | 0.00 | 0.04 | 0.04 | 0.03 | 0.00 | 0.04 |
|  | VIT_204s0044g00200 | 0.00 | 0.00 | 0.00 | 0.00 | 0.00 | 0.00 | 0.00 | 1.28 | 0.00 | 0.00 | 0.00 | 0.00 | 0.00 | 0.00 |
|  | VIT_200s0346g00120 | 0.00 | 0.00 | 0.00 | 0.00 | 0.00 | 0.00 | 0.00 | 0.00 | 0.00 | 0.00 | 0.00 | 0.00 | 0.00 | 0.00 |
|  | VIT_200s0615g00010 | 1.87 | 0.69 | 1.13 | 5.80 | 3.46 | 5.28 | 5.05 | 2.86 | 2.94 | 4.40 | 2.26 | 3.87 | 3.22 | 1.71 |
|  | VIT_200s0371g00060 | 0.09 | 0.00 | 0.00 | 0.00 | 0.00 | 0.00 | 0.00 | 0.00 | 0.00 | 0.00 | 0.00 | 0.00 | 0.00 | 0.00 |
|  | VIT_202s0025g03110 | 0.00 | 0.00 | 0.00 | 0.00 | 0.09 | 0.00 | 0.00 | 0.00 | 0.00 | 0.00 | 0.00 | 0.00 | 0.00 | 0.00 |
|  | VIT_200s0371g00010 | 0.17 | 0.00 | 0.00 | 0.00 | 0.00 | 0.27 | 0.00 | 0.00 | 0.00 | 0.00 | 0.00 | 0.00 | 0.00 | 0.00 |
|  | VIT_200s0371g00040 | 0.14 | 0.00 | 0.00 | 0.00 | 0.00 | 0.07 | 0.00 | 0.00 | 0.35 | 0.00 | 0.21 | 0.00 | 0.00 | 0.07 |
| 4CL | VIT_206s0061g00450 | 76.83 | 71.87 | 64.36 | 39.89 | 48.40 | 34.18 | 31.53 | 40.14 | 28.40 | 22.84 | 31.77 | 22.70 | 21.76 | 23.62 |
|  | VIT_202s0025g03660 | 32.91 | 51.18 | 53.02 | 16.85 | 22.87 | 13.14 | 13.68 | 21.93 | 15.38 | 14.95 | 22.58 | 15.91 | 13.64 | 13.83 |
|  | VIT_211s0052g01090 | 373.22 | 24.34 | 49.63 | 9.74 | 9.06 | 7.51 | 5.01 | 7.04 | 9.35 | 6.90 | 5.85 | 14.91 | 7.01 | 10.98 |
|  | VIT_216s0050g00390 | 24.47 | 35.44 | 27.25 | 64.04 | 83.44 | 67.44 | 78.69 | 102.50 | 84.18 | 95.57 | 87.86 | 96.78 | 109.99 | 92.23 |
|  | VIT_216s0039g02040 | 58.65 | 17.82 | 30.47 | 12.46 | 3.42 | 6.67 | 3.16 | 5.09 | 9.04 | 6.07 | 6.21 | 16.14 | 12.36 | 10.69 |
|  | VIT_206s0004g01560 | 0.18 | 0.35 | 0.30 | 0.29 | 0.19 | 0.70 | 0.47 | 0.64 | 1.22 | 1.38 | 1.07 | 0.91 | 1.03 | 0.78 |
|  | VIT_211s0052g01110 | 0.26 | 0.02 | 0.13 | 0.07 | 0.00 | 0.04 | 0.00 | 0.13 | 0.04 | 0.04 | 0.24 | 0.02 | 0.00 | 0.09 |
|  | VIT_208s0007g05050 | 0.00 | 0.00 | 0.07 | 0.04 | 0.00 | 0.04 | 0.00 | 0.00 | 0.00 | 0.00 | 0.00 | 0.00 | 0.00 | 0.07 |
|  | VIT_201s0010g03720 | 0.04 | 0.00 | 0.00 | 0.00 | 0.00 | 0.04 | 0.07 | 0.07 | 0.08 | 0.07 | 0.15 | 0.04 | 0.04 | 0.00 |
|  | VIT_214s0036g00870 | 0.00 | 0.03 | 0.00 | 0.00 | 0.00 | 0.00 | 0.00 | 0.00 | 0.00 | 0.03 | 0.00 | 0.00 | 0.00 | 0.00 |
| CHS | VIT_214s0068g00920 | 242.02 | 50.27 | 221.14 | 247.59 | 42.26 | 142.55 | 78.82 | 75.72 | 213.06 | 151.95 | 139.50 | 219.14 | 215.30 | 141.87 |
|  | VIT_205s0136g00260 | 242.74 | 57.88 | 215.50 | 912.34 | 221.79 | 784.59 | 579.64 | 357.93 | 758.89 | 670.13 | 490.00 | 571.63 | 560.87 | 371.74 |
|  | VIT_203s0038g01460 | 0.63 | 0.64 | 0.89 | 0.61 | 0.62 | 0.72 | 0.63 | 0.99 | 1.46 | 1.17 | 0.93 | 2.22 | 1.73 | 1.12 |
|  | VIT_215s0021g02170 | 0.09 | 0.00 | 0.09 | 0.00 | 0.00 | 0.14 | 0.09 | 0.10 | 0.05 | 0.00 | 0.05 | 0.18 | 0.19 | 0.05 |
|  | VIT_216s0022g01020 | 0.30 | 0.00 | 0.00 | 0.00 | 0.06 | 0.49 | 0.43 | 0.06 | 0.65 | 0.55 | 0.33 | 0.06 | 0.00 | 0.09 |
|  | VIT_216s0022g01190 | 0.00 | 0.00 | 0.07 | 0.03 | 0.00 | 0.12 | 0.51 | 0.00 | 0.05 | 0.03 | 0.04 | 0.07 | 0.00 | 0.11 |
|  | VIT_200s1492g00010 | 0.71 | 0.00 | 0.18 | 0.34 | 0.00 | 1.01 | 0.00 | 0.34 | 0.57 | 0.34 | 0.47 | 0.33 | 0.35 | 0.00 |
|  | VIT_216s0022g01140 | 0.14 | 0.00 | 0.00 | 0.03 | 0.00 | 0.48 | 0.05 | 0.00 | 0.05 | 0.03 | 0.04 | 0.00 | 0.00 | 0.00 |
| CHI | VIT_213s0067g02870 | 117.55 | 40.61 | 82.90 | 62.48 | 24.58 | 41.90 | 34.70 | 27.14 | 47.62 | 37.77 | 34.77 | 39.77 | 27.09 | 25.56 |
|  | VIT_213s0067g03820 | 57.38 | 29.09 | 50.76 | 85.06 | 24.33 | 82.62 | 52.07 | 33.02 | 104.08 | 78.55 | 54.83 | 80.61 | 63.61 | 49.19 |
| F3H | VIT_204s0023g03370 | 144.41 | 31.79 | 117.87 | 129.77 | 26.04 | 54.99 | 35.37 | 33.53 | 106.27 | 72.79 | 63.01 | 144.43 | 129.61 | 92.35 |
|  | VIT_218s0001g14310 | 73.18 | 47.42 | 74.02 | 107.77 | 52.68 | 76.27 | 49.11 | 51.52 | 126.98 | 59.42 | 107.68 | 119.42 | 67.47 | 100.62 |
|  | VIT_200s0521g00030 | 1.73 | 0.24 | 1.00 | 0.38 | 0.00 | 0.00 | 0.00 | 0.00 | 0.00 | 0.00 | 0.00 | 0.00 | 0.00 | 0.00 |
|  | VIT_216s0098g00860 | 1.56 | 1.04 | 1.18 | 1.47 | 3.91 | 3.58 | 6.15 | 6.30 | 0.81 | 1.88 | 1.03 | 1.16 | 0.69 | 1.63 |
|  | VIT_200s0687g00020 | 0.50 | 1.01 | 0.63 | 0.00 | 0.76 | 0.00 | 0.39 | 0.38 | 0.19 | 0.35 | 0.00 | 0.57 | 0.00 | 0.19 |
|  | VIT_213s0047g00210 | 2.29 | 6.10 | 3.98 | 3.63 | 3.11 | 2.01 | 1.85 | 1.57 | 0.70 | 0.17 | 0.53 | 0.28 | 0.11 | 0.04 |
|  | VIT_203s0063g01210 | 0.60 | 0.92 | 1.31 | 0.48 | 0.18 | 0.44 | 0.15 | 0.51 | 0.59 | 0.28 | 1.39 | 0.14 | 0.34 | 0.75 |
|  | VIT_206s0004g00760 | 0.24 | 0.27 | 0.34 | 0.14 | 0.13 | 0.03 | 0.14 | 0.17 | 0.12 | 0.03 | 0.04 | 0.28 | 0.27 | 0.32 |
|  | VIT_203s0063g01280 | 0.16 | 0.30 | 0.00 | 0.21 | 0.06 | 0.00 | 0.20 | 0.35 | 0.19 | 0.06 | 0.14 | 0.00 | 0.08 | 0.00 |
| F3'H | VIT_217s0000g07200 | 75.10 | 42.56 | 74.97 | 58.31 | 28.26 | 28.36 | 26.38 | 27.12 | 58.09 | 48.08 | 52.95 | 89.74 | 82.38 | 65.18 |
|  | VIT_217s0000g07210 | 17.91 | 13.26 | 25.74 | 16.65 | 7.49 | 9.43 | 6.75 | 6.57 | 16.18 | 10.75 | 9.91 | 25.02 | 19.93 | 16.15 |
|  | VIT_202s0025g04864 | 8.80 | 104.62 | 68.07 | 261.29 | 174.84 | 155.96 | 154.26 | 172.54 | 123.77 | 163.85 | 131.92 | 145.58 | 158.21 | 134.62 |
|  | VIT_209s0002g01090 | 30.96 | 37.35 | 35.60 | 37.64 | 32.26 | 19.23 | 28.44 | 29.98 | 19.48 | 18.93 | 10.71 | 8.32 | 6.25 | 7.08 |
|  | VIT_211s0016g01020 | 9.43 | 8.64 | 8.68 | 9.32 | 1.78 | 3.42 | 4.07 | 2.45 | 2.24 | 1.40 | 1.42 | 0.06 | 0.06 | 0.03 |
|  | VIT_202s0025g04866 | 0.12 | 0.60 | 1.04 | 3.52 | 2.94 | 1.61 | 1.03 | 1.28 | 1.01 | 1.27 | 1.04 | 1.61 | 2.48 | 1.48 |
|  | VIT_211s0016g01030 | 0.72 | 0.45 | 0.37 | 0.35 | 0.15 | 0.47 | 0.11 | 0.18 | 0.00 | 0.00 | 0.13 | 0.00 | 0.00 | 0.00 |
|  | VIT_202s0109g00290 | 0.00 | 0.00 | 0.15 | 0.00 | 0.00 | 0.00 | 0.00 | 0.00 | 0.00 | 0.00 | 0.32 | 0.00 | 0.15 | 0.00 |
|  | VIT_211s0016g01050 | 0.14 | 2.14 | 1.35 | 0.30 | 0.38 | 0.11 | 0.34 | 0.10 | 0.00 | 0.07 | 0.03 | 0.04 | 0.00 | 0.07 |
|  | VIT_215s0046g00330 | 0.00 | 0.15 | 0.19 | 0.00 | 0.00 | 0.00 | 0.00 | 0.19 | 0.04 | 0.00 | 0.00 | 0.04 | 0.00 | 0.00 |
|  | VIT_200s0555g00020 | 0.05 | 0.03 | 0.06 | 0.00 | 0.03 | 0.00 | 0.00 | 0.14 | 0.17 | 0.03 | 0.14 | 0.17 | 0.00 | 0.18 |
|  | VIT_215s0048g01500 | 0.00 | 0.22 | 0.00 | 0.00 | 0.09 | 0.00 | 0.00 | 0.00 | 0.00 | 0.00 | 0.00 | 0.00 | 0.00 | 0.00 |
|  | VIT_217s0000g07220 | 0.00 | 0.00 | 0.00 | 0.00 | 0.00 | 0.00 | 0.00 | 0.00 | 0.00 | 0.00 | 0.15 | 0.00 | 0.15 | 0.00 |
|  | VIT_211s0016g00980 | 0.00 | 0.25 | 0.17 | 0.18 | 0.00 | 0.00 | 0.00 | 0.00 | 0.00 | 0.06 | 0.04 | 0.00 | 0.00 | 0.03 |
|  | VIT_202s0012g02380 | 0.00 | 0.00 | 0.00 | 0.00 | 0.00 | 0.13 | 0.10 | 0.00 | 0.12 | 0.00 | 0.00 | 0.00 | 0.00 | 0.00 |
| F3'5'H | VIT_208s0007g05160 | 7.03 | 8.46 | 10.77 | 5.44 | 5.57 | 4.77 | 5.30 | 4.95 | 8.00 | 6.85 | 7.27 | 9.23 | 9.59 | 7.51 |
|  | VIT_206s0009g02840 | 29.60 | 4.72 | 9.80 | 54.46 | 6.49 | 40.70 | 23.70 | 10.75 | 55.46 | 50.67 | 30.84 | 44.38 | 57.75 | 30.48 |
|  | VIT_206s0009g02805 | 27.08 | 1.48 | 7.07 | 88.60 | 13.33 | 55.02 | 33.67 | 18.99 | 77.71 | 51.28 | 44.44 | 37.07 | 56.49 | 21.14 |
|  | VIT_206s0009g02810 | 28.48 | 2.85 | 6.06 | 81.42 | 13.09 | 56.64 | 40.97 | 24.89 | 90.74 | 77.29 | 56.16 | 70.02 | 83.17 | 51.19 |
|  | VIT_206s0009g03000 | 4.18 | 0.98 | 2.48 | 10.56 | 1.80 | 9.60 | 5.56 | 5.19 | 25.65 | 20.90 | 12.92 | 19.18 | 20.15 | 12.39 |
|  | VIT_206s0009g02880 | 3.03 | 0.83 | 0.70 | 0.07 | 0.00 | 8.65 | 4.13 | 0.00 | 0.00 | 1.79 | 0.00 | 0.00 | 0.00 | 0.00 |
|  | VIT_206s0009g03010 | 6.23 | 1.45 | 1.51 | 18.71 | 2.13 | 13.20 | 13.20 | 8.73 | 32.52 | 29.76 | 15.34 | 27.52 | 34.47 | 14.55 |
|  | VIT_206s0009g02860 | 1.94 | 0.13 | 0.59 | 0.29 | 0.18 | 0.00 | 0.00 | 0.00 | 0.00 | 1.10 | 0.00 | 0.00 | 0.00 | 0.15 |
|  | VIT_206s0009g03050 | 0.00 | 0.07 | 0.01 | 0.00 | 0.00 | 0.00 | 0.00 | 0.00 | 0.00 | 0.00 | 0.00 | 0.00 | 0.00 | 0.00 |
|  | VIT_206s0009g02830 | 37.49 | 3.35 | 2.05 | 139.14 | 14.03 | 93.41 | 61.86 | 43.18 | 120.51 | 90.93 | 85.22 | 80.31 | 100.18 | 61.39 |
|  | VIT_206s0009g02910 | 0.00 | 0.00 | 0.00 | 0.00 | 0.00 | 0.00 | 0.00 | 0.00 | 0.00 | 0.00 | 0.10 | 0.00 | 0.00 | 0.11 |
|  | VIT_206s0009g03016 | 0.00 | 0.00 | 0.00 | 0.24 | 0.00 | 0.00 | 0.00 | 0.00 | 0.25 | 0.25 | 0.00 | 0.33 | 0.21 | 0.00 |
|  | VIT_206s0009g03040 | 0.00 | 0.08 | 0.00 | 0.07 | 0.00 | 0.00 | 0.00 | 0.00 | 0.00 | 0.00 | 0.00 | 0.08 | 0.00 | 0.00 |
|  | VIT_206s0009g03140 | 2.39 | 0.00 | 0.00 | 0.00 | 0.00 | 0.00 | 0.00 | 0.00 | 0.00 | 0.00 | 0.00 | 0.00 | 0.00 | 0.04 |
|  | VIT_206s0009g02873 | 0.14 | 0.44 | 0.00 | 0.89 | 0.42 | 2.12 | 0.33 | 0.47 | 1.57 | 0.91 | 1.46 | 0.09 | 0.00 | 0.00 |
|  | VIT_206s0009g02970 | 25.09 | 1.53 | 0.57 | 72.52 | 10.93 | 61.17 | 46.89 | 22.23 | 68.07 | 64.41 | 48.61 | 62.29 | 90.30 | 41.22 |
|  | VIT_206s0009g02920 | 2.26 | 0.35 | 0.16 | 4.53 | 0.12 | 1.18 | 0.83 | 0.79 | 1.90 | 0.86 | 1.61 | 1.55 | 1.00 | 0.33 |
|  | VIT_206s0009g03013 | 0.26 | 0.42 | 0.00 | 0.00 | 0.00 | 0.49 | 0.18 | 0.19 | 1.66 | 1.38 | 0.57 | 0.93 | 0.79 | 0.80 |
| FLS | VIT_202s0012g00320 | 1.29 | 1.70 | 1.24 | 0.92 | 0.67 | 0.46 | 0.55 | 0.64 | 0.43 | 0.35 | 0.43 | 0.51 | 0.98 | 0.44 |
|  | VIT_202s0012g00360 | 7.84 | 9.92 | 9.21 | 8.63 | 11.72 | 3.46 | 5.49 | 6.42 | 1.62 | 1.85 | 2.36 | 0.63 | 0.62 | 0.42 |
|  | VIT_202s0012g00430 | 0.00 | 0.00 | 0.00 | 0.00 | 0.00 | 0.00 | 0.00 | 0.00 | 0.00 | 0.00 | 0.00 | 0.16 | 0.00 | 0.00 |
|  | VIT_202s0012g00450 | 16.75 | 23.07 | 24.79 | 1.35 | 2.94 | 0.54 | 0.69 | 2.34 | 4.09 | 1.83 | 2.05 | 2.58 | 0.93 | 1.40 |
|  | VIT_211s0118g00390 | 68.18 | 74.79 | 62.42 | 88.06 | 65.33 | 52.46 | 56.49 | 54.32 | 45.02 | 52.36 | 37.52 | 18.23 | 18.56 | 20.42 |
|  | VIT_213s0067g01020 | 2.28 | 19.14 | 16.31 | 1.09 | 1.58 | 0.42 | 1.27 | 3.49 | 0.26 | 0.19 | 1.16 | 0.09 | 0.34 | 0.50 |
|  | **VIT_218s0001g03430** | 56.40 | 19.29 | 44.46 | 15.15 | 10.32 | 17.52 | 13.81 | 19.98 | 35.29 | 25.52 | 36.21 | 53.00 | 52.10 | 53.01 |
|  | **VIT_218s0001g03470** | 7.95 | 0.22 | 0.09 | 0.82 | 0.00 | 1.68 | 0.00 | 0.00 | 6.55 | 0.00 | 4.17 | 1.66 | 0.00 | 2.37 |
|  | **VIT_218s0001g03490** | 0.12 | 0.06 | 0.00 | 0.00 | 0.00 | 0.00 | 0.00 | 0.00 | 0.00 | 0.00 | 0.00 | 0.00 | 0.00 | 0.00 |
|  | VIT_202s0012g00380 | 1.05 | 0.83 | 0.38 | 0.22 | 0.29 | 0.29 | 0.30 | 0.60 | 1.00 | 1.42 | 0.70 | 0.53 | 0.15 | 0.16 |
|  | VIT_202s0012g00390 | 1.23 | 13.88 | 8.08 | 1.49 | 2.69 | 0.66 | 1.33 | 1.33 | 2.09 | 0.51 | 2.94 | 0.76 | 0.00 | 0.50 |
|  | VIT_202s0012g00400 | 6.74 | 49.19 | 39.63 | 5.37 | 12.93 | 2.36 | 3.90 | 8.44 | 4.38 | 2.40 | 12.23 | 2.29 | 0.63 | 1.75 |
|  | VIT_202s0012g00410 | 4.20 | 13.61 | 9.87 | 3.41 | 5.13 | 2.56 | 2.78 | 4.34 | 3.96 | 1.86 | 3.50 | 3.59 | 4.17 | 3.44 |
|  | VIT_203s0017g02350 | 8.24 | 8.45 | 7.12 | 12.78 | 12.19 | 11.32 | 10.44 | 10.86 | 12.49 | 11.50 | 11.94 | 12.19 | 10.86 | 11.14 |
|  | VIT_203s0091g01080 | 1.93 | 0.49 | 0.49 | 0.00 | 0.19 | 0.09 | 0.00 | 0.44 | 0.15 | 0.00 | 0.84 | 0.00 | 0.10 | 0.10 |
|  | VIT_208s0007g00750 | 2.35 | 0.15 | 0.03 | 0.18 | 0.00 | 0.09 | 0.00 | 0.09 | 0.12 | 0.00 | 0.11 | 0.00 | 0.00 | 0.03 |
|  | VIT_208s0105g00380 | 22.90 | 121.58 | 87.81 | 77.91 | 40.15 | 152.02 | 118.24 | 148.04 | 52.61 | 64.15 | 49.56 | 23.78 | 18.49 | 23.46 |
|  | VIT_209s0002g08090 | 26.37 | 21.22 | 17.84 | 12.90 | 11.13 | 10.33 | 8.69 | 9.85 | 8.94 | 7.27 | 7.38 | 4.45 | 4.10 | 3.96 |
|  | VIT_210s0003g02260 | 0.05 | 1.24 | 0.47 | 0.11 | 0.10 | 0.00 | 0.00 | 0.05 | 0.00 | 0.00 | 0.00 | 0.00 | 0.00 | 0.00 |
|  | VIT_211s0118g00360 | 7.40 | 16.64 | 11.26 | 15.59 | 15.75 | 7.70 | 11.98 | 12.19 | 8.76 | 6.98 | 8.10 | 4.31 | 4.31 | 4.05 |
|  | VIT_211s0118g00370 | 0.43 | 0.12 | 0.41 | 0.18 | 0.28 | 0.21 | 0.02 | 0.21 | 0.37 | 0.51 | 0.17 | 0.61 | 0.49 | 0.34 |
|  | VIT_213s0019g02010 | 0.00 | 0.00 | 0.11 | 0.06 | 0.16 | 0.00 | 0.00 | 0.06 | 0.11 | 0.06 | 0.00 | 0.06 | 0.00 | 0.06 |
|  | VIT_216s0022g00420 | 0.05 | 0.05 | 0.00 | 0.00 | 0.05 | 0.00 | 0.00 | 0.00 | 0.00 | 0.00 | 0.00 | 0.00 | 0.00 | 0.00 |
|  | VIT_216s0022g00430 | 0.00 | 0.00 | 0.05 | 0.00 | 0.10 | 0.00 | 0.00 | 0.00 | 0.00 | 0.05 | 0.00 | 0.00 | 0.00 | 0.00 |
|  | **VIT_218s0001g03510** | 0.85 | 0.47 | 0.50 | 0.08 | 0.24 | 0.08 | 0.11 | 0.17 | 0.00 | 0.03 | 0.03 | 0.03 | 0.14 | 0.00 |
| DFR | VIT_203s0038g04230 | 0.00 | 0.08 | 0.00 | 0.00 | 0.00 | 0.00 | 0.00 | 0.00 | 0.00 | 0.00 | 0.00 | 0.00 | 0.00 | 0.00 |
|  | VIT_204s0023g02080 | 0.25 | 0.25 | 0.24 | 0.00 | 0.00 | 0.00 | 0.00 | 0.25 | 0.24 | 0.00 | 0.00 | 0.00 | 0.00 | 0.00 |
|  | VIT_204s0023g02090 | 0.00 | 0.23 | 0.12 | 0.00 | 0.11 | 0.00 | 0.11 | 0.00 | 0.00 | 0.00 | 0.12 | 0.00 | 0.00 | 0.00 |
|  | VIT_213s0047g00700 | 0.55 | 0.00 | 0.00 | 0.30 | 0.00 | 0.17 | 0.25 | 0.29 | 0.09 | 0.00 | 0.00 | 0.00 | 0.00 | 0.11 |
|  | VIT_213s0064g00340 | 0.20 | 0.20 | 0.39 | 0.19 | 0.00 | 0.00 | 0.00 | 0.00 | 0.00 | 0.00 | 0.00 | 0.00 | 0.00 | 0.00 |
|  | VIT_215s0048g00980 | 6.30 | 0.00 | 0.35 | 0.02 | 0.04 | 0.02 | 0.02 | 0.02 | 0.05 | 0.04 | 0.00 | 0.04 | 0.04 | 0.08 |
|  | VIT_215s0048g01000 | 12.60 | 29.04 | 17.94 | 12.05 | 16.90 | 9.33 | 12.20 | 9.61 | 7.28 | 7.56 | 7.12 | 4.29 | 4.35 | 5.99 |
|  | VIT_215s0048g01010 | 27.73 | 31.38 | 14.23 | 12.51 | 10.79 | 6.67 | 8.29 | 7.61 | 10.59 | 5.13 | 8.38 | 4.66 | 2.75 | 4.36 |
|  | VIT_216s0039g02350 | 7.31 | 11.42 | 14.96 | 14.02 | 13.82 | 11.54 | 14.18 | 11.25 | 11.31 | 10.76 | 6.35 | 5.64 | 3.11 | 3.91 |
|  | VIT_218s0001g12790 | 0.00 | 0.00 | 0.00 | 0.08 | 0.00 | 0.08 | 0.00 | 0.00 | 0.00 | 0.00 | 0.00 | 0.00 | 0.00 | 0.00 |
|  | VIT_218s0001g12800 | 125.22 | 65.19 | 112.02 | 97.28 | 74.44 | 46.21 | 45.60 | 51.57 | 46.72 | 35.49 | 39.73 | 104.41 | 86.52 | 70.39 |
|  | VIT_218s0001g12810 | 0.05 | 0.00 | 0.00 | 0.00 | 0.00 | 0.00 | 0.00 | 0.00 | 0.00 | 0.00 | 0.00 | 0.00 | 0.00 | 0.05 |
|  | VIT_219s0014g04980 | 25.23 | 25.04 | 22.30 | 23.70 | 27.66 | 14.25 | 18.74 | 18.99 | 18.79 | 20.21 | 17.16 | 7.78 | 11.98 | 7.81 |
| LDOX | VIT_202s0025g04720 | 180.65 | 43.95 | 72.63 | 255.14 | 87.67 | 152.12 | 110.04 | 76.34 | 180.96 | 127.28 | 123.69 | 127.76 | 123.48 | 85.31 |
|  | VIT_210s0003g02450 | 11.48 | 52.03 | 56.04 | 58.93 | 56.78 | 54.64 | 44.91 | 44.12 | 54.53 | 53.65 | 47.39 | 50.36 | 53.44 | 52.86 |
|  | VIT_210s0003g02510 | 0.47 | 0.83 | 1.07 | 0.44 | 0.42 | 2.01 | 1.17 | 0.79 | 1.13 | 1.24 | 1.34 | 1.99 | 0.38 | 0.98 |
| LAR | VIT_201s0011g02960 | 10.86 | 1.11 | 6.36 | 3.80 | 4.77 | 1.08 | 1.92 | 1.43 | 1.98 | 0.97 | 0.62 | 0.99 | 0.54 | 0.63 |
|  | VIT_217s0000g04150 | 50.02 | 28.72 | 61.08 | 23.12 | 15.81 | 6.34 | 5.93 | 30.58 | 7.48 | 5.12 | 7.42 | 8.95 | 5.68 | 7.63 |
| ANR | VIT_202s0025g01260 | 3.30 | 7.23 | 1.75 | 2.04 | 2.91 | 0.81 | 0.70 | 0.69 | 0.24 | 0.12 | 0.15 | 0.00 | 0.03 | 0.09 |
|  | VIT_215s0046g01150 | 0.00 | 0.00 | 0.00 | 0.18 | 0.37 | 0.33 | 1.08 | 0.65 | 0.00 | 0.69 | 0.19 | 0.34 | 0.24 | 0.18 |
|  | VIT_215s0046g01160 | 0.17 | 0.04 | 0.08 | 0.25 | 0.22 | 0.61 | 0.21 | 0.17 | 0.47 | 0.29 | 0.28 | 0.25 | 0.21 | 0.42 |
|  | VIT_215s0046g01170 | 2.28 | 0.10 | 0.66 | 0.66 | 1.36 | 0.05 | 0.39 | 0.10 | 0.00 | 0.00 | 0.05 | 0.00 | 0.00 | 0.00 |
|  | VIT_200s0361g00040 | 57.20 | 9.36 | 41.75 | 7.10 | 1.93 | 0.36 | 0.33 | 2.15 | 1.63 | 0.67 | 1.26 | 3.00 | 1.35 | 1.27 |
| UFGT | VIT_200s0218g00170 | 16.46 | 31.82 | 37.43 | 14.69 | 13.97 | 11.07 | 8.31 | 13.36 | 20.17 | 14.03 | 18.65 | 13.45 | 10.20 | 9.99 |
|  | VIT_200s0218g00190 | 6.27 | 15.90 | 19.25 | 1.72 | 4.19 | 2.05 | 2.11 | 2.77 | 1.31 | 1.38 | 0.49 | 0.68 | 0.94 | 1.30 |
|  | VIT_206s0009g01960 | 0.05 | 0.00 | 0.09 | 0.05 | 0.00 | 0.00 | 0.00 | 0.00 | 0.05 | 0.00 | 0.00 | 0.00 | 0.00 | 0.05 |
|  | VIT_206s0009g01990 | 0.15 | 0.41 | 0.15 | 0.00 | 0.00 | 0.00 | 0.00 | 0.00 | 0.00 | 0.07 | 0.08 | 0.00 | 0.04 | 0.00 |
|  | VIT_206s0009g02010 | 0.00 | 0.00 | 0.16 | 0.08 | 0.00 | 0.00 | 0.08 | 0.00 | 0.00 | 0.00 | 0.00 | 0.00 | 0.00 | 0.00 |
|  | VIT_211s0052g01580 | 0.96 | 0.11 | 0.15 | 0.04 | 0.00 | 0.00 | 0.00 | 0.11 | 0.00 | 0.00 | 0.04 | 0.00 | 0.00 | 0.15 |
|  | VIT_211s0052g01600 | 2.33 | 1.64 | 0.16 | 2.16 | 0.07 | 2.87 | 0.16 | 0.65 | 4.28 | 0.16 | 3.52 | 2.99 | 0.03 | 3.15 |
|  | VIT_211s0052g01630 | 1.11 | 1.01 | 0.04 | 0.97 | 0.04 | 1.32 | 0.04 | 0.14 | 2.35 | 0.04 | 1.97 | 0.59 | 0.00 | 0.52 |
|  | VIT_216s0039g02230 | 13.23 | 8.79 | 9.30 | 90.57 | 30.30 | 59.99 | 53.53 | 30.63 | 75.39 | 74.01 | 48.08 | 59.11 | 60.21 | 40.21 |
| COMT | VIT_212s0059g01760 | 0.09 | 0.00 | 0.00 | 0.00 | 0.00 | 0.00 | 0.00 | 0.00 | 0.00 | 0.00 | 0.00 | 0.00 | 0.00 | 0.00 |
|  | VIT_212s0059g01770 | 19.52 | 2.32 | 1.55 | 0.69 | 1.60 | 0.28 | 0.38 | 0.19 | 0.10 | 0.09 | 0.00 | 0.00 | 0.10 | 0.20 |
| GT5 | VIT_211s0052g01640 | 1.03 | 1.12 | 1.49 | 1.82 | 1.00 | 1.64 | 1.19 | 2.48 | 2.99 | 3.00 | 2.21 | 2.98 | 3.47 | 3.51 |
| GT6 | VIT_202s0025g02920 | 0.77 | 2.96 | 4.73 | 9.08 | 4.14 | 12.31 | 9.36 | 10.41 | 16.51 | 19.18 | 15.01 | 16.69 | 12.04 | 11.92 |
|  | VIT_208s0007g04520 | 50.35 | 45.59 | 38.39 | 59.22 | 49.88 | 51.30 | 49.48 | 50.84 | 56.63 | 48.64 | 51.23 | 40.77 | 37.09 | 39.26 |
|  | VIT_208s0032g01130 | 0.00 | 0.00 | 0.00 | 0.00 | 0.00 | 0.00 | 0.00 | 0.10 | 0.00 | 0.00 | 0.00 | 0.00 | 0.00 | 0.00 |
|  | VIT_210s0003g04160 | 0.00 | 0.06 | 0.00 | 0.00 | 0.00 | 0.16 | 0.16 | 0.00 | 0.00 | 0.00 | 0.00 | 0.00 | 0.00 | 0.00 |
|  | VIT_213s0074g00220 | 0.00 | 0.17 | 0.16 | 0.00 | 0.00 | 0.00 | 0.00 | 0.00 | 0.00 | 0.00 | 0.00 | 0.00 | 0.00 | 0.00 |
|  | VIT_215s0048g02450 | 15.96 | 0.00 | 0.00 | 0.00 | 0.00 | 0.00 | 0.00 | 0.00 | 0.00 | 0.00 | 0.00 | 0.00 | 0.00 | 0.00 |
|  | VIT_215s0048g02460 | 1.07 | 0.23 | 0.18 | 0.00 | 0.00 | 0.00 | 0.00 | 0.22 | 0.00 | 0.04 | 0.04 | 0.00 | 0.00 | 0.00 |
|  | VIT_215s0048g02480 | 0.75 | 1.10 | 0.41 | 1.58 | 0.26 | 4.29 | 3.11 | 1.79 | 4.95 | 6.47 | 2.25 | 0.20 | 0.48 | 0.89 |
|  | VIT_215s0048g02490 | 0.47 | 2.09 | 0.34 | 1.03 | 0.34 | 3.28 | 2.69 | 1.16 | 4.80 | 3.04 | 3.27 | 0.29 | 0.12 | 1.04 |
|  | VIT_216s0098g00850 | 294.90 | 69.54 | 65.17 | 48.05 | 67.57 | 25.93 | 34.32 | 35.58 | 15.52 | 16.51 | 11.79 | 9.16 | 5.48 | 7.09 |
|  | VIT_218s0001g02610 | 32.27 | 17.21 | 29.44 | 12.05 | 17.75 | 12.69 | 15.51 | 17.08 | 8.61 | 10.59 | 9.21 | 4.69 | 5.26 | 4.77 |
|  | VIT_218s0072g00920 | 12.32 | 1.60 | 2.66 | 0.00 | 0.30 | 0.00 | 0.00 | 1.56 | 0.23 | 0.57 | 0.64 | 0.19 | 0.57 | 0.40 |
|  | VIT_219s0135g00030 | 0.05 | 0.21 | 0.37 | 0.05 | 0.00 | 0.00 | 0.12 | 0.00 | 0.00 | 0.00 | 0.13 | 0.00 | 0.05 | 0.00 |

T1: cluster bagging from 3 WAF until harvest; T2: control group; T8: cluster bagging at E-L 35 stage and bag removal at E-L 36 stage. PAL, phenylalanine ammonia-lyase; C4H, trans-cinnamate 4-monooxygenase; CCR, cinnamoyl-CoA reductase; CAD, cinnamyl-alcohol dehydrogenase; 4CL, 4-coumarate: CoA ligase; CHS: chalcone synthase; CHI, chalcone isomerase; F3H, flavanone 3-hydroxylase; F3'H, flavonoid 3'-hydroxylase; F3'5'H, flavonoid 3',5'-hydroxylase; FLS, flavonol synthase; DFR, dihydroflavonol 4-reductase; LDOX, leucoanthocyanidin dioxygenase; LAR, leucoanthocyanidin reductase; ANR, anthocyanidin reductase; UFGT, UDP-glucose: flavonoid 3-*O*-glucosyltransferase; COMT, caffeic acid 3-*O*-methyltransferase; GT5, uridine diphosphate (UDP)-glucuronic acid:flavonol-3-*O*-glucuronosyltransferase; GT6, bifunctional UDP-glucose/UDP-galactose:flavonol-3-*O*-glucosyltransferase/galactosyltransferase. Red and green boxes represent genes that are significantly up and down-regulated after cluster bagging treatment, respectively.
