# Supplementary material for: Comparative physiological, metabolomic, and transcriptomic analyses reveal developmental stage-dependent effects of cluster bagging on phenolic metabolism in Cabernet Sauvignon grape berries
Source: BMC Plant Biol. 2019 Dec 26;19:583. doi: 10.1186/s12870-019-2186-z (PMC6933938; doi:10.1186/s12870-019-2186-z)
Supplement: Supplementary file 8 — Additional file 8: Table S6. Correlation analysis between the accumulation of phenolic compounds and the transcription of phenolic biosynthesis-related genes among samples. [file 12870_2019_2186_MOESM8_ESM.docx]

**Table S6.** Correlation analysis between the accumulation of phenolic compounds and the transcription of phenolic biosynthesis-related genes among samples.

| Enzyme | Gene ID | Total HCAs | Total HBAs | Total flavan-3-ols | Total anthocynanins | Total flavonols | Total flavonoids | malvidin-3-*O*-glucoside | peonidin-3-*O*-(6-*O*-acetyl)-glucoside | malvidin-3-*O*-(6-*O*-acetyl)-glucoside | myricetin-3-*O*-glucoside | rutin |
| --- | --- | --- | --- | --- | --- | --- | --- | --- | --- | --- | --- | --- |
| PAL | VIT_216s0039g01100 | 0.295 | -0.454 | 0.245 | -0.499 | -0.339 | -0.434 | -0.505 | -0.453 | -0.525 | -0.332 | -0.498 |
|  | VIT_200s2849g00010 | 0.543 | -0.177 | 0.426 | -0.468 | -0.376 | -0.215 | -0.465 | -0.464 | -0.489 | -0.338 | -0.430 |
|  | VIT_208s0040g01710 | 0.597 | 0.281 | 0.604 | -0.280 | -0.154 | 0.228 | -0.267 | -0.302 | -0.289 | -0.217 | -0.206 |
|  | VIT_216s0039g01120 | 0.323 | -0.406 | 0.218 | -0.485 | -0.253 | -0.435 | -0.492 | -0.439 | -0.507 | -0.285 | -0.462 |
|  | VIT_216s0039g01170 | 0.354 | -0.199 | 0.167 | -0.296 | -0.195 | -0.235 | -0.313 | -0.255 | -0.321 | -0.157 | -0.272 |
|  | VIT_206s0004g02620 | 0.360 | 0.094 | 0.463 | -0.195 | -0.220 | 0.192 | -0.184 | -0.194 | -0.214 | -0.182 | -0.187 |
|  | VIT_216s0039g01130 | 0.636 | -0.183 | 0.583 | -0.584 | -0.360 | -0.206 | -0.573 | -0.572 | -0.608 | -0.409 | -0.531 |
|  | VIT_216s0039g01110 | 0.540 | -0.218 | 0.563 | -0.549 | -0.412 | -0.185 | -0.538 | -0.551 | -0.570 | -0.397 | -0.510 |
|  | VIT_213s0019g04460 | 0.486 | 0.522 | 0.427 | 0.029 | 0.033 | 0.470 | 0.039 | 0.001 | 0.021 | 0.023 | 0.087 |
|  | VIT_216s0039g01320 | 0.004 | 0.210 | -0.245 | 0.271 | 0.182 | 0.123 | 0.256 | 0.241 | 0.277 | 0.300 | 0.336 |
|  | VIT_216s0039g01360 | 0.568 | -0.078 | 0.472 | -0.465 | -0.249 | -0.153 | -0.467 | -0.447 | -0.471 | -0.361 | -0.385 |
|  | VIT_211s0016g01520 | 0.418 | -0.183 | 0.367 | -0.349 | -0.330 | -0.115 | -0.333 | -0.385 | -0.358 | -0.265 | -0.326 |
|  | VIT_211s0016g01640 | 0.260 | -0.283 | 0.281 | -0.324 | -0.291 | -0.165 | -0.307 | -0.354 | -0.338 | -0.237 | -0.311 |
|  | VIT_211s0016g01660 | 0.438 | -0.230 | 0.392 | -0.421 | -0.363 | -0.185 | -0.404 | -0.456 | -0.434 | -0.313 | -0.394 |
|  | VIT_216s0039g01300 | 0.216 | 0.098 | 0.238 | -0.024 | -0.057 | 0.203 | -0.022 | -0.051 | -0.037 | 0.015 | -0.005 |
|  | VIT_216s0039g01280 | 0.403 | 0.055 | 0.387 | -0.221 | -0.042 | 0.096 | -0.243 | -0.165 | -0.252 | -0.057 | -0.175 |
|  | VIT_216s0039g01240 | 0.324 | 0.035 | 0.094 | -0.021 | -0.018 | 0.065 | -0.057 | 0.035 | -0.040 | 0.037 | -0.048 |
|  | VIT_211s0016g01535 | -0.097 | 0.190 | -0.044 | 0.096 | -0.086 | 0.073 | 0.116 | 0.049 | 0.154 | -0.046 | 0.150 |
|  | VIT_211s0016g01510 | 0.267 | 0.124 | 0.235 | -0.045 | -0.057 | 0.172 | -0.021 | -0.058 | -0.082 | -0.082 | -0.055 |
|  | VIT_200s2508g00010 | -0.094 | 0.277 | -0.130 | 0.285 | 0.242 | 0.261 | 0.271 | 0.246 | 0.282 | 0.289 | 0.221 |
|  | VIT_218s0111g00036 | -0.062 | 0.346 | -0.312 | 0.487 | 0.504 | 0.364 | 0.475 | 0.440 | 0.472 | 0.532 | 0.473 |
| C4H | VIT_206s0004g08150 | 0.601 | 0.436 | 0.548 | -0.133 | -0.110 | 0.368 | -0.116 | -0.174 | -0.135 | -0.128 | -0.052 |
|  | VIT_211s0065g00350 | **0.737** | 0.271 | 0.563 | -0.297 | -0.229 | 0.158 | -0.277 | -0.345 | -0.311 | -0.222 | -0.232 |
|  | VIT_211s0078g00290 | 0.616 | 0.248 | 0.569 | -0.283 | -0.185 | 0.186 | -0.270 | -0.317 | -0.292 | -0.220 | -0.216 |
| CCR | VIT_206s0004g07130 | 0.573 | -0.202 | **0.802** | **-0.708** | -0.555 | -0.165 | -0.679 | -0.688 | **-0.720** | -0.621 | -0.650 |
|  | VIT_210s0042g00640 | 0.557 | -0.175 | 0.549 | -0.527 | -0.266 | -0.159 | -0.506 | -0.559 | -0.543 | -0.339 | -0.470 |
|  | VIT_209s0070g00240 | 0.648 | 0.264 | 0.671 | -0.335 | -0.187 | 0.221 | -0.320 | -0.359 | -0.346 | -0.260 | -0.254 |
|  | VIT_218s0122g00640 | 0.286 | 0.163 | -0.088 | 0.012 | -0.088 | -0.081 | 0.018 | -0.061 | 0.027 | -0.004 | 0.056 |
|  | **VIT_206s0004g02380** | **0.799** | -0.035 | **0.778** | -0.616 | -0.480 | -0.063 | -0.590 | -0.642 | -0.635 | -0.494 | -0.554 |
|  | VIT_214s0066g01150 | 0.483 | -0.298 | 0.262 | -0.445 | -0.375 | -0.348 | -0.435 | -0.471 | -0.461 | -0.302 | -0.439 |
|  | VIT_216s0039g01670 | 0.353 | 0.511 | 0.067 | 0.292 | 0.302 | 0.474 | 0.255 | 0.294 | 0.292 | 0.285 | 0.311 |
|  | **VIT_206s0004g02370** | **0.778** | -0.147 | **0.754** | -0.640 | -0.512 | -0.120 | -0.618 | -0.663 | -0.665 | -0.490 | -0.599 |
|  | VIT_218s0122g00620 | 0.636 | -0.148 | 0.624 | -0.569 | -0.295 | -0.140 | -0.547 | -0.606 | -0.579 | -0.409 | -0.494 |
|  | VIT_202s0012g01570 | -0.261 | 0.341 | -0.163 | 0.475 | 0.104 | 0.465 | 0.475 | 0.423 | 0.505 | 0.251 | 0.463 |
|  | VIT_212s0035g02070 | 0.260 | -0.076 | 0.149 | -0.135 | 0.129 | -0.015 | -0.131 | -0.127 | -0.155 | 0.020 | -0.124 |
|  | VIT_218s0122g00630 | -0.022 | **-0.781** | 0.120 | -0.549 | -0.317 | -0.621 | -0.581 | -0.421 | -0.579 | -0.384 | -0.638 |
|  | VIT_214s0083g00320 | -0.345 | 0.594 | -0.547 | **0.774** | 0.608 | 0.511 | **0.732** | **0.759** | **0.798** | 0.672 | **0.793** |
|  | **VIT_213s0067g00620** | **0.727** | -0.235 | 0.612 | -0.649 | -0.517 | -0.276 | -0.631 | -0.649 | -0.680 | -0.504 | -0.648 |
|  | VIT_213s0047g00540 | -0.015 | -0.349 | 0.460 | -0.432 | -0.113 | -0.111 | -0.478 | -0.295 | -0.443 | -0.343 | -0.443 |
|  | VIT_213s0101g00240 | -0.017 | -0.393 | 0.362 | -0.340 | -0.118 | -0.090 | -0.396 | -0.160 | -0.393 | -0.227 | -0.442 |
|  | VIT_213s0064g00270 | 0.341 | -0.453 | 0.095 | -0.420 | -0.396 | -0.487 | -0.428 | -0.376 | -0.453 | -0.320 | -0.518 |
|  | VIT_213s0047g00940 | 0.287 | 0.023 | 0.353 | -0.189 | -0.034 | 0.104 | -0.208 | -0.174 | -0.220 | -0.066 | -0.165 |
|  | VIT_213s0064g00280 | 0.524 | -0.102 | 0.407 | -0.369 | -0.309 | -0.099 | -0.339 | -0.428 | -0.379 | -0.268 | -0.333 |
|  | VIT_203s0110g00350 | 0.520 | -0.413 | 0.551 | -0.679 | -0.508 | -0.375 | -0.691 | -0.612 | **-0.708** | -0.549 | **-0.717** |
|  | VIT_213s0064g00290 | 0.429 | -0.415 | 0.394 | -0.566 | -0.482 | -0.384 | -0.564 | -0.575 | -0.593 | -0.426 | -0.579 |
|  | VIT_201s0011g03480 | -0.021 | -0.658 | 0.112 | -0.494 | -0.344 | -0.560 | -0.489 | -0.457 | -0.505 | -0.379 | -0.537 |
|  | VIT_213s0047g00770 | 0.588 | -0.187 | 0.472 | -0.529 | -0.362 | -0.247 | -0.529 | -0.479 | -0.560 | -0.426 | -0.545 |
|  | VIT_215s0107g00210 | 0.452 | -0.395 | 0.172 | -0.430 | -0.321 | -0.416 | -0.414 | -0.446 | -0.470 | -0.256 | -0.494 |
|  | **VIT_203s0038g04220** | **0.711** | 0.048 | **0.754** | -0.508 | -0.386 | 0.062 | -0.484 | -0.540 | -0.526 | -0.406 | -0.441 |
|  | VIT_203s0110g00310 | 0.365 | -0.079 | 0.167 | -0.222 | -0.165 | -0.136 | -0.219 | -0.197 | -0.243 | -0.097 | -0.223 |
|  | VIT_213s0019g01160 | 0.199 | -0.194 | 0.239 | -0.233 | -0.188 | -0.080 | -0.225 | -0.247 | -0.240 | -0.163 | -0.220 |
|  | VIT_213s0067g00530 | 0.061 | -0.404 | 0.165 | -0.356 | -0.145 | -0.312 | -0.354 | -0.325 | -0.355 | -0.247 | -0.327 |
|  | VIT_212s0142g00710 | 0.340 | 0.201 | 0.303 | -0.078 | -0.083 | 0.195 | -0.062 | -0.132 | -0.083 | -0.068 | -0.004 |
|  | VIT_213s0047g00550 | 0.280 | 0.049 | 0.252 | -0.184 | -0.115 | 0.003 | -0.199 | -0.084 | -0.214 | -0.111 | -0.184 |
|  | VIT_212s0142g00510 | 0.529 | 0.271 | 0.598 | -0.264 | -0.143 | 0.244 | -0.251 | -0.285 | -0.272 | -0.206 | -0.187 |
|  | VIT_213s0067g00580 | -0.112 | -0.317 | 0.217 | -0.268 | -0.238 | -0.152 | -0.273 | -0.275 | -0.256 | -0.268 | -0.279 |
|  | VIT_213s0067g00690 | 0.130 | -0.170 | 0.170 | -0.163 | -0.178 | -0.057 | -0.155 | -0.176 | -0.168 | -0.127 | -0.165 |
|  | VIT_213s0101g00250 | 0.322 | -0.226 | 0.038 | -0.240 | -0.300 | -0.300 | -0.230 | -0.270 | -0.261 | -0.173 | -0.264 |
|  | VIT_203s0110g00330 | -0.075 | 0.226 | -0.041 | 0.103 | -0.076 | 0.086 | 0.113 | 0.048 | 0.172 | -0.040 | 0.194 |
|  | VIT_213s0067g00460 | 0.059 | 0.006 | -0.219 | 0.027 | 0.083 | -0.177 | 0.035 | -0.016 | 0.024 | 0.034 | 0.012 |
|  | VIT_213s0067g00590 | 0.487 | 0.079 | 0.048 | -0.095 | -0.176 | -0.091 | -0.073 | -0.136 | -0.106 | -0.103 | -0.127 |
|  | VIT_213s0047g00990 | -0.010 | -0.246 | 0.060 | -0.266 | -0.238 | -0.307 | -0.274 | -0.246 | -0.254 | -0.268 | -0.287 |
|  | VIT_211s0037g01030 | 0.032 | -0.146 | 0.283 | -0.159 | -0.132 | 0.065 | -0.152 | -0.165 | -0.164 | -0.127 | -0.178 |
|  | VIT_213s0101g00330 | 0.028 | -0.198 | 0.042 | -0.200 | -0.200 | -0.236 | -0.193 | -0.190 | -0.214 | -0.171 | -0.241 |
|  | VIT_203s0110g00320 | -0.087 | 0.180 | -0.157 | 0.266 | 0.147 | 0.201 | 0.293 | 0.250 | 0.229 | 0.186 | 0.240 |
|  | VIT_213s0067g00560 | -0.057 | -0.199 | -0.056 | -0.122 | -0.122 | -0.225 | -0.120 | -0.105 | -0.109 | -0.122 | -0.127 |
|  | VIT_213s0047g00760 | -0.140 | -0.212 | 0.181 | -0.158 | -0.170 | -0.039 | -0.181 | -0.086 | -0.171 | -0.146 | -0.179 |
|  | VIT_218s0122g00650 | 0.148 | -0.074 | 0.206 | -0.263 | -0.069 | -0.142 | -0.256 | -0.259 | -0.265 | -0.218 | -0.211 |
|  | VIT_213s0067g00680 | 0.019 | -0.041 | -0.263 | 0.102 | 0.320 | -0.104 | 0.133 | -0.009 | 0.086 | 0.285 | 0.119 |
| CAD | **VIT_200s0615g00030** | **0.742** | -0.154 | 0.534 | -0.599 | -0.498 | -0.287 | -0.578 | -0.617 | -0.620 | -0.470 | -0.566 |
|  | VIT_200s0371g00050 | -0.304 | **-0.787** | 0.149 | -0.623 | -0.426 | -0.698 | -0.657 | -0.456 | -0.628 | -0.607 | -0.673 |
|  | VIT_200s0371g00100 | 0.671 | -0.285 | 0.465 | -0.612 | -0.484 | -0.373 | -0.586 | -0.645 | -0.633 | -0.461 | -0.583 |
|  | VIT_203s0180g00260 | 0.664 | 0.364 | 0.583 | -0.162 | -0.100 | 0.365 | -0.140 | -0.216 | -0.179 | -0.106 | -0.105 |
|  | VIT_202s0025g03100 | 0.408 | **0.775** | -0.104 | 0.525 | 0.270 | 0.602 | 0.531 | 0.399 | 0.539 | 0.454 | 0.544 |
|  | VIT_204s0044g00210 | -0.076 | -0.215 | -0.098 | -0.142 | 0.245 | -0.263 | -0.129 | -0.158 | -0.128 | -0.042 | -0.081 |
|  | VIT_200s0615g00020 | 0.661 | -0.050 | 0.494 | -0.484 | -0.340 | -0.163 | -0.461 | -0.515 | -0.501 | -0.347 | -0.433 |
|  | **VIT_200s0346g00080** | **0.723** | -0.184 | 0.517 | -0.585 | -0.495 | -0.286 | -0.563 | -0.620 | -0.598 | -0.489 | -0.539 |
|  | VIT_204s0044g00190 | 0.625 | 0.289 | 0.674 | -0.309 | -0.168 | 0.259 | -0.296 | -0.333 | -0.319 | -0.242 | -0.225 |
|  | VIT_218s0001g01160 | 0.466 | -0.357 | 0.242 | -0.486 | -0.423 | -0.427 | -0.473 | -0.479 | -0.510 | -0.378 | -0.527 |
|  | VIT_200s0346g00110 | -0.436 | -0.404 | -0.026 | -0.253 | -0.202 | -0.373 | -0.296 | -0.109 | -0.246 | -0.285 | -0.309 |
|  | VIT_200s0218g00010 | 0.548 | -0.050 | 0.365 | -0.401 | -0.310 | -0.182 | -0.384 | -0.417 | -0.414 | -0.309 | -0.360 |
|  | VIT_218s0001g14910 | 0.334 | 0.100 | 0.510 | -0.259 | -0.089 | 0.167 | -0.294 | -0.134 | -0.291 | -0.189 | -0.252 |
|  | VIT_200s1389g00010 | -0.378 | -0.301 | -0.013 | -0.138 | -0.067 | -0.198 | -0.181 | 0.024 | -0.156 | -0.138 | -0.205 |
|  | VIT_207s0129g01030 | -0.134 | 0.068 | -0.104 | 0.197 | 0.061 | 0.157 | 0.219 | 0.059 | 0.231 | 0.064 | 0.220 |
|  | VIT_203s0180g00250 | -0.153 | -0.368 | 0.364 | -0.443 | -0.117 | -0.222 | -0.447 | -0.388 | -0.431 | -0.440 | -0.391 |
|  | VIT_210s0003g04910 | -0.067 | -0.145 | 0.075 | -0.075 | -0.200 | -0.039 | -0.098 | -0.113 | -0.053 | -0.142 | -0.052 |
|  | VIT_215s0048g01710 | 0.188 | -0.221 | -0.045 | -0.152 | 0.020 | -0.241 | -0.154 | -0.159 | -0.168 | -0.069 | -0.170 |
|  | VIT_200s0346g00100 | 0.560 | 0.064 | 0.339 | -0.304 | -0.243 | -0.077 | -0.290 | -0.329 | -0.314 | -0.238 | -0.266 |
|  | VIT_218s0122g00450 | -0.190 | -0.113 | -0.307 | 0.034 | 0.465 | -0.223 | 0.021 | 0.077 | 0.050 | 0.144 | 0.085 |
|  | VIT_200s0174g00270 | 0.323 | 0.134 | 0.153 | -0.065 | 0.052 | 0.073 | -0.060 | -0.088 | -0.072 | 0.011 | -0.002 |
|  | VIT_204s0044g00200 | -0.127 | -0.180 | -0.009 | -0.120 | 0.091 | -0.158 | -0.124 | -0.087 | -0.114 | -0.117 | -0.102 |
|  | VIT_200s0615g00010 | -0.386 | -0.130 | -0.070 | 0.048 | -0.045 | -0.012 | 0.015 | 0.148 | 0.056 | -0.064 | 0.012 |
|  | VIT_200s0371g00060 | 0.413 | 0.188 | 0.387 | -0.163 | -0.068 | 0.170 | -0.155 | -0.176 | -0.168 | -0.127 | -0.111 |
|  | VIT_202s0025g03110 | 0.032 | -0.146 | 0.283 | -0.159 | -0.132 | 0.065 | -0.152 | -0.165 | -0.164 | -0.127 | -0.178 |
|  | VIT_200s0371g00010 | 0.168 | 0.131 | 0.152 | -0.058 | 0.016 | 0.078 | -0.074 | 0.012 | -0.078 | -0.045 | -0.047 |
|  | VIT_200s0371g00040 | 0.146 | 0.337 | -0.098 | 0.281 | 0.461 | 0.308 | 0.238 | 0.402 | 0.247 | 0.443 | 0.294 |
| 4CL | VIT_206s0061g00450 | **0.729** | -0.312 | 0.698 | **-0.731** | -0.499 | -0.295 | **-0.711** | **-0.720** | **-0.766** | -0.548 | **-0.706** |
|  | VIT_202s0025g03660 | 0.583 | -0.292 | 0.368 | -0.522 | -0.402 | -0.346 | -0.501 | -0.555 | -0.540 | -0.372 | -0.501 |
|  | VIT_211s0052g01090 | 0.637 | 0.275 | 0.640 | -0.307 | -0.184 | 0.227 | -0.291 | -0.334 | -0.318 | -0.239 | -0.230 |
|  | VIT_216s0050g00390 | -0.654 | 0.251 | -0.594 | 0.590 | 0.496 | 0.215 | 0.582 | 0.548 | 0.643 | 0.417 | 0.618 |
|  | VIT_216s0039g02040 | **0.701** | 0.259 | 0.599 | -0.287 | -0.259 | 0.204 | -0.259 | -0.339 | -0.301 | -0.240 | -0.219 |
|  | VIT_206s0004g01560 | -0.377 | 0.501 | -0.684 | **0.754** | 0.562 | 0.345 | **0.700** | **0.745** | **0.799** | 0.607 | **0.787** |
|  | VIT_211s0052g01110 | 0.312 | 0.072 | 0.207 | -0.113 | 0.186 | 0.077 | -0.111 | -0.142 | -0.133 | 0.099 | -0.068 |
|  | VIT_208s0007g05050 | 0.022 | -0.038 | 0.017 | -0.008 | -0.023 | 0.006 | 0.007 | -0.030 | -0.038 | 0.033 | -0.105 |
|  | VIT_201s0010g03720 | -0.221 | 0.079 | -0.278 | 0.236 | 0.346 | 0.059 | 0.201 | 0.290 | 0.244 | 0.267 | 0.224 |
|  | VIT_214s0036g00870 | 0.128 | 0.011 | -0.055 | -0.077 | -0.198 | -0.172 | -0.094 | -0.113 | -0.046 | -0.143 | -0.081 |
| CHS | VIT_214s0068g00920 | 0.116 | 0.361 | 0.082 | 0.251 | 0.070 | 0.415 | 0.254 | 0.243 | 0.247 | 0.159 | 0.256 |
|  | VIT_205s0136g00260 | -0.566 | 0.105 | -0.393 | 0.443 | 0.253 | 0.205 | 0.391 | 0.572 | 0.442 | 0.280 | 0.363 |
|  | VIT_203s0038g01460 | -0.098 | 0.528 | -0.425 | 0.669 | 0.369 | 0.476 | 0.685 | 0.587 | 0.693 | 0.424 | 0.680 |
|  | VIT_215s0021g02170 | -0.012 | 0.272 | -0.199 | 0.200 | 0.134 | 0.072 | 0.219 | 0.222 | 0.206 | 0.084 | 0.291 |
|  | VIT_216s0022g01020 | -0.109 | 0.109 | -0.068 | 0.184 | 0.198 | 0.189 | 0.129 | 0.245 | 0.180 | 0.177 | 0.173 |
|  | VIT_216s0022g01190 | -0.105 | -0.147 | -0.151 | -0.032 | -0.065 | -0.198 | -0.035 | -0.013 | -0.025 | -0.038 | -0.054 |
|  | VIT_200s1492g00010 | -0.018 | 0.158 | 0.028 | 0.099 | 0.173 | 0.172 | 0.084 | 0.140 | 0.083 | 0.116 | 0.120 |
|  | VIT_216s0022g01140 | -0.045 | 0.059 | 0.009 | 0.009 | 0.037 | 0.024 | -0.020 | 0.131 | -0.018 | 0.004 | -0.019 |
| CHI | VIT_213s0067g02870 | 0.533 | 0.056 | 0.580 | -0.322 | -0.272 | 0.139 | -0.320 | -0.301 | -0.349 | -0.260 | -0.315 |
|  | VIT_213s0067g03820 | -0.182 | 0.365 | -0.242 | 0.512 | 0.277 | 0.448 | 0.462 | 0.598 | 0.499 | 0.363 | 0.445 |
| F3H | VIT_204s0023g03370 | 0.210 | 0.478 | 0.104 | 0.291 | 0.055 | 0.489 | 0.313 | 0.234 | 0.295 | 0.172 | 0.307 |
|  | VIT_218s0001g14310 | -0.025 | 0.432 | -0.291 | 0.646 | 0.566 | 0.598 | 0.644 | 0.652 | 0.587 | 0.680 | 0.531 |
|  | VIT_200s0521g00030 | 0.575 | 0.033 | 0.580 | -0.403 | -0.318 | 0.030 | -0.385 | -0.423 | -0.418 | -0.321 | -0.349 |
|  | VIT_216s0098g00860 | -0.375 | -0.496 | 0.068 | -0.387 | -0.071 | -0.442 | -0.403 | -0.267 | -0.383 | -0.361 | -0.362 |
|  | VIT_200s0687g00020 | 0.329 | -0.127 | 0.220 | -0.249 | -0.255 | -0.125 | -0.231 | -0.272 | -0.257 | -0.245 | -0.294 |
|  | VIT_213s0047g00210 | 0.396 | -0.554 | 0.432 | **-0.703** | -0.578 | -0.531 | -0.692 | -0.640 | **-0.739** | -0.566 | **-0.750** |
|  | VIT_203s0063g01210 | 0.275 | -0.047 | -0.066 | -0.060 | 0.152 | -0.132 | -0.055 | -0.094 | -0.089 | 0.207 | -0.058 |
|  | VIT_206s0004g00760 | 0.356 | 0.168 | 0.076 | -0.044 | -0.155 | 0.006 | 0.014 | -0.145 | -0.047 | -0.097 | -0.027 |
|  | VIT_203s0063g01280 | 0.189 | -0.193 | -0.004 | -0.225 | 0.017 | -0.295 | -0.244 | -0.152 | -0.223 | -0.172 | -0.212 |
| F3'H | VIT_217s0000g07200 | 0.293 | 0.578 | -0.018 | 0.388 | 0.085 | 0.495 | 0.430 | 0.255 | 0.401 | 0.270 | 0.430 |
|  | VIT_217s0000g07210 | 0.324 | 0.361 | 0.042 | 0.238 | -0.040 | 0.349 | 0.271 | 0.152 | 0.238 | 0.137 | 0.245 |
|  | VIT_202s0025g04864 | -0.697 | -0.352 | -0.348 | 0.106 | 0.023 | -0.209 | 0.091 | 0.166 | 0.120 | -0.007 | 0.031 |
|  | VIT_209s0002g01090 | 0.245 | **-0.705** | 0.582 | **-0.825** | -0.577 | -0.540 | **-0.836** | **-0.727** | **-0.853** | -0.696 | **-0.856** |
|  | VIT_211s0016g01020 | 0.390 | -0.368 | 0.475 | -0.620 | -0.510 | -0.375 | -0.614 | -0.564 | -0.655 | -0.506 | -0.652 |
|  | VIT_202s0025g04866 | -0.296 | -0.231 | 0.019 | 0.006 | -0.157 | 0.013 | 0.032 | -0.002 | 0.001 | -0.099 | -0.025 |
|  | VIT_211s0016g01030 | 0.441 | -0.212 | 0.461 | -0.456 | -0.291 | -0.157 | -0.449 | -0.403 | -0.490 | -0.349 | -0.442 |
|  | VIT_202s0109g00290 | -0.026 | 0.042 | -0.117 | 0.102 | 0.077 | 0.022 | 0.113 | 0.009 | 0.122 | 0.154 | 0.108 |
|  | VIT_211s0016g01050 | 0.374 | -0.317 | 0.139 | -0.428 | -0.437 | -0.455 | -0.411 | -0.445 | -0.441 | -0.346 | -0.454 |
|  | VIT_215s0046g00330 | 0.153 | -0.317 | -0.016 | -0.292 | -0.103 | -0.405 | -0.283 | -0.277 | -0.296 | -0.257 | -0.285 |
|  | VIT_200s0555g00020 | 0.001 | 0.292 | -0.262 | 0.460 | 0.631 | 0.392 | 0.475 | 0.365 | 0.427 | 0.581 | 0.464 |
|  | VIT_215s0048g01500 | 0.328 | -0.187 | 0.097 | -0.272 | -0.264 | -0.279 | -0.259 | -0.290 | -0.280 | -0.214 | -0.294 |
|  | VIT_217s0000g07220 | -0.077 | 0.166 | -0.197 | 0.138 | 0.143 | -0.006 | 0.130 | 0.173 | 0.184 | 0.157 | 0.195 |
|  | VIT_211s0016g00980 | 0.119 | -0.279 | 0.007 | -0.246 | -0.275 | -0.337 | -0.242 | -0.246 | -0.257 | -0.191 | -0.282 |
|  | VIT_202s0012g02380 | -0.168 | 0.062 | -0.080 | 0.072 | 0.160 | 0.027 | 0.032 | 0.179 | 0.061 | 0.137 | 0.083 |
| F3'5'H | VIT_208s0007g05160 | 0.369 | 0.393 | -0.089 | 0.270 | -0.042 | 0.258 | 0.311 | 0.116 | 0.289 | 0.185 | 0.308 |
|  | VIT_206s0009g02840 | -0.293 | 0.510 | -0.330 | 0.605 | 0.265 | 0.480 | 0.573 | 0.641 | 0.629 | 0.377 | 0.601 |
|  | VIT_206s0009g02805 | -0.347 | 0.163 | -0.246 | 0.400 | 0.234 | 0.294 | 0.354 | 0.517 | 0.396 | 0.270 | 0.353 |
|  | VIT_206s0009g02810 | -0.406 | 0.457 | -0.477 | 0.678 | 0.393 | 0.438 | 0.643 | **0.713** | 0.700 | 0.475 | 0.652 |
|  | VIT_206s0009g03000 | -0.315 | 0.572 | -0.519 | **0.787** | 0.485 | 0.547 | **0.748** | **0.780** | **0.813** | 0.573 | **0.795** |
|  | VIT_206s0009g02880 | -0.009 | -0.018 | 0.036 | -0.090 | -0.075 | -0.087 | -0.116 | -0.009 | -0.098 | -0.104 | -0.093 |
|  | VIT_206s0009g03010 | -0.384 | 0.534 | -0.514 | **0.722** | 0.361 | 0.456 | 0.693 | **0.705** | **0.772** | 0.446 | **0.738** |
|  | VIT_206s0009g02860 | 0.324 | 0.081 | 0.525 | -0.251 | -0.275 | 0.176 | -0.263 | -0.302 | -0.240 | -0.275 | -0.205 |
|  | VIT_206s0009g03050 | 0.285 | -0.111 | -0.004 | -0.185 | -0.190 | -0.261 | -0.176 | -0.200 | -0.191 | -0.145 | -0.196 |
|  | VIT_206s0009g02830 | -0.465 | 0.286 | -0.445 | 0.568 | 0.377 | 0.326 | 0.530 | 0.644 | 0.570 | 0.420 | 0.521 |
|  | VIT_206s0009g02910 | -0.020 | 0.184 | -0.320 | 0.266 | 0.339 | 0.055 | 0.267 | 0.201 | 0.252 | 0.415 | 0.297 |
|  | VIT_206s0009g03016 | -0.226 | 0.173 | -0.124 | 0.276 | 0.055 | 0.240 | 0.287 | 0.283 | 0.267 | 0.109 | 0.217 |
|  | VIT_206s0009g03040 | 0.159 | -0.022 | -0.113 | -0.011 | -0.112 | -0.137 | 0.010 | -0.003 | -0.042 | -0.038 | -0.067 |
|  | VIT_206s0009g03140 | 0.592 | 0.299 | 0.645 | -0.278 | -0.141 | 0.273 | -0.264 | -0.303 | -0.287 | -0.216 | -0.196 |
|  | VIT_206s0009g02873 | -0.132 | -0.042 | -0.152 | 0.142 | 0.188 | 0.048 | 0.102 | 0.231 | 0.116 | 0.186 | 0.070 |
|  | VIT_206s0009g02970 | -0.468 | 0.438 | -0.474 | 0.615 | 0.302 | 0.351 | 0.588 | 0.650 | 0.655 | 0.382 | 0.622 |
|  | VIT_206s0009g02920 | -0.086 | 0.021 | 0.060 | 0.051 | 0.065 | 0.132 | 0.040 | 0.129 | 0.025 | 0.052 | -0.015 |
|  | VIT_206s0009g03013 | -0.066 | 0.503 | -0.417 | 0.604 | 0.394 | 0.403 | 0.567 | 0.585 | 0.617 | 0.457 | 0.594 |
| FLS | VIT_202s0012g00320 | 0.653 | -0.123 | 0.413 | -0.499 | -0.496 | -0.278 | -0.455 | -0.543 | -0.502 | -0.463 | -0.453 |
|  | VIT_202s0012g00360 | 0.341 | **-0.710** | 0.662 | **-0.867** | -0.630 | -0.469 | **-0.854** | **-0.814** | **-0.895** | **-0.707** | **-0.892** |
|  | VIT_202s0012g00430 | -0.087 | 0.180 | -0.157 | 0.266 | 0.147 | 0.201 | 0.293 | 0.250 | 0.229 | 0.186 | 0.240 |
|  | VIT_202s0012g00450 | **0.753** | -0.131 | 0.457 | -0.498 | -0.441 | -0.228 | -0.477 | -0.540 | -0.519 | -0.376 | -0.473 |
|  | VIT_211s0118g00390 | 0.236 | -0.681 | 0.580 | **-0.785** | -0.574 | -0.489 | **-0.807** | -0.674 | **-0.818** | -0.654 | **-0.847** |
|  | VIT_213s0067g01020 | 0.500 | -0.344 | 0.183 | -0.494 | -0.437 | -0.498 | -0.474 | -0.519 | -0.508 | -0.383 | -0.498 |
|  | VIT_218s0001g03430 | 0.337 | 0.268 | -0.031 | 0.405 | 0.527 | 0.519 | 0.444 | 0.272 | 0.416 | 0.360 | 0.484 |
|  | **VIT_218s0001g03470** | 0.421 | 0.433 | 0.197 | 0.255 | **0.712** | 0.567 | 0.240 | 0.277 | 0.202 | 0.418 | 0.254 |
|  | VIT_218s0001g03490 | 0.405 | 0.093 | 0.333 | -0.222 | -0.151 | 0.032 | -0.211 | -0.240 | -0.228 | -0.173 | -0.179 |
|  | VIT_202s0012g00380 | 0.258 | 0.185 | 0.089 | 0.036 | 0.063 | 0.141 | -0.017 | 0.027 | 0.055 | 0.033 | 0.043 |
|  | VIT_202s0012g00390 | 0.516 | -0.301 | 0.104 | -0.392 | -0.344 | -0.437 | -0.379 | -0.406 | -0.417 | -0.257 | -0.422 |
|  | VIT_202s0012g00400 | 0.499 | -0.367 | 0.185 | -0.471 | -0.388 | -0.461 | -0.455 | -0.490 | -0.489 | -0.321 | -0.484 |
|  | VIT_202s0012g00410 | 0.568 | -0.220 | 0.184 | -0.406 | -0.360 | -0.375 | -0.378 | -0.436 | -0.423 | -0.305 | -0.405 |
|  | VIT_203s0017g02350 | -0.546 | 0.071 | -0.353 | 0.485 | 0.415 | 0.313 | 0.466 | 0.533 | 0.469 | 0.413 | 0.377 |
|  | VIT_203s0091g01080 | 0.601 | 0.165 | 0.502 | -0.314 | -0.037 | 0.092 | -0.295 | -0.363 | -0.329 | -0.127 | -0.242 |
|  | VIT_208s0007g00750 | 0.571 | 0.254 | 0.602 | -0.283 | -0.138 | 0.225 | -0.271 | -0.291 | -0.298 | -0.207 | -0.215 |
|  | VIT_208s0105g00380 | -0.165 | -0.598 | -0.116 | -0.436 | -0.186 | **-0.700** | -0.475 | -0.253 | -0.458 | -0.390 | -0.483 |
|  | VIT_209s0002g08090 | 0.691 | -0.246 | 0.671 | -0.662 | -0.478 | -0.230 | -0.656 | -0.628 | -0.697 | -0.521 | -0.656 |
|  | VIT_210s0003g02260 | 0.506 | -0.258 | 0.095 | -0.406 | -0.400 | -0.468 | -0.388 | -0.429 | -0.419 | -0.322 | -0.423 |
|  | VIT_211s0118g00360 | 0.103 | **-0.782** | 0.375 | **-0.718** | -0.480 | -0.601 | **-0.725** | -0.632 | **-0.750** | -0.566 | **-0.800** |
|  | VIT_211s0118g00370 | 0.097 | 0.443 | 0.092 | 0.282 | 0.078 | 0.466 | 0.292 | 0.234 | 0.295 | 0.150 | 0.323 |
|  | VIT_213s0019g02010 | -0.077 | -0.116 | 0.130 | -0.036 | 0.003 | 0.084 | -0.054 | -0.039 | -0.032 | 0.022 | -0.043 |
|  | VIT_216s0022g00420 | 0.433 | -0.017 | 0.390 | -0.287 | -0.231 | -0.003 | -0.274 | -0.305 | -0.295 | -0.226 | -0.273 |
|  | VIT_216s0022g00430 | -0.068 | -0.252 | 0.332 | -0.228 | -0.284 | 0.011 | -0.233 | -0.259 | -0.212 | -0.238 | -0.246 |
|  | VIT_218s0001g03510 | 0.679 | -0.083 | **0.708** | -0.594 | -0.463 | -0.103 | -0.567 | -0.623 | -0.597 | -0.506 | -0.524 |
| DFR | VIT_203s0038g04230 | 0.269 | -0.087 | -0.028 | -0.163 | -0.166 | -0.254 | -0.155 | -0.176 | -0.168 | -0.127 | -0.174 |
|  | VIT_204s0023g02080 | 0.231 | -0.044 | 0.157 | -0.204 | 0.016 | -0.106 | -0.212 | -0.182 | -0.211 | -0.099 | -0.144 |
|  | VIT_204s0023g02090 | 0.230 | -0.321 | 0.031 | -0.263 | -0.233 | -0.331 | -0.248 | -0.311 | -0.269 | -0.151 | -0.295 |
|  | VIT_213s0047g00700 | 0.132 | -0.062 | 0.238 | -0.196 | -0.074 | -0.022 | -0.190 | -0.157 | -0.213 | -0.157 | -0.186 |
|  | VIT_213s0064g00340 | 0.131 | -0.195 | 0.248 | -0.300 | -0.280 | -0.166 | -0.287 | -0.311 | -0.314 | -0.243 | -0.303 |
|  | VIT_215s0048g00980 | 0.598 | 0.291 | 0.627 | -0.280 | -0.153 | 0.252 | -0.266 | -0.304 | -0.289 | -0.220 | -0.203 |
|  | VIT_215s0048g01000 | 0.456 | -0.521 | 0.401 | -0.697 | -0.571 | -0.555 | -0.689 | -0.677 | **-0.721** | -0.545 | **-0.722** |
|  | VIT_215s0048g01010 | **0.791** | -0.149 | 0.563 | -0.580 | -0.413 | -0.226 | -0.568 | -0.578 | -0.615 | -0.409 | -0.585 |
|  | VIT_216s0039g02350 | -0.116 | **-0.752** | 0.336 | -0.586 | -0.433 | -0.463 | -0.627 | -0.441 | -0.613 | -0.493 | -0.667 |
|  | VIT_218s0001g12790 | -0.273 | -0.181 | 0.022 | -0.048 | -0.053 | -0.045 | -0.064 | 0.058 | -0.072 | -0.060 | -0.098 |
|  | VIT_218s0001g12800 | 0.442 | 0.126 | 0.465 | -0.188 | -0.292 | 0.197 | -0.135 | -0.256 | -0.197 | -0.227 | -0.170 |
|  | VIT_218s0001g12810 | 0.280 | 0.261 | 0.178 | 0.033 | 0.133 | 0.234 | 0.065 | -0.041 | 0.016 | 0.135 | 0.115 |
|  | VIT_219s0014g04980 | 0.324 | -0.552 | 0.671 | **-0.750** | -0.552 | -0.350 | **-0.770** | -0.685 | **-0.760** | -0.622 | **-0.786** |
| LDOX | VIT_202s0025g04720 | -0.097 | 0.130 | 0.126 | 0.151 | 0.077 | 0.331 | 0.125 | 0.248 | 0.125 | 0.090 | 0.082 |
|  | VIT_210s0003g02450 | -0.449 | -0.286 | -0.429 | 0.228 | 0.022 | -0.132 | 0.215 | 0.252 | 0.224 | 0.167 | 0.153 |
|  | VIT_210s0003g02510 | -0.203 | 0.162 | -0.396 | 0.370 | 0.316 | 0.111 | 0.335 | 0.463 | 0.343 | 0.355 | 0.299 |
| LAR | **VIT_201s0011g02960** | 0.484 | -0.094 | **0.793** | -0.485 | -0.359 | 0.133 | -0.470 | -0.482 | -0.508 | -0.389 | -0.456 |
|  | VIT_217s0000g04150 | 0.562 | -0.268 | 0.614 | -0.621 | -0.400 | -0.227 | -0.597 | -0.628 | -0.643 | -0.498 | -0.581 |
| ANR | VIT_202s0025g01260 | 0.621 | -0.323 | 0.458 | -0.620 | -0.528 | -0.392 | -0.599 | -0.618 | -0.645 | -0.499 | -0.637 |
|  | VIT_215s0046g01150 | -0.542 | -0.281 | -0.179 | -0.066 | -0.110 | -0.275 | -0.083 | -0.044 | -0.024 | -0.184 | -0.067 |
|  | VIT_215s0046g01160 | -0.201 | 0.206 | -0.197 | 0.354 | 0.326 | 0.291 | 0.319 | 0.420 | 0.337 | 0.328 | 0.254 |
|  | **VIT_215s0046g01170** | 0.506 | -0.114 | **0.881** | -0.565 | -0.408 | 0.113 | -0.544 | -0.567 | -0.582 | -0.456 | -0.528 |
|  | VIT_200s0361g00040 | 0.584 | 0.035 | 0.632 | -0.403 | -0.316 | 0.082 | -0.383 | -0.433 | -0.418 | -0.319 | -0.350 |
| UFGT | VIT_200s0218g00170 | 0.510 | -0.237 | 0.205 | -0.291 | -0.257 | -0.195 | -0.285 | -0.312 | -0.322 | -0.171 | -0.323 |
|  | VIT_200s0218g00190 | 0.554 | -0.340 | 0.377 | -0.553 | -0.514 | -0.386 | -0.532 | -0.576 | -0.571 | -0.442 | -0.539 |
|  | VIT_206s0009g01960 | 0.116 | 0.006 | 0.144 | -0.060 | -0.062 | 0.061 | -0.055 | -0.074 | -0.090 | -0.019 | -0.128 |
|  | VIT_206s0009g01990 | 0.573 | -0.049 | 0.177 | -0.317 | -0.339 | -0.264 | -0.304 | -0.376 | -0.314 | -0.249 | -0.316 |
|  | VIT_206s0009g02010 | -0.145 | -0.394 | 0.137 | -0.288 | -0.307 | -0.264 | -0.281 | -0.270 | -0.296 | -0.249 | -0.296 |
|  | VIT_211s0052g01580 | 0.596 | 0.230 | 0.599 | -0.315 | -0.135 | 0.180 | -0.296 | -0.348 | -0.329 | -0.218 | -0.234 |
|  | VIT_211s0052g01600 | 0.164 | 0.507 | -0.305 | 0.599 | **0.702** | 0.534 | 0.586 | 0.642 | 0.508 | **0.757** | 0.488 |
|  | VIT_211s0052g01630 | 0.221 | 0.296 | -0.150 | 0.343 | 0.504 | 0.340 | 0.311 | 0.423 | 0.274 | 0.532 | 0.259 |
|  | VIT_216s0039g02230 | -0.608 | 0.139 | -0.442 | 0.510 | 0.260 | 0.243 | 0.463 | 0.591 | 0.524 | 0.324 | 0.432 |
| COMT | VIT_212s0059g01760 | 0.312 | 0.149 | 0.385 | -0.163 | -0.083 | 0.167 | -0.155 | -0.176 | -0.168 | -0.127 | -0.110 |
|  | VIT_212s0059g01770 | 0.650 | 0.226 | 0.673 | -0.358 | -0.224 | 0.191 | -0.342 | -0.381 | -0.370 | -0.283 | -0.288 |
| GT5 | VIT_211s0052g01640 | -0.277 | 0.491 | -0.489 | 0.634 | 0.472 | 0.376 | 0.633 | 0.577 | 0.661 | 0.476 | 0.653 |
| GT6 | **VIT_202s0025g02920** | -0.574 | 0.356 | **-0.724** | **0.767** | 0.580 | 0.322 | **0.713** | **0.783** | **0.795** | 0.608 | **0.726** |
|  | VIT_208s0007g04520 | -0.126 | -0.313 | 0.145 | -0.122 | 0.076 | -0.007 | -0.161 | 0.016 | -0.175 | -0.051 | -0.235 |
|  | VIT_208s0032g01130 | -0.069 | -0.148 | -0.092 | -0.119 | 0.160 | -0.233 | -0.122 | -0.086 | -0.112 | -0.114 | -0.071 |
|  | VIT_210s0003g04160 | -0.124 | -0.139 | -0.121 | -0.114 | -0.098 | -0.278 | -0.134 | -0.015 | -0.122 | -0.112 | -0.138 |
|  | VIT_213s0074g00220 | 0.197 | -0.184 | 0.049 | -0.233 | -0.245 | -0.276 | -0.222 | -0.252 | -0.240 | -0.183 | -0.242 |
|  | VIT_215s0048g02450 | 0.555 | 0.272 | 0.614 | -0.265 | -0.135 | 0.259 | -0.253 | -0.287 | -0.273 | -0.208 | -0.184 |
|  | VIT_215s0048g02460 | 0.635 | 0.155 | 0.589 | -0.371 | -0.165 | 0.094 | -0.360 | -0.386 | -0.377 | -0.292 | -0.278 |
|  | VIT_215s0048g02480 | -0.352 | -0.013 | -0.244 | 0.210 | 0.171 | 0.043 | 0.123 | 0.308 | 0.222 | 0.138 | 0.177 |
|  | VIT_215s0048g02490 | -0.170 | 0.030 | -0.302 | 0.234 | 0.341 | 0.031 | 0.161 | 0.332 | 0.221 | 0.332 | 0.186 |
|  | VIT_216s0098g00850 | 0.672 | 0.073 | **0.772** | -0.513 | -0.337 | 0.077 | -0.498 | -0.517 | -0.531 | -0.413 | -0.453 |
|  | VIT_218s0001g02610 | 0.459 | -0.360 | **0.726** | **-0.708** | -0.475 | -0.234 | **-0.704** | -0.671 | **-0.724** | -0.574 | -0.663 |
|  | VIT_218s0072g00920 | 0.670 | 0.228 | 0.664 | -0.377 | -0.203 | 0.158 | -0.360 | -0.412 | -0.382 | -0.294 | -0.280 |
|  | VIT_219s0135g00030 | 0.274 | -0.285 | 0.070 | -0.367 | -0.333 | -0.437 | -0.363 | -0.346 | -0.361 | -0.237 | -0.351 |
| HY5 | VIT_204s0008g05210 | 0.585 | 0.104 | 0.429 | -0.278 | -0.058 | 0.064 | -0.286 | -0.212 | -0.339 | -0.098 | -0.290 |
| HYH | VIT_205s0020g01090 | 0.332 | 0.001 | -0.009 | -0.039 | 0.078 | -0.053 | -0.058 | 0.088 | -0.118 | 0.103 | -0.143 |
| MYBF1 | VIT_207s0005g01210 | 0.624 | 0.049 | **0.809** | -0.510 | -0.350 | 0.117 | -0.491 | -0.517 | -0.530 | -0.406 | -0.461 |
| MYB5a | VIT_208s0007g07240 | 0.500 | -0.207 | 0.298 | -0.440 | -0.454 | -0.313 | -0.392 | -0.551 | -0.438 | -0.355 | -0.388 |
| MYB5b | VIT_206s0004g00570 | -0.424 | 0.426 | **-0.851** | **0.777** | 0.546 | 0.205 | **0.760** | **0.752** | **0.803** | 0.628 | **0.773** |
| MYBPA1 | VIT_215s0046g00170 | 0.365 | 0.527 | 0.139 | 0.273 | 0.084 | 0.503 | 0.315 | 0.201 | 0.261 | 0.178 | 0.287 |
| MYBPA2 | VIT_211s0016g01320 | 0.599 | 0.192 | 0.697 | -0.380 | -0.219 | 0.186 | -0.364 | -0.391 | -0.395 | -0.307 | -0.310 |
| MYBPAR | VIT_211s0016g01300 | 0.681 | 0.093 | 0.639 | -0.423 | -0.325 | 0.062 | -0.400 | -0.480 | -0.430 | -0.327 | -0.353 |
| MYBC2-L1 | VIT_201s0011g04760 | 0.500 | 0.534 | -0.030 | 0.296 | 0.038 | 0.359 | 0.320 | 0.200 | 0.304 | 0.211 | 0.303 |
| MYBC2-L2 | VIT_217s0000g02660 | 0.607 | 0.143 | 0.659 | -0.358 | -0.248 | 0.173 | -0.342 | -0.383 | -0.368 | -0.282 | -0.291 |
| MYBC2-L3 | VIT_214s0006g01620 | **0.742** | 0.102 | **0.711** | -0.473 | -0.352 | 0.068 | -0.451 | -0.508 | -0.486 | -0.376 | -0.408 |
| MYBA1 | VIT_202s0033g00410 | -0.363 | -0.046 | -0.214 | 0.300 | 0.309 | 0.202 | 0.253 | 0.433 | 0.246 | 0.336 | 0.168 |
| MYBA2 | VIT_202s0033g00390 | -0.667 | 0.127 | -0.502 | 0.532 | 0.331 | 0.219 | 0.477 | 0.587 | 0.561 | 0.369 | 0.468 |
| MYC1 | VIT_207s0104g00090 | 0.583 | -0.044 | **0.779** | -0.580 | -0.578 | -0.023 | -0.527 | -0.656 | -0.571 | -0.561 | -0.491 |
| WDR1 | VIT_216s0098g00870 | 0.238 | **-0.745** | 0.634 | **-0.885** | -0.613 | -0.569 | **-0.884** | **-0.802** | **-0.908** | **-0.731** | **-0.908** |
| WDR2 | VIT_214s0068g00660 | 0.015 | **0.740** | -0.499 | **0.775** | 0.492 | 0.551 | **0.805** | 0.640 | **0.800** | 0.636 | **0.823** |
| MYCA1 | VIT_215s0046g02560 | 0.527 | 0.208 | 0.621 | -0.311 | -0.117 | 0.208 | -0.296 | -0.320 | -0.324 | -0.227 | -0.225 |
| WRKY26 | VIT_208s0040g03070 | 0.662 | -0.211 | 0.645 | -0.669 | -0.584 | -0.274 | -0.657 | -0.654 | -0.678 | -0.617 | -0.614 |

HCAs, hydroxycinnamic acids; HBAs, hydroxybenzoic acid. PAL, phenylalanine ammonia-lyase; C4H, trans-cinnamate 4-monooxygenase; CCR, cinnamoyl-CoA reductase; CAD, cinnamyl-alcohol dehydrogenase; 4CL, 4-coumarate: CoA ligase; CHS: chalcone synthase; CHI, chalcone isomerase; F3H, flavanone 3-hydroxylase; F3'H, flavonoid 3'-hydroxylase; F3'5'H, flavonoid 3',5'-hydroxylase; FLS, flavonol synthase; DFR, dihydroflavonol 4-reductase; LDOX, leucoanthocyanidin dioxygenase; LAR, leucoanthocyanidin reductase; ANR, anthocyanidin reductase; UFGT, UDP-glucose: flavonoid 3-*O*-glucosyltransferase; COMT, caffeic acid 3-*O*-methyltransferase; GT5, uridine diphosphate (UDP)-glucuronic acid:flavonol-3-*O*-glucuronosyltransferase; GT6, bifunctional UDP-glucose/UDP-galactose:flavonol-3-*O*-glucosyltransferase/galactosyltransferase; HY5, ELONGATED HYPOCOTYL 5; HYH, HY5-homolog.
