# Supplementary material for: Comparative physiological, metabolomic, and transcriptomic analyses reveal developmental stage-dependent effects of cluster bagging on phenolic metabolism in Cabernet Sauvignon grape berries
Source: BMC Plant Biol. 2019 Dec 26;19:583. doi: 10.1186/s12870-019-2186-z (PMC6933938; doi:10.1186/s12870-019-2186-z)
Supplement: Supplementary file 12 — Additional file 12: Table S7. Transcription profile of light and plant hormone signal transduction related genes among samples. [file 12870_2019_2186_MOESM12_ESM.docx]

**Table S7.** Transcription profile of light and plant hormone signal transduction related genes among samples.

| Family | Gene ID | Average expression level (FPKM) | | | | | | | | | | | | | |
| --- | --- | --- | --- | --- | --- | --- | --- | --- | --- | --- | --- | --- | --- | --- | --- |
|  |  | E-L 29 | E-L 31 | | E-L 35 | | E-L 36 | | | E-L 37 | | | E-L 38 | | |
|  |  | T2 | T2 | T1 | T2 | T1 | T2 | T1 | T8 | T2 | T1 | T8 | T2 | T1 | T8 |
| PHYA | VIT_214s0060g00100 | 6.78 | 6.63 | 8.45 | 9.87 | 9.48 | 16.97 | 14.92 | 16.32 | 15.21 | 14.36 | 17.88 | 13.92 | 15.13 | 16.86 |
| PHYB | VIT_212s0057g00980 | 6.88 | 2.44 | 5.26 | 4.49 | 4.34 | 3.28 | 3.77 | 6.83 | 5.67 | 5.38 | 8.21 | 5.95 | 6.27 | 6.42 |
|  | VIT_205s0077g00940 | 3.99 | 5.23 | 6.51 | 5.60 | 4.79 | 7.47 | 7.47 | 9.39 | 5.90 | 6.81 | 8.56 | 7.92 | 8.75 | 10.18 |
| PHYE | VIT_210s0003g02680 | 17.92 | 12.97 | 10.05 | 3.02 | 3.32 | 1.88 | 1.87 | 2.47 | 1.90 | 1.26 | 2.45 | 0.77 | 0.82 | 1.01 |
| PHOT | VIT_203s0038g04210 | 2.65 | 0.70 | 0.18 | 2.03 | 0.37 | 2.59 | 0.60 | 1.00 | 4.80 | 0.86 | 3.80 | 0.98 | 0.34 | 1.56 |
|  | VIT_206s0004g03700 | 20.34 | 10.67 | 9.42 | 5.21 | 3.19 | 9.28 | 7.49 | 12.61 | 8.82 | 7.08 | 11.49 | 7.89 | 6.25 | 9.34 |
| CRY1 | VIT_218s0001g05680 | 22.30 | 26.33 | 20.58 | 29.19 | 25.62 | 25.13 | 26.18 | 25.66 | 20.99 | 19.10 | 20.34 | 13.50 | 12.58 | 15.18 |
| CRY2 | VIT_204s0044g00014 | 0.10 | 0.10 | 0.42 | 0.20 | 0.10 | 0.20 | 0.61 | 0.20 | 0.20 | 0.61 | 0.20 | 0.00 | 0.10 | 0.52 |
|  | VIT_205s0049g00960 | 28.42 | 27.26 | 27.01 | 32.68 | 33.97 | 47.10 | 51.46 | 55.11 | 38.03 | 42.93 | 44.20 | 35.01 | 38.50 | 37.83 |
|  | VIT_200s0160g00140 | 0.00 | 0.00 | 0.29 | 0.13 | 0.00 | 0.14 | 0.00 | 0.00 | 0.00 | 0.00 | 0.00 | 0.00 | 0.00 | 0.00 |
|  | VIT_209s0002g05990 | 5.27 | 4.80 | 1.29 | 2.83 | 0.41 | 5.44 | 0.50 | 1.12 | 3.34 | 0.38 | 3.16 | 2.06 | 0.58 | 2.59 |
| ZTL | VIT_204s0008g02490 | 25.35 | 17.47 | 19.14 | 14.99 | 17.09 | 18.47 | 18.43 | 20.42 | 25.27 | 22.48 | 24.91 | 29.19 | 28.77 | 28.78 |
|  | VIT_211s0052g00720 | 13.24 | 14.31 | 15.24 | 20.41 | 15.51 | 22.95 | 20.45 | 29.47 | 39.45 | 39.22 | 44.71 | 54.94 | 62.14 | 66.74 |
|  | VIT_211s0052g00730 | 29.13 | 29.00 | 29.50 | 39.84 | 36.18 | 50.31 | 45.38 | 51.17 | 77.12 | 66.43 | 69.04 | 104.12 | 106.30 | 107.60 |
| FKF1 | VIT_201s0011g05810 | 3.19 | 7.72 | 5.55 | 3.95 | 1.83 | 8.43 | 5.74 | 7.01 | 21.95 | 13.94 | 19.56 | 25.53 | 21.19 | 25.92 |
| LKP2 | VIT_209s0054g00610 | 2.75 | 4.69 | 4.23 | 4.95 | 3.08 | 6.50 | 4.89 | 6.17 | 14.36 | 10.80 | 12.15 | 16.21 | 16.70 | 16.04 |
| UVR8 | VIT_207s0031g02850 | 3.82 | 2.41 | 2.86 | 3.82 | 3.20 | 4.03 | 3.08 | 3.76 | 5.75 | 4.85 | 4.47 | 6.14 | 4.56 | 5.52 |
|  | VIT_201s0137g00230 | 67.70 | 64.73 | 76.36 | 68.34 | 67.81 | 47.46 | 46.54 | 59.28 | 81.48 | 70.95 | 74.02 | 112.45 | 116.15 | 109.81 |
|  | VIT_212s0057g01540 | 5.47 | 4.16 | 4.72 | 3.90 | 3.32 | 3.12 | 4.20 | 4.43 | 4.39 | 3.49 | 3.81 | 5.22 | 4.26 | 4.17 |
|  | VIT_219s0014g03080 | 15.53 | 16.03 | 16.20 | 12.89 | 14.17 | 13.71 | 16.70 | 15.76 | 10.83 | 11.46 | 11.53 | 9.95 | 9.51 | 10.96 |
|  | VIT_201s0026g01520 | 13.21 | 11.13 | 12.14 | 9.66 | 10.49 | 9.82 | 11.48 | 10.18 | 11.31 | 10.62 | 9.29 | 13.61 | 13.62 | 12.53 |
|  | VIT_207s0031g02560 | 38.34 | 29.86 | 18.93 | 17.89 | 8.18 | 11.41 | 12.61 | 11.17 | 7.19 | 5.01 | 8.32 | 3.73 | 2.45 | 4.64 |
|  | VIT_208s0032g00280 | 11.77 | 7.62 | 10.72 | 12.07 | 10.17 | 11.35 | 11.43 | 15.76 | 17.13 | 17.04 | 18.73 | 19.71 | 22.17 | 22.22 |
|  | VIT_218s0001g13750 | 13.27 | 12.07 | 14.56 | 4.47 | 6.85 | 5.71 | 5.81 | 7.01 | 6.66 | 5.97 | 7.04 | 7.23 | 6.45 | 7.42 |
|  | VIT_201s0026g02170 | 5.86 | 2.15 | 4.52 | 4.90 | 3.80 | 6.04 | 6.60 | 7.24 | 9.57 | 8.94 | 10.08 | 14.05 | 13.54 | 14.09 |
|  | VIT_203s0017g02290 | 0.92 | 0.90 | 0.97 | 0.92 | 0.83 | 0.65 | 0.68 | 1.41 | 1.00 | 0.92 | 1.35 | 1.77 | 1.79 | 2.09 |
|  | VIT_204s0008g03320 | 35.88 | 45.20 | 43.42 | 26.85 | 30.57 | 30.85 | 26.78 | 31.66 | 38.84 | 33.51 | 38.93 | 38.51 | 38.49 | 39.97 |
|  | VIT_204s0079g00290 | 2.03 | 1.21 | 2.89 | 0.31 | 0.77 | 0.24 | 0.25 | 0.50 | 0.82 | 1.07 | 1.05 | 0.90 | 0.97 | 0.66 |
|  | VIT_205s0049g01140 | 0.04 | 0.02 | 0.02 | 0.00 | 0.00 | 0.00 | 0.04 | 0.02 | 0.00 | 0.04 | 0.02 | 0.02 | 0.00 | 0.00 |
|  | VIT_206s0009g03550 | 9.71 | 7.97 | 7.12 | 16.62 | 11.88 | 18.55 | 17.08 | 19.28 | 16.19 | 18.49 | 17.59 | 11.28 | 11.86 | 13.48 |
|  | VIT_208s0007g00930 | 35.22 | 17.69 | 15.70 | 8.73 | 11.69 | 11.66 | 10.07 | 14.37 | 9.59 | 8.31 | 12.45 | 11.10 | 11.26 | 13.84 |
|  | VIT_213s0067g03380 | 13.85 | 7.85 | 8.50 | 14.13 | 11.44 | 13.93 | 15.75 | 14.71 | 11.28 | 12.96 | 12.39 | 11.65 | 12.01 | 12.38 |
|  | VIT_214s0006g03240 | 34.96 | 26.47 | 33.83 | 22.27 | 20.24 | 26.51 | 25.17 | 25.50 | 27.89 | 25.33 | 29.12 | 23.81 | 26.25 | 25.90 |
|  | VIT_214s0083g01020 | 12.87 | 4.42 | 4.22 | 2.10 | 2.26 | 2.27 | 2.26 | 2.35 | 3.35 | 2.32 | 2.40 | 2.65 | 1.98 | 2.33 |
|  | VIT_217s0000g05980 | 0.41 | 0.18 | 0.62 | 0.03 | 0.06 | 0.17 | 0.21 | 0.12 | 0.21 | 0.20 | 0.38 | 0.43 | 0.56 | 0.66 |
|  | VIT_218s0166g00210 | 15.98 | 11.09 | 16.29 | 9.11 | 8.64 | 8.75 | 9.72 | 11.18 | 11.44 | 12.50 | 13.94 | 17.36 | 17.81 | 16.94 |
| COP1 | VIT_210s0523g00030 | 14.26 | 14.03 | 13.10 | 10.75 | 7.36 | 11.19 | 8.01 | 7.66 | 11.12 | 9.52 | 11.74 | 11.83 | 9.22 | 11.87 |
|  | VIT_212s0059g01420 | 6.37 | 8.92 | 9.48 | 5.56 | 5.08 | 6.84 | 7.33 | 8.21 | 10.95 | 9.38 | 10.77 | 15.50 | 16.02 | 16.02 |
|  | VIT_200s0388g00040 | 0.07 | 0.00 | 0.43 | 0.09 | 0.12 | 0.00 | 0.00 | 0.00 | 0.92 | 0.00 | 0.07 | 0.31 | 0.00 | 0.29 |
|  | VIT_200s0574g00030 | 41.18 | 39.13 | 40.22 | 27.17 | 29.63 | 27.02 | 24.43 | 27.00 | 35.68 | 29.95 | 30.70 | 49.01 | 43.69 | 44.69 |
|  | VIT_202s0025g01100 | 4.13 | 5.38 | 7.73 | 3.01 | 3.73 | 3.68 | 4.04 | 3.89 | 5.19 | 3.57 | 4.77 | 3.83 | 4.35 | 3.54 |
|  | VIT_202s0025g01110 | 12.45 | 12.68 | 16.03 | 7.88 | 8.13 | 10.75 | 11.04 | 13.36 | 11.97 | 13.10 | 15.98 | 11.48 | 11.75 | 13.63 |
|  | VIT_210s0523g00040 | 4.89 | 7.21 | 6.04 | 6.64 | 7.61 | 8.11 | 8.60 | 7.11 | 11.03 | 12.08 | 12.54 | 17.56 | 14.18 | 14.68 |
|  | VIT_216s0050g00020 | 0.81 | 0.14 | 0.06 | 0.08 | 0.00 | 0.19 | 0.00 | 0.02 | 0.75 | 0.02 | 0.42 | 0.09 | 0.00 | 0.27 |
|  | VIT_217s0000g08690 | 1.76 | 3.22 | 2.08 | 2.29 | 3.02 | 2.03 | 2.57 | 2.68 | 3.76 | 2.38 | 2.97 | 1.65 | 1.93 | 2.15 |
|  | VIT_219s0014g02650 | 17.00 | 18.68 | 18.59 | 21.28 | 23.21 | 24.85 | 23.32 | 23.41 | 28.66 | 29.63 | 27.38 | 29.27 | 28.41 | 28.15 |
|  | VIT_219s0014g02660 | 26.20 | 24.28 | 22.56 | 21.55 | 16.08 | 26.28 | 21.21 | 23.53 | 32.28 | 25.48 | 33.74 | 46.24 | 45.58 | 48.48 |
| HY5/HYH | VIT_204s0008g05210 | 34.52 | 18.32 | 16.12 | 13.95 | 6.61 | 21.63 | 11.71 | 10.48 | 14.46 | 7.97 | 13.90 | 9.54 | 2.59 | 12.65 |
|  | VIT_205s0020g01090 | 6.37 | 10.97 | 1.29 | 9.00 | 0.12 | 10.62 | 0.63 | 1.76 | 6.53 | 0.32 | 6.08 | 3.03 | 0.00 | 2.32 |
|  | VIT_208s0040g00870 | 89.13 | 64.20 | 96.54 | 56.52 | 64.13 | 64.51 | 58.41 | 67.46 | 81.30 | 78.94 | 91.71 | 123.47 | 123.51 | 123.16 |
| SPA1 | VIT_212s0028g03570 | 3.07 | 2.49 | 2.81 | 1.64 | 0.83 | 1.31 | 0.69 | 2.29 | 0.58 | 0.61 | 1.98 | 0.59 | 0.52 | 1.04 |
|  | VIT_215s0046g01310 | 8.67 | 9.48 | 9.93 | 7.49 | 5.96 | 9.82 | 6.93 | 7.12 | 7.82 | 6.65 | 8.74 | 4.71 | 4.06 | 5.28 |
| BES1 | VIT_219s0014g00880 | 0.17 | 0.17 | 0.00 | 0.52 | 0.00 | 0.08 | 0.16 | 0.00 | 0.17 | 0.00 | 0.26 | 0.08 | 0.00 | 0.00 |
|  | VIT_219s0014g00870 | 8.33 | 11.37 | 10.91 | 5.75 | 5.94 | 7.18 | 7.87 | 9.46 | 8.25 | 9.00 | 8.23 | 6.77 | 6.56 | 6.77 |
| CCA1 | VIT_204s0079g00410 | 31.91 | 43.14 | 35.72 | 59.58 | 59.17 | 63.17 | 68.38 | 67.79 | 66.19 | 68.85 | 64.53 | 42.53 | 42.33 | 48.00 |
|  | VIT_215s0046g02260 | 31.05 | 27.44 | 27.87 | 24.73 | 21.27 | 32.40 | 28.14 | 33.39 | 46.06 | 37.90 | 44.59 | 77.64 | 77.24 | 81.03 |
|  | VIT_216s0050g01180 | 16.60 | 15.66 | 15.59 | 16.43 | 15.11 | 12.30 | 13.83 | 14.55 | 13.41 | 12.51 | 13.39 | 15.82 | 17.84 | 16.53 |
| CK2α | VIT_207s0129g00270 | 51.10 | 52.78 | 51.98 | 40.15 | 44.92 | 44.47 | 44.15 | 49.38 | 63.20 | 55.44 | 56.27 | 64.90 | 62.81 | 66.99 |
|  | VIT_218s0001g09320 | 26.93 | 35.62 | 27.30 | 25.02 | 24.70 | 18.84 | 20.25 | 19.92 | 18.67 | 19.87 | 18.97 | 22.31 | 23.57 | 27.22 |
|  | VIT_207s0129g00410 | 85.23 | 92.12 | 84.02 | 80.50 | 82.64 | 65.57 | 65.97 | 74.32 | 58.41 | 59.58 | 58.79 | 55.31 | 55.75 | 52.41 |
| CK2β | VIT_216s0050g02050 | 0.00 | 0.00 | 0.05 | 0.00 | 0.00 | 0.00 | 0.00 | 0.00 | 0.00 | 0.00 | 0.05 | 0.00 | 0.00 | 0.00 |
|  | VIT_215s0021g01020 | 21.08 | 28.07 | 30.48 | 20.47 | 21.74 | 25.42 | 24.84 | 26.05 | 31.18 | 30.94 | 29.88 | 32.25 | 34.17 | 31.82 |
|  | VIT_202s0012g00090 | 12.77 | 24.57 | 20.86 | 38.66 | 33.95 | 64.57 | 63.02 | 60.10 | 40.25 | 58.92 | 51.08 | 19.52 | 21.52 | 24.32 |
|  | VIT_215s0021g01010 | 8.84 | 7.96 | 10.45 | 6.86 | 6.72 | 7.78 | 7.08 | 9.66 | 9.53 | 8.87 | 11.02 | 12.27 | 10.78 | 12.30 |
|  | VIT_202s0012g00080 | 56.84 | 94.55 | 87.98 | 46.94 | 55.17 | 58.08 | 57.11 | 57.09 | 66.56 | 60.39 | 58.21 | 66.56 | 66.50 | 66.82 |
|  | VIT_218s0001g04620 | 0.00 | 0.10 | 0.00 | 0.00 | 0.00 | 0.00 | 0.00 | 0.00 | 0.00 | 0.00 | 0.00 | 0.00 | 0.00 | 0.00 |
|  | VIT_218s0001g03290 | 7.97 | 7.71 | 8.15 | 10.61 | 8.62 | 10.48 | 9.05 | 10.43 | 14.02 | 13.70 | 11.76 | 16.05 | 17.86 | 15.82 |
| CO/COL | VIT_212s0134g00400 | 10.35 | 4.84 | 3.46 | 2.70 | 1.68 | 2.65 | 2.40 | 3.39 | 3.32 | 1.44 | 3.26 | 3.36 | 1.53 | 2.95 |
|  | VIT_201s0011g04240 | 5.85 | 10.47 | 5.03 | 32.99 | 37.73 | 20.42 | 24.77 | 24.31 | 14.10 | 18.41 | 15.84 | 7.55 | 8.68 | 7.10 |
|  | VIT_200s0203g00210 | 6.52 | 4.17 | 3.75 | 7.62 | 4.06 | 9.79 | 8.62 | 6.46 | 18.09 | 17.09 | 12.29 | 25.21 | 28.43 | 26.46 |
|  | VIT_204s0023g03030 | 44.22 | 51.17 | 33.67 | 18.52 | 19.06 | 19.47 | 16.16 | 19.29 | 13.48 | 12.04 | 14.67 | 7.95 | 8.54 | 10.00 |
|  | VIT_201s0011g03520 | 18.23 | 9.22 | 4.24 | 3.76 | 2.79 | 1.54 | 2.90 | 6.62 | 5.11 | 4.90 | 6.68 | 5.09 | 3.72 | 3.51 |
|  | VIT_212s0057g01350 | 4.51 | 14.93 | 11.32 | 2.84 | 2.55 | 4.80 | 4.57 | 4.65 | 11.53 | 10.61 | 10.52 | 7.66 | 7.69 | 7.35 |
|  | VIT_203s0038g00340 | 0.48 | 2.02 | 0.89 | 0.21 | 0.39 | 0.16 | 0.12 | 0.12 | 0.58 | 0.20 | 0.25 | 0.64 | 0.49 | 0.46 |
|  | VIT_200s0194g00070 | 2.47 | 5.60 | 4.11 | 2.82 | 1.80 | 4.98 | 3.61 | 4.56 | 8.50 | 4.95 | 8.79 | 4.74 | 3.72 | 4.50 |
|  | VIT_205s0102g00750 | 27.09 | 38.48 | 20.13 | 9.43 | 8.34 | 10.09 | 7.66 | 9.46 | 12.13 | 4.15 | 11.28 | 6.28 | 1.95 | 5.92 |
|  | VIT_211s0052g01800 | 41.36 | 75.74 | 49.17 | 40.81 | 41.38 | 38.87 | 39.94 | 37.29 | 27.05 | 22.07 | 29.75 | 22.12 | 16.22 | 18.35 |
|  | VIT_219s0014g03960 | 38.50 | 46.39 | 43.89 | 14.00 | 16.73 | 10.53 | 10.48 | 12.36 | 9.97 | 10.92 | 13.64 | 5.58 | 4.89 | 5.77 |
|  | VIT_214s0219g00220 | 15.16 | 10.69 | 4.91 | 6.47 | 6.52 | 5.76 | 4.76 | 4.25 | 10.82 | 6.03 | 7.06 | 4.50 | 3.27 | 4.54 |
|  | VIT_219s0014g05120 | 0.42 | 0.00 | 0.00 | 0.06 | 0.00 | 0.10 | 0.00 | 0.00 | 0.11 | 0.05 | 0.11 | 1.32 | 1.01 | 0.72 |
|  | VIT_203s0038g00690 | 42.73 | 32.25 | 13.49 | 16.97 | 13.75 | 15.78 | 13.61 | 16.11 | 10.55 | 9.18 | 10.34 | 8.91 | 7.02 | 6.60 |
|  | VIT_207s0104g01360 | 27.48 | 36.62 | 28.73 | 34.37 | 33.90 | 28.71 | 29.04 | 27.97 | 32.69 | 34.22 | 25.39 | 23.97 | 25.17 | 25.58 |
|  | VIT_214s0068g01380 | 6.73 | 5.74 | 5.76 | 0.61 | 1.24 | 0.87 | 1.02 | 2.03 | 1.65 | 1.57 | 3.43 | 1.43 | 1.13 | 1.78 |
|  | VIT_204s0008g07340 | 56.19 | 51.23 | 41.54 | 16.96 | 18.18 | 14.39 | 12.95 | 15.87 | 18.07 | 10.84 | 18.45 | 16.29 | 10.34 | 14.38 |
|  | VIT_212s0059g02510 | 2.88 | 13.28 | 4.59 | 18.03 | 10.54 | 15.02 | 11.21 | 8.36 | 38.05 | 24.18 | 22.77 | 32.13 | 22.13 | 28.87 |
|  | VIT_214s0083g00640 | 26.89 | 22.10 | 11.30 | 6.67 | 6.75 | 7.34 | 5.54 | 6.52 | 7.79 | 6.92 | 6.39 | 4.81 | 5.41 | 5.66 |
|  | VIT_218s0001g13520 | 0.68 | 1.11 | 1.93 | 0.30 | 0.46 | 0.09 | 0.31 | 1.87 | 0.51 | 0.47 | 1.87 | 0.54 | 0.52 | 0.89 |
|  | VIT_214s0083g00650 | 11.71 | 9.79 | 5.27 | 2.60 | 3.07 | 2.66 | 2.69 | 3.37 | 3.72 | 3.53 | 3.13 | 2.46 | 2.34 | 2.61 |
|  | VIT_200s0347g00030 | 4.49 | 10.85 | 4.59 | 12.87 | 8.01 | 19.39 | 9.76 | 15.51 | 45.75 | 30.36 | 45.87 | 54.70 | 41.35 | 43.86 |
|  | VIT_217s0000g03740 | 1.16 | 0.23 | 0.63 | 0.00 | 0.07 | 0.00 | 0.08 | 0.00 | 0.00 | 0.08 | 0.00 | 0.00 | 0.00 | 0.00 |
|  | VIT_212s0059g02500 | 0.28 | 0.14 | 0.14 | 0.14 | 0.04 | 0.37 | 0.14 | 0.19 | 0.77 | 0.33 | 0.19 | 0.43 | 0.23 | 0.10 |
|  | VIT_218s0089g01280 | 0.00 | 0.10 | 0.31 | 0.00 | 0.00 | 0.00 | 0.10 | 0.00 | 0.10 | 0.00 | 0.22 | 0.00 | 0.00 | 0.00 |
|  | VIT_209s0054g00530 | 24.09 | 16.38 | 12.26 | 10.70 | 14.60 | 11.31 | 10.59 | 11.08 | 11.00 | 9.37 | 13.68 | 6.30 | 4.97 | 7.58 |
|  | VIT_219s0014g00350 | 9.70 | 8.54 | 9.34 | 6.10 | 6.11 | 7.71 | 7.43 | 9.11 | 12.26 | 9.68 | 11.26 | 15.21 | 14.83 | 14.88 |
| CUL1 | VIT_205s0020g01330 | 1.12 | 0.80 | 0.16 | 0.49 | 0.63 | 0.00 | 0.00 | 0.15 | 0.63 | 0.00 | 0.16 | 0.00 | 0.00 | 0.00 |
|  | VIT_200s0619g00010 | 0.00 | 0.13 | 0.00 | 0.00 | 0.00 | 0.00 | 0.00 | 0.00 | 0.00 | 0.00 | 0.00 | 0.00 | 0.00 | 0.00 |
|  | VIT_205s0020g01340 | 1.32 | 0.94 | 0.67 | 0.93 | 0.38 | 0.00 | 0.13 | 0.13 | 0.39 | 0.13 | 0.53 | 0.39 | 0.40 | 0.55 |
|  | VIT_218s0122g00200 | 0.52 | 0.22 | 0.80 | 0.23 | 0.13 | 0.38 | 0.38 | 0.07 | 0.18 | 0.07 | 0.22 | 0.15 | 0.22 | 0.30 |
| CUL3 | VIT_214s0108g00490 | 32.71 | 42.13 | 39.40 | 58.24 | 61.90 | 46.93 | 53.63 | 53.39 | 46.26 | 47.61 | 53.61 | 61.96 | 67.30 | 65.60 |
|  | VIT_201s0026g02230 | 17.78 | 19.81 | 22.26 | 17.08 | 17.96 | 21.20 | 22.30 | 23.10 | 24.63 | 27.57 | 26.24 | 30.30 | 30.35 | 30.73 |
|  | VIT_204s0069g00110 | 0.00 | 0.00 | 0.00 | 0.00 | 0.00 | 0.00 | 0.00 | 0.00 | 0.15 | 0.00 | 0.15 | 0.00 | 0.00 | 0.00 |
|  | VIT_204s0069g00120 | 0.00 | 0.00 | 0.25 | 0.00 | 0.24 | 0.00 | 0.00 | 0.00 | 0.26 | 0.24 | 0.00 | 0.00 | 0.00 | 0.00 |
|  | VIT_217s0053g00583 | 0.15 | 0.00 | 0.28 | 0.15 | 0.00 | 0.41 | 0.13 | 0.00 | 0.15 | 0.14 | 0.57 | 0.57 | 0.57 | 1.59 |
| CUL4 | VIT_210s0003g03710 | 33.70 | 27.86 | 33.55 | 36.37 | 31.07 | 36.66 | 40.07 | 45.24 | 43.70 | 51.65 | 55.01 | 56.09 | 62.46 | 58.63 |
| DDB1 | VIT_205s0020g01320 | 72.60 | 49.84 | 63.03 | 49.95 | 49.79 | 49.42 | 50.05 | 56.23 | 63.17 | 55.60 | 67.17 | 78.83 | 78.45 | 78.82 |
|  | VIT_207s0104g01590 | 15.90 | 16.94 | 18.69 | 15.23 | 15.01 | 19.63 | 18.75 | 21.75 | 34.17 | 31.51 | 30.40 | 46.85 | 45.81 | 45.10 |
|  | VIT_208s0032g00250 | 0.76 | 0.38 | 1.14 | 0.36 | 0.00 | 0.73 | 0.36 | 0.00 | 0.00 | 0.38 | 0.00 | 0.74 | 0.00 | 0.79 |
| DET1 | VIT_216s0050g02430 | 2.61 | 2.23 | 3.91 | 0.54 | 0.88 | 0.34 | 0.64 | 1.76 | 1.56 | 1.45 | 1.35 | 1.09 | 1.18 | 0.99 |
| EEP1/PKL | VIT_204s0008g05880 | 14.59 | 6.82 | 11.79 | 9.36 | 8.18 | 9.19 | 9.37 | 11.45 | 11.28 | 11.43 | 15.34 | 9.36 | 10.40 | 10.31 |
| EEP2/SEU | VIT_204s0069g00360 | 5.04 | 2.30 | 6.27 | 3.13 | 3.85 | 6.53 | 6.06 | 3.75 | 7.54 | 8.10 | 3.92 | 3.88 | 5.57 | 4.54 |
| ELF3 | VIT_209s0002g02680 | 6.57 | 7.12 | 6.92 | 3.64 | 3.19 | 5.37 | 4.74 | 8.42 | 11.28 | 7.76 | 13.24 | 10.94 | 7.55 | 10.47 |
| FAR1 | VIT_203s0038g00220 | 10.28 | 3.87 | 4.62 | 12.50 | 11.91 | 11.84 | 16.60 | 14.29 | 18.46 | 22.54 | 17.73 | 5.99 | 7.28 | 4.78 |
|  | VIT_203s0063g00250 | 0.00 | 0.00 | 0.00 | 0.00 | 0.02 | 0.00 | 0.00 | 0.00 | 0.02 | 0.02 | 0.00 | 0.02 | 0.00 | 0.02 |
|  | VIT_204s0008g06370 | 0.00 | 0.06 | 0.94 | 0.00 | 0.00 | 0.00 | 0.00 | 0.00 | 0.00 | 0.00 | 0.00 | 0.00 | 0.00 | 0.00 |
|  | VIT_204s0008g06410 | 23.32 | 16.27 | 15.41 | 6.98 | 10.13 | 11.06 | 10.05 | 11.29 | 15.87 | 12.25 | 12.93 | 16.44 | 15.65 | 14.61 |
|  | VIT_205s0102g00780 | 1.54 | 1.50 | 0.72 | 0.24 | 0.10 | 0.05 | 0.19 | 0.05 | 0.05 | 0.00 | 0.05 | 0.39 | 0.25 | 0.10 |
|  | VIT_206s0009g02452 | 0.00 | 0.00 | 0.09 | 0.00 | 0.04 | 0.00 | 0.00 | 0.09 | 0.10 | 0.05 | 0.23 | 0.14 | 0.00 | 0.14 |
|  | VIT_208s0007g06900 | 2.35 | 0.34 | 0.42 | 0.00 | 0.16 | 0.00 | 0.00 | 0.09 | 0.00 | 0.00 | 0.00 | 0.00 | 0.00 | 0.00 |
|  | VIT_209s0002g08220 | 0.28 | 0.10 | 0.09 | 0.10 | 0.09 | 0.00 | 0.09 | 0.00 | 0.00 | 0.00 | 0.10 | 0.09 | 0.00 | 0.10 |
| FHY | VIT_208s0007g00610 | 18.39 | 20.53 | 16.59 | 13.14 | 13.00 | 10.18 | 11.83 | 12.50 | 7.00 | 7.44 | 6.38 | 2.72 | 2.74 | 3.85 |
|  | VIT_203s0038g00130 | 271.11 | 787.48 | 456.87 | 294.27 | 475.61 | 149.91 | 183.35 | 187.91 | 280.98 | 202.53 | 324.76 | 338.15 | 284.58 | 318.55 |
| FHL | VIT_208s0007g00800 | 8.83 | 8.28 | 6.99 | 5.52 | 5.84 | 5.40 | 4.01 | 4.60 | 7.01 | 5.57 | 5.68 | 8.93 | 7.64 | 7.99 |
| GI | VIT_204s0008g00660 | 7.49 | 8.88 | 9.10 | 11.52 | 10.05 | 14.72 | 13.64 | 14.28 | 26.94 | 25.41 | 27.33 | 33.53 | 34.51 | 32.57 |
| LAF1 | VIT_217s0000g03560 | 1.41 | 2.72 | 1.80 | 0.08 | 0.15 | 0.00 | 0.00 | 0.98 | 0.16 | 0.08 | 0.36 | 0.08 | 0.12 | 0.16 |
|  | VIT_202s0025g02210 | 1.98 | 0.97 | 1.41 | 0.10 | 0.00 | 0.00 | 0.00 | 0.00 | 0.00 | 0.00 | 0.00 | 0.00 | 0.00 | 0.00 |
|  | VIT_215s0048g02120 | 0.14 | 0.28 | 0.36 | 0.64 | 0.34 | 1.40 | 1.00 | 1.73 | 4.71 | 4.41 | 3.58 | 8.16 | 7.03 | 7.35 |
|  | VIT_212s0035g02160 | 1.12 | 3.06 | 2.28 | 0.48 | 0.48 | 1.34 | 1.25 | 2.09 | 2.84 | 2.73 | 2.70 | 4.49 | 4.86 | 3.75 |
| LHY | VIT_215s0048g02410 | 235.49 | 229.66 | 175.57 | 150.98 | 129.10 | 127.47 | 115.90 | 130.04 | 89.85 | 91.16 | 107.16 | 115.33 | 112.12 | 127.23 |
|  | VIT_208s0040g03220 | 43.44 | 51.36 | 33.16 | 33.22 | 31.26 | 29.40 | 26.28 | 22.57 | 26.06 | 23.07 | 22.42 | 29.33 | 29.37 | 31.34 |
|  | VIT_215s0048g02400 | 112.51 | 110.46 | 84.06 | 75.01 | 63.44 | 62.75 | 58.85 | 65.46 | 46.78 | 48.03 | 56.92 | 59.18 | 57.51 | 65.27 |
| PAP1 | VIT_211s0016g01320 | 2.87 | 0.23 | 0.14 | 0.50 | 0.13 | 0.04 | 0.00 | 0.22 | 0.00 | 0.00 | 0.00 | 0.04 | 0.00 | 0.00 |
|  | VIT_206s0004g06280 | 0.09 | 0.00 | 0.54 | 0.00 | 0.00 | 0.00 | 0.00 | 0.00 | 0.00 | 0.00 | 0.05 | 0.00 | 0.00 | 0.05 |
| PKS1 | VIT_201s0026g02330 | 2.98 | 3.90 | 3.02 | 0.85 | 2.26 | 0.11 | 0.46 | 0.53 | 0.04 | 0.11 | 0.25 | 0.04 | 0.00 | 0.07 |
|  | VIT_217s0000g06040 | 0.68 | 0.00 | 0.10 | 0.05 | 0.09 | 0.10 | 0.10 | 0.05 | 0.50 | 0.05 | 0.10 | 0.75 | 0.50 | 0.46 |
| PKS4 | VIT_208s0056g00720 | 0.00 | 0.00 | 0.00 | 0.00 | 0.04 | 0.00 | 0.04 | 0.00 | 0.04 | 0.04 | 0.04 | 0.08 | 0.04 | 0.04 |
| PPR5 | VIT_205s0020g03380 | 6.28 | 2.53 | 4.63 | 5.24 | 3.65 | 2.52 | 2.60 | 2.90 | 2.68 | 1.82 | 2.10 | 1.36 | 0.98 | 1.39 |
|  | VIT_206s0004g07210 | 0.21 | 0.21 | 0.04 | 0.00 | 0.00 | 0.00 | 0.00 | 0.00 | 0.00 | 0.04 | 0.04 | 0.00 | 0.00 | 0.00 |
|  | VIT_216s0098g00900 | 9.97 | 23.80 | 22.10 | 8.68 | 7.80 | 19.37 | 10.72 | 15.75 | 36.09 | 30.30 | 40.79 | 53.76 | 45.61 | 53.64 |
| PPR7 | VIT_215s0048g02540 | 18.06 | 34.95 | 32.69 | 13.77 | 11.25 | 20.81 | 13.51 | 17.84 | 25.42 | 16.68 | 29.49 | 35.49 | 24.43 | 38.88 |
|  | VIT_208s0040g03120 | 0.65 | 0.00 | 0.00 | 0.00 | 0.06 | 0.13 | 0.00 | 0.00 | 0.06 | 0.06 | 0.00 | 0.00 | 0.00 | 0.00 |
|  | VIT_213s0067g03390 | 7.35 | 5.36 | 12.73 | 0.75 | 1.83 | 2.08 | 1.48 | 6.96 | 2.10 | 1.41 | 7.63 | 2.20 | 2.71 | 4.22 |
|  | VIT_203s0038g00480 | 37.96 | 47.41 | 35.19 | 38.20 | 45.07 | 29.40 | 30.64 | 31.76 | 27.06 | 22.79 | 29.57 | 27.70 | 24.82 | 26.43 |
|  | VIT_208s0040g03075 | 0.78 | 0.52 | 0.28 | 0.22 | 0.24 | 0.12 | 0.26 | 0.26 | 0.21 | 0.26 | 0.34 | 0.43 | 0.29 | 0.18 |
| PPR9 | VIT_201s0146g00360 | 5.75 | 15.95 | 16.68 | 1.33 | 1.74 | 1.06 | 1.13 | 3.34 | 1.09 | 1.11 | 2.14 | 0.81 | 0.67 | 0.70 |
| RCD1 | VIT_218s0001g08290 | 38.82 | 39.67 | 31.73 | 75.78 | 70.46 | 84.26 | 80.53 | 73.21 | 79.02 | 71.20 | 73.63 | 122.92 | 120.34 | 126.74 |
| RBX1 | VIT_200s0396g00050 | 0.87 | 1.39 | 1.23 | 1.17 | 1.41 | 1.19 | 1.33 | 0.78 | 1.59 | 1.30 | 1.73 | 2.19 | 1.76 | 2.34 |
|  | VIT_200s0629g00020 | 2.35 | 6.12 | 4.51 | 2.68 | 3.32 | 2.11 | 2.43 | 2.54 | 3.27 | 2.31 | 3.97 | 3.65 | 3.46 | 3.62 |
| SKP1 | VIT_201s0010g02370 | 19.70 | 20.36 | 22.50 | 21.85 | 24.71 | 30.28 | 30.02 | 30.64 | 41.24 | 38.83 | 39.64 | 43.42 | 44.59 | 43.18 |
|  | VIT_217s0000g00300 | 23.35 | 21.28 | 20.91 | 24.59 | 28.70 | 23.89 | 26.35 | 28.08 | 29.23 | 26.77 | 24.74 | 26.52 | 28.50 | 26.80 |
|  | VIT_208s0007g03160 | 41.05 | 22.13 | 13.44 | 12.53 | 10.01 | 10.62 | 9.88 | 12.12 | 16.64 | 13.45 | 15.09 | 13.87 | 13.95 | 12.96 |
|  | VIT_209s0002g07290 | 30.60 | 29.64 | 33.06 | 38.50 | 33.50 | 43.47 | 39.80 | 43.18 | 63.98 | 55.84 | 53.66 | 70.12 | 67.30 | 69.91 |
|  | VIT_205s0020g00020 | 30.20 | 56.28 | 37.99 | 39.91 | 41.04 | 36.35 | 29.39 | 31.84 | 43.64 | 33.18 | 34.01 | 35.44 | 32.66 | 36.28 |
|  | VIT_205s0020g00570 | 2.98 | 5.40 | 3.61 | 3.19 | 4.34 | 4.50 | 6.10 | 4.82 | 5.23 | 6.00 | 4.37 | 5.11 | 4.85 | 4.71 |
|  | VIT_205s0094g00400 | 26.49 | 34.49 | 19.89 | 21.05 | 25.40 | 22.09 | 20.46 | 19.32 | 28.23 | 22.55 | 20.86 | 26.87 | 26.82 | 26.11 |
|  | VIT_213s0067g01780 | 18.34 | 15.56 | 16.85 | 25.55 | 25.47 | 31.18 | 31.26 | 30.82 | 51.52 | 45.97 | 46.41 | 56.34 | 52.34 | 51.58 |
|  | VIT_213s0067g01800 | 27.79 | 30.64 | 31.99 | 35.07 | 46.33 | 28.81 | 33.30 | 32.45 | 25.17 | 25.55 | 24.46 | 28.77 | 27.52 | 27.89 |
|  | VIT_218s0001g02030 | 2.20 | 2.26 | 2.91 | 1.12 | 1.04 | 0.73 | 0.76 | 0.80 | 1.59 | 1.71 | 1.59 | 1.46 | 1.36 | 1.46 |
| F-box | VIT_214s0066g00590 | 18.72 | 27.11 | 21.80 | 20.82 | 18.90 | 22.73 | 24.59 | 24.18 | 26.83 | 27.39 | 27.50 | 27.46 | 28.58 | 26.95 |
|  | VIT_206s0004g06760 | 8.09 | 8.29 | 7.95 | 7.13 | 7.76 | 8.51 | 8.31 | 8.03 | 8.60 | 7.84 | 9.04 | 7.67 | 7.94 | 9.41 |
|  | VIT_207s0031g02690 | 5.84 | 5.08 | 3.75 | 5.14 | 3.75 | 5.72 | 3.97 | 3.40 | 5.37 | 3.31 | 5.20 | 4.45 | 4.13 | 4.52 |
| STO/BBX24 | VIT_200s0131g00030 | 0.17 | 0.00 | 0.00 | 0.18 | 0.00 | 0.00 | 0.00 | 0.00 | 0.00 | 0.00 | 0.00 | 0.00 | 0.00 | 0.00 |
|  | VIT_200s0125g00430 | 0.58 | 0.79 | 1.18 | 0.49 | 1.16 | 0.68 | 2.52 | 1.84 | 2.02 | 2.63 | 1.58 | 1.47 | 1.39 | 1.60 |
|  | VIT_200s0125g00050 | 3.66 | 4.59 | 4.42 | 4.36 | 8.61 | 6.12 | 5.78 | 6.18 | 10.57 | 7.45 | 7.25 | 7.81 | 4.54 | 7.26 |
|  | VIT_200s0120g00010 | 2.85 | 3.07 | 2.96 | 2.69 | 2.53 | 2.85 | 2.99 | 3.58 | 4.78 | 4.40 | 5.72 | 5.99 | 6.32 | 6.42 |
| TOC1 | VIT_217s0000g06520 | 0.00 | 0.25 | 0.16 | 0.00 | 0.24 | 0.08 | 0.08 | 0.00 | 0.08 | 0.08 | 0.16 | 0.33 | 0.00 | 0.08 |
|  | VIT_218s0001g07720 | 21.30 | 16.90 | 17.78 | 17.18 | 16.30 | 20.99 | 18.83 | 19.09 | 29.79 | 24.28 | 26.02 | 31.99 | 33.89 | 30.57 |
|  | VIT_209s0054g00440 | 16.69 | 13.68 | 13.38 | 16.43 | 15.40 | 17.50 | 17.40 | 15.58 | 19.06 | 15.39 | 18.01 | 20.34 | 17.20 | 18.56 |
| ZFP3 | VIT_206s0009g02470 | 0.00 | 0.00 | 0.00 | 0.04 | 0.00 | 0.04 | 0.00 | 0.04 | 0.59 | 0.13 | 0.25 | 0.84 | 0.47 | 0.52 |
| AUX1 | VIT_202s0025g01230 | 5.65 | 1.20 | 2.07 | 1.58 | 1.11 | 3.31 | 2.58 | 3.34 | 11.33 | 11.24 | 11.18 | 16.98 | 18.14 | 10.18 |
|  | VIT_203s0038g02140 | 9.47 | 0.30 | 0.49 | 0.57 | 0.30 | 1.68 | 2.27 | 1.09 | 10.89 | 11.51 | 7.20 | 10.94 | 13.25 | 10.28 |
|  | VIT_203s0038g03540 | 0.87 | 0.03 | 0.32 | 0.06 | 0.03 | 0.09 | 0.00 | 0.06 | 0.03 | 0.00 | 0.11 | 0.00 | 0.00 | 0.12 |
|  | VIT_206s0061g01210 | 0.67 | 7.05 | 7.34 | 1.80 | 0.59 | 1.54 | 0.59 | 2.95 | 4.02 | 1.69 | 10.72 | 3.63 | 1.74 | 2.68 |
|  | VIT_208s0007g02030 | 25.57 | 14.71 | 20.92 | 12.43 | 11.55 | 6.89 | 11.47 | 15.89 | 6.15 | 5.85 | 7.66 | 2.73 | 2.78 | 3.89 |
|  | VIT_213s0067g00330 | 20.91 | 1.75 | 8.61 | 4.79 | 2.60 | 8.79 | 6.52 | 5.63 | 10.07 | 12.88 | 9.35 | 12.92 | 16.04 | 14.17 |
|  | VIT_213s0074g00540 | 1.24 | 2.70 | 2.27 | 3.06 | 2.15 | 2.15 | 2.71 | 3.25 | 2.62 | 2.37 | 2.12 | 0.50 | 0.95 | 1.06 |
|  | VIT_218s0001g03540 | 32.93 | 4.21 | 4.00 | 12.69 | 17.49 | 3.32 | 8.29 | 9.24 | 1.74 | 2.98 | 1.10 | 1.64 | 1.49 | 0.83 |
| TIR1 | VIT_200s0181g00040 | 5.01 | 2.67 | 3.29 | 5.49 | 3.24 | 6.15 | 6.26 | 6.82 | 13.64 | 16.89 | 13.01 | 12.70 | 13.67 | 12.34 |
|  | VIT_200s0208g00110 | 0.00 | 0.00 | 0.18 | 0.00 | 0.00 | 0.00 | 0.00 | 0.00 | 0.00 | 0.00 | 0.00 | 0.00 | 0.00 | 0.00 |
|  | VIT_201s0146g00450 | 12.77 | 10.77 | 11.26 | 10.40 | 10.49 | 7.84 | 7.75 | 8.67 | 7.64 | 6.77 | 7.19 | 5.81 | 5.77 | 5.87 |
|  | VIT_205s0020g00220 | 0.50 | 0.00 | 0.00 | 0.37 | 0.00 | 0.34 | 0.26 | 0.21 | 0.22 | 0.22 | 0.36 | 1.01 | 0.48 | 0.10 |
|  | VIT_205s0020g04830 | 0.00 | 0.14 | 0.00 | 0.10 | 0.00 | 0.03 | 0.03 | 0.00 | 0.00 | 0.00 | 0.00 | 0.00 | 0.10 | 0.25 |
|  | VIT_207s0104g01320 | 16.87 | 7.94 | 10.43 | 3.81 | 3.30 | 3.13 | 4.00 | 6.09 | 4.86 | 3.70 | 5.28 | 2.33 | 2.32 | 2.60 |
|  | VIT_212s0035g00130 | 0.10 | 0.00 | 0.00 | 0.00 | 0.00 | 0.00 | 0.12 | 0.27 | 0.00 | 0.00 | 0.00 | 0.00 | 0.31 | 0.01 |
|  | VIT_214s0030g01240 | 12.38 | 13.71 | 14.85 | 11.39 | 11.54 | 11.29 | 10.10 | 13.10 | 14.29 | 11.87 | 14.76 | 10.49 | 12.58 | 11.08 |
|  | VIT_214s0068g01330 | 46.49 | 36.20 | 39.79 | 63.23 | 55.12 | 58.19 | 62.89 | 68.89 | 81.96 | 78.80 | 70.49 | 67.90 | 67.39 | 67.19 |
|  | VIT_216s0039g02730 | 0.15 | 0.00 | 0.00 | 0.00 | 0.00 | 0.00 | 0.00 | 0.14 | 0.00 | 0.14 | 0.14 | 0.00 | 0.00 | 0.00 |
|  | VIT_218s0001g07120 | 21.65 | 17.68 | 25.08 | 20.48 | 17.40 | 32.73 | 29.53 | 30.98 | 27.25 | 29.61 | 24.19 | 17.72 | 17.81 | 17.00 |
| AUX/IAA | VIT_201s0011g04070 | 0.00 | 0.42 | 0.41 | 0.00 | 0.00 | 0.00 | 0.00 | 0.00 | 0.00 | 0.00 | 0.00 | 0.00 | 0.00 | 0.00 |
|  | VIT_204s0008g00220 | 2.69 | 0.57 | 0.82 | 2.50 | 0.88 | 0.68 | 1.19 | 1.60 | 0.42 | 0.39 | 0.37 | 0.04 | 0.04 | 0.21 |
|  | VIT_204s0008g05560 | 0.00 | 0.00 | 0.00 | 0.00 | 0.00 | 0.00 | 0.00 | 0.32 | 0.05 | 0.00 | 0.24 | 0.00 | 0.00 | 0.05 |
|  | VIT_205s0020g01070 | 21.49 | 1.19 | 2.45 | 0.29 | 0.41 | 7.03 | 1.19 | 3.39 | 2.75 | 0.83 | 3.73 | 3.74 | 5.54 | 12.05 |
|  | VIT_205s0020g04670 | 30.63 | 115.03 | 84.27 | 17.72 | 18.98 | 83.22 | 69.46 | 87.63 | 94.05 | 85.18 | 49.54 | 54.16 | 54.82 | 59.69 |
|  | VIT_205s0020g04690 | 7.43 | 1.55 | 1.80 | 0.04 | 0.09 | 0.17 | 0.09 | 0.87 | 0.22 | 0.09 | 0.23 | 0.17 | 0.18 | 0.40 |
|  | VIT_205s0049g01970 | 14.37 | 2.61 | 4.53 | 6.73 | 4.64 | 12.52 | 11.27 | 12.29 | 13.39 | 13.81 | 11.46 | 8.67 | 10.30 | 10.87 |
|  | VIT_207s0005g04380 | 7.11 | 6.61 | 5.53 | 1.54 | 1.29 | 1.83 | 2.04 | 3.25 | 4.55 | 3.45 | 3.00 | 4.38 | 3.84 | 4.58 |
|  | VIT_207s0141g00270 | 80.34 | 126.27 | 73.92 | 13.71 | 27.87 | 20.77 | 20.17 | 23.33 | 36.51 | 27.34 | 20.54 | 24.78 | 28.91 | 28.20 |
|  | VIT_207s0141g00290 | 48.19 | 37.07 | 22.03 | 21.68 | 26.34 | 19.92 | 29.68 | 23.50 | 16.80 | 20.49 | 13.79 | 8.08 | 9.40 | 7.73 |
|  | VIT_209s0002g03410 | 21.74 | 63.69 | 68.52 | 21.95 | 20.53 | 20.35 | 24.30 | 32.73 | 34.57 | 38.93 | 29.06 | 30.65 | 27.94 | 29.82 |
|  | VIT_209s0002g04080 | 33.08 | 7.96 | 12.96 | 2.02 | 4.87 | 0.69 | 1.73 | 3.53 | 0.35 | 0.54 | 1.59 | 0.32 | 0.24 | 0.73 |
|  | VIT_209s0002g05150 | 37.71 | 24.01 | 27.13 | 187.68 | 128.18 | 379.04 | 339.79 | 312.30 | 244.81 | 307.87 | 201.08 | 132.37 | 161.60 | 154.86 |
|  | VIT_209s0002g05160 | 45.76 | 47.69 | 39.66 | 48.93 | 34.59 | 86.99 | 74.73 | 75.46 | 88.94 | 114.56 | 71.99 | 66.02 | 86.18 | 79.48 |
|  | VIT_211s0016g03540 | 143.72 | 59.92 | 98.62 | 10.06 | 20.59 | 4.83 | 6.38 | 16.54 | 6.10 | 5.77 | 8.18 | 3.45 | 2.84 | 3.73 |
|  | VIT_211s0016g04490 | 50.20 | 133.26 | 97.97 | 50.85 | 66.17 | 27.06 | 35.92 | 36.23 | 23.79 | 22.34 | 25.03 | 18.51 | 16.63 | 21.41 |
|  | VIT_211s0016g05640 | 1.66 | 0.72 | 1.75 | 0.00 | 0.15 | 0.08 | 0.31 | 0.82 | 0.32 | 0.32 | 0.41 | 0.08 | 0.32 | 0.17 |
|  | VIT_211s0052g00870 | 0.38 | 0.51 | 0.32 | 0.13 | 0.25 | 0.19 | 0.32 | 0.31 | 1.35 | 0.82 | 1.35 | 2.26 | 2.11 | 2.16 |
|  | VIT_214s0030g00110 | 0.00 | 0.00 | 0.06 | 0.00 | 0.00 | 0.00 | 0.00 | 0.00 | 0.00 | 0.00 | 0.00 | 0.00 | 0.00 | 0.00 |
|  | VIT_214s0030g02310 | 5.85 | 5.32 | 4.63 | 0.89 | 1.55 | 3.91 | 5.02 | 6.98 | 27.78 | 27.09 | 8.32 | 12.28 | 13.53 | 11.60 |
|  | VIT_214s0081g00010 | 93.95 | 73.91 | 53.42 | 45.49 | 46.35 | 55.61 | 56.57 | 50.59 | 43.14 | 60.25 | 31.12 | 20.25 | 21.70 | 22.33 |
|  | VIT_218s0001g08090 | 235.87 | 174.96 | 168.03 | 66.92 | 83.98 | 63.92 | 64.40 | 72.95 | 34.48 | 32.77 | 34.59 | 24.82 | 31.82 | 31.17 |
| ARF | VIT_200s0198g00140 | 2.97 | 3.62 | 5.11 | 5.00 | 3.07 | 4.24 | 6.71 | 4.53 | 5.79 | 3.63 | 4.82 | 5.91 | 7.15 | 5.81 |
|  | VIT_200s1203g00010 | 0.11 | 1.32 | 1.18 | 0.30 | 0.22 | 0.74 | 1.05 | 0.17 | 0.16 | 0.69 | 0.00 | 0.00 | 0.18 | 0.00 |
|  | VIT_201s0244g00150 | 132.98 | 138.57 | 152.07 | 228.57 | 214.41 | 277.59 | 279.33 | 280.88 | 246.63 | 278.07 | 264.74 | 199.11 | 238.03 | 228.24 |
|  | VIT_202s0025g01740 | 26.34 | 18.79 | 19.32 | 2.80 | 4.60 | 4.06 | 4.82 | 11.43 | 2.82 | 3.12 | 6.90 | 1.26 | 2.30 | 2.06 |
|  | VIT_203s0167g00010 | 0.00 | 0.00 | 0.00 | 0.00 | 0.00 | 0.00 | 0.69 | 0.33 | 0.00 | 0.00 | 0.00 | 0.00 | 0.07 | 1.00 |
|  | VIT_204s0023g02540 | 1.32 | 0.92 | 1.56 | 1.18 | 0.66 | 1.11 | 1.01 | 1.29 | 2.35 | 1.42 | 1.23 | 1.56 | 1.64 | 1.46 |
|  | VIT_204s0043g00940 | 0.06 | 0.00 | 0.00 | 0.00 | 0.13 | 0.00 | 0.06 | 0.33 | 0.00 | 0.00 | 0.07 | 0.00 | 0.00 | 0.00 |
|  | VIT_204s0079g00160 | 7.84 | 5.41 | 7.30 | 4.32 | 5.84 | 3.35 | 4.86 | 5.89 | 2.70 | 3.07 | 2.96 | 2.29 | 2.57 | 2.72 |
|  | VIT_204s0079g00200 | 29.70 | 19.89 | 23.37 | 16.84 | 21.32 | 11.41 | 15.19 | 14.74 | 11.06 | 12.54 | 11.10 | 7.67 | 7.57 | 7.12 |
|  | VIT_205s0020g00190 | 15.70 | 16.45 | 20.95 | 33.44 | 23.43 | 35.06 | 33.59 | 32.46 | 28.26 | 35.53 | 29.62 | 26.65 | 29.64 | 30.79 |
|  | VIT_206s0004g02750 | 16.84 | 19.71 | 23.23 | 10.28 | 10.85 | 9.82 | 9.95 | 11.30 | 11.33 | 10.63 | 10.07 | 6.89 | 5.23 | 6.44 |
|  | VIT_206s0004g03130 | 30.37 | 23.56 | 24.70 | 9.57 | 9.20 | 6.63 | 6.90 | 10.07 | 8.04 | 6.71 | 7.03 | 7.56 | 7.41 | 7.90 |
|  | VIT_207s0104g01230 | 0.21 | 0.00 | 0.79 | 0.02 | 0.00 | 0.00 | 0.08 | 0.54 | 0.04 | 0.11 | 0.84 | 0.10 | 0.20 | 0.11 |
|  | VIT_208s0040g01810 | 10.19 | 9.00 | 8.95 | 5.18 | 5.92 | 3.96 | 4.61 | 5.26 | 7.02 | 6.30 | 7.21 | 6.53 | 6.77 | 7.45 |
|  | VIT_209s0070g00600 | 9.31 | 4.54 | 6.86 | 6.56 | 5.45 | 12.50 | 10.47 | 11.04 | 13.36 | 12.47 | 11.56 | 19.15 | 17.46 | 17.60 |
|  | VIT_210s0003g00420 | 19.94 | 23.49 | 26.56 | 9.36 | 10.02 | 11.84 | 13.79 | 15.35 | 8.58 | 8.38 | 8.06 | 4.00 | 4.09 | 6.08 |
|  | VIT_210s0003g04100 | 23.11 | 27.70 | 31.61 | 6.86 | 9.05 | 6.26 | 7.89 | 11.47 | 6.51 | 5.59 | 9.21 | 4.84 | 4.04 | 4.33 |
|  | VIT_211s0016g00640 | 10.64 | 12.10 | 14.98 | 18.96 | 14.14 | 27.00 | 24.25 | 27.99 | 34.41 | 39.88 | 44.17 | 35.12 | 35.83 | 38.63 |
|  | VIT_211s0065g00310 | 46.07 | 45.57 | 56.25 | 19.55 | 23.44 | 28.12 | 30.60 | 43.17 | 26.81 | 35.56 | 31.55 | 30.22 | 32.86 | 33.64 |
|  | VIT_212s0028g01170 | 26.01 | 26.23 | 36.24 | 17.78 | 18.10 | 14.71 | 20.53 | 21.38 | 16.53 | 18.28 | 18.96 | 14.88 | 17.12 | 15.64 |
|  | VIT_212s0035g01800 | 53.49 | 47.93 | 59.83 | 25.55 | 27.90 | 36.57 | 35.12 | 44.22 | 40.36 | 39.46 | 43.72 | 49.43 | 49.92 | 52.73 |
|  | VIT_213s0019g00110 | 1.19 | 3.24 | 1.78 | 0.91 | 1.20 | 1.78 | 1.75 | 1.01 | 1.74 | 1.76 | 1.02 | 1.03 | 1.33 | 1.42 |
|  | VIT_213s0019g04380 | 16.29 | 14.53 | 15.11 | 2.84 | 4.84 | 4.20 | 4.77 | 5.11 | 4.17 | 4.18 | 4.07 | 3.36 | 3.99 | 4.20 |
|  | VIT_215s0046g00290 | 35.09 | 22.36 | 30.68 | 1.40 | 2.50 | 0.91 | 0.97 | 2.40 | 1.70 | 1.82 | 2.07 | 1.90 | 1.69 | 1.88 |
|  | VIT_217s0000g00320 | 44.21 | 47.14 | 58.43 | 35.45 | 30.75 | 49.47 | 52.37 | 59.55 | 54.82 | 54.82 | 56.41 | 69.57 | 69.48 | 72.21 |
|  | VIT_217s0000g03900 | 0.00 | 0.00 | 0.00 | 0.00 | 0.00 | 2.12 | 0.00 | 1.12 | 4.43 | 2.16 | 1.08 | 11.98 | 9.83 | 5.61 |
|  | VIT_218s0001g04180 | 7.40 | 6.78 | 7.42 | 5.08 | 4.40 | 6.26 | 6.52 | 6.79 | 5.20 | 6.00 | 6.09 | 4.63 | 4.34 | 4.29 |
|  | VIT_218s0001g13930 | 9.82 | 6.59 | 9.20 | 20.44 | 15.55 | 16.38 | 16.20 | 16.31 | 23.05 | 22.04 | 28.73 | 34.81 | 33.32 | 32.28 |
|  | VIT_218s0089g00910 | 57.40 | 57.05 | 77.89 | 19.20 | 25.74 | 17.79 | 20.68 | 26.21 | 21.83 | 18.84 | 25.08 | 26.47 | 24.53 | 25.76 |
| GH3 | VIT_201s0150g00300 | 2.59 | 2.26 | 2.95 | 1.92 | 1.97 | 0.89 | 1.69 | 1.56 | 0.49 | 0.57 | 0.46 | 0.29 | 0.15 | 0.11 |
|  | VIT_203s0091g00310 | 69.55 | 42.80 | 35.11 | 43.68 | 27.89 | 56.95 | 53.22 | 69.33 | 55.31 | 70.49 | 59.63 | 21.71 | 22.61 | 29.12 |
|  | VIT_204s0008g04880 | 2.84 | 3.28 | 3.38 | 0.92 | 1.42 | 0.91 | 1.12 | 0.80 | 0.93 | 1.97 | 1.30 | 0.79 | 0.44 | 0.82 |
|  | VIT_207s0005g00090 | 28.28 | 6.55 | 14.58 | 0.42 | 1.13 | 0.52 | 0.62 | 0.34 | 0.09 | 0.06 | 0.14 | 0.07 | 0.00 | 0.03 |
|  | VIT_207s0104g00800 | 7.33 | 6.99 | 7.33 | 5.32 | 4.77 | 5.94 | 5.67 | 6.29 | 8.24 | 8.57 | 6.95 | 4.72 | 4.22 | 4.21 |
|  | VIT_207s0129g00660 | 10.82 | 27.83 | 21.60 | 0.25 | 1.04 | 0.02 | 0.02 | 0.91 | 0.04 | 0.15 | 0.18 | 0.13 | 0.07 | 0.18 |
|  | VIT_212s0034g00640 | 0.00 | 0.16 | 0.49 | 0.00 | 0.15 | 0.00 | 0.18 | 0.15 | 0.16 | 0.31 | 0.53 | 0.48 | 0.59 | 0.32 |
|  | VIT_212s0059g01870 | 1.08 | 0.18 | 0.08 | 1.08 | 0.35 | 0.76 | 0.81 | 1.12 | 0.84 | 0.46 | 0.91 | 0.18 | 0.05 | 0.24 |
|  | VIT_212s0134g00230 | 0.03 | 0.00 | 0.00 | 0.00 | 0.00 | 0.00 | 0.00 | 0.00 | 0.00 | 0.00 | 0.00 | 0.00 | 0.00 | 0.00 |
|  | VIT_219s0014g04690 | 28.90 | 0.74 | 4.32 | 0.16 | 0.31 | 0.18 | 0.34 | 3.98 | 0.64 | 0.83 | 4.10 | 0.90 | 1.32 | 1.78 |
| SAUR | VIT_201s0146g00180 | 1.17 | 0.62 | 0.96 | 0.36 | 0.61 | 0.11 | 0.16 | 1.41 | 0.11 | 0.39 | 1.37 | 0.34 | 0.00 | 0.29 |
|  | VIT_202s0154g00010 | 2.56 | 3.55 | 1.02 | 0.84 | 0.44 | 0.36 | 0.43 | 1.15 | 1.56 | 1.32 | 1.11 | 3.53 | 2.89 | 2.20 |
|  | VIT_203s0038g00930 | 1.33 | 0.21 | 0.99 | 0.21 | 0.00 | 0.00 | 0.07 | 0.14 | 0.15 | 0.07 | 0.36 | 0.00 | 0.07 | 0.15 |
|  | VIT_203s0038g00940 | 10.98 | 13.66 | 5.20 | 11.45 | 13.47 | 6.70 | 6.79 | 6.75 | 5.46 | 5.80 | 3.30 | 1.16 | 0.75 | 0.94 |
|  | VIT_203s0038g00950 | 1.65 | 0.09 | 0.47 | 0.00 | 0.09 | 0.00 | 0.00 | 0.27 | 0.46 | 0.64 | 0.19 | 0.65 | 1.02 | 0.67 |
|  | VIT_203s0038g01080 | 0.00 | 0.00 | 0.00 | 0.23 | 0.00 | 0.47 | 0.24 | 0.00 | 0.00 | 0.00 | 0.00 | 0.00 | 0.00 | 0.25 |
|  | VIT_203s0038g01090 | 3.40 | 1.42 | 0.17 | 0.16 | 0.16 | 0.00 | 0.00 | 0.00 | 0.00 | 0.00 | 0.00 | 0.00 | 0.00 | 0.00 |
|  | VIT_203s0038g01100 | 0.24 | 0.00 | 0.00 | 0.00 | 0.00 | 0.00 | 0.25 | 0.00 | 0.00 | 0.00 | 0.00 | 0.00 | 0.00 | 0.00 |
|  | VIT_203s0038g01110 | 8.43 | 2.70 | 0.31 | 0.23 | 0.35 | 0.00 | 0.11 | 0.00 | 0.00 | 0.12 | 0.00 | 0.00 | 0.00 | 0.00 |
|  | VIT_203s0038g01130 | 7.04 | 2.42 | 1.50 | 0.59 | 0.13 | 0.00 | 0.19 | 0.13 | 0.00 | 0.00 | 0.00 | 0.00 | 0.00 | 0.00 |
|  | VIT_203s0038g01150 | 2.78 | 0.77 | 0.29 | 0.19 | 0.75 | 0.00 | 0.09 | 0.00 | 0.00 | 0.00 | 0.00 | 0.00 | 0.00 | 0.00 |
|  | VIT_203s0038g01160 | 0.00 | 0.00 | 0.00 | 0.24 | 0.00 | 0.00 | 0.00 | 0.00 | 0.00 | 0.00 | 0.00 | 0.00 | 0.00 | 0.00 |
|  | VIT_203s0038g01170 | 0.00 | 0.00 | 0.00 | 0.23 | 0.00 | 0.00 | 0.00 | 0.00 | 0.00 | 0.00 | 0.00 | 0.00 | 0.00 | 0.00 |
|  | VIT_203s0038g01180 | 0.00 | 0.00 | 0.00 | 1.27 | 0.40 | 0.62 | 0.20 | 0.41 | 0.21 | 0.42 | 0.21 | 0.22 | 0.00 | 0.00 |
|  | VIT_203s0038g01190 | 0.75 | 0.00 | 0.00 | 0.18 | 0.00 | 0.00 | 0.00 | 0.18 | 0.00 | 0.00 | 0.00 | 0.00 | 0.00 | 0.00 |
|  | VIT_203s0038g01210 | 0.30 | 0.00 | 0.00 | 0.00 | 0.00 | 0.00 | 0.15 | 0.00 | 0.00 | 0.00 | 0.00 | 0.00 | 0.00 | 0.00 |
|  | VIT_203s0038g01220 | 0.00 | 0.00 | 0.00 | 0.35 | 0.00 | 0.00 | 0.00 | 0.00 | 0.00 | 0.00 | 0.00 | 0.00 | 0.00 | 0.00 |
|  | VIT_203s0038g01230 | 0.09 | 0.00 | 0.00 | 0.00 | 0.00 | 0.00 | 0.00 | 0.00 | 0.00 | 0.00 | 0.00 | 0.00 | 0.00 | 0.00 |
|  | VIT_203s0038g01250 | 0.00 | 0.00 | 0.00 | 0.00 | 0.12 | 0.00 | 0.00 | 0.00 | 0.00 | 0.00 | 0.00 | 0.00 | 0.00 | 0.00 |
|  | VIT_203s0038g01260 | 0.00 | 0.00 | 0.00 | 0.00 | 0.00 | 0.00 | 0.23 | 0.00 | 0.00 | 0.00 | 0.00 | 0.00 | 0.00 | 0.00 |
|  | VIT_203s0038g01280 | 0.00 | 0.00 | 0.00 | 0.21 | 0.00 | 0.00 | 0.00 | 0.00 | 0.00 | 0.00 | 0.00 | 0.00 | 0.00 | 0.00 |
|  | VIT_203s0038g01285 | 0.00 | 0.00 | 0.00 | 0.00 | 0.00 | 0.00 | 0.09 | 0.18 | 0.00 | 0.00 | 0.00 | 0.00 | 0.00 | 0.00 |
|  | VIT_203s0038g01300 | 2.22 | 7.06 | 0.83 | 1.21 | 2.02 | 0.00 | 0.81 | 1.45 | 0.61 | 0.20 | 0.00 | 0.41 | 0.41 | 0.00 |
|  | VIT_203s0038g01310 | 1.57 | 4.58 | 2.34 | 3.67 | 4.08 | 2.94 | 3.92 | 4.26 | 5.63 | 3.87 | 2.64 | 3.30 | 4.00 | 2.78 |
|  | VIT_203s0038g03450 | 0.00 | 0.18 | 0.00 | 1.57 | 0.84 | 0.85 | 1.02 | 0.33 | 0.71 | 0.51 | 1.05 | 0.34 | 0.00 | 0.54 |
|  | VIT_203s0038g03470 | 0.00 | 0.00 | 0.00 | 0.00 | 0.00 | 0.10 | 0.07 | 0.00 | 0.07 | 0.00 | 0.00 | 0.00 | 0.00 | 0.00 |
|  | VIT_203s0038g03480 | 0.25 | 0.00 | 0.00 | 0.13 | 0.00 | 0.33 | 0.00 | 0.25 | 0.00 | 0.00 | 0.00 | 0.00 | 0.00 | 0.13 |
|  | VIT_203s0038g03500 | 0.07 | 0.00 | 0.00 | 0.00 | 0.07 | 0.28 | 0.35 | 0.35 | 0.43 | 0.35 | 0.07 | 0.00 | 0.07 | 0.00 |
|  | VIT_203s0038g03520 | 0.00 | 0.00 | 0.21 | 0.07 | 0.13 | 0.20 | 0.07 | 0.14 | 0.00 | 0.00 | 0.07 | 0.00 | 0.00 | 0.00 |
|  | VIT_203s0038g03756 | 0.34 | 0.12 | 0.13 | 0.19 | 0.09 | 0.09 | 0.15 | 0.10 | 0.19 | 0.06 | 0.00 | 0.22 | 0.19 | 0.07 |
|  | VIT_203s0038g03760 | 0.40 | 0.14 | 0.00 | 0.00 | 0.00 | 0.13 | 0.40 | 0.00 | 0.29 | 0.00 | 0.00 | 0.00 | 0.14 | 0.00 |
|  | VIT_203s0038g03770 | 0.12 | 0.47 | 0.13 | 0.07 | 0.13 | 0.06 | 0.38 | 0.13 | 0.00 | 0.06 | 0.13 | 0.07 | 0.13 | 0.07 |
|  | VIT_203s0038g03780 | 0.10 | 0.00 | 0.00 | 0.29 | 0.00 | 0.54 | 0.27 | 0.18 | 0.18 | 0.27 | 0.00 | 0.00 | 0.00 | 0.00 |
|  | VIT_204s0008g02800 | 0.50 | 1.24 | 0.50 | 12.64 | 6.49 | 6.95 | 7.42 | 5.10 | 3.67 | 5.09 | 2.49 | 0.92 | 1.08 | 0.94 |
|  | VIT_204s0023g00490 | 0.12 | 0.00 | 0.00 | 0.50 | 0.58 | 0.70 | 0.59 | 0.48 | 0.24 | 0.24 | 0.00 | 0.00 | 0.00 | 0.00 |
|  | VIT_204s0023g00500 | 0.00 | 0.00 | 0.23 | 0.00 | 0.00 | 0.00 | 0.00 | 0.47 | 0.00 | 0.45 | 0.00 | 0.00 | 1.15 | 0.23 |
|  | VIT_204s0023g00505 | 0.35 | 0.12 | 0.36 | 0.13 | 0.00 | 0.12 | 0.58 | 0.00 | 0.00 | 0.12 | 0.00 | 0.00 | 0.00 | 0.00 |
|  | VIT_204s0023g00510 | 0.00 | 0.00 | 0.23 | 0.46 | 0.22 | 0.22 | 0.66 | 0.68 | 0.00 | 0.22 | 0.24 | 0.00 | 0.00 | 0.00 |
|  | VIT_204s0023g00520 | 0.00 | 0.00 | 0.00 | 1.12 | 0.22 | 2.85 | 2.21 | 1.77 | 2.96 | 3.79 | 0.68 | 0.24 | 0.45 | 0.23 |
|  | VIT_204s0023g00530 | 0.00 | 0.00 | 0.18 | 1.62 | 0.17 | 2.54 | 2.06 | 1.74 | 3.16 | 1.96 | 1.80 | 1.11 | 0.72 | 0.55 |
|  | VIT_204s0023g00540 | 0.21 | 0.00 | 0.00 | 0.00 | 0.21 | 0.00 | 0.00 | 0.00 | 0.00 | 0.00 | 0.00 | 0.00 | 0.00 | 0.00 |
|  | VIT_204s0023g00550 | 0.00 | 0.00 | 0.00 | 0.28 | 0.11 | 0.29 | 0.65 | 0.33 | 0.11 | 0.00 | 0.11 | 0.00 | 0.11 | 0.12 |
|  | VIT_204s0023g00555 | 0.00 | 0.09 | 0.00 | 0.33 | 0.09 | 0.98 | 0.49 | 0.29 | 0.70 | 0.28 | 0.18 | 0.09 | 0.19 | 0.19 |
|  | VIT_204s0023g00560 | 0.00 | 0.13 | 0.00 | 0.26 | 0.12 | 0.13 | 0.13 | 0.13 | 0.00 | 0.26 | 0.00 | 0.00 | 0.00 | 0.00 |
|  | VIT_204s0023g00580 | 6.91 | 1.99 | 1.72 | 2.70 | 1.93 | 3.73 | 2.35 | 1.57 | 3.04 | 3.65 | 2.25 | 0.40 | 0.40 | 0.68 |
|  | VIT_204s0023g03230 | 2.66 | 2.31 | 0.53 | 27.87 | 12.21 | 20.37 | 16.17 | 15.52 | 24.78 | 21.64 | 15.07 | 11.04 | 12.16 | 12.58 |
|  | VIT_207s0031g02740 | 4.39 | 1.17 | 0.85 | 3.44 | 1.75 | 3.84 | 5.76 | 2.02 | 12.94 | 4.01 | 4.70 | 9.99 | 8.10 | 8.10 |
|  | VIT_208s0007g03120 | 3.03 | 0.39 | 0.13 | 0.00 | 0.00 | 0.13 | 0.00 | 0.00 | 0.25 | 0.00 | 0.00 | 0.12 | 0.00 | 0.13 |
|  | VIT_208s0007g04145 | 0.00 | 0.00 | 0.00 | 0.00 | 0.15 | 0.00 | 0.00 | 0.00 | 0.17 | 0.00 | 0.00 | 0.49 | 0.00 | 0.17 |
|  | VIT_208s0040g01390 | 2.47 | 3.05 | 2.77 | 2.92 | 2.33 | 1.94 | 3.35 | 2.86 | 4.74 | 4.06 | 4.10 | 7.34 | 10.22 | 8.59 |
|  | VIT_208s0040g01395 | 6.73 | 0.00 | 0.00 | 0.00 | 0.00 | 0.00 | 0.00 | 0.00 | 0.00 | 0.00 | 0.00 | 0.30 | 0.00 | 0.00 |
|  | VIT_208s0040g01425 | 0.91 | 0.44 | 0.73 | 0.25 | 0.43 | 0.11 | 0.28 | 0.18 | 0.18 | 0.32 | 0.15 | 0.32 | 0.44 | 0.15 |
|  | VIT_208s0040g01670 | 0.00 | 0.17 | 0.00 | 0.00 | 0.00 | 0.00 | 0.00 | 0.00 | 0.00 | 0.00 | 0.51 | 0.34 | 0.00 | 0.52 |
|  | VIT_208s0058g01160 | 17.63 | 18.15 | 8.47 | 4.49 | 9.63 | 2.69 | 3.05 | 3.90 | 0.72 | 0.83 | 2.76 | 0.00 | 0.12 | 0.73 |
|  | VIT_209s0002g00650 | 4.80 | 5.45 | 4.45 | 2.14 | 0.37 | 1.70 | 2.96 | 1.77 | 3.05 | 2.63 | 3.40 | 0.61 | 1.36 | 1.56 |
|  | VIT_209s0002g00670 | 13.82 | 11.41 | 10.04 | 0.00 | 0.90 | 0.44 | 0.14 | 3.50 | 1.09 | 1.51 | 2.74 | 0.60 | 1.22 | 0.15 |
|  | VIT_209s0054g00660 | 0.00 | 0.12 | 0.00 | 0.00 | 0.00 | 0.00 | 0.00 | 0.12 | 0.00 | 0.00 | 0.00 | 0.12 | 0.12 | 0.13 |
|  | VIT_209s0054g00725 | 0.24 | 0.90 | 0.28 | 0.05 | 0.19 | 0.05 | 0.09 | 0.14 | 0.20 | 0.05 | 0.20 | 0.10 | 0.09 | 0.10 |
|  | VIT_209s0054g00740 | 0.00 | 0.12 | 0.25 | 0.13 | 0.00 | 0.00 | 0.00 | 0.00 | 0.00 | 0.00 | 0.00 | 0.00 | 0.00 | 0.00 |
|  | VIT_210s0003g05755 | 12.98 | 42.42 | 21.87 | 28.81 | 26.16 | 24.02 | 24.52 | 16.45 | 13.83 | 13.75 | 12.10 | 2.44 | 1.86 | 3.02 |
|  | VIT_211s0016g00500 | 3.44 | 2.52 | 1.56 | 10.97 | 12.07 | 5.67 | 7.64 | 5.41 | 6.89 | 4.54 | 5.13 | 9.03 | 6.27 | 6.05 |
|  | VIT_211s0016g00520 | 0.52 | 7.22 | 3.49 | 0.91 | 1.13 | 1.11 | 0.98 | 1.61 | 2.47 | 2.07 | 2.09 | 0.82 | 0.74 | 0.66 |
|  | VIT_211s0078g00400 | 1.25 | 0.32 | 0.31 | 0.00 | 0.10 | 0.10 | 0.00 | 0.32 | 0.21 | 0.21 | 0.00 | 0.21 | 0.21 | 0.22 |
|  | VIT_215s0048g00530 | 23.90 | 19.10 | 12.98 | 7.05 | 4.90 | 6.85 | 4.25 | 5.75 | 11.10 | 6.96 | 9.39 | 12.20 | 8.15 | 12.02 |
|  | VIT_215s0048g02860 | 13.93 | 13.09 | 6.75 | 17.34 | 12.38 | 16.16 | 13.39 | 9.30 | 21.32 | 17.69 | 18.40 | 17.67 | 16.16 | 18.42 |
|  | VIT_216s0098g01150 | 15.10 | 13.06 | 6.45 | 39.39 | 53.69 | 48.96 | 37.75 | 42.41 | 103.10 | 110.35 | 93.81 | 182.66 | 155.57 | 134.66 |
|  | VIT_217s0000g00931 | 0.06 | 0.00 | 0.00 | 0.07 | 0.00 | 0.06 | 0.00 | 0.19 | 0.00 | 0.06 | 0.00 | 0.00 | 0.00 | 0.00 |
|  | VIT_218s0001g00300 | 0.00 | 0.00 | 0.00 | 0.00 | 0.00 | 0.00 | 0.16 | 0.00 | 0.00 | 0.00 | 0.00 | 0.00 | 0.00 | 0.00 |
|  | VIT_218s0001g13960 | 2.16 | 0.87 | 0.35 | 0.72 | 1.10 | 0.68 | 0.59 | 0.26 | 0.17 | 0.60 | 0.18 | 0.18 | 0.09 | 0.09 |
|  | VIT_218s0001g13980 | 0.29 | 0.00 | 0.14 | 0.00 | 0.14 | 0.28 | 0.00 | 0.00 | 0.15 | 0.29 | 0.00 | 0.00 | 0.29 | 0.60 |
|  | VIT_218s0001g14000 | 0.14 | 0.14 | 0.98 | 0.29 | 0.13 | 0.00 | 0.14 | 0.00 | 0.43 | 0.14 | 0.29 | 0.42 | 0.28 | 0.29 |
|  | VIT_219s0027g00510 | 0.26 | 0.00 | 0.00 | 0.00 | 0.00 | 0.00 | 0.00 | 0.00 | 0.00 | 0.00 | 0.00 | 0.00 | 0.00 | 0.00 |
|  | VIT_219s0085g00010 | 12.26 | 1.63 | 2.43 | 0.53 | 0.54 | 0.26 | 0.09 | 0.53 | 0.99 | 0.53 | 0.82 | 0.80 | 0.54 | 0.46 |
| CRE1 | VIT_201s0010g03780 | 17.80 | 20.22 | 21.81 | 19.05 | 18.03 | 20.95 | 23.88 | 31.76 | 25.91 | 27.25 | 32.96 | 29.85 | 32.43 | 29.96 |
|  | VIT_201s0011g00570 | 43.44 | 44.79 | 32.61 | 76.64 | 72.03 | 78.84 | 87.86 | 87.62 | 73.60 | 89.82 | 77.55 | 65.56 | 77.88 | 65.50 |
|  | VIT_201s0011g05500 | 12.31 | 5.98 | 8.74 | 16.18 | 11.63 | 22.68 | 21.48 | 21.33 | 18.94 | 24.22 | 18.17 | 12.88 | 12.13 | 13.96 |
|  | VIT_201s0011g06190 | 9.30 | 1.00 | 2.54 | 2.63 | 2.62 | 4.02 | 5.37 | 4.58 | 8.25 | 9.28 | 7.58 | 5.74 | 6.75 | 5.65 |
|  | VIT_203s0017g02240 | 4.31 | 0.10 | 0.14 | 0.49 | 0.19 | 0.47 | 0.47 | 0.38 | 0.00 | 0.05 | 0.00 | 0.00 | 0.05 | 0.10 |
|  | VIT_203s0063g02490 | 177.34 | 32.46 | 53.41 | 18.29 | 15.30 | 6.99 | 11.82 | 16.57 | 10.31 | 11.11 | 12.37 | 7.87 | 8.35 | 8.41 |
|  | VIT_204s0008g03460 | 0.92 | 1.26 | 1.11 | 0.60 | 0.81 | 0.52 | 1.44 | 0.75 | 1.17 | 0.99 | 1.23 | 0.81 | 1.09 | 1.04 |
|  | VIT_204s0023g03680 | 6.66 | 2.49 | 3.98 | 1.03 | 1.68 | 1.63 | 2.43 | 3.11 | 2.27 | 2.31 | 1.90 | 1.39 | 1.30 | 1.73 |
|  | VIT_204s0069g00750 | 0.26 | 0.19 | 0.29 | 0.32 | 0.11 | 0.44 | 0.55 | 0.13 | 0.28 | 0.17 | 0.23 | 0.06 | 0.08 | 0.08 |
|  | VIT_205s0029g00190 | 0.74 | 2.23 | 17.59 | 0.98 | 0.20 | 0.86 | 0.98 | 1.04 | 1.81 | 1.82 | 1.48 | 1.58 | 1.47 | 1.43 |
|  | VIT_205s0029g00250 | 0.10 | 0.00 | 0.00 | 0.00 | 0.00 | 0.00 | 0.00 | 0.21 | 0.00 | 0.00 | 0.45 | 0.11 | 0.32 | 0.34 |
|  | VIT_205s0062g01430 | 27.95 | 24.22 | 20.16 | 5.86 | 5.89 | 3.74 | 3.11 | 4.53 | 2.80 | 2.54 | 3.10 | 1.43 | 1.45 | 1.87 |
|  | VIT_206s0004g00720 | 11.08 | 3.58 | 5.90 | 2.84 | 3.93 | 2.84 | 3.42 | 3.40 | 2.90 | 2.67 | 2.73 | 2.41 | 2.24 | 2.39 |
|  | VIT_207s0005g01380 | 0.53 | 0.00 | 0.11 | 0.00 | 0.00 | 0.00 | 0.00 | 0.02 | 0.00 | 0.00 | 0.02 | 0.02 | 0.00 | 0.00 |
|  | VIT_207s0005g05040 | 1.33 | 0.15 | 0.60 | 0.61 | 0.57 | 0.58 | 0.44 | 0.00 | 0.73 | 0.45 | 0.89 | 1.52 | 1.05 | 1.23 |
|  | VIT_207s0129g00230 | 0.46 | 0.07 | 0.08 | 0.03 | 0.03 | 0.07 | 0.06 | 0.08 | 0.04 | 0.00 | 0.10 | 0.00 | 0.03 | 0.03 |
|  | VIT_210s0003g02720 | 5.02 | 0.17 | 0.79 | 1.43 | 0.75 | 1.59 | 1.17 | 1.14 | 2.57 | 2.50 | 1.79 | 4.52 | 4.25 | 3.73 |
|  | VIT_210s0003g02960 | 88.89 | 0.29 | 1.54 | 0.00 | 0.00 | 0.00 | 0.00 | 0.00 | 0.09 | 0.19 | 0.38 | 0.19 | 0.10 | 0.10 |
|  | VIT_210s0003g04340 | 0.46 | 0.00 | 0.09 | 0.00 | 0.00 | 0.00 | 0.09 | 0.00 | 0.00 | 0.00 | 0.00 | 0.00 | 0.00 | 0.00 |
|  | VIT_210s0116g01640 | 14.08 | 17.75 | 16.05 | 16.27 | 15.47 | 22.36 | 24.65 | 20.33 | 17.92 | 20.01 | 20.68 | 13.31 | 14.97 | 15.14 |
|  | VIT_211s0016g00220 | 2.37 | 0.09 | 0.13 | 0.31 | 0.12 | 0.25 | 0.40 | 0.32 | 0.07 | 0.09 | 0.13 | 0.09 | 0.06 | 0.06 |
|  | VIT_211s0016g02960 | 22.77 | 12.70 | 12.78 | 7.32 | 9.50 | 3.93 | 5.44 | 6.06 | 2.50 | 3.23 | 3.11 | 1.45 | 1.88 | 1.88 |
|  | VIT_211s0016g04360 | 0.85 | 1.09 | 1.93 | 1.53 | 0.56 | 1.21 | 1.65 | 0.99 | 0.35 | 0.30 | 0.55 | 0.58 | 0.58 | 0.57 |
|  | VIT_212s0028g00050 | 3.03 | 5.89 | 4.12 | 4.21 | 5.25 | 3.44 | 3.68 | 3.42 | 4.17 | 5.38 | 4.31 | 1.81 | 1.25 | 1.29 |
|  | VIT_212s0028g02540 | 15.29 | 25.55 | 13.63 | 19.84 | 23.93 | 14.37 | 14.27 | 12.66 | 13.49 | 12.02 | 10.04 | 5.93 | 7.86 | 7.43 |
|  | VIT_212s0057g00690 | 2.62 | 4.73 | 6.06 | 5.50 | 4.04 | 8.69 | 7.60 | 8.56 | 12.02 | 12.19 | 13.41 | 12.32 | 13.73 | 13.60 |
|  | VIT_212s0057g00700 | 0.94 | 4.60 | 0.74 | 16.28 | 18.54 | 15.47 | 14.79 | 11.28 | 5.03 | 7.32 | 4.39 | 0.76 | 1.01 | 1.29 |
|  | VIT_212s0059g01320 | 75.07 | 25.22 | 19.09 | 44.05 | 26.21 | 38.89 | 30.49 | 31.22 | 41.07 | 43.19 | 46.83 | 25.82 | 31.17 | 28.37 |
|  | VIT_213s0019g01180 | 49.14 | 56.44 | 65.25 | 21.45 | 44.16 | 9.44 | 15.72 | 20.80 | 6.06 | 6.48 | 10.70 | 8.36 | 6.08 | 7.38 |
|  | VIT_214s0006g00520 | 7.29 | 5.53 | 3.39 | 8.05 | 7.68 | 8.96 | 9.41 | 7.59 | 3.10 | 4.50 | 3.46 | 0.95 | 1.36 | 2.06 |
|  | VIT_214s0060g00430 | 19.79 | 29.25 | 26.35 | 13.89 | 18.47 | 11.06 | 14.76 | 15.57 | 11.68 | 11.46 | 11.94 | 11.20 | 10.75 | 11.20 |
|  | VIT_214s0060g00440 | 9.83 | 8.97 | 9.01 | 7.89 | 9.52 | 3.98 | 9.15 | 7.18 | 2.22 | 1.75 | 1.99 | 2.35 | 2.12 | 2.40 |
|  | VIT_216s0098g01560 | 2.07 | 0.16 | 0.24 | 0.41 | 0.20 | 0.52 | 0.44 | 0.25 | 0.49 | 0.28 | 0.16 | 0.12 | 0.16 | 0.09 |
|  | VIT_217s0000g04920 | 55.92 | 39.51 | 67.11 | 12.71 | 35.78 | 7.16 | 8.25 | 19.83 | 8.50 | 9.87 | 5.56 | 0.45 | 0.45 | 0.57 |
|  | VIT_217s0000g07260 | 57.73 | 31.77 | 35.18 | 27.58 | 26.25 | 32.17 | 30.78 | 30.83 | 46.58 | 47.00 | 42.32 | 55.85 | 61.78 | 51.24 |
|  | VIT_218s0001g12390 | 13.28 | 10.69 | 14.66 | 50.40 | 26.71 | 26.75 | 30.23 | 30.60 | 18.26 | 18.01 | 18.01 | 16.57 | 18.17 | 14.24 |
|  | VIT_218s0122g00980 | 63.11 | 10.78 | 8.35 | 2.90 | 5.52 | 1.01 | 2.01 | 1.98 | 0.07 | 0.10 | 0.13 | 0.10 | 0.00 | 0.00 |
|  | VIT_219s0014g00090 | 8.86 | 1.89 | 1.08 | 0.14 | 0.27 | 0.00 | 0.07 | 0.07 | 0.00 | 0.00 | 0.00 | 0.06 | 0.07 | 0.00 |
|  | VIT_219s0085g00570 | 0.00 | 0.00 | 0.00 | 0.02 | 0.00 | 0.00 | 0.00 | 0.00 | 0.00 | 0.00 | 0.00 | 0.00 | 0.00 | 0.00 |
|  | VIT_219s0085g00810 | 1.68 | 0.00 | 0.04 | 0.25 | 0.04 | 0.00 | 0.08 | 0.12 | 0.00 | 0.00 | 0.04 | 0.04 | 0.04 | 0.00 |
|  | VIT_219s0090g01070 | 30.34 | 4.08 | 7.67 | 1.08 | 2.32 | 1.09 | 1.49 | 6.48 | 3.73 | 4.43 | 11.94 | 6.19 | 5.14 | 5.54 |
| AHP | VIT_204s0008g00210 | 0.00 | 0.00 | 0.11 | 0.00 | 0.00 | 0.00 | 0.00 | 0.12 | 0.23 | 0.00 | 0.34 | 0.12 | 0.00 | 0.35 |
|  | VIT_205s0020g02210 | 74.08 | 40.30 | 28.86 | 6.59 | 11.98 | 6.19 | 7.86 | 6.21 | 3.90 | 2.83 | 3.13 | 1.73 | 0.88 | 1.36 |
|  | VIT_206s0080g00060 | 1.22 | 2.15 | 1.52 | 0.42 | 0.89 | 0.70 | 0.49 | 0.41 | 0.92 | 0.30 | 0.40 | 1.03 | 0.31 | 0.63 |
|  | VIT_207s0104g00880 | 32.47 | 45.53 | 30.20 | 24.83 | 24.90 | 21.23 | 20.48 | 17.73 | 29.89 | 20.41 | 23.57 | 31.12 | 29.41 | 28.20 |
|  | VIT_209s0002g03520 | 10.21 | 1.15 | 3.34 | 0.28 | 0.80 | 0.10 | 0.05 | 0.67 | 0.00 | 0.05 | 0.05 | 0.00 | 0.00 | 0.11 |
|  | VIT_209s0002g06960 | 0.00 | 0.06 | 0.06 | 0.00 | 0.00 | 0.00 | 0.00 | 0.06 | 0.00 | 0.06 | 0.00 | 0.00 | 0.00 | 0.00 |
|  | VIT_211s0016g03170 | 40.14 | 38.36 | 46.21 | 13.22 | 23.81 | 8.03 | 10.99 | 14.74 | 7.82 | 6.82 | 7.52 | 16.28 | 12.50 | 12.92 |
|  | VIT_213s0074g00735 | 0.73 | 0.24 | 2.50 | 0.00 | 0.00 | 0.00 | 0.00 | 0.74 | 0.26 | 0.00 | 0.26 | 0.00 | 0.25 | 0.25 |
|  | VIT_213s0074g00740 | 1.45 | 0.00 | 1.65 | 0.00 | 0.00 | 0.00 | 0.00 | 0.00 | 0.00 | 0.00 | 0.00 | 0.15 | 0.00 | 0.15 |
|  | VIT_214s0030g00410 | 35.62 | 20.55 | 15.71 | 5.04 | 9.24 | 2.43 | 4.11 | 5.06 | 6.06 | 4.95 | 4.91 | 2.80 | 2.84 | 2.66 |
|  | VIT_219s0027g00430 | 0.41 | 0.42 | 0.55 | 0.28 | 0.40 | 0.55 | 0.68 | 0.27 | 1.41 | 1.52 | 2.26 | 0.99 | 0.84 | 1.45 |
| B-ARR | VIT_200s0475g00030 | 9.18 | 12.37 | 13.09 | 5.96 | 8.18 | 9.16 | 8.79 | 7.47 | 11.26 | 10.29 | 10.63 | 7.64 | 7.33 | 9.30 |
|  | VIT_200s0475g00040 | 18.07 | 15.35 | 18.56 | 10.56 | 11.20 | 12.42 | 11.66 | 12.76 | 18.01 | 16.99 | 17.53 | 18.88 | 18.45 | 17.56 |
|  | VIT_200s1624g00010 | 0.87 | 0.18 | 3.97 | 0.00 | 0.35 | 0.17 | 0.17 | 3.22 | 0.19 | 0.00 | 2.14 | 0.00 | 0.72 | 0.55 |
|  | VIT_201s0010g02230 | 1.78 | 1.37 | 2.01 | 2.68 | 1.92 | 2.66 | 2.44 | 2.44 | 3.89 | 3.05 | 3.25 | 3.59 | 3.08 | 3.88 |
|  | VIT_201s0010g02240 | 2.66 | 1.28 | 0.96 | 1.34 | 1.16 | 2.22 | 1.68 | 1.99 | 1.95 | 2.07 | 2.06 | 2.00 | 2.22 | 2.30 |
|  | VIT_201s0011g03110 | 23.25 | 12.53 | 17.56 | 4.84 | 6.84 | 4.89 | 7.10 | 5.98 | 5.47 | 6.43 | 4.13 | 8.97 | 8.54 | 5.81 |
|  | VIT_201s0011g04193 | 0.66 | 0.37 | 0.37 | 0.12 | 0.25 | 0.08 | 0.10 | 0.12 | 0.00 | 0.00 | 0.02 | 0.00 | 0.00 | 0.02 |
|  | VIT_201s0011g04220 | 0.15 | 0.79 | 0.60 | 0.31 | 0.15 | 0.26 | 0.07 | 0.11 | 0.12 | 0.04 | 0.04 | 0.12 | 0.19 | 0.04 |
|  | VIT_201s0011g05830 | 28.33 | 26.55 | 33.99 | 28.23 | 28.61 | 33.99 | 35.98 | 36.61 | 36.97 | 39.06 | 43.47 | 40.48 | 42.64 | 44.75 |
|  | VIT_201s0026g02550 | 3.72 | 0.42 | 1.03 | 0.72 | 0.58 | 0.73 | 0.68 | 0.70 | 0.85 | 0.36 | 0.55 | 0.19 | 0.27 | 0.40 |
|  | VIT_202s0012g00570 | 32.80 | 16.99 | 8.29 | 9.02 | 4.56 | 7.80 | 5.44 | 6.40 | 7.32 | 4.53 | 5.83 | 2.39 | 1.62 | 2.83 |
|  | VIT_202s0012g01940 | 16.67 | 13.70 | 10.56 | 17.79 | 14.58 | 14.71 | 14.04 | 14.02 | 17.42 | 16.40 | 13.28 | 13.64 | 12.29 | 12.82 |
|  | VIT_203s0017g02280 | 2.44 | 0.00 | 0.09 | 0.28 | 0.30 | 0.12 | 0.17 | 0.17 | 0.18 | 0.00 | 0.00 | 0.04 | 0.31 | 0.28 |
|  | VIT_204s0008g05900 | 15.60 | 9.97 | 11.99 | 11.37 | 7.74 | 16.04 | 15.28 | 15.77 | 15.26 | 16.49 | 15.14 | 13.57 | 14.90 | 15.89 |
|  | VIT_205s0029g00060 | 18.14 | 11.60 | 9.09 | 15.79 | 14.74 | 14.74 | 12.10 | 12.85 | 11.62 | 10.86 | 10.86 | 11.37 | 12.40 | 11.25 |
|  | VIT_205s0077g01480 | 22.91 | 38.52 | 42.06 | 51.51 | 58.74 | 61.45 | 68.67 | 64.65 | 46.08 | 61.07 | 55.72 | 35.36 | 31.68 | 34.38 |
|  | VIT_206s0004g05120 | 1.47 | 3.85 | 2.88 | 3.11 | 2.43 | 4.91 | 3.30 | 3.38 | 7.79 | 5.98 | 7.18 | 5.18 | 4.01 | 5.03 |
|  | VIT_207s0005g01010 | 0.00 | 0.00 | 0.00 | 0.00 | 0.00 | 0.03 | 0.00 | 0.04 | 0.00 | 0.00 | 0.00 | 0.07 | 0.00 | 0.00 |
|  | VIT_207s0005g04120 | 19.59 | 14.86 | 18.83 | 20.03 | 17.31 | 21.01 | 21.51 | 20.55 | 26.45 | 25.44 | 25.08 | 35.41 | 34.50 | 31.65 |
|  | VIT_208s0007g06180 | 2.25 | 1.25 | 0.79 | 0.83 | 0.65 | 0.49 | 0.36 | 0.60 | 0.49 | 0.46 | 0.43 | 0.65 | 1.03 | 0.80 |
|  | VIT_208s0007g06310 | 4.90 | 9.99 | 7.78 | 5.08 | 7.51 | 3.59 | 3.99 | 4.84 | 2.47 | 1.43 | 2.69 | 1.24 | 0.99 | 1.14 |
|  | VIT_208s0007g07580 | 5.89 | 1.03 | 2.97 | 4.65 | 3.35 | 3.02 | 3.24 | 3.65 | 4.99 | 4.05 | 3.78 | 3.69 | 3.45 | 4.31 |
|  | VIT_208s0040g00100 | 18.49 | 15.52 | 13.90 | 17.49 | 13.22 | 18.18 | 14.68 | 17.00 | 27.17 | 22.13 | 22.80 | 28.40 | 32.44 | 27.52 |
|  | VIT_208s0040g00900 | 8.31 | 7.88 | 7.96 | 3.98 | 4.90 | 5.46 | 5.89 | 6.49 | 5.35 | 5.67 | 5.96 | 5.88 | 5.54 | 5.68 |
|  | VIT_208s0058g00240 | 0.06 | 0.00 | 0.00 | 0.00 | 0.00 | 0.00 | 0.00 | 0.00 | 0.00 | 0.00 | 0.00 | 0.00 | 0.00 | 0.00 |
|  | VIT_208s0105g00370 | 0.05 | 0.10 | 0.29 | 0.80 | 0.38 | 1.46 | 0.61 | 0.53 | 1.23 | 1.32 | 1.24 | 1.54 | 1.62 | 1.29 |
|  | VIT_209s0054g01620 | 11.30 | 13.56 | 12.67 | 8.23 | 7.59 | 4.69 | 5.42 | 8.80 | 14.39 | 10.54 | 13.86 | 26.49 | 19.90 | 21.93 |
|  | VIT_209s0070g00405 | 0.00 | 0.04 | 0.00 | 0.00 | 0.00 | 0.00 | 0.00 | 0.03 | 0.03 | 0.00 | 0.02 | 0.02 | 0.03 | 0.00 |
|  | VIT_210s0003g01380 | 0.74 | 0.24 | 0.90 | 0.32 | 0.36 | 0.51 | 0.52 | 0.53 | 0.66 | 0.40 | 0.68 | 1.08 | 0.71 | 0.33 |
|  | VIT_211s0206g00060 | 5.87 | 6.81 | 8.93 | 8.22 | 7.08 | 17.39 | 14.08 | 13.54 | 22.06 | 18.99 | 21.97 | 28.29 | 27.52 | 31.09 |
|  | VIT_212s0028g00980 | 0.57 | 1.62 | 0.46 | 2.06 | 1.83 | 0.84 | 1.21 | 2.38 | 7.76 | 6.76 | 8.63 | 9.94 | 10.85 | 7.64 |
|  | VIT_212s0028g03100 | 15.83 | 6.49 | 4.03 | 1.10 | 1.00 | 0.38 | 0.34 | 0.38 | 0.16 | 0.07 | 0.13 | 0.12 | 0.04 | 0.04 |
|  | VIT_213s0067g01500 | 0.00 | 0.00 | 0.33 | 0.00 | 0.00 | 0.00 | 0.03 | 0.10 | 0.03 | 0.00 | 0.13 | 0.00 | 0.03 | 0.00 |
|  | VIT_213s0156g00370 | 0.13 | 0.09 | 0.30 | 0.06 | 0.03 | 0.00 | 0.00 | 0.03 | 0.03 | 0.00 | 0.00 | 0.03 | 0.00 | 0.02 |
|  | VIT_214s0036g01380 | 3.06 | 3.91 | 4.74 | 4.61 | 4.27 | 3.36 | 3.82 | 3.59 | 5.19 | 3.93 | 4.26 | 5.34 | 4.02 | 4.42 |
|  | VIT_215s0048g01025 | 1.70 | 0.42 | 3.95 | 0.00 | 0.00 | 0.00 | 0.00 | 0.15 | 0.00 | 0.00 | 0.00 | 0.00 | 0.00 | 0.00 |
|  | VIT_216s0039g01900 | 20.10 | 12.74 | 17.55 | 13.40 | 11.28 | 16.61 | 15.42 | 22.62 | 33.47 | 39.54 | 35.82 | 46.87 | 42.86 | 35.46 |
|  | VIT_216s0100g00420 | 0.44 | 0.24 | 0.70 | 0.00 | 0.00 | 0.00 | 0.00 | 0.00 | 0.00 | 0.00 | 0.00 | 0.00 | 0.00 | 0.04 |
|  | VIT_217s0000g10100 | 2.58 | 3.89 | 4.34 | 4.82 | 4.45 | 5.42 | 4.13 | 5.21 | 5.65 | 4.99 | 5.86 | 11.41 | 11.48 | 9.50 |
|  | VIT_217s0000g10110 | 0.97 | 1.01 | 1.38 | 0.68 | 0.66 | 0.92 | 0.89 | 0.88 | 0.95 | 1.00 | 1.06 | 0.80 | 0.73 | 0.55 |
|  | VIT_217s0000g10120 | 0.00 | 0.00 | 0.52 | 0.00 | 0.25 | 0.51 | 0.26 | 0.26 | 0.26 | 0.53 | 0.26 | 0.25 | 0.53 | 0.27 |
|  | VIT_219s0085g00890 | 6.21 | 2.78 | 5.17 | 3.95 | 3.74 | 1.65 | 2.41 | 2.89 | 2.05 | 2.06 | 1.95 | 3.57 | 2.98 | 3.49 |
| A-ARR | VIT_201s0026g00940 | 14.60 | 2.93 | 3.59 | 5.97 | 4.84 | 16.39 | 15.24 | 12.05 | 34.82 | 33.77 | 24.03 | 14.04 | 13.98 | 11.80 |
|  | VIT_208s0007g05390 | 12.15 | 6.99 | 6.50 | 9.50 | 8.75 | 6.09 | 6.06 | 5.72 | 16.70 | 12.26 | 9.68 | 14.24 | 14.58 | 14.59 |
|  | VIT_213s0067g03070 | 14.57 | 20.69 | 8.49 | 23.30 | 37.44 | 20.62 | 19.57 | 15.29 | 9.75 | 11.09 | 11.38 | 6.82 | 6.18 | 6.61 |
|  | VIT_213s0067g03430 | 38.36 | 10.43 | 8.56 | 22.11 | 24.07 | 23.24 | 23.50 | 19.56 | 32.13 | 30.26 | 23.76 | 9.93 | 13.09 | 10.18 |
|  | VIT_213s0067g03450 | 0.00 | 0.00 | 0.00 | 0.00 | 0.00 | 0.00 | 0.00 | 0.00 | 0.00 | 0.12 | 0.00 | 0.00 | 0.00 | 0.00 |
|  | VIT_213s0067g03490 | 8.78 | 6.08 | 4.69 | 3.15 | 2.53 | 2.29 | 3.54 | 2.65 | 5.83 | 3.32 | 4.32 | 2.54 | 1.53 | 4.12 |
|  | VIT_213s0067g03510 | 4.46 | 1.12 | 0.81 | 0.49 | 0.19 | 3.57 | 1.45 | 2.14 | 12.12 | 7.58 | 5.51 | 8.90 | 8.57 | 10.04 |
|  | VIT_217s0000g07580 | 25.77 | 4.36 | 6.06 | 2.39 | 2.84 | 2.76 | 2.47 | 2.84 | 4.96 | 3.25 | 2.82 | 0.68 | 1.34 | 1.22 |
|  | VIT_218s0001g02540 | 25.27 | 0.41 | 0.48 | 0.54 | 0.30 | 0.30 | 0.34 | 0.20 | 0.18 | 0.11 | 0.06 | 0.06 | 0.06 | 0.06 |
| GID1 | VIT_200s1008g00020 | 0.99 | 0.00 | 0.18 | 0.16 | 0.00 | 0.11 | 0.00 | 0.12 | 0.36 | 0.04 | 0.47 | 0.17 | 1.15 | 0.31 |
|  | VIT_201s0010g02260 | 19.09 | 23.74 | 20.24 | 25.30 | 29.94 | 29.42 | 32.04 | 30.32 | 22.53 | 23.65 | 28.73 | 30.57 | 29.32 | 31.39 |
|  | VIT_201s0011g03270 | 0.00 | 0.00 | 0.17 | 0.00 | 0.00 | 0.00 | 0.00 | 0.12 | 0.00 | 0.06 | 0.00 | 0.00 | 0.00 | 0.00 |
|  | VIT_204s0008g04590 | 0.00 | 0.00 | 0.60 | 0.67 | 0.25 | 0.49 | 0.11 | 0.11 | 0.37 | 0.41 | 0.49 | 0.12 | 0.07 | 0.00 |
|  | VIT_204s0008g05340 | 8.02 | 8.60 | 2.96 | 2.90 | 2.13 | 1.96 | 1.32 | 1.33 | 2.08 | 1.98 | 2.04 | 1.29 | 0.97 | 1.31 |
|  | VIT_204s0008g05350 | 0.61 | 0.52 | 0.44 | 0.19 | 0.00 | 0.18 | 0.00 | 0.00 | 0.29 | 0.13 | 0.07 | 0.06 | 0.13 | 0.07 |
|  | VIT_204s0008g05360 | 3.97 | 5.80 | 4.93 | 4.12 | 5.01 | 4.02 | 3.94 | 5.37 | 7.44 | 5.80 | 5.27 | 4.36 | 4.40 | 4.78 |
|  | VIT_204s0008g05380 | 6.18 | 14.33 | 10.87 | 12.93 | 18.39 | 7.77 | 9.73 | 9.22 | 8.57 | 7.44 | 6.89 | 6.68 | 5.28 | 5.98 |
|  | VIT_204s0008g05390 | 0.07 | 0.37 | 0.14 | 0.00 | 0.22 | 0.19 | 0.00 | 0.59 | 0.41 | 0.46 | 0.48 | 0.42 | 0.18 | 0.30 |
|  | VIT_204s0008g05400 | 0.05 | 0.49 | 0.48 | 0.48 | 0.52 | 0.05 | 0.10 | 0.16 | 0.21 | 0.16 | 0.11 | 0.22 | 0.11 | 0.00 |
|  | VIT_204s0008g05410 | 4.62 | 20.66 | 14.72 | 6.97 | 12.36 | 2.89 | 5.25 | 6.25 | 3.14 | 2.50 | 4.73 | 3.18 | 2.47 | 3.28 |
|  | VIT_206s0009g00980 | 2.54 | 12.82 | 11.92 | 9.17 | 3.34 | 5.77 | 7.00 | 6.03 | 3.58 | 3.79 | 2.73 | 1.55 | 1.55 | 1.76 |
|  | VIT_207s0104g00930 | 27.09 | 159.62 | 133.47 | 78.39 | 87.61 | 84.62 | 99.51 | 88.23 | 110.24 | 104.33 | 102.55 | 85.80 | 92.38 | 87.73 |
|  | VIT_207s0130g00320 | 23.87 | 25.77 | 22.72 | 14.12 | 18.61 | 18.94 | 22.50 | 21.43 | 18.50 | 19.10 | 19.46 | 13.24 | 13.31 | 11.33 |
|  | VIT_207s0130g00340 | 5.98 | 14.01 | 9.19 | 3.44 | 8.59 | 5.12 | 7.89 | 6.89 | 2.80 | 5.18 | 4.52 | 3.13 | 3.13 | 2.64 |
|  | VIT_208s0032g00040 | 0.11 | 0.00 | 0.00 | 0.00 | 0.00 | 0.00 | 0.00 | 0.00 | 0.00 | 0.00 | 0.00 | 0.00 | 0.00 | 0.00 |
|  | VIT_208s0032g00510 | 0.14 | 0.05 | 0.19 | 0.05 | 0.00 | 0.00 | 0.00 | 0.05 | 0.00 | 0.00 | 0.00 | 0.00 | 0.00 | 0.00 |
|  | VIT_208s0032g00700 | 0.98 | 0.00 | 0.15 | 0.00 | 0.05 | 0.00 | 0.05 | 0.05 | 0.00 | 0.05 | 0.10 | 0.00 | 0.05 | 0.00 |
|  | VIT_208s0032g00710 | 0.00 | 0.13 | 0.13 | 0.00 | 0.04 | 0.00 | 0.00 | 0.09 | 0.00 | 0.00 | 0.00 | 0.00 | 0.00 | 0.00 |
|  | VIT_208s0040g00810 | 0.33 | 0.00 | 0.00 | 0.00 | 0.00 | 0.00 | 0.00 | 0.00 | 0.00 | 0.00 | 0.00 | 0.00 | 0.00 | 0.00 |
|  | VIT_208s0040g00960 | 0.00 | 0.38 | 0.90 | 0.00 | 0.00 | 0.00 | 0.00 | 0.00 | 0.11 | 0.05 | 0.00 | 0.27 | 0.05 | 0.06 |
|  | VIT_213s0084g00130 | 42.90 | 23.56 | 25.09 | 3.50 | 1.10 | 0.05 | 0.54 | 1.97 | 1.29 | 0.15 | 2.81 | 0.42 | 0.05 | 0.37 |
|  | VIT_214s0006g03180 | 0.12 | 0.12 | 0.30 | 0.00 | 0.00 | 0.00 | 0.00 | 0.24 | 0.11 | 0.18 | 0.18 | 0.06 | 0.06 | 0.00 |
|  | VIT_214s0030g00440 | 22.15 | 31.07 | 32.85 | 31.37 | 28.35 | 38.13 | 33.35 | 37.80 | 40.15 | 37.77 | 38.16 | 40.98 | 40.54 | 40.63 |
|  | VIT_214s0219g00020 | 16.00 | 15.04 | 15.61 | 10.83 | 15.15 | 11.69 | 11.79 | 11.93 | 13.31 | 12.06 | 11.68 | 11.52 | 11.71 | 11.54 |
|  | VIT_215s0048g01345 | 0.00 | 0.00 | 0.00 | 0.00 | 0.00 | 0.00 | 0.00 | 0.00 | 0.06 | 0.00 | 0.00 | 0.00 | 0.00 | 0.00 |
|  | VIT_215s0048g01350 | 4.85 | 1.37 | 2.32 | 0.35 | 0.17 | 0.38 | 0.60 | 0.74 | 1.30 | 2.14 | 2.13 | 0.84 | 0.10 | 0.95 |
|  | VIT_215s0048g01365 | 3.87 | 2.27 | 3.65 | 0.14 | 0.17 | 0.29 | 0.21 | 1.16 | 0.52 | 0.59 | 0.97 | 0.89 | 0.37 | 0.64 |
|  | VIT_215s0048g01390 | 88.94 | 14.47 | 22.12 | 2.94 | 3.36 | 1.19 | 1.98 | 3.47 | 2.84 | 1.94 | 2.10 | 2.41 | 1.25 | 1.63 |
|  | VIT_216s0022g02300 | 0.07 | 0.00 | 0.00 | 0.00 | 0.00 | 0.00 | 0.00 | 0.00 | 0.00 | 0.00 | 0.00 | 0.00 | 0.00 | 0.00 |
|  | VIT_216s0022g02430 | 0.25 | 0.33 | 0.53 | 0.08 | 0.08 | 0.00 | 0.00 | 0.08 | 0.25 | 0.08 | 0.29 | 0.33 | 0.50 | 1.23 |
|  | VIT_216s0022g02440 | 0.80 | 1.49 | 2.47 | 0.30 | 0.18 | 0.18 | 0.18 | 2.13 | 1.18 | 0.99 | 2.70 | 3.45 | 3.49 | 6.07 |
|  | VIT_216s0100g00010 | 0.37 | 0.81 | 0.42 | 0.12 | 0.00 | 0.00 | 0.00 | 0.82 | 0.24 | 0.29 | 0.56 | 1.03 | 0.80 | 1.99 |
|  | VIT_218s0089g00370 | 2.80 | 2.69 | 3.28 | 1.30 | 1.03 | 2.00 | 1.82 | 2.44 | 4.85 | 4.91 | 5.45 | 8.49 | 7.51 | 6.62 |
|  | VIT_218s0089g00380 | 0.00 | 0.18 | 0.06 | 0.00 | 0.00 | 0.00 | 0.11 | 0.00 | 0.30 | 0.06 | 0.12 | 0.00 | 0.00 | 0.06 |
|  | VIT_218s0089g00390 | 0.19 | 0.06 | 0.19 | 0.00 | 0.06 | 0.00 | 0.00 | 0.00 | 0.00 | 0.00 | 0.06 | 0.00 | 0.00 | 0.00 |
|  | VIT_218s0089g00400 | 6.12 | 8.96 | 2.48 | 2.20 | 1.38 | 1.60 | 1.27 | 0.90 | 1.08 | 0.50 | 0.74 | 0.17 | 0.17 | 0.23 |
|  | VIT_207s0104g00130 | 3.35 | 3.07 | 3.87 | 1.47 | 1.13 | 1.03 | 1.25 | 1.57 | 2.38 | 1.75 | 1.91 | 1.42 | 0.93 | 1.47 |
|  | VIT_207s0129g01000 | 10.64 | 8.56 | 7.21 | 1.57 | 2.68 | 1.66 | 2.52 | 2.58 | 2.37 | 2.00 | 2.12 | 2.85 | 2.36 | 2.00 |
|  | VIT_212s0057g00720 | 0.00 | 0.00 | 0.00 | 0.00 | 0.00 | 0.00 | 0.00 | 0.00 | 0.08 | 0.00 | 0.08 | 0.00 | 0.00 | 0.00 |
|  | VIT_218s0001g09700 | 87.75 | 186.40 | 130.21 | 164.93 | 156.67 | 206.24 | 225.13 | 212.76 | 149.21 | 167.54 | 151.08 | 122.58 | 119.29 | 109.31 |
| DELLA | VIT_200s0226g00070 | 2.23 | 0.28 | 0.37 | 0.05 | 0.14 | 0.09 | 0.46 | 0.55 | 0.28 | 0.32 | 1.57 | 0.60 | 0.47 | 0.62 |
|  | VIT_200s0463g00020 | 81.96 | 28.27 | 30.48 | 38.98 | 38.21 | 32.69 | 29.52 | 35.10 | 24.76 | 33.40 | 33.19 | 29.20 | 28.74 | 30.51 |
|  | VIT_201s0010g02270 | 0.06 | 0.42 | 0.25 | 0.17 | 0.16 | 0.03 | 0.03 | 0.17 | 0.08 | 0.00 | 0.06 | 0.08 | 0.08 | 0.03 |
|  | VIT_201s0011g05260 | 8.05 | 11.69 | 13.61 | 7.87 | 7.82 | 7.02 | 8.52 | 21.38 | 11.72 | 13.03 | 23.51 | 12.15 | 12.05 | 15.43 |
|  | VIT_202s0025g04000 | 0.69 | 0.02 | 0.05 | 0.02 | 0.12 | 0.02 | 0.05 | 0.00 | 0.00 | 0.02 | 0.00 | 0.05 | 0.05 | 0.05 |
|  | VIT_202s0033g00050 | 0.00 | 0.00 | 0.00 | 0.00 | 0.00 | 0.24 | 0.23 | 0.48 | 0.22 | 0.00 | 0.21 | 0.00 | 0.00 | 0.62 |
|  | VIT_202s0154g00400 | 43.87 | 36.58 | 37.72 | 32.08 | 23.82 | 26.36 | 28.00 | 29.69 | 28.53 | 32.31 | 27.39 | 34.95 | 34.75 | 29.86 |
|  | VIT_203s0017g01660 | 8.15 | 12.15 | 8.84 | 2.95 | 3.22 | 3.25 | 2.92 | 3.51 | 4.40 | 4.78 | 3.17 | 2.56 | 2.51 | 2.18 |
|  | VIT_204s0023g01380 | 16.47 | 9.20 | 12.91 | 29.39 | 13.36 | 22.50 | 26.16 | 25.29 | 51.05 | 52.44 | 41.23 | 80.26 | 87.71 | 69.46 |
|  | VIT_204s0023g01660 | 30.99 | 11.14 | 8.61 | 7.51 | 11.11 | 4.39 | 6.62 | 8.99 | 2.24 | 3.19 | 3.57 | 1.28 | 1.17 | 1.68 |
|  | VIT_204s0044g00620 | 4.02 | 0.46 | 1.14 | 0.00 | 0.00 | 0.00 | 0.05 | 0.05 | 0.00 | 0.00 | 0.00 | 0.00 | 0.00 | 0.00 |
|  | VIT_204s0044g01370 | 0.33 | 0.37 | 0.31 | 0.36 | 0.39 | 0.24 | 0.20 | 0.27 | 0.19 | 0.27 | 0.19 | 0.13 | 0.15 | 0.30 |
|  | VIT_205s0077g01120 | 0.00 | 0.00 | 0.00 | 0.00 | 0.00 | 0.00 | 0.00 | 0.00 | 0.00 | 0.04 | 0.00 | 0.00 | 0.00 | 0.04 |
|  | VIT_206s0004g04950 | 23.84 | 38.60 | 47.42 | 14.19 | 10.91 | 19.43 | 13.06 | 17.38 | 34.63 | 33.86 | 33.54 | 24.17 | 23.03 | 26.35 |
|  | VIT_206s0004g04960 | 17.13 | 23.18 | 27.89 | 12.03 | 14.11 | 11.45 | 11.79 | 11.34 | 6.97 | 9.31 | 11.80 | 3.33 | 3.09 | 4.14 |
|  | VIT_206s0004g04970 | 0.05 | 0.15 | 0.12 | 0.03 | 0.00 | 0.10 | 0.05 | 0.03 | 0.41 | 0.00 | 0.02 | 0.00 | 0.00 | 0.03 |
|  | VIT_206s0004g04980 | 20.63 | 36.76 | 36.54 | 13.87 | 12.61 | 14.77 | 13.39 | 15.91 | 17.79 | 17.01 | 19.32 | 17.09 | 13.27 | 17.30 |
|  | VIT_206s0004g04990 | 0.00 | 0.37 | 0.09 | 0.00 | 0.09 | 0.00 | 0.00 | 0.14 | 0.05 | 0.19 | 0.14 | 0.09 | 0.19 | 0.00 |
|  | VIT_206s0009g02390 | 5.81 | 2.83 | 4.13 | 3.50 | 4.17 | 13.46 | 10.29 | 11.96 | 10.37 | 12.93 | 11.00 | 9.03 | 8.86 | 8.25 |
|  | VIT_207s0005g01500 | 0.00 | 0.02 | 0.02 | 0.02 | 0.00 | 0.00 | 0.00 | 0.02 | 0.20 | 0.00 | 0.05 | 0.00 | 0.02 | 0.00 |
|  | VIT_207s0005g03700 | 0.00 | 0.08 | 0.17 | 0.00 | 0.00 | 0.00 | 0.00 | 0.00 | 0.00 | 0.00 | 0.00 | 0.08 | 0.00 | 0.00 |
|  | VIT_207s0129g00030 | 6.30 | 2.01 | 3.06 | 2.81 | 2.71 | 2.07 | 2.18 | 2.52 | 1.75 | 2.65 | 1.49 | 1.39 | 1.31 | 1.15 |
|  | VIT_207s0129g00340 | 0.00 | 0.00 | 0.04 | 0.04 | 0.00 | 0.00 | 0.00 | 0.00 | 0.00 | 0.00 | 0.00 | 0.00 | 0.00 | 0.00 |
|  | VIT_208s0007g00510 | 43.76 | 61.75 | 74.88 | 45.21 | 41.86 | 34.71 | 37.69 | 34.59 | 20.54 | 25.32 | 25.85 | 14.58 | 14.61 | 15.57 |
|  | VIT_208s0056g00050 | 0.15 | 0.00 | 0.08 | 0.02 | 0.00 | 0.02 | 0.00 | 0.02 | 0.00 | 0.02 | 0.02 | 0.00 | 0.00 | 0.00 |
|  | VIT_208s0058g00295 | 0.03 | 0.00 | 0.03 | 0.04 | 0.00 | 0.00 | 0.03 | 0.03 | 0.03 | 0.00 | 0.04 | 0.28 | 0.07 | 0.14 |
|  | VIT_208s0058g00305 | 0.05 | 0.00 | 0.00 | 0.00 | 0.00 | 0.00 | 0.00 | 0.00 | 0.05 | 0.05 | 0.05 | 0.15 | 0.25 | 0.05 |
|  | VIT_208s0058g00310 | 0.12 | 0.12 | 0.04 | 0.00 | 0.00 | 0.00 | 0.12 | 0.20 | 0.24 | 0.16 | 0.25 | 0.76 | 0.32 | 0.75 |
|  | VIT_209s0002g01190 | 1.01 | 3.61 | 3.19 | 1.44 | 1.04 | 3.18 | 1.89 | 2.26 | 5.55 | 4.60 | 3.78 | 7.10 | 6.12 | 5.37 |
|  | VIT_211s0016g04630 | 63.56 | 80.09 | 71.31 | 44.27 | 49.65 | 40.04 | 47.98 | 40.53 | 31.52 | 43.69 | 37.75 | 14.78 | 16.25 | 16.63 |
|  | VIT_212s0057g00560 | 13.96 | 5.97 | 11.34 | 7.06 | 5.70 | 18.86 | 17.47 | 21.97 | 14.92 | 18.28 | 14.53 | 21.05 | 19.20 | 17.86 |
|  | VIT_212s0059g02770 | 0.06 | 0.00 | 0.03 | 0.06 | 0.00 | 0.56 | 0.22 | 0.42 | 1.23 | 0.73 | 1.20 | 0.64 | 0.60 | 0.70 |
|  | VIT_213s0019g01220 | 10.87 | 4.80 | 7.04 | 1.38 | 1.73 | 7.83 | 6.86 | 8.87 | 9.03 | 5.15 | 6.51 | 6.65 | 6.25 | 7.42 |
|  | VIT_213s0019g01700 | 0.23 | 0.02 | 0.33 | 0.00 | 0.02 | 0.11 | 0.02 | 0.09 | 0.33 | 0.32 | 0.28 | 0.37 | 0.38 | 0.39 |
|  | VIT_213s0019g01710 | 0.77 | 2.93 | 2.02 | 0.34 | 0.26 | 0.21 | 0.34 | 0.47 | 1.42 | 1.08 | 0.69 | 0.64 | 0.74 | 0.61 |
|  | VIT_213s0019g01780 | 0.03 | 0.00 | 0.10 | 0.00 | 0.00 | 0.00 | 0.03 | 0.00 | 0.00 | 0.03 | 0.00 | 0.00 | 0.00 | 0.04 |
|  | VIT_213s0019g01790 | 0.00 | 0.00 | 0.00 | 0.00 | 0.00 | 0.00 | 0.00 | 0.00 | 0.03 | 0.03 | 0.00 | 0.00 | 0.00 | 0.00 |
|  | VIT_213s0019g01810 | 4.28 | 4.38 | 5.62 | 3.70 | 3.05 | 3.13 | 3.23 | 4.37 | 6.51 | 5.04 | 7.38 | 10.45 | 9.53 | 10.57 |
|  | VIT_214s0006g00640 | 41.26 | 42.67 | 42.20 | 29.56 | 28.93 | 24.95 | 28.75 | 42.11 | 36.23 | 32.12 | 46.46 | 35.52 | 31.93 | 35.52 |
|  | VIT_214s0068g01610 | 0.00 | 0.00 | 0.00 | 0.00 | 0.00 | 0.00 | 0.00 | 0.00 | 0.02 | 0.00 | 0.04 | 0.03 | 0.00 | 0.00 |
|  | VIT_215s0048g00270 | 20.93 | 10.54 | 12.75 | 16.32 | 8.62 | 20.90 | 22.18 | 21.50 | 28.10 | 30.77 | 25.10 | 21.92 | 22.40 | 21.95 |
|  | VIT_216s0050g00950 | 104.37 | 73.60 | 78.56 | 134.13 | 143.95 | 146.39 | 149.60 | 161.17 | 167.78 | 190.30 | 181.45 | 245.69 | 240.26 | 232.40 |
|  | VIT_218s0001g03310 | 0.04 | 0.12 | 0.12 | 0.04 | 0.04 | 0.00 | 0.00 | 0.04 | 0.00 | 0.00 | 0.04 | 0.00 | 0.00 | 0.00 |
|  | VIT_218s0001g15270 | 48.50 | 28.39 | 36.42 | 40.46 | 33.95 | 42.54 | 44.21 | 42.10 | 37.09 | 40.67 | 41.36 | 38.85 | 32.23 | 37.40 |
|  | VIT_218s0072g00685 | 0.00 | 0.00 | 0.18 | 0.18 | 0.35 | 0.00 | 0.37 | 0.00 | 0.19 | 0.00 | 0.00 | 1.29 | 0.92 | 0.58 |
|  | VIT_218s0076g00200 | 0.06 | 0.06 | 0.26 | 0.06 | 0.00 | 0.12 | 0.06 | 0.00 | 0.00 | 0.06 | 0.26 | 0.13 | 0.06 | 0.00 |
|  | VIT_219s0014g04940 | 74.86 | 101.57 | 136.61 | 47.53 | 46.19 | 41.30 | 39.66 | 48.30 | 34.42 | 42.17 | 56.74 | 34.64 | 32.80 | 40.33 |
|  | VIT_219s0085g00540 | 0.13 | 0.51 | 0.56 | 0.00 | 0.03 | 0.03 | 0.00 | 0.12 | 0.00 | 0.03 | 0.03 | 0.03 | 0.03 | 0.00 |
|  | VIT_219s0090g01740 | 199.99 | 249.30 | 290.98 | 104.91 | 146.32 | 77.45 | 95.71 | 89.67 | 43.13 | 43.57 | 69.77 | 46.60 | 44.50 | 43.24 |
| PIF3 | VIT_201s0244g00010 | 83.93 | 305.06 | 167.12 | 365.05 | 319.89 | 375.45 | 333.39 | 266.96 | 471.31 | 443.11 | 373.78 | 266.26 | 237.72 | 249.92 |
|  | VIT_201s0010g00740 | 7.55 | 2.15 | 3.26 | 2.41 | 2.53 | 2.17 | 2.10 | 2.97 | 3.23 | 2.44 | 3.45 | 3.98 | 3.89 | 4.04 |
|  | VIT_218s0001g08600 | 3.49 | 1.53 | 2.52 | 1.83 | 1.38 | 2.16 | 2.32 | 2.09 | 3.61 | 2.50 | 2.48 | 1.42 | 1.05 | 1.67 |
|  | VIT_215s0021g02690 | 5.16 | 3.82 | 1.02 | 0.83 | 1.34 | 1.70 | 0.22 | 13.20 | 5.00 | 1.54 | 15.40 | 1.85 | 1.52 | 4.50 |
|  | VIT_217s0000g03580 | 1.97 | 0.71 | 0.27 | 0.00 | 0.11 | 0.00 | 0.00 | 0.00 | 0.05 | 0.00 | 0.00 | 0.00 | 0.00 | 0.06 |
|  | VIT_203s0038g04760 | 10.19 | 2.24 | 2.27 | 1.98 | 1.44 | 1.45 | 1.60 | 1.41 | 2.25 | 1.70 | 1.44 | 1.40 | 1.80 | 0.99 |
|  | VIT_207s0151g00450 | 4.25 | 0.13 | 0.65 | 0.21 | 0.03 | 0.10 | 0.03 | 0.00 | 0.10 | 0.34 | 0.04 | 0.03 | 0.03 | 0.07 |
|  | VIT_211s0016g00380 | 13.92 | 10.06 | 13.16 | 10.24 | 9.94 | 7.97 | 9.02 | 12.25 | 14.75 | 12.27 | 16.58 | 20.78 | 20.84 | 19.10 |
|  | VIT_218s0001g10270 | 1.20 | 0.50 | 1.80 | 0.59 | 0.50 | 0.56 | 0.47 | 0.64 | 1.27 | 0.94 | 1.08 | 1.04 | 0.70 | 0.64 |
|  | VIT_205s0020g04620 | 13.38 | 16.58 | 18.07 | 8.00 | 6.98 | 8.81 | 8.59 | 10.29 | 14.63 | 13.26 | 12.37 | 20.00 | 18.88 | 18.06 |
|  | VIT_204s0023g01930 | 0.95 | 1.56 | 1.10 | 2.44 | 2.02 | 0.84 | 2.10 | 2.82 | 1.96 | 1.30 | 2.98 | 0.82 | 0.98 | 1.37 |
|  | VIT_207s0031g00450 | 15.29 | 10.93 | 10.89 | 5.15 | 7.79 | 2.59 | 3.99 | 4.54 | 5.56 | 4.17 | 4.01 | 7.31 | 6.48 | 5.62 |
|  | VIT_214s0060g00260 | 12.89 | 7.83 | 8.35 | 2.50 | 2.79 | 2.81 | 2.90 | 3.67 | 3.21 | 2.64 | 2.31 | 1.53 | 1.50 | 1.46 |
|  | VIT_217s0000g00430 | 11.63 | 263.97 | 122.73 | 1059.85 | 887.12 | 1079.08 | 1083.65 | 938.08 | 604.18 | 774.98 | 561.33 | 459.84 | 497.86 | 464.05 |
|  | VIT_217s0000g06930 | 0.35 | 0.12 | 0.87 | 0.10 | 0.05 | 0.28 | 0.10 | 0.25 | 1.19 | 0.23 | 0.71 | 1.72 | 0.32 | 1.51 |
|  | VIT_207s0005g02510 | 1.05 | 0.19 | 0.80 | 0.10 | 0.21 | 0.19 | 0.09 | 0.47 | 0.12 | 0.09 | 0.12 | 0.14 | 0.05 | 0.10 |
|  | VIT_206s0004g01740 | 1.48 | 0.41 | 1.36 | 0.79 | 0.40 | 0.30 | 0.38 | 0.97 | 1.66 | 1.06 | 1.23 | 1.44 | 1.39 | 1.92 |
|  | VIT_205s0029g00390 | 12.91 | 9.64 | 10.32 | 15.30 | 17.01 | 12.59 | 16.97 | 14.51 | 10.23 | 12.32 | 10.61 | 3.99 | 2.95 | 3.45 |
|  | VIT_213s0064g01290 | 4.47 | 4.08 | 3.79 | 0.78 | 1.00 | 0.85 | 1.12 | 1.84 | 0.86 | 0.99 | 0.68 | 0.43 | 0.26 | 0.47 |
|  | VIT_201s0011g03720 | 0.56 | 0.11 | 0.10 | 0.27 | 0.15 | 0.21 | 0.31 | 0.10 | 1.10 | 0.26 | 1.02 | 3.69 | 0.80 | 3.26 |
|  | VIT_217s0000g05370 | 13.96 | 21.15 | 15.65 | 7.81 | 11.41 | 5.10 | 5.86 | 7.85 | 3.93 | 3.17 | 4.38 | 2.72 | 2.11 | 2.94 |
|  | VIT_208s0007g07810 | 2.28 | 5.04 | 4.40 | 0.88 | 0.46 | 0.94 | 0.90 | 2.02 | 3.01 | 2.18 | 2.82 | 3.37 | 3.78 | 3.41 |
|  | VIT_201s0026g01140 | 4.74 | 2.32 | 4.37 | 4.55 | 4.45 | 4.75 | 4.83 | 5.43 | 2.54 | 1.97 | 3.43 | 1.76 | 1.61 | 2.06 |
|  | VIT_213s0067g01350 | 6.18 | 15.85 | 1.62 | 0.43 | 0.25 | 0.37 | 0.38 | 0.75 | 1.25 | 0.63 | 0.56 | 1.62 | 1.24 | 1.14 |
|  | VIT_212s0028g01110 | 24.90 | 22.78 | 14.73 | 22.85 | 28.20 | 26.70 | 35.87 | 31.38 | 18.92 | 22.28 | 18.57 | 8.32 | 8.11 | 9.53 |
| PYR/PYL | VIT_201s0011g05090 | 0.00 | 0.07 | 0.07 | 0.00 | 0.13 | 0.06 | 0.00 | 0.26 | 0.07 | 0.00 | 0.00 | 0.00 | 0.00 | 0.00 |
|  | VIT_201s0026g00570 | 44.31 | 0.63 | 0.43 | 2.34 | 1.19 | 0.69 | 0.92 | 0.51 | 0.04 | 0.12 | 0.04 | 0.04 | 0.00 | 0.00 |
|  | VIT_202s0012g01270 | 21.69 | 24.35 | 22.65 | 25.20 | 26.36 | 7.78 | 9.66 | 17.80 | 3.71 | 7.80 | 6.83 | 3.46 | 4.50 | 3.75 |
|  | VIT_202s0025g01340 | 57.31 | 54.46 | 44.54 | 45.13 | 39.89 | 43.98 | 44.54 | 46.07 | 62.49 | 54.01 | 57.73 | 73.98 | 66.71 | 63.01 |
|  | VIT_204s0008g00270 | 0.00 | 0.00 | 0.00 | 0.00 | 0.00 | 0.12 | 0.00 | 0.12 | 0.00 | 0.00 | 0.00 | 0.00 | 0.06 | 0.00 |
|  | VIT_204s0008g00890 | 0.44 | 0.11 | 0.11 | 0.00 | 0.00 | 0.11 | 0.00 | 0.00 | 0.89 | 0.11 | 0.33 | 0.74 | 0.88 | 0.56 |
|  | VIT_205s0077g01550 | 0.00 | 1.31 | 6.11 | 0.00 | 0.00 | 0.00 | 0.16 | 0.00 | 2.10 | 0.15 | 0.00 | 0.94 | 0.70 | 1.06 |
|  | VIT_205s0077g01630 | 0.00 | 0.00 | 0.00 | 0.16 | 0.00 | 0.00 | 0.00 | 0.00 | 0.00 | 0.00 | 0.02 | 0.00 | 0.00 | 0.00 |
|  | VIT_206s0004g04605 | 0.20 | 0.05 | 0.00 | 0.00 | 0.00 | 0.20 | 0.00 | 0.00 | 0.00 | 0.05 | 0.21 | 0.10 | 0.05 | 0.11 |
|  | VIT_206s0004g07385 | 0.25 | 0.25 | 0.25 | 0.18 | 0.49 | 0.16 | 0.08 | 0.16 | 0.24 | 0.24 | 0.35 | 0.16 | 0.33 | 0.43 |
|  | VIT_208s0058g00230 | 0.00 | 0.00 | 0.00 | 0.00 | 0.00 | 0.32 | 0.19 | 0.14 | 0.07 | 0.14 | 0.23 | 0.16 | 0.08 | 0.22 |
|  | VIT_208s0058g00470 | 16.82 | 15.82 | 30.73 | 1.25 | 4.26 | 0.35 | 0.92 | 6.14 | 1.08 | 1.27 | 4.07 | 1.00 | 1.09 | 1.51 |
|  | VIT_210s0003g01335 | 0.00 | 0.22 | 1.56 | 0.00 | 1.40 | 0.21 | 1.16 | 10.42 | 0.45 | 0.44 | 13.84 | 0.43 | 0.22 | 2.03 |
|  | VIT_213s0067g01940 | 1.76 | 0.92 | 0.85 | 0.00 | 0.00 | 0.00 | 0.00 | 0.25 | 0.09 | 0.00 | 0.34 | 0.00 | 0.00 | 0.34 |
|  | VIT_214s0066g00275 | 1.05 | 2.10 | 2.21 | 1.78 | 0.69 | 2.01 | 0.67 | 0.82 | 2.95 | 0.99 | 3.75 | 2.13 | 0.29 | 2.19 |
|  | VIT_216s0050g02620 | 17.34 | 22.66 | 21.63 | 16.51 | 18.75 | 21.79 | 18.83 | 20.35 | 22.37 | 21.47 | 22.00 | 17.12 | 17.14 | 17.16 |
| PP2C | VIT_200s0179g00110 | 5.82 | 3.71 | 4.93 | 3.29 | 3.18 | 3.09 | 3.56 | 3.92 | 4.47 | 3.31 | 4.20 | 4.49 | 5.68 | 5.14 |
|  | VIT_200s0179g00140 | 2.59 | 1.31 | 1.99 | 1.56 | 1.51 | 1.80 | 2.33 | 2.42 | 2.57 | 3.11 | 2.59 | 4.20 | 4.81 | 4.72 |
|  | VIT_200s0434g00020 | 38.66 | 56.62 | 52.52 | 41.87 | 47.89 | 56.18 | 59.03 | 54.27 | 66.89 | 64.79 | 65.57 | 57.73 | 56.34 | 58.58 |
|  | VIT_201s0150g00510 | 13.64 | 6.67 | 6.96 | 2.01 | 0.96 | 0.68 | 0.78 | 0.81 | 2.21 | 1.72 | 2.12 | 1.67 | 0.72 | 0.59 |
|  | VIT_202s0012g01005 | 0.00 | 0.00 | 0.00 | 0.00 | 0.04 | 0.00 | 0.00 | 0.00 | 0.00 | 0.04 | 0.00 | 0.00 | 0.00 | 0.08 |
|  | VIT_202s0025g01390 | 0.16 | 0.90 | 6.84 | 0.08 | 1.41 | 0.24 | 0.24 | 8.37 | 1.12 | 0.90 | 6.14 | 0.54 | 2.34 | 3.53 |
|  | VIT_202s0025g01550 | 0.06 | 0.06 | 0.12 | 0.00 | 0.00 | 0.00 | 0.00 | 0.81 | 0.00 | 0.00 | 0.58 | 0.12 | 0.00 | 0.13 |
|  | VIT_203s0038g02650 | 26.22 | 33.76 | 27.33 | 29.36 | 31.32 | 41.99 | 42.05 | 38.65 | 36.76 | 42.04 | 35.55 | 15.31 | 16.23 | 17.91 |
|  | VIT_204s0008g01420 | 24.27 | 13.80 | 18.89 | 11.89 | 12.50 | 12.15 | 13.03 | 15.01 | 15.12 | 15.34 | 14.47 | 15.81 | 15.99 | 17.15 |
|  | VIT_205s0077g00830 | 28.40 | 21.00 | 21.96 | 11.75 | 12.60 | 14.59 | 14.09 | 14.76 | 9.53 | 10.15 | 10.09 | 5.42 | 6.32 | 6.24 |
|  | VIT_206s0004g05460 | 22.72 | 18.96 | 19.36 | 49.77 | 54.81 | 42.46 | 44.83 | 52.74 | 38.49 | 41.59 | 44.39 | 42.23 | 42.36 | 39.83 |
|  | VIT_208s0058g00660 | 6.36 | 0.30 | 0.39 | 0.09 | 0.03 | 0.03 | 0.03 | 0.03 | 0.13 | 0.10 | 0.10 | 0.03 | 0.00 | 0.04 |
|  | VIT_209s0002g01850 | 18.50 | 18.40 | 18.89 | 18.70 | 18.57 | 18.75 | 17.63 | 22.43 | 23.81 | 19.50 | 25.19 | 24.92 | 26.12 | 27.68 |
|  | VIT_209s0002g03530 | 4.00 | 3.67 | 11.41 | 2.82 | 2.05 | 3.22 | 3.27 | 5.17 | 5.03 | 5.19 | 5.61 | 4.51 | 4.02 | 4.31 |
|  | VIT_209s0002g03600 | 1.43 | 1.68 | 5.82 | 1.77 | 1.26 | 1.91 | 1.32 | 1.53 | 2.80 | 2.27 | 3.48 | 1.53 | 2.96 | 2.20 |
|  | VIT_211s0016g01780 | 5.61 | 6.98 | 7.04 | 4.83 | 4.98 | 5.65 | 5.42 | 5.57 | 6.70 | 6.07 | 7.35 | 6.34 | 6.30 | 7.11 |
|  | VIT_211s0016g03180 | 22.65 | 22.22 | 19.63 | 32.73 | 41.11 | 18.53 | 24.86 | 23.27 | 15.52 | 16.53 | 15.40 | 21.72 | 19.87 | 19.31 |
|  | VIT_213s0019g02200 | 42.92 | 65.19 | 56.77 | 66.87 | 84.52 | 58.09 | 62.05 | 68.58 | 68.68 | 72.25 | 64.55 | 72.64 | 70.70 | 66.58 |
|  | VIT_214s0060g01580 | 108.58 | 71.48 | 51.63 | 81.10 | 90.85 | 43.44 | 52.02 | 45.75 | 35.82 | 32.68 | 34.32 | 24.12 | 21.46 | 22.45 |
|  | VIT_216s0022g02210 | 0.26 | 0.81 | 7.28 | 0.05 | 0.13 | 0.00 | 0.04 | 1.53 | 0.05 | 0.00 | 0.28 | 0.00 | 0.00 | 0.13 |
|  | VIT_216s0050g02680 | 1.75 | 0.45 | 1.06 | 4.07 | 4.39 | 3.14 | 3.95 | 5.25 | 7.46 | 7.50 | 4.41 | 4.26 | 4.37 | 3.53 |
|  | VIT_216s0098g01650 | 12.52 | 7.01 | 2.06 | 4.67 | 2.37 | 3.02 | 2.82 | 3.96 | 4.93 | 3.10 | 3.82 | 3.95 | 3.48 | 4.33 |
|  | VIT_218s0001g10700 | 22.36 | 20.71 | 19.80 | 17.34 | 27.63 | 30.20 | 34.15 | 29.12 | 20.47 | 24.40 | 20.51 | 15.19 | 13.03 | 14.88 |
|  | VIT_218s0001g15050 | 31.60 | 39.45 | 37.43 | 24.93 | 33.07 | 28.14 | 26.47 | 27.79 | 29.81 | 26.22 | 26.61 | 38.52 | 38.22 | 31.30 |
|  | VIT_218s0086g00500 | 0.00 | 0.00 | 0.00 | 0.00 | 0.00 | 0.00 | 0.00 | 0.00 | 0.00 | 0.00 | 0.29 | 0.00 | 0.14 | 0.14 |
| SnRK2 | VIT_200s0710g00020 | 8.80 | 6.60 | 6.21 | 9.40 | 9.55 | 5.82 | 6.64 | 6.87 | 6.21 | 5.11 | 4.86 | 3.37 | 6.12 | 3.95 |
|  | VIT_202s0236g00130 | 37.78 | 21.73 | 17.55 | 32.32 | 38.76 | 15.37 | 17.67 | 20.21 | 26.03 | 23.32 | 23.32 | 33.52 | 35.08 | 30.57 |
|  | VIT_203s0063g01080 | 27.26 | 25.27 | 28.06 | 20.40 | 18.76 | 25.88 | 24.27 | 29.73 | 37.71 | 33.34 | 37.71 | 39.90 | 39.47 | 40.28 |
|  | VIT_207s0031g03210 | 22.02 | 19.61 | 17.31 | 14.33 | 18.29 | 19.57 | 18.33 | 19.04 | 14.02 | 16.30 | 12.08 | 5.77 | 6.22 | 5.36 |
|  | VIT_207s0191g00070 | 10.14 | 6.91 | 4.53 | 10.59 | 9.85 | 5.86 | 8.20 | 4.46 | 5.53 | 4.91 | 5.43 | 5.03 | 3.35 | 3.27 |
|  | VIT_207s0197g00080 | 12.23 | 8.56 | 23.43 | 2.73 | 4.52 | 3.01 | 3.11 | 9.17 | 4.50 | 2.89 | 6.33 | 2.10 | 2.40 | 3.16 |
|  | VIT_212s0035g00310 | 51.72 | 74.01 | 80.12 | 51.08 | 62.04 | 43.92 | 45.58 | 46.13 | 46.68 | 44.39 | 47.65 | 49.91 | 51.39 | 53.44 |
|  | VIT_218s0001g06310 | 29.28 | 44.36 | 36.81 | 26.73 | 30.69 | 18.00 | 18.53 | 22.76 | 16.95 | 17.96 | 19.70 | 15.62 | 15.67 | 16.51 |
| ABF | VIT_200s0144g00230 | 0.00 | 0.17 | 0.11 | 0.02 | 0.03 | 0.21 | 0.00 | 0.34 | 0.00 | 0.00 | 0.00 | 0.00 | 0.21 | 0.00 |
|  | VIT_200s0144g00240 | 0.00 | 0.00 | 1.14 | 0.00 | 0.00 | 0.23 | 0.00 | 0.96 | 0.00 | 0.00 | 0.00 | 0.00 | 0.00 | 0.00 |
|  | VIT_200s0349g00050 | 0.12 | 0.00 | 0.12 | 0.00 | 0.00 | 0.00 | 0.00 | 0.12 | 0.00 | 0.00 | 0.00 | 0.00 | 0.00 | 0.00 |
|  | VIT_203s0063g00310 | 40.65 | 28.35 | 24.80 | 53.91 | 50.34 | 54.66 | 56.58 | 56.07 | 53.43 | 55.53 | 48.55 | 55.51 | 62.19 | 57.74 |
|  | VIT_204s0069g01150 | 9.30 | 5.36 | 5.62 | 4.95 | 4.79 | 2.19 | 2.51 | 6.22 | 7.27 | 5.78 | 6.60 | 5.68 | 5.86 | 5.83 |
|  | VIT_206s0004g08070 | 4.58 | 2.78 | 2.93 | 2.57 | 2.57 | 2.31 | 3.09 | 2.32 | 2.00 | 1.73 | 1.08 | 1.76 | 1.17 | 1.45 |
|  | VIT_206s0009g01790 | 9.41 | 8.05 | 8.01 | 7.76 | 6.77 | 7.86 | 7.38 | 7.55 | 8.57 | 6.56 | 6.10 | 7.45 | 7.57 | 7.72 |
|  | VIT_206s0080g00340 | 1.07 | 1.63 | 17.99 | 0.07 | 0.06 | 0.00 | 0.00 | 0.58 | 0.07 | 0.00 | 0.13 | 0.00 | 0.06 | 0.06 |
|  | VIT_208s0007g03420 | 0.46 | 0.66 | 6.97 | 0.16 | 1.14 | 0.25 | 0.49 | 13.63 | 0.95 | 1.61 | 12.66 | 1.01 | 2.88 | 4.38 |
|  | VIT_212s0028g01380 | 3.05 | 1.86 | 2.38 | 0.87 | 0.34 | 0.86 | 0.86 | 1.65 | 1.68 | 2.35 | 1.31 | 1.57 | 1.59 | 2.17 |
|  | VIT_212s0034g00110 | 6.40 | 3.10 | 3.85 | 2.28 | 1.99 | 1.57 | 2.13 | 2.55 | 3.31 | 2.87 | 3.90 | 3.78 | 2.67 | 3.85 |
|  | VIT_212s0055g00420 | 10.76 | 13.79 | 14.80 | 12.86 | 13.25 | 13.48 | 12.94 | 14.02 | 19.95 | 20.09 | 21.00 | 32.05 | 28.34 | 29.91 |
|  | VIT_213s0175g00120 | 22.13 | 18.15 | 19.39 | 13.65 | 13.72 | 16.74 | 15.13 | 17.07 | 25.59 | 18.88 | 24.71 | 28.00 | 27.49 | 27.68 |
|  | VIT_218s0001g10450 | 23.71 | 45.43 | 35.65 | 44.19 | 59.47 | 33.77 | 49.29 | 45.27 | 28.04 | 32.39 | 28.85 | 26.52 | 31.63 | 32.81 |
|  | VIT_218s0001g14890 | 0.06 | 0.06 | 0.00 | 0.03 | 0.03 | 0.00 | 0.08 | 0.03 | 0.09 | 0.09 | 0.00 | 0.05 | 0.08 | 0.03 |
|  | VIT_218s0072g00470 | 17.76 | 28.74 | 28.00 | 16.80 | 22.61 | 13.69 | 15.67 | 13.33 | 15.51 | 12.68 | 14.34 | 18.33 | 20.04 | 15.82 |
|  | VIT_218s0076g00330 | 19.19 | 25.11 | 28.60 | 52.78 | 51.22 | 61.26 | 62.84 | 63.63 | 74.04 | 79.40 | 75.63 | 86.03 | 89.99 | 81.26 |
|  | VIT_219s0015g01020 | 3.43 | 2.06 | 17.75 | 0.63 | 0.42 | 0.78 | 0.42 | 0.86 | 0.89 | 0.85 | 0.95 | 0.71 | 1.12 | 1.02 |
| ETR | VIT_204s0043g00690 | 5.68 | 2.43 | 10.22 | 0.16 | 0.00 | 0.15 | 0.00 | 0.16 | 0.16 | 0.31 | 0.31 | 0.15 | 0.16 | 0.47 |
|  | VIT_219s0014g04420 | 1.28 | 0.28 | 3.08 | 0.00 | 0.00 | 0.00 | 0.00 | 0.00 | 0.00 | 0.00 | 0.00 | 0.00 | 0.00 | 0.00 |
|  | VIT_205s0049g00090 | 11.80 | 49.66 | 44.29 | 8.38 | 6.56 | 5.03 | 4.93 | 5.69 | 5.78 | 6.65 | 5.63 | 10.52 | 9.34 | 7.97 |
|  | VIT_206s0004g05240 | 0.49 | 0.89 | 2.22 | 0.34 | 0.76 | 0.29 | 0.44 | 0.67 | 0.82 | 1.40 | 1.28 | 1.28 | 1.19 | 1.18 |
|  | VIT_207s0005g00850 | 23.23 | 29.68 | 32.84 | 17.13 | 18.06 | 11.37 | 16.02 | 18.02 | 16.36 | 16.92 | 16.35 | 18.66 | 18.31 | 15.37 |
|  | VIT_214s0081g00630 | 13.74 | 13.66 | 16.69 | 13.93 | 12.78 | 14.11 | 13.51 | 14.51 | 17.76 | 19.59 | 18.10 | 21.67 | 20.83 | 22.24 |
|  | VIT_219s0093g00580 | 11.31 | 10.61 | 10.80 | 12.08 | 11.07 | 12.42 | 11.84 | 12.30 | 15.07 | 15.62 | 17.05 | 20.14 | 19.27 | 20.90 |
| CTR1 | VIT_200s0684g00010 | 7.30 | 5.52 | 8.81 | 3.22 | 3.54 | 2.44 | 2.54 | 4.33 | 5.73 | 2.62 | 6.16 | 4.12 | 5.49 | 4.89 |
|  | VIT_200s1002g00020 | 1.55 | 0.00 | 1.19 | 0.08 | 0.32 | 0.00 | 0.33 | 0.54 | 0.00 | 0.73 | 0.00 | 1.33 | 2.00 | 1.76 |
|  | VIT_200s2483g00010 | 0.00 | 0.51 | 0.36 | 0.00 | 0.00 | 1.07 | 0.00 | 0.00 | 0.11 | 0.11 | 1.67 | 0.22 | 0.00 | 0.00 |
|  | VIT_201s0011g01490 | 112.20 | 83.30 | 101.87 | 90.29 | 88.08 | 87.74 | 88.82 | 100.69 | 101.43 | 107.59 | 121.19 | 135.71 | 147.73 | 154.48 |
|  | VIT_202s0025g04110 | 0.26 | 0.41 | 0.81 | 0.12 | 0.25 | 0.31 | 0.47 | 1.92 | 0.70 | 0.60 | 2.20 | 0.69 | 1.08 | 1.65 |
|  | VIT_204s0008g01710 | 0.05 | 0.00 | 0.00 | 0.00 | 0.00 | 0.00 | 0.00 | 0.00 | 0.00 | 0.00 | 0.00 | 0.00 | 0.05 | 0.00 |
|  | VIT_204s0008g05600 | 13.58 | 10.41 | 8.76 | 14.31 | 9.22 | 15.23 | 10.83 | 12.63 | 25.47 | 21.15 | 22.14 | 35.76 | 38.21 | 38.99 |
|  | VIT_205s0077g00920 | 9.77 | 10.14 | 9.98 | 12.22 | 10.30 | 18.50 | 18.64 | 17.03 | 14.45 | 16.42 | 15.76 | 12.09 | 11.69 | 12.44 |
|  | VIT_205s0094g01080 | 27.18 | 31.95 | 29.60 | 3.72 | 5.73 | 3.68 | 4.68 | 4.60 | 4.85 | 2.94 | 2.84 | 2.27 | 1.87 | 2.20 |
|  | VIT_208s0007g03910 | 18.34 | 24.56 | 28.13 | 14.17 | 16.89 | 13.20 | 14.87 | 16.32 | 15.04 | 16.30 | 16.33 | 17.44 | 16.40 | 18.68 |
|  | VIT_208s0058g01180 | 13.58 | 9.45 | 8.01 | 8.47 | 7.22 | 10.04 | 8.50 | 8.04 | 10.72 | 10.22 | 11.13 | 13.39 | 13.43 | 13.00 |
|  | VIT_209s0002g07110 | 20.34 | 20.54 | 24.00 | 73.04 | 52.33 | 51.00 | 44.57 | 57.62 | 81.11 | 66.97 | 71.68 | 152.42 | 158.18 | 153.31 |
|  | VIT_210s0003g01350 | 1.24 | 0.47 | 0.32 | 0.17 | 0.46 | 0.15 | 0.15 | 0.63 | 1.44 | 0.31 | 0.63 | 1.09 | 0.79 | 0.16 |
|  | VIT_210s0003g01355 | 0.78 | 0.61 | 0.53 | 0.70 | 0.47 | 0.77 | 0.74 | 0.85 | 1.05 | 1.19 | 1.26 | 1.19 | 1.37 | 1.29 |
|  | VIT_210s0003g02060 | 29.40 | 47.38 | 47.30 | 25.42 | 42.77 | 15.98 | 21.19 | 20.63 | 8.96 | 10.11 | 11.18 | 7.44 | 7.79 | 7.60 |
|  | VIT_211s0016g04880 | 0.53 | 0.16 | 0.22 | 0.10 | 0.12 | 0.00 | 0.21 | 0.02 | 0.08 | 0.19 | 0.14 | 0.06 | 0.02 | 0.00 |
|  | VIT_212s0028g02130 | 13.73 | 14.14 | 14.21 | 11.66 | 9.94 | 12.13 | 13.27 | 13.06 | 18.89 | 18.28 | 16.88 | 16.37 | 18.53 | 16.02 |
|  | VIT_213s0074g00430 | 13.32 | 9.93 | 12.43 | 11.12 | 9.90 | 9.88 | 9.29 | 10.47 | 11.53 | 10.04 | 12.59 | 12.26 | 14.22 | 14.90 |
|  | VIT_214s0006g02090 | 0.00 | 0.00 | 0.00 | 0.40 | 0.00 | 0.20 | 0.37 | 0.39 | 0.78 | 0.40 | 0.20 | 0.00 | 0.31 | 0.00 |
|  | VIT_214s0066g00370 | 55.38 | 63.12 | 76.86 | 28.87 | 40.08 | 20.91 | 25.46 | 22.45 | 17.12 | 15.75 | 18.93 | 11.70 | 11.74 | 12.55 |
|  | VIT_214s0066g01400 | 14.11 | 10.19 | 10.89 | 7.65 | 8.00 | 5.26 | 5.18 | 7.58 | 4.85 | 5.36 | 6.12 | 4.61 | 4.59 | 4.24 |
|  | VIT_215s0046g02840 | 1.49 | 1.41 | 2.11 | 0.97 | 1.58 | 1.27 | 1.80 | 2.17 | 2.85 | 2.62 | 2.11 | 2.60 | 3.76 | 2.32 |
|  | VIT_215s0046g02850 | 0.34 | 0.69 | 0.72 | 0.34 | 0.37 | 0.37 | 0.44 | 0.87 | 1.01 | 0.53 | 0.57 | 1.06 | 1.17 | 0.82 |
|  | VIT_217s0000g08140 | 27.91 | 39.92 | 32.33 | 85.51 | 69.84 | 105.70 | 99.51 | 95.50 | 95.54 | 102.85 | 91.53 | 102.57 | 109.68 | 105.99 |
|  | VIT_217s0000g09290 | 0.06 | 0.47 | 0.23 | 0.35 | 0.17 | 0.34 | 0.87 | 0.52 | 0.53 | 0.47 | 0.83 | 2.23 | 2.35 | 1.57 |
|  | VIT_218s0001g00720 | 6.86 | 5.33 | 8.36 | 0.20 | 0.63 | 0.37 | 0.21 | 0.30 | 0.43 | 0.40 | 0.58 | 0.16 | 0.20 | 0.10 |
|  | VIT_218s0001g07700 | 6.05 | 3.77 | 22.87 | 2.64 | 3.02 | 1.76 | 3.16 | 15.42 | 3.50 | 4.33 | 12.62 | 2.30 | 5.01 | 5.23 |
| SIMKK | VIT_209s0018g01820 | 22.01 | 44.56 | 50.41 | 20.90 | 23.10 | 29.84 | 31.11 | 35.78 | 51.02 | 56.14 | 56.27 | 61.79 | 65.54 | 57.75 |
|  | VIT_217s0000g01970 | 36.33 | 126.59 | 113.96 | 14.97 | 18.37 | 11.30 | 13.04 | 21.24 | 22.84 | 19.73 | 25.22 | 8.91 | 11.41 | 10.29 |
|  | VIT_217s0000g06490 | 2.38 | 6.44 | 5.52 | 2.12 | 2.04 | 3.89 | 3.39 | 3.90 | 4.66 | 4.64 | 6.72 | 6.62 | 8.36 | 4.27 |
| MPK6 | VIT_205s0094g00900 | 44.18 | 53.52 | 43.88 | 55.21 | 56.68 | 56.21 | 57.19 | 53.55 | 71.09 | 66.69 | 63.60 | 77.92 | 79.47 | 76.07 |
|  | VIT_208s0040g01730 | 35.40 | 32.06 | 37.96 | 40.44 | 40.50 | 44.49 | 50.41 | 59.12 | 51.12 | 55.79 | 59.35 | 59.24 | 62.93 | 63.00 |
|  | VIT_214s0036g00600 | 0.00 | 0.00 | 0.00 | 0.00 | 0.00 | 0.00 | 0.00 | 0.00 | 0.10 | 0.00 | 0.00 | 0.00 | 0.00 | 0.13 |
| EIN2 | VIT_200s0357g00120 | 0.00 | 0.13 | 0.08 | 0.00 | 0.08 | 0.00 | 0.00 | 0.42 | 0.04 | 0.00 | 0.46 | 0.00 | 0.00 | 0.00 |
|  | VIT_206s0004g01610 | 6.43 | 9.45 | 11.19 | 3.12 | 3.56 | 3.27 | 3.66 | 5.29 | 5.52 | 5.18 | 6.16 | 7.99 | 6.93 | 7.09 |
|  | VIT_206s0009g01380 | 21.33 | 22.24 | 28.04 | 7.48 | 8.29 | 5.13 | 7.27 | 9.10 | 9.86 | 9.60 | 9.65 | 6.85 | 6.18 | 5.70 |
|  | VIT_213s0047g00250 | 273.56 | 356.26 | 417.65 | 198.13 | 239.37 | 187.26 | 218.37 | 271.70 | 194.38 | 206.87 | 210.23 | 203.27 | 211.40 | 201.89 |
| EIN3 | VIT_204s0008g05470 | 23.43 | 31.36 | 34.26 | 11.16 | 10.07 | 18.82 | 17.34 | 23.25 | 33.50 | 32.29 | 33.57 | 44.27 | 41.74 | 41.52 |
|  | VIT_207s0031g00340 | 5.00 | 6.60 | 3.96 | 3.19 | 2.11 | 3.11 | 3.34 | 3.62 | 6.39 | 4.03 | 5.79 | 3.58 | 3.41 | 3.73 |
|  | VIT_208s0007g03580 | 2.73 | 3.56 | 3.60 | 1.68 | 2.33 | 2.68 | 2.04 | 2.35 | 2.74 | 2.95 | 2.59 | 2.82 | 3.12 | 2.30 |
|  | VIT_209s0002g08270 | 21.60 | 24.02 | 26.37 | 26.09 | 22.29 | 22.63 | 19.95 | 27.19 | 38.27 | 35.28 | 39.41 | 39.89 | 48.11 | 49.50 |
|  | VIT_211s0016g05410 | 36.25 | 89.68 | 90.87 | 31.64 | 30.26 | 54.95 | 51.60 | 59.59 | 53.64 | 59.20 | 53.39 | 45.97 | 46.56 | 50.64 |
|  | VIT_211s0118g00480 | 17.97 | 15.71 | 18.62 | 15.37 | 15.18 | 11.63 | 10.90 | 17.82 | 17.47 | 17.82 | 19.87 | 22.65 | 27.10 | 23.11 |
|  | VIT_219s0015g01910 | 2.61 | 1.08 | 1.68 | 1.67 | 1.15 | 1.75 | 1.56 | 2.07 | 2.12 | 1.74 | 2.26 | 3.54 | 3.05 | 2.61 |
|  | VIT_219s0090g00450 | 5.73 | 10.30 | 8.94 | 6.18 | 5.04 | 7.12 | 6.98 | 6.77 | 11.74 | 10.33 | 9.87 | 16.55 | 16.40 | 15.61 |
| EBF1/2 | VIT_205s0049g00490 | 0.00 | 0.56 | 0.14 | 0.00 | 0.41 | 0.00 | 0.00 | 0.28 | 0.14 | 0.00 | 0.15 | 0.00 | 0.00 | 0.00 |
|  | VIT_205s0049g00510 | 4.43 | 160.29 | 13.78 | 2906.29 | 1957.95 | 3077.60 | 2688.82 | 2008.61 | 2370.57 | 2378.42 | 2106.05 | 1888.57 | 1981.43 | 1860.83 |
|  | VIT_207s0005g03200 | 0.14 | 0.42 | 0.56 | 0.00 | 0.00 | 0.00 | 0.00 | 0.27 | 0.00 | 0.14 | 0.00 | 0.00 | 0.00 | 0.00 |
|  | VIT_207s0005g03210 | 0.27 | 0.98 | 0.99 | 0.00 | 0.09 | 0.00 | 0.00 | 0.00 | 0.00 | 0.00 | 0.27 | 0.00 | 0.00 | 0.00 |
|  | VIT_207s0005g03230 | 2.80 | 23.05 | 23.31 | 0.63 | 0.76 | 0.23 | 0.49 | 1.60 | 0.28 | 0.44 | 1.26 | 0.00 | 0.06 | 0.00 |
|  | VIT_207s0005g03260 | 1.20 | 18.11 | 19.40 | 0.37 | 1.96 | 0.18 | 0.09 | 1.56 | 0.45 | 0.21 | 1.65 | 0.06 | 0.00 | 0.06 |
|  | VIT_214s0081g00730 | 0.25 | 1.84 | 2.02 | 0.09 | 0.08 | 0.00 | 0.25 | 0.34 | 0.08 | 0.00 | 1.11 | 0.52 | 0.34 | 0.00 |
|  | VIT_200s0211g00030 | 1.84 | 3.81 | 3.25 | 3.53 | 3.16 | 3.70 | 4.31 | 3.17 | 3.84 | 3.97 | 3.54 | 1.92 | 2.14 | 2.52 |
|  | VIT_200s0258g00040 | 1.22 | 1.13 | 2.30 | 1.04 | 1.33 | 1.44 | 1.73 | 1.73 | 1.35 | 0.79 | 1.02 | 0.87 | 0.92 | 0.89 |
|  | VIT_200s0258g00060 | 0.45 | 0.12 | 0.86 | 0.00 | 0.28 | 0.00 | 0.18 | 0.08 | 0.00 | 0.00 | 0.00 | 0.00 | 0.00 | 0.00 |
|  | VIT_200s0258g00070 | 0.00 | 0.00 | 0.00 | 0.19 | 0.00 | 0.00 | 0.00 | 0.00 | 0.00 | 0.00 | 0.00 | 0.00 | 0.00 | 0.00 |
|  | VIT_200s0265g00100 | 0.36 | 0.65 | 0.12 | 0.31 | 0.12 | 0.50 | 0.16 | 0.21 | 0.28 | 0.15 | 0.30 | 0.09 | 0.09 | 0.13 |
|  | VIT_200s0294g00040 | 0.24 | 1.69 | 0.65 | 1.37 | 0.39 | 0.61 | 0.40 | 0.45 | 2.21 | 1.09 | 1.28 | 0.47 | 0.14 | 0.40 |
|  | VIT_200s0425g00020 | 0.00 | 0.00 | 0.00 | 0.00 | 0.28 | 0.00 | 0.00 | 0.00 | 0.00 | 0.00 | 0.00 | 0.00 | 0.00 | 0.00 |
|  | VIT_200s0425g00030 | 0.31 | 0.16 | 0.26 | 0.88 | 0.00 | 0.12 | 0.23 | 0.17 | 0.00 | 0.18 | 0.00 | 0.22 | 0.11 | 0.18 |
|  | VIT_200s0425g00040 | 0.00 | 1.59 | 0.00 | 0.61 | 0.65 | 0.69 | 0.28 | 0.32 | 0.35 | 0.33 | 0.10 | 0.00 | 0.10 | 0.01 |
|  | VIT_200s0897g00010 | 56.82 | 48.26 | 58.87 | 36.48 | 37.66 | 24.07 | 32.96 | 41.48 | 20.51 | 23.50 | 32.41 | 21.70 | 21.72 | 25.51 |
|  | VIT_201s0011g04060 | 9.46 | 3.39 | 5.43 | 1.27 | 1.14 | 1.13 | 1.87 | 1.65 | 1.38 | 1.36 | 0.82 | 0.77 | 0.54 | 0.67 |
|  | VIT_202s0241g00120 | 4.70 | 2.08 | 2.72 | 3.36 | 2.41 | 3.61 | 5.38 | 4.07 | 3.68 | 3.75 | 2.84 | 0.48 | 0.93 | 0.70 |
|  | VIT_203s0091g00560 | 57.23 | 70.32 | 66.82 | 36.76 | 40.35 | 30.61 | 30.37 | 35.35 | 40.76 | 31.96 | 35.88 | 37.84 | 36.32 | 31.78 |
|  | VIT_203s0097g00070 | 1.45 | 0.46 | 0.87 | 0.52 | 0.71 | 0.15 | 0.61 | 0.24 | 0.38 | 0.15 | 0.15 | 0.69 | 0.28 | 0.21 |
|  | VIT_203s0097g00120 | 0.00 | 0.00 | 0.03 | 0.00 | 0.00 | 0.00 | 0.00 | 0.03 | 0.00 | 0.00 | 0.00 | 0.00 | 0.00 | 0.00 |
|  | VIT_204s0044g00720 | 3.54 | 1.85 | 2.43 | 0.34 | 0.74 | 0.10 | 0.07 | 0.72 | 0.08 | 0.02 | 0.08 | 0.03 | 0.00 | 0.08 |
|  | VIT_205s0020g03220 | 0.00 | 0.00 | 0.00 | 0.00 | 0.03 | 0.00 | 0.09 | 0.00 | 0.00 | 0.00 | 0.06 | 0.00 | 0.16 | 0.00 |
|  | VIT_205s0020g03240 | 23.13 | 10.67 | 8.46 | 3.14 | 1.46 | 1.51 | 0.80 | 2.77 | 0.72 | 0.32 | 1.40 | 0.24 | 0.08 | 0.97 |
|  | VIT_205s0020g03290 | 0.46 | 0.17 | 0.55 | 0.17 | 0.04 | 0.21 | 0.00 | 0.13 | 0.42 | 0.25 | 0.26 | 0.52 | 0.34 | 0.44 |
|  | VIT_205s0020g03300 | 0.21 | 0.13 | 0.34 | 0.00 | 0.08 | 0.04 | 0.04 | 0.25 | 0.12 | 0.25 | 1.11 | 0.46 | 0.04 | 0.39 |
|  | VIT_205s0029g00080 | 21.82 | 4.17 | 3.81 | 0.79 | 1.65 | 0.74 | 1.04 | 1.47 | 0.70 | 0.55 | 0.83 | 0.76 | 0.60 | 0.76 |
|  | VIT_206s0004g08210 | 13.53 | 5.91 | 5.01 | 5.79 | 7.72 | 5.90 | 7.89 | 6.53 | 3.08 | 3.09 | 2.93 | 1.09 | 0.75 | 0.98 |
|  | VIT_207s0129g01070 | 4.80 | 0.81 | 0.53 | 0.48 | 0.14 | 0.14 | 0.10 | 0.44 | 0.20 | 0.04 | 0.12 | 0.00 | 0.00 | 0.08 |
|  | VIT_207s0151g00470 | 7.72 | 5.79 | 6.16 | 2.47 | 2.17 | 1.76 | 1.67 | 3.27 | 2.58 | 2.21 | 2.25 | 1.71 | 1.33 | 2.14 |
|  | VIT_208s0007g06560 | 0.46 | 0.26 | 0.55 | 0.09 | 0.05 | 0.17 | 0.14 | 0.38 | 0.65 | 0.52 | 0.61 | 0.09 | 0.15 | 0.18 |
|  | VIT_208s0007g08230 | 8.12 | 8.37 | 8.16 | 4.66 | 4.20 | 5.56 | 5.18 | 5.12 | 4.48 | 4.83 | 5.72 | 2.16 | 1.82 | 2.69 |
|  | VIT_208s0040g02210 | 10.99 | 4.37 | 4.81 | 2.70 | 4.40 | 1.11 | 2.01 | 4.11 | 2.35 | 1.92 | 2.91 | 1.61 | 2.12 | 1.55 |
|  | VIT_209s0002g02720 | 0.00 | 0.00 | 0.00 | 0.00 | 0.00 | 0.00 | 0.03 | 0.00 | 0.03 | 0.00 | 0.03 | 0.00 | 0.00 | 0.00 |
|  | VIT_209s0002g02800 | 0.00 | 0.04 | 0.00 | 0.04 | 0.00 | 0.15 | 0.07 | 0.04 | 0.00 | 0.00 | 0.00 | 0.00 | 0.00 | 0.00 |
|  | VIT_209s0002g03130 | 0.93 | 0.00 | 0.00 | 0.10 | 0.05 | 0.19 | 0.22 | 0.13 | 0.13 | 0.05 | 0.00 | 0.00 | 0.00 | 0.00 |
|  | VIT_210s0003g01860 | 3.61 | 5.10 | 4.41 | 4.02 | 3.98 | 3.31 | 3.53 | 3.12 | 5.94 | 5.72 | 5.57 | 2.49 | 2.40 | 2.72 |
|  | VIT_210s0003g01990 | 6.49 | 19.36 | 19.48 | 7.80 | 14.69 | 3.95 | 9.72 | 4.56 | 0.68 | 1.16 | 2.18 | 0.07 | 0.17 | 0.08 |
|  | VIT_211s0016g04840 | 3.24 | 1.43 | 2.09 | 0.75 | 0.60 | 0.55 | 0.39 | 0.35 | 0.00 | 0.04 | 0.18 | 0.00 | 0.04 | 0.00 |
|  | VIT_212s0055g01150 | 6.46 | 6.83 | 8.47 | 2.87 | 2.65 | 1.16 | 1.07 | 1.39 | 0.46 | 0.59 | 1.86 | 0.17 | 0.29 | 0.54 |
|  | VIT_212s0055g01160 | 19.63 | 33.12 | 37.27 | 18.83 | 19.26 | 17.71 | 17.33 | 19.73 | 15.30 | 15.01 | 17.14 | 12.50 | 12.58 | 12.79 |
|  | VIT_212s0055g01280 | 0.37 | 1.26 | 0.69 | 0.29 | 0.19 | 0.40 | 0.18 | 0.37 | 0.15 | 0.42 | 0.41 | 0.11 | 0.11 | 0.32 |
|  | VIT_212s0055g01285 | 0.80 | 1.58 | 1.14 | 0.55 | 0.00 | 0.36 | 0.36 | 0.44 | 0.70 | 0.00 | 0.27 | 0.00 | 0.00 | 0.00 |
|  | VIT_212s0121g00300 | 1.58 | 3.57 | 2.72 | 1.73 | 1.28 | 0.92 | 1.10 | 1.49 | 0.94 | 1.24 | 1.13 | 0.37 | 0.45 | 0.51 |
|  | VIT_212s0121g00310 | 0.33 | 1.04 | 0.34 | 0.26 | 0.10 | 0.29 | 0.17 | 0.17 | 0.48 | 0.22 | 0.47 | 0.24 | 0.19 | 0.37 |
|  | VIT_212s0121g00430 | 1.00 | 2.07 | 1.41 | 0.68 | 0.00 | 0.41 | 0.82 | 0.49 | 0.00 | 0.28 | 0.50 | 0.14 | 0.28 | 0.00 |
|  | VIT_212s0134g00243 | 2.37 | 1.98 | 1.88 | 0.52 | 0.54 | 0.25 | 0.10 | 0.66 | 0.43 | 0.16 | 0.21 | 0.00 | 0.00 | 0.25 |
|  | VIT_212s0134g00320 | 0.00 | 0.27 | 0.00 | 0.78 | 0.00 | 0.18 | 0.00 | 0.25 | 0.00 | 0.32 | 0.00 | 0.21 | 0.09 | 0.00 |
|  | VIT_213s0106g00210 | 0.31 | 0.00 | 0.30 | 0.00 | 0.00 | 0.00 | 0.00 | 0.00 | 0.00 | 0.00 | 0.00 | 0.00 | 0.29 | 0.00 |
|  | VIT_213s0156g00620 | 0.16 | 0.95 | 0.76 | 0.41 | 0.16 | 0.16 | 0.40 | 0.78 | 0.50 | 0.44 | 0.65 | 0.20 | 0.59 | 0.68 |
|  | VIT_214s0030g00390 | 0.07 | 0.00 | 0.03 | 0.00 | 0.00 | 0.03 | 0.03 | 0.00 | 0.00 | 0.05 | 0.00 | 0.00 | 0.04 | 0.07 |
|  | VIT_215s0046g01810 | 2.88 | 1.09 | 1.79 | 2.53 | 1.89 | 3.34 | 3.44 | 2.82 | 3.60 | 4.03 | 3.43 | 1.57 | 1.29 | 1.39 |
|  | VIT_216s0013g01170 | 0.00 | 0.10 | 0.00 | 0.00 | 0.05 | 0.04 | 0.05 | 0.00 | 0.00 | 0.00 | 0.00 | 0.00 | 0.00 | 0.00 |
|  | VIT_216s0013g01190 | 0.00 | 0.04 | 0.04 | 0.00 | 0.00 | 0.02 | 0.00 | 0.00 | 0.00 | 0.03 | 0.00 | 0.00 | 0.00 | 0.00 |
|  | VIT_216s0013g01230 | 0.00 | 0.00 | 0.02 | 0.00 | 0.00 | 0.04 | 0.02 | 0.00 | 0.00 | 0.04 | 0.00 | 0.00 | 0.02 | 0.00 |
|  | VIT_216s0013g01365 | 0.00 | 0.00 | 0.00 | 0.02 | 0.00 | 0.00 | 0.02 | 0.00 | 0.00 | 0.00 | 0.00 | 0.02 | 0.00 | 0.00 |
|  | VIT_216s0013g01370 | 0.00 | 0.00 | 0.00 | 0.00 | 0.00 | 0.00 | 0.00 | 0.00 | 0.00 | 0.00 | 0.00 | 0.00 | 0.06 | 0.00 |
|  | VIT_216s0013g01386 | 0.00 | 0.00 | 0.00 | 0.00 | 0.00 | 0.00 | 0.00 | 0.00 | 0.00 | 0.00 | 0.03 | 0.00 | 0.00 | 0.00 |
|  | VIT_216s0022g01640 | 4.41 | 3.26 | 2.49 | 0.91 | 0.36 | 0.72 | 0.59 | 0.31 | 0.41 | 0.05 | 0.37 | 0.00 | 0.05 | 0.00 |
|  | VIT_216s0039g01150 | 0.94 | 0.87 | 0.12 | 0.23 | 0.63 | 0.36 | 0.34 | 0.47 | 0.00 | 0.14 | 0.00 | 0.00 | 0.00 | 0.00 |
|  | VIT_216s0039g01180 | 1.84 | 1.16 | 1.40 | 1.67 | 1.07 | 1.02 | 1.69 | 1.94 | 2.16 | 1.63 | 1.55 | 3.03 | 3.20 | 3.35 |
|  | VIT_216s0050g01720 | 1.18 | 9.23 | 3.57 | 1.96 | 0.76 | 1.02 | 1.61 | 1.25 | 0.97 | 0.73 | 2.25 | 0.23 | 0.00 | 0.23 |
|  | VIT_216s0050g02700 | 1.97 | 1.23 | 0.37 | 2.25 | 1.87 | 0.89 | 1.41 | 4.44 | 1.68 | 0.90 | 0.95 | 0.65 | 0.20 | 0.86 |
|  | VIT_216s0050g02770 | 0.00 | 0.00 | 0.00 | 0.00 | 0.00 | 0.00 | 0.00 | 0.00 | 0.06 | 0.00 | 0.00 | 0.00 | 0.00 | 0.00 |
|  | VIT_216s0098g00240 | 1.16 | 1.33 | 1.63 | 1.60 | 1.03 | 3.06 | 2.27 | 2.72 | 3.68 | 4.35 | 4.20 | 4.97 | 4.96 | 5.43 |
|  | VIT_216s0098g00400 | 0.64 | 1.39 | 0.56 | 0.25 | 0.04 | 0.00 | 0.00 | 0.04 | 0.00 | 0.00 | 0.00 | 0.00 | 0.00 | 0.08 |
|  | VIT_216s0148g00070 | 0.94 | 1.11 | 1.50 | 0.95 | 0.23 | 0.45 | 1.15 | 1.02 | 0.36 | 0.52 | 0.84 | 0.17 | 0.38 | 0.05 |
|  | VIT_216s0148g00100 | 1.01 | 2.06 | 1.63 | 1.71 | 0.81 | 1.16 | 1.29 | 2.04 | 1.56 | 1.17 | 1.10 | 1.64 | 1.47 | 1.34 |
|  | VIT_216s0148g00140 | 0.13 | 2.37 | 3.49 | 0.58 | 0.04 | 0.45 | 0.11 | 0.38 | 0.12 | 0.15 | 0.23 | 0.05 | 0.12 | 0.05 |
|  | VIT_216s0148g00220 | 1.76 | 0.67 | 0.83 | 0.18 | 0.18 | 0.00 | 0.00 | 0.91 | 0.00 | 0.00 | 0.00 | 0.06 | 0.00 | 0.17 |
|  | VIT_216s0148g00280 | 1.42 | 2.43 | 3.28 | 2.30 | 0.64 | 1.71 | 2.08 | 2.33 | 2.91 | 3.04 | 1.37 | 0.87 | 1.23 | 2.64 |
|  | VIT_217s0000g03330 | 0.80 | 1.01 | 1.19 | 0.11 | 0.30 | 0.06 | 0.14 | 0.37 | 0.22 | 0.22 | 0.46 | 0.00 | 0.14 | 0.06 |
|  | VIT_218s0001g01960 | 3.41 | 11.35 | 12.78 | 1.64 | 1.36 | 1.15 | 0.58 | 1.58 | 0.80 | 0.74 | 2.60 | 0.81 | 0.25 | 0.54 |
|  | VIT_218s0075g00520 | 3.47 | 6.33 | 5.26 | 8.01 | 5.38 | 6.12 | 4.92 | 5.85 | 8.48 | 6.77 | 6.97 | 10.47 | 9.30 | 9.96 |
|  | VIT_218s0082g00009 | 0.38 | 0.27 | 0.27 | 0.00 | 0.00 | 0.05 | 0.00 | 0.11 | 0.00 | 0.00 | 0.05 | 0.05 | 0.00 | 0.00 |
|  | VIT_218s0166g00120 | 0.12 | 0.17 | 0.06 | 0.12 | 0.11 | 0.06 | 0.06 | 0.23 | 0.41 | 0.23 | 0.12 | 0.51 | 0.17 | 0.30 |
|  | VIT_219s0014g00480 | 0.89 | 0.78 | 1.51 | 0.30 | 0.16 | 0.47 | 0.24 | 0.73 | 0.40 | 0.29 | 0.35 | 0.35 | 0.55 | 0.47 |
|  | VIT_219s0014g00680 | 2.61 | 0.93 | 1.08 | 0.13 | 0.18 | 0.12 | 0.15 | 0.09 | 0.05 | 0.05 | 0.10 | 0.02 | 0.02 | 0.04 |
| BAK1 | VIT_216s0039g01990 | 0.07 | 0.19 | 0.10 | 0.00 | 0.00 | 0.31 | 0.18 | 0.26 | 0.08 | 0.06 | 0.06 | 0.13 | 0.00 | 0.13 |
|  | VIT_200s0144g00110 | 2.29 | 2.07 | 1.68 | 2.46 | 1.95 | 2.09 | 1.65 | 2.65 | 2.30 | 2.32 | 1.50 | 0.00 | 0.00 | 0.39 |
|  | VIT_200s0220g00120 | 48.94 | 31.27 | 34.20 | 36.68 | 33.91 | 29.05 | 27.37 | 30.18 | 26.87 | 28.72 | 29.18 | 21.95 | 23.99 | 24.88 |
|  | VIT_200s0397g00050 | 22.51 | 39.75 | 34.44 | 21.77 | 16.76 | 11.66 | 14.16 | 13.91 | 9.14 | 13.20 | 9.90 | 2.89 | 2.67 | 3.76 |
|  | VIT_200s0400g00030 | 0.34 | 2.16 | 2.06 | 0.92 | 0.76 | 0.52 | 0.16 | 0.17 | 0.00 | 0.00 | 0.00 | 0.00 | 0.00 | 0.12 |
|  | VIT_200s0421g00020 | 0.00 | 0.00 | 0.00 | 0.00 | 0.00 | 0.00 | 0.05 | 0.05 | 0.00 | 0.00 | 0.05 | 0.00 | 0.00 | 0.00 |
|  | VIT_200s0421g00040 | 4.28 | 11.84 | 9.14 | 5.20 | 2.38 | 1.31 | 2.18 | 2.42 | 1.80 | 1.67 | 2.86 | 0.17 | 0.11 | 0.29 |
|  | VIT_200s1526g00005 | 0.04 | 0.00 | 0.00 | 0.00 | 0.00 | 0.00 | 0.00 | 0.02 | 0.03 | 0.00 | 0.00 | 0.06 | 0.00 | 0.05 |
|  | VIT_200s1764g00020 | 8.75 | 2.98 | 2.73 | 8.86 | 14.43 | 8.54 | 9.57 | 9.13 | 1.51 | 2.40 | 1.63 | 0.64 | 0.28 | 0.53 |
|  | VIT_200s2085g00010 | 20.90 | 30.41 | 28.37 | 22.24 | 22.68 | 17.43 | 20.45 | 20.44 | 17.11 | 15.65 | 15.50 | 9.35 | 9.06 | 9.24 |
|  | VIT_201s0010g01710 | 4.32 | 8.65 | 10.80 | 9.21 | 6.29 | 9.07 | 8.78 | 10.60 | 18.80 | 18.55 | 18.11 | 16.42 | 17.80 | 15.70 |
|  | VIT_201s0010g03124 | 0.14 | 0.00 | 0.00 | 0.00 | 0.14 | 0.00 | 0.00 | 0.10 | 0.00 | 0.00 | 0.00 | 0.00 | 0.00 | 0.00 |
|  | VIT_201s0010g03210 | 2.32 | 0.99 | 0.90 | 1.12 | 2.47 | 0.53 | 1.57 | 1.27 | 0.79 | 1.33 | 0.55 | 0.25 | 0.12 | 0.29 |
|  | VIT_201s0010g03255 | 1.35 | 1.75 | 1.93 | 1.78 | 3.07 | 1.74 | 2.13 | 1.62 | 1.85 | 0.65 | 0.65 | 0.48 | 0.50 | 0.41 |
|  | VIT_201s0011g00420 | 18.45 | 7.67 | 11.01 | 9.33 | 10.03 | 8.02 | 8.46 | 10.81 | 6.60 | 7.67 | 9.85 | 6.16 | 5.31 | 6.77 |
|  | VIT_201s0011g06410 | 3.82 | 0.50 | 0.64 | 0.43 | 0.54 | 0.92 | 0.74 | 1.17 | 0.95 | 0.81 | 0.84 | 0.54 | 0.40 | 0.59 |
|  | VIT_201s0026g01060 | 18.24 | 11.15 | 11.31 | 17.83 | 15.91 | 17.44 | 19.11 | 16.74 | 23.19 | 22.50 | 20.62 | 14.78 | 13.65 | 15.74 |
|  | VIT_201s0026g01780 | 30.36 | 17.35 | 19.55 | 8.69 | 8.05 | 2.23 | 3.39 | 4.26 | 1.17 | 1.55 | 2.07 | 0.50 | 0.71 | 0.74 |
|  | VIT_201s0150g00020 | 2.51 | 0.99 | 1.74 | 2.11 | 2.49 | 2.21 | 2.58 | 2.48 | 1.81 | 2.67 | 1.45 | 0.65 | 0.58 | 1.04 |
|  | VIT_203s0180g00150 | 5.14 | 2.52 | 2.36 | 0.09 | 0.38 | 0.02 | 0.04 | 0.16 | 0.00 | 0.00 | 0.00 | 0.00 | 0.00 | 0.00 |
|  | VIT_204s0008g06290 | 2.46 | 2.44 | 1.64 | 0.99 | 0.97 | 0.66 | 1.09 | 0.98 | 0.37 | 0.56 | 0.29 | 0.34 | 0.43 | 0.34 |
|  | VIT_204s0008g06300 | 6.23 | 5.01 | 3.47 | 2.44 | 1.72 | 2.38 | 3.06 | 2.61 | 1.94 | 2.83 | 1.59 | 1.28 | 1.20 | 1.05 |
|  | VIT_205s0049g01210 | 0.13 | 0.25 | 0.12 | 0.00 | 0.00 | 0.49 | 0.51 | 0.38 | 0.00 | 0.00 | 0.13 | 0.00 | 0.00 | 0.00 |
|  | VIT_205s0094g01270 | 0.42 | 0.25 | 0.34 | 0.62 | 0.42 | 0.42 | 0.43 | 0.30 | 0.41 | 0.79 | 0.74 | 0.06 | 0.12 | 0.51 |
|  | VIT_205s0094g01290 | 0.35 | 0.00 | 0.68 | 0.33 | 0.16 | 0.00 | 0.17 | 0.85 | 1.21 | 0.84 | 1.03 | 0.86 | 0.34 | 0.18 |
|  | VIT_206s0004g00332 | 0.02 | 0.25 | 0.28 | 0.00 | 0.06 | 0.31 | 0.08 | 0.00 | 0.00 | 0.00 | 0.09 | 0.00 | 0.00 | 0.00 |
|  | VIT_207s0005g04060 | 0.07 | 0.03 | 0.17 | 0.04 | 0.03 | 0.00 | 0.03 | 0.07 | 0.00 | 0.00 | 0.14 | 0.00 | 0.00 | 0.00 |
|  | VIT_207s0031g01850 | 40.47 | 37.17 | 35.07 | 37.01 | 37.76 | 22.55 | 27.03 | 30.59 | 28.04 | 30.00 | 30.03 | 25.01 | 24.38 | 23.81 |
|  | VIT_208s0058g00080 | 0.00 | 0.00 | 0.00 | 0.09 | 0.18 | 0.97 | 0.44 | 0.73 | 1.62 | 2.15 | 2.10 | 1.74 | 1.90 | 3.92 |
|  | VIT_209s0002g00080 | 4.84 | 3.92 | 4.49 | 4.04 | 3.92 | 3.48 | 4.51 | 5.18 | 4.51 | 6.20 | 6.02 | 2.81 | 3.23 | 2.49 |
|  | VIT_209s0002g07565 | 0.00 | 0.00 | 0.00 | 0.12 | 0.00 | 0.00 | 0.00 | 0.12 | 0.00 | 0.00 | 0.00 | 0.00 | 0.00 | 0.13 |
|  | VIT_209s0002g07570 | 0.03 | 0.00 | 0.00 | 0.00 | 0.00 | 0.00 | 0.04 | 0.08 | 0.00 | 0.00 | 0.00 | 0.00 | 0.00 | 0.00 |
|  | VIT_209s0002g07825 | 0.00 | 0.00 | 0.00 | 0.00 | 0.00 | 0.00 | 0.00 | 0.00 | 0.00 | 0.00 | 0.00 | 0.00 | 0.00 | 0.25 |
|  | VIT_209s0002g07830 | 0.01 | 0.00 | 0.04 | 0.00 | 0.00 | 0.00 | 0.00 | 0.12 | 0.15 | 0.04 | 0.00 | 0.04 | 0.00 | 0.00 |
|  | VIT_209s0018g00440 | 0.00 | 0.04 | 0.00 | 0.00 | 0.00 | 0.00 | 0.00 | 0.00 | 0.00 | 0.00 | 0.00 | 0.00 | 0.00 | 0.00 |
|  | VIT_209s0054g00050 | 0.00 | 0.00 | 0.00 | 0.00 | 0.00 | 0.00 | 0.00 | 0.00 | 0.09 | 0.00 | 0.00 | 0.00 | 0.00 | 0.10 |
|  | VIT_209s0070g00400 | 0.00 | 0.00 | 0.00 | 0.00 | 0.00 | 0.00 | 0.00 | 0.15 | 0.00 | 0.00 | 0.00 | 0.00 | 0.00 | 0.00 |
|  | VIT_209s0096g00800 | 0.00 | 0.00 | 0.00 | 0.00 | 0.00 | 0.00 | 0.00 | 0.00 | 0.00 | 0.00 | 0.00 | 0.04 | 0.00 | 0.00 |
|  | VIT_210s0003g01430 | 26.49 | 41.58 | 25.12 | 109.60 | 154.81 | 73.27 | 95.13 | 100.20 | 87.89 | 112.01 | 92.33 | 69.41 | 84.20 | 69.37 |
|  | VIT_210s0042g00590 | 0.23 | 0.25 | 0.04 | 0.22 | 0.06 | 0.03 | 0.03 | 0.00 | 0.03 | 0.00 | 0.13 | 0.03 | 0.07 | 0.00 |
|  | VIT_210s0042g00600 | 0.09 | 0.15 | 0.22 | 0.06 | 0.15 | 0.08 | 0.19 | 0.17 | 0.03 | 0.35 | 0.03 | 0.03 | 0.07 | 0.00 |
|  | VIT_210s0042g00610 | 0.60 | 0.37 | 0.85 | 0.67 | 0.32 | 0.58 | 0.61 | 0.59 | 0.86 | 1.17 | 1.28 | 0.82 | 1.48 | 1.72 |
|  | VIT_210s0042g00770 | 0.65 | 0.15 | 0.30 | 0.05 | 0.02 | 0.00 | 0.02 | 0.12 | 0.05 | 0.02 | 0.00 | 0.05 | 0.02 | 0.02 |
|  | VIT_211s0016g00040 | 1.04 | 0.77 | 3.16 | 0.66 | 0.30 | 0.58 | 0.60 | 1.04 | 0.72 | 0.70 | 0.73 | 0.65 | 0.44 | 0.84 |
|  | VIT_211s0037g00680 | 0.00 | 0.08 | 0.32 | 0.00 | 0.00 | 0.19 | 0.13 | 0.19 | 0.26 | 0.05 | 0.06 | 0.07 | 0.00 | 0.00 |
|  | VIT_211s0037g00770 | 0.00 | 0.05 | 0.07 | 0.05 | 0.00 | 0.00 | 0.00 | 0.00 | 0.00 | 0.00 | 0.00 | 0.00 | 0.00 | 0.00 |
|  | VIT_211s0118g00040 | 0.00 | 0.48 | 0.39 | 0.10 | 0.01 | 0.00 | 0.00 | 0.00 | 0.45 | 0.08 | 0.25 | 0.07 | 0.10 | 0.20 |
|  | VIT_212s0028g00340 | 0.00 | 0.00 | 0.00 | 0.00 | 0.00 | 0.00 | 0.00 | 0.00 | 0.00 | 0.04 | 0.00 | 0.00 | 0.00 | 0.00 |
|  | VIT_212s0028g03390 | 0.75 | 0.44 | 0.52 | 0.04 | 0.12 | 0.00 | 0.04 | 0.04 | 0.08 | 0.08 | 0.08 | 0.04 | 0.00 | 0.08 |
|  | VIT_212s0035g00070 | 1.09 | 0.00 | 0.07 | 0.46 | 0.00 | 0.05 | 0.22 | 0.11 | 0.00 | 0.04 | 0.30 | 0.00 | 0.03 | 0.13 |
|  | VIT_212s0035g00120 | 0.93 | 0.39 | 0.58 | 0.20 | 0.19 | 0.00 | 0.20 | 0.19 | 0.38 | 0.00 | 0.19 | 0.00 | 0.20 | 0.20 |
|  | VIT_212s0035g00140 | 0.21 | 0.15 | 0.19 | 0.00 | 0.00 | 0.00 | 0.15 | 0.04 | 0.00 | 0.00 | 0.00 | 0.00 | 0.00 | 0.00 |
|  | VIT_212s0035g00180 | 0.06 | 0.05 | 0.05 | 0.00 | 0.00 | 0.05 | 0.19 | 0.00 | 0.00 | 0.00 | 0.00 | 0.00 | 0.00 | 0.00 |
|  | VIT_212s0057g00760 | 0.07 | 0.04 | 0.00 | 0.04 | 0.04 | 0.11 | 0.11 | 0.07 | 0.04 | 0.04 | 0.04 | 0.00 | 0.00 | 0.00 |
|  | VIT_212s0057g00780 | 0.09 | 0.23 | 0.19 | 0.20 | 0.03 | 0.20 | 0.14 | 0.32 | 0.45 | 0.25 | 0.30 | 0.32 | 0.31 | 0.32 |
|  | VIT_212s0057g00810 | 0.00 | 0.55 | 0.00 | 0.00 | 0.00 | 0.00 | 0.00 | 0.00 | 0.00 | 0.00 | 0.00 | 0.00 | 0.00 | 0.00 |
|  | VIT_212s0057g00820 | 0.07 | 0.07 | 0.26 | 0.18 | 0.00 | 0.13 | 0.00 | 0.20 | 0.42 | 0.17 | 0.12 | 0.21 | 0.47 | 0.12 |
|  | VIT_212s0057g00870 | 0.00 | 0.10 | 0.05 | 0.00 | 0.05 | 0.00 | 0.00 | 0.10 | 0.00 | 0.05 | 0.00 | 0.00 | 0.00 | 0.00 |
|  | VIT_212s0142g00780 | 3.79 | 4.03 | 11.24 | 2.19 | 7.77 | 1.82 | 2.77 | 3.23 | 1.79 | 1.95 | 1.47 | 1.21 | 1.23 | 0.86 |
|  | VIT_213s0067g02030 | 0.69 | 0.16 | 0.15 | 0.04 | 0.17 | 0.00 | 0.04 | 0.04 | 0.04 | 0.06 | 0.10 | 0.04 | 0.04 | 0.02 |
|  | VIT_213s0067g03050 | 15.59 | 2.54 | 3.08 | 2.23 | 1.87 | 2.41 | 2.51 | 2.48 | 1.86 | 2.17 | 1.68 | 2.05 | 2.15 | 2.22 |
|  | VIT_214s0066g02250 | 66.16 | 47.63 | 54.21 | 35.03 | 36.92 | 26.58 | 29.26 | 30.58 | 16.02 | 19.16 | 18.50 | 7.80 | 8.47 | 10.02 |
|  | VIT_214s0066g02670 | 0.06 | 0.14 | 0.03 | 0.03 | 0.00 | 0.00 | 0.03 | 0.00 | 0.00 | 0.00 | 0.08 | 0.03 | 0.03 | 0.03 |
|  | VIT_214s0066g02680 | 0.33 | 0.23 | 0.26 | 0.14 | 0.02 | 0.00 | 0.02 | 0.05 | 0.14 | 0.00 | 0.00 | 0.00 | 0.00 | 0.00 |
|  | VIT_214s0068g02250 | 0.08 | 0.11 | 0.19 | 0.10 | 0.00 | 0.00 | 0.00 | 0.00 | 0.00 | 0.00 | 0.00 | 0.00 | 0.00 | 0.00 |
|  | VIT_214s0068g02270 | 0.26 | 0.99 | 0.43 | 0.62 | 0.14 | 0.00 | 0.10 | 0.00 | 0.14 | 0.48 | 0.28 | 0.00 | 0.07 | 0.00 |
|  | VIT_214s0068g02300 | 0.04 | 0.00 | 0.07 | 0.04 | 0.00 | 0.00 | 0.00 | 0.04 | 0.00 | 0.00 | 0.00 | 0.00 | 0.00 | 0.00 |
|  | VIT_214s0108g00300 | 0.05 | 0.00 | 0.00 | 0.00 | 0.00 | 0.00 | 0.02 | 0.00 | 0.07 | 0.00 | 0.03 | 0.00 | 0.00 | 0.00 |
|  | VIT_214s0108g00310 | 0.11 | 0.08 | 0.07 | 0.08 | 0.00 | 0.00 | 0.00 | 0.00 | 0.04 | 0.00 | 0.00 | 0.00 | 0.00 | 0.00 |
|  | VIT_214s0108g00370 | 2.26 | 1.87 | 2.24 | 1.24 | 1.86 | 1.59 | 0.91 | 1.17 | 1.49 | 1.29 | 1.31 | 1.06 | 1.14 | 0.96 |
|  | VIT_214s0171g00015 | 0.17 | 0.11 | 0.00 | 0.00 | 0.00 | 0.00 | 0.00 | 0.05 | 0.00 | 0.00 | 0.10 | 0.00 | 0.00 | 0.00 |
|  | VIT_215s0024g01750 | 5.28 | 3.88 | 3.18 | 5.28 | 4.91 | 5.57 | 4.23 | 5.66 | 8.60 | 7.13 | 5.43 | 8.16 | 8.39 | 5.40 |
|  | VIT_216s0013g01500 | 0.10 | 0.21 | 0.21 | 0.04 | 0.00 | 0.00 | 0.07 | 0.04 | 0.18 | 0.17 | 0.04 | 0.29 | 0.00 | 0.11 |
|  | VIT_216s0013g01710 | 0.00 | 0.00 | 0.12 | 0.51 | 0.22 | 0.16 | 0.51 | 0.00 | 0.76 | 0.80 | 0.06 | 0.94 | 0.50 | 0.30 |
|  | VIT_216s0013g01940 | 3.28 | 2.19 | 1.68 | 10.70 | 6.02 | 13.80 | 11.35 | 9.29 | 17.03 | 15.90 | 18.78 | 9.14 | 9.20 | 10.25 |
|  | VIT_216s0022g00110 | 1.61 | 0.86 | 0.46 | 0.87 | 0.60 | 1.18 | 0.73 | 1.35 | 0.44 | 0.22 | 0.48 | 0.35 | 0.33 | 0.55 |
|  | VIT_216s0022g00240 | 3.95 | 2.34 | 3.61 | 3.21 | 2.25 | 1.87 | 2.92 | 2.24 | 1.49 | 1.71 | 0.82 | 0.51 | 0.50 | 0.88 |
|  | VIT_216s0022g00270 | 1.47 | 2.48 | 5.23 | 4.39 | 4.56 | 6.80 | 5.59 | 5.69 | 3.49 | 9.35 | 3.51 | 4.50 | 3.49 | 4.85 |
|  | VIT_216s0050g01740 | 0.00 | 0.08 | 0.00 | 0.00 | 0.00 | 0.00 | 0.08 | 0.00 | 0.00 | 0.00 | 0.00 | 0.00 | 0.00 | 0.00 |
|  | VIT_216s0100g00710 | 0.50 | 0.06 | 0.23 | 0.08 | 0.03 | 0.03 | 0.01 | 0.03 | 0.03 | 0.00 | 0.09 | 0.00 | 0.03 | 0.00 |
|  | VIT_217s0000g06710 | 7.40 | 7.76 | 12.01 | 3.76 | 3.29 | 5.91 | 5.14 | 5.90 | 10.75 | 12.04 | 12.54 | 9.09 | 9.18 | 10.45 |
|  | VIT_217s0053g00390 | 0.00 | 0.00 | 0.07 | 0.00 | 0.00 | 0.00 | 0.00 | 0.00 | 0.00 | 0.00 | 0.00 | 0.00 | 0.00 | 0.00 |
|  | VIT_218s0001g12520 | 25.95 | 42.19 | 49.12 | 18.72 | 23.67 | 11.98 | 15.00 | 17.14 | 8.22 | 10.11 | 13.90 | 4.59 | 4.86 | 5.81 |
|  | VIT_218s0089g00650 | 0.86 | 0.15 | 0.15 | 0.42 | 0.20 | 0.31 | 0.20 | 0.32 | 0.37 | 0.36 | 0.56 | 0.29 | 0.14 | 0.12 |
|  | VIT_218s0089g00660 | 11.91 | 2.05 | 2.87 | 5.24 | 3.89 | 5.46 | 5.37 | 6.13 | 4.72 | 7.09 | 6.21 | 4.14 | 3.84 | 2.71 |
|  | VIT_218s0089g00680 | 0.30 | 0.16 | 0.17 | 0.32 | 0.52 | 0.38 | 0.00 | 0.56 | 0.81 | 0.95 | 1.33 | 0.60 | 0.39 | 0.80 |
|  | VIT_219s0014g02360 | 5.03 | 3.23 | 4.13 | 2.84 | 2.34 | 2.01 | 2.39 | 2.87 | 5.17 | 5.27 | 4.22 | 2.82 | 2.73 | 2.62 |
|  | VIT_219s0015g01890 | 6.54 | 3.72 | 4.58 | 2.71 | 1.64 | 7.52 | 6.25 | 7.38 | 10.88 | 12.55 | 12.73 | 18.44 | 19.59 | 21.61 |
|  | VIT_219s0015g02410 | 11.94 | 6.98 | 9.22 | 11.31 | 12.52 | 7.76 | 9.78 | 11.18 | 6.08 | 9.67 | 7.68 | 6.27 | 6.11 | 5.00 |
|  | VIT_219s0085g00150 | 0.00 | 0.00 | 0.00 | 0.00 | 0.14 | 0.00 | 0.00 | 0.00 | 0.00 | 0.00 | 0.00 | 0.00 | 0.00 | 0.00 |
|  | VIT_218s0001g10690 | 25.97 | 27.06 | 15.50 | 6.10 | 5.82 | 2.52 | 2.10 | 3.06 | 5.09 | 3.30 | 2.84 | 3.89 | 2.30 | 1.94 |
|  | VIT_202s0012g01140 | 3.50 | 1.34 | 2.29 | 1.01 | 1.67 | 1.54 | 1.69 | 1.62 | 1.15 | 1.76 | 1.19 | 0.76 | 0.72 | 0.77 |
|  | VIT_202s0025g03550 | 11.62 | 17.59 | 22.32 | 6.88 | 9.97 | 6.29 | 7.75 | 8.75 | 7.33 | 5.79 | 8.07 | 8.14 | 7.94 | 8.87 |
|  | VIT_203s0038g03270 | 39.30 | 29.98 | 27.45 | 55.12 | 52.80 | 31.92 | 34.16 | 32.27 | 25.13 | 27.69 | 27.14 | 21.55 | 22.31 | 23.72 |
|  | VIT_203s0097g00660 | 15.08 | 13.58 | 11.88 | 11.54 | 12.51 | 13.09 | 11.93 | 11.98 | 19.20 | 17.03 | 16.86 | 23.50 | 22.36 | 22.75 |
|  | VIT_206s0004g05810 | 20.65 | 18.48 | 16.94 | 41.30 | 40.34 | 26.50 | 33.39 | 32.27 | 12.75 | 15.63 | 13.57 | 9.34 | 9.48 | 7.35 |
|  | VIT_208s0007g01520 | 16.48 | 22.99 | 16.28 | 31.97 | 40.68 | 45.42 | 48.78 | 44.68 | 17.53 | 24.27 | 17.42 | 7.62 | 6.92 | 7.67 |
|  | VIT_215s0046g02900 | 20.75 | 34.89 | 32.93 | 22.01 | 23.76 | 27.23 | 31.07 | 30.72 | 21.35 | 22.53 | 22.05 | 20.00 | 19.55 | 21.41 |
|  | VIT_217s0000g08700 | 0.11 | 0.00 | 0.00 | 0.00 | 0.00 | 0.00 | 0.00 | 0.00 | 0.00 | 0.00 | 0.00 | 0.00 | 0.00 | 0.00 |
|  | VIT_218s0001g00180 | 43.69 | 55.93 | 47.67 | 40.84 | 40.75 | 39.15 | 34.06 | 36.69 | 42.80 | 36.32 | 42.67 | 41.33 | 38.49 | 39.95 |
| BSK | VIT_210s0003g01480 | 191.81 | 318.21 | 263.93 | 298.32 | 316.69 | 283.34 | 300.84 | 280.20 | 242.90 | 271.05 | 227.70 | 229.36 | 249.48 | 231.93 |
|  | VIT_212s0028g01810 | 81.50 | 104.21 | 100.35 | 78.75 | 78.83 | 78.72 | 79.58 | 88.68 | 81.25 | 82.69 | 81.85 | 91.18 | 95.83 | 91.80 |
| BIN2 | VIT_204s0023g01250 | 62.46 | 154.29 | 130.52 | 76.48 | 84.20 | 63.59 | 75.60 | 74.82 | 69.26 | 80.33 | 70.06 | 39.91 | 41.36 | 40.85 |
|  | VIT_210s0003g01790 | 7.13 | 3.62 | 4.66 | 8.33 | 6.77 | 6.40 | 7.69 | 10.53 | 9.58 | 10.87 | 12.83 | 9.19 | 9.04 | 8.19 |
|  | VIT_210s0003g04710 | 0.60 | 0.79 | 0.52 | 0.27 | 0.26 | 0.52 | 0.77 | 1.15 | 0.98 | 0.70 | 0.72 | 0.78 | 0.62 | 0.73 |
|  | VIT_218s0001g12020 | 14.49 | 11.02 | 8.57 | 10.18 | 9.55 | 6.04 | 6.01 | 7.21 | 4.10 | 3.78 | 3.60 | 3.83 | 2.50 | 3.01 |
| BZR1/2 | VIT_211s0052g01190 | 40.95 | 15.53 | 14.22 | 38.30 | 46.90 | 45.14 | 38.82 | 28.23 | 14.73 | 15.91 | 21.72 | 2.67 | 2.17 | 3.53 |
|  | VIT_211s0052g01230 | 0.67 | 42.17 | 357.42 | 0.13 | 6.16 | 0.18 | 0.18 | 20.76 | 0.44 | 0.60 | 6.64 | 0.12 | 1.10 | 1.02 |
|  | VIT_211s0052g01330 | 12.30 | 8.69 | 6.13 | 5.64 | 6.97 | 2.91 | 4.25 | 2.32 | 2.58 | 1.26 | 3.31 | 0.00 | 0.00 | 0.71 |
| TCH4 | VIT_200s0194g00280 | 0.48 | 0.84 | 0.82 | 0.35 | 0.06 | 0.21 | 0.36 | 0.20 | 0.14 | 0.05 | 0.27 | 0.07 | 0.04 | 0.17 |
|  | VIT_200s0203g00160 | 4.20 | 1.43 | 1.66 | 0.13 | 0.17 | 0.00 | 0.04 | 0.17 | 0.09 | 0.04 | 0.00 | 0.08 | 0.04 | 0.05 |
|  | VIT_203s0097g00610 | 0.17 | 0.00 | 0.57 | 0.00 | 0.00 | 0.16 | 0.16 | 0.00 | 0.18 | 0.00 | 0.00 | 0.00 | 0.00 | 0.00 |
|  | VIT_203s0180g00040 | 12.99 | 4.74 | 6.14 | 16.21 | 8.04 | 10.88 | 11.78 | 8.67 | 8.02 | 7.83 | 7.44 | 1.46 | 0.89 | 1.97 |
|  | VIT_205s0094g00110 | 0.14 | 0.07 | 0.13 | 0.07 | 0.00 | 0.00 | 0.00 | 0.00 | 0.07 | 0.00 | 0.00 | 0.00 | 0.00 | 0.00 |
|  | VIT_207s0129g00200 | 10.01 | 3.31 | 2.99 | 0.47 | 0.75 | 0.30 | 0.40 | 0.26 | 0.40 | 0.31 | 0.42 | 0.21 | 0.26 | 0.16 |
|  | VIT_207s0129g01100 | 3.28 | 0.34 | 0.56 | 0.20 | 0.27 | 0.39 | 0.15 | 0.43 | 0.68 | 0.24 | 0.47 | 0.31 | 0.12 | 0.06 |
|  | VIT_208s0007g00790 | 0.00 | 0.00 | 0.00 | 0.00 | 0.00 | 0.12 | 0.12 | 0.00 | 0.00 | 0.00 | 0.00 | 0.00 | 0.00 | 0.00 |
|  | VIT_208s0105g00530 | 0.57 | 0.00 | 0.00 | 0.16 | 0.33 | 0.09 | 0.18 | 0.00 | 0.00 | 0.10 | 0.00 | 0.12 | 0.00 | 0.00 |
|  | VIT_212s0134g00500 | 0.29 | 0.00 | 0.17 | 0.04 | 0.08 | 0.00 | 0.08 | 0.16 | 0.00 | 0.04 | 0.00 | 0.00 | 0.00 | 0.00 |
|  | VIT_213s0101g00515 | 0.00 | 0.00 | 0.19 | 0.38 | 0.20 | 0.57 | 0.20 | 0.19 | 0.00 | 0.00 | 0.00 | 0.19 | 0.00 | 0.00 |
|  | VIT_218s0001g01240 | 6.49 | 5.91 | 4.51 | 3.71 | 3.91 | 3.17 | 3.13 | 3.62 | 3.92 | 3.88 | 3.34 | 4.06 | 4.64 | 3.84 |
|  | VIT_218s0001g09160 | 0.59 | 0.48 | 0.62 | 0.23 | 0.19 | 0.30 | 0.41 | 0.53 | 0.18 | 0.45 | 0.26 | 0.23 | 0.09 | 0.18 |
|  | VIT_218s0001g09920 | 7.10 | 0.35 | 0.61 | 0.12 | 0.26 | 0.08 | 0.09 | 0.23 | 0.06 | 0.00 | 0.06 | 0.00 | 0.03 | 0.00 |
| CYCD3 | VIT_215s0046g01280 | 18.50 | 27.98 | 29.18 | 26.06 | 17.47 | 27.79 | 24.70 | 32.59 | 48.33 | 39.09 | 40.37 | 38.53 | 36.37 | 38.77 |
| JAR1 | VIT_213s0019g03380 | 35.33 | 29.81 | 31.50 | 49.41 | 43.11 | 60.88 | 58.21 | 61.79 | 73.56 | 77.66 | 75.49 | 86.87 | 89.64 | 95.16 |
| COI1 | VIT_200s0179g00090 | 14.59 | 11.08 | 11.87 | 16.01 | 12.15 | 13.41 | 10.65 | 10.74 | 16.11 | 13.43 | 12.91 | 21.86 | 16.69 | 19.32 |
|  | VIT_201s0011g05560 | 83.99 | 204.87 | 180.91 | 79.65 | 91.32 | 73.88 | 77.73 | 80.64 | 65.77 | 53.15 | 57.21 | 55.70 | 53.54 | 56.93 |
|  | VIT_201s0146g00480 | 16.80 | 75.49 | 90.82 | 3.03 | 8.48 | 1.40 | 1.77 | 7.09 | 0.49 | 0.26 | 6.17 | 0.18 | 0.09 | 0.30 |
|  | VIT_204s0008g00110 | 0.19 | 0.34 | 2.95 | 0.38 | 0.00 | 0.00 | 0.00 | 1.43 | 0.46 | 0.00 | 0.00 | 0.21 | 0.18 | 0.00 |
|  | VIT_204s0008g04950 | 10.26 | 6.86 | 8.30 | 8.26 | 8.86 | 11.56 | 10.39 | 11.05 | 8.91 | 9.77 | 9.71 | 6.45 | 7.32 | 7.52 |
|  | VIT_209s0002g00890 | 67.71 | 397.28 | 381.81 | 56.79 | 100.54 | 33.13 | 48.18 | 53.52 | 87.72 | 56.50 | 79.25 | 66.10 | 56.86 | 58.81 |
|  | VIT_210s0003g03790 | 9.29 | 75.56 | 48.59 | 25.45 | 31.66 | 24.43 | 21.35 | 21.02 | 74.01 | 54.18 | 47.29 | 29.12 | 26.01 | 23.53 |
|  | VIT_210s0003g03800 | 40.99 | 56.50 | 38.78 | 18.47 | 43.08 | 10.36 | 20.28 | 15.84 | 30.16 | 14.32 | 13.60 | 9.21 | 7.22 | 10.99 |
|  | VIT_211s0016g00710 | 109.34 | 187.92 | 179.56 | 118.35 | 154.25 | 83.34 | 102.94 | 92.96 | 59.97 | 59.05 | 79.18 | 45.71 | 40.43 | 46.42 |
|  | VIT_212s0035g00900 | 5.50 | 3.61 | 4.37 | 0.35 | 0.25 | 0.11 | 0.00 | 0.10 | 0.00 | 0.00 | 0.00 | 0.06 | 0.00 | 0.00 |
|  | VIT_217s0000g02230 | 26.30 | 21.55 | 22.04 | 13.82 | 12.64 | 12.79 | 15.55 | 13.28 | 15.88 | 15.17 | 13.62 | 21.31 | 18.62 | 18.98 |
| JAZ | VIT_218s0001g07410 | 4.63 | 2.51 | 4.13 | 0.91 | 1.26 | 1.18 | 1.35 | 2.55 | 1.55 | 2.48 | 1.97 | 1.61 | 2.43 | 3.09 |
|  | VIT_200s0271g00085 | 0.00 | 0.00 | 0.00 | 0.00 | 0.00 | 0.00 | 0.00 | 0.00 | 0.00 | 0.00 | 0.11 | 0.00 | 0.00 | 0.00 |
|  | VIT_200s0274g00045 | 0.12 | 0.00 | 0.00 | 0.00 | 0.00 | 0.00 | 0.00 | 0.00 | 0.00 | 0.00 | 0.00 | 0.00 | 0.00 | 0.00 |
|  | VIT_200s0532g00050 | 10.90 | 10.97 | 8.67 | 8.29 | 8.38 | 11.26 | 8.53 | 7.19 | 10.39 | 4.71 | 8.65 | 4.84 | 3.44 | 4.11 |
|  | VIT_200s0824g00020 | 0.41 | 1.08 | 0.81 | 0.00 | 0.38 | 0.06 | 0.00 | 0.14 | 0.00 | 0.00 | 0.27 | 0.00 | 0.00 | 0.00 |
|  | VIT_200s0899g00005 | 2.30 | 2.89 | 2.54 | 0.32 | 0.45 | 0.25 | 0.11 | 0.00 | 0.00 | 0.02 | 0.18 | 0.00 | 0.00 | 0.10 |
|  | VIT_200s0927g00010 | 0.36 | 0.61 | 1.02 | 0.00 | 0.00 | 0.00 | 0.00 | 0.23 | 0.00 | 0.00 | 0.00 | 0.00 | 0.00 | 0.00 |
|  | VIT_200s1312g00010 | 50.48 | 3.62 | 9.02 | 1.34 | 1.88 | 0.31 | 0.51 | 1.31 | 0.65 | 0.39 | 0.21 | 0.24 | 0.28 | 0.27 |
|  | VIT_200s1314g00010 | 2.38 | 3.74 | 3.05 | 0.26 | 0.40 | 0.12 | 0.29 | 0.27 | 0.00 | 0.11 | 0.10 | 0.00 | 0.00 | 0.00 |
|  | VIT_201s0127g00650 | 0.11 | 0.51 | 0.52 | 0.10 | 0.20 | 0.10 | 0.10 | 0.21 | 0.41 | 0.20 | 0.32 | 0.61 | 0.51 | 1.05 |
|  | VIT_201s0127g00860 | 0.62 | 0.00 | 0.07 | 0.00 | 0.00 | 0.00 | 0.00 | 0.00 | 0.00 | 0.00 | 0.00 | 0.00 | 0.00 | 0.00 |
|  | VIT_202s0012g01320 | 72.87 | 185.42 | 227.15 | 39.63 | 52.46 | 29.03 | 34.58 | 52.13 | 47.37 | 43.47 | 60.68 | 40.69 | 43.88 | 46.09 |
|  | VIT_202s0012g01450 | 16.43 | 13.57 | 11.88 | 3.41 | 6.40 | 4.37 | 4.05 | 3.21 | 1.11 | 1.57 | 1.11 | 0.37 | 0.61 | 0.47 |
|  | VIT_202s0025g02610 | 0.08 | 4.60 | 7.19 | 0.04 | 0.15 | 0.04 | 0.11 | 0.00 | 0.00 | 0.00 | 0.08 | 0.04 | 0.04 | 0.00 |
|  | VIT_202s0025g03450 | 2.55 | 1.63 | 2.54 | 0.42 | 0.43 | 0.24 | 0.31 | 0.59 | 0.19 | 0.46 | 0.16 | 0.15 | 0.24 | 0.29 |
|  | VIT_203s0038g02540 | 1.94 | 0.06 | 0.66 | 0.03 | 0.03 | 0.00 | 0.00 | 0.00 | 0.00 | 0.00 | 0.00 | 0.00 | 0.00 | 0.00 |
|  | VIT_203s0063g00170 | 0.88 | 0.13 | 1.12 | 0.14 | 0.23 | 0.13 | 0.03 | 0.28 | 0.17 | 0.10 | 0.24 | 0.20 | 0.14 | 0.22 |
|  | VIT_203s0088g01240 | 0.49 | 0.03 | 0.24 | 0.00 | 0.00 | 0.00 | 0.00 | 0.33 | 0.00 | 0.03 | 0.03 | 0.00 | 0.00 | 0.00 |
|  | VIT_205s0020g02700 | 0.00 | 0.45 | 2.55 | 0.00 | 0.75 | 0.00 | 0.00 | 0.00 | 0.00 | 0.11 | 0.00 | 0.11 | 0.00 | 0.12 |
|  | VIT_205s0049g00460 | 25.10 | 40.34 | 34.84 | 11.07 | 16.83 | 9.53 | 9.18 | 11.66 | 8.11 | 9.11 | 12.69 | 13.25 | 8.89 | 9.16 |
|  | VIT_206s0061g00720 | 0.00 | 0.00 | 0.00 | 0.08 | 0.00 | 0.08 | 0.04 | 0.00 | 0.00 | 0.08 | 0.00 | 0.00 | 0.04 | 0.00 |
|  | VIT_207s0104g00090 | 35.54 | 21.93 | 25.11 | 21.34 | 23.58 | 11.85 | 16.61 | 15.66 | 10.67 | 13.33 | 11.33 | 17.88 | 21.27 | 16.27 |
|  | VIT_207s0141g01060 | 29.11 | 39.87 | 38.01 | 24.28 | 24.79 | 23.31 | 22.93 | 25.43 | 28.43 | 27.23 | 27.51 | 24.56 | 23.92 | 26.07 |
|  | VIT_207s0205g00160 | 0.00 | 0.00 | 0.00 | 0.00 | 0.03 | 0.00 | 0.00 | 0.00 | 0.00 | 0.00 | 0.00 | 0.00 | 0.03 | 0.03 |
|  | VIT_207s0205g00180 | 0.30 | 0.06 | 0.18 | 0.00 | 0.00 | 0.00 | 0.00 | 0.00 | 0.00 | 0.00 | 0.00 | 0.00 | 0.00 | 0.00 |
|  | VIT_207s0205g00190 | 0.30 | 0.30 | 0.79 | 0.07 | 0.17 | 0.12 | 0.06 | 0.60 | 0.00 | 0.12 | 0.31 | 0.00 | 0.06 | 0.00 |
|  | VIT_208s0040g01240 | 1.45 | 1.54 | 1.83 | 0.83 | 1.35 | 0.43 | 0.86 | 1.19 | 1.03 | 0.94 | 0.82 | 1.17 | 0.99 | 1.13 |
|  | VIT_210s0003g01160 | 3.85 | 15.72 | 12.95 | 1.35 | 1.56 | 0.88 | 1.22 | 3.62 | 3.27 | 2.16 | 3.65 | 2.70 | 1.81 | 2.33 |
|  | VIT_211s0016g02070 | 2.08 | 3.14 | 5.35 | 0.65 | 0.43 | 0.68 | 0.55 | 2.13 | 0.36 | 0.36 | 1.32 | 0.11 | 0.12 | 0.37 |
|  | VIT_211s0052g00100 | 187.58 | 262.37 | 326.61 | 41.54 | 67.99 | 14.18 | 18.83 | 44.76 | 5.55 | 9.84 | 42.56 | 2.71 | 2.81 | 6.76 |
|  | VIT_212s0028g02350 | 4.22 | 13.73 | 11.57 | 4.24 | 5.78 | 1.38 | 2.72 | 3.47 | 2.52 | 1.39 | 1.61 | 1.26 | 0.91 | 0.90 |
|  | VIT_212s0059g02650 | 24.71 | 18.54 | 16.27 | 14.70 | 15.09 | 12.56 | 11.27 | 12.38 | 14.73 | 11.79 | 12.76 | 12.03 | 12.30 | 12.66 |
|  | VIT_214s0068g01200 | 8.01 | 7.91 | 13.16 | 4.81 | 6.30 | 3.51 | 3.54 | 6.84 | 7.59 | 5.07 | 8.34 | 9.85 | 8.21 | 8.44 |
|  | VIT_214s0068g01580 | 0.31 | 0.06 | 0.08 | 0.06 | 0.00 | 0.00 | 0.03 | 0.00 | 0.03 | 0.00 | 0.00 | 0.00 | 0.00 | 0.06 |
|  | VIT_214s0128g00110 | 4.61 | 1.28 | 1.38 | 0.07 | 0.10 | 0.00 | 0.03 | 0.23 | 0.03 | 0.06 | 0.00 | 0.03 | 0.03 | 0.10 |
|  | VIT_215s0046g00320 | 51.46 | 46.65 | 58.32 | 22.18 | 32.16 | 16.30 | 20.39 | 28.64 | 12.46 | 12.17 | 20.36 | 10.85 | 11.08 | 12.80 |
|  | VIT_215s0046g02560 | 13.93 | 3.58 | 4.54 | 4.76 | 3.98 | 4.28 | 4.03 | 5.08 | 3.91 | 3.74 | 4.32 | 3.47 | 4.09 | 4.57 |
|  | VIT_215s0048g02510 | 0.02 | 0.00 | 0.04 | 0.00 | 0.00 | 0.00 | 0.00 | 0.00 | 0.00 | 0.00 | 0.00 | 0.00 | 0.02 | 0.02 |
|  | VIT_215s0048g02820 | 1.63 | 0.40 | 0.51 | 0.04 | 0.04 | 0.00 | 0.00 | 0.04 | 0.00 | 0.00 | 0.00 | 0.04 | 0.00 | 0.00 |
|  | VIT_215s0107g00380 | 0.57 | 0.04 | 0.20 | 0.00 | 0.04 | 0.04 | 0.00 | 0.00 | 0.00 | 0.00 | 0.00 | 0.00 | 0.00 | 0.00 |
|  | VIT_217s0000g00330 | 2.32 | 0.66 | 1.09 | 0.17 | 0.14 | 0.35 | 0.28 | 0.29 | 0.32 | 0.20 | 0.51 | 0.60 | 0.43 | 0.44 |
|  | VIT_217s0000g03550 | 1.02 | 0.09 | 0.00 | 0.04 | 0.00 | 0.05 | 0.00 | 0.00 | 0.00 | 0.00 | 0.00 | 0.00 | 0.00 | 0.00 |
|  | VIT_218s0001g08040 | 1.53 | 5.99 | 5.18 | 0.27 | 0.32 | 0.29 | 0.20 | 0.59 | 0.60 | 0.33 | 0.44 | 0.10 | 0.17 | 0.14 |
|  | VIT_218s0001g10300 | 1.75 | 0.64 | 2.43 | 0.58 | 0.38 | 0.45 | 0.22 | 1.19 | 4.22 | 2.16 | 3.97 | 4.56 | 3.73 | 3.15 |
|  | VIT_219s0014g05100 | 110.84 | 104.77 | 112.56 | 83.93 | 98.83 | 71.04 | 70.30 | 82.53 | 84.25 | 76.01 | 74.76 | 81.11 | 81.23 | 81.70 |
| MYC2 | VIT_208s0007g05740 | 0.53 | 0.28 | 0.33 | 0.21 | 0.15 | 0.17 | 0.22 | 0.55 | 0.64 | 0.30 | 0.72 | 0.57 | 0.28 | 0.81 |
|  | VIT_210s0042g01250 | 28.17 | 46.20 | 47.92 | 16.19 | 20.22 | 13.57 | 15.26 | 18.15 | 13.13 | 13.20 | 16.70 | 15.76 | 16.93 | 16.40 |
|  | VIT_211s0016g01990 | 14.53 | 22.27 | 28.09 | 17.72 | 19.79 | 22.26 | 21.46 | 24.15 | 36.48 | 31.76 | 35.88 | 37.63 | 37.78 | 37.59 |
| NPR1 | VIT_200s0183g00210 | 0.13 | 0.20 | 0.03 | 0.01 | 0.16 | 0.06 | 0.05 | 0.01 | 0.00 | 0.06 | 0.06 | 0.00 | 0.00 | 0.00 |
|  | VIT_200s0192g00020 | 0.08 | 0.00 | 0.08 | 0.00 | 0.00 | 0.25 | 0.08 | 0.48 | 0.31 | 0.25 | 0.17 | 0.08 | 0.09 | 0.26 |
|  | VIT_200s0524g00030 | 50.58 | 69.30 | 40.31 | 145.79 | 191.31 | 95.83 | 118.07 | 103.26 | 44.07 | 51.97 | 56.45 | 35.20 | 36.90 | 38.56 |
|  | VIT_201s0010g02890 | 12.12 | 13.71 | 14.06 | 5.66 | 7.37 | 3.79 | 5.52 | 6.27 | 3.51 | 3.95 | 3.84 | 2.46 | 2.57 | 2.55 |
|  | VIT_201s0011g03230 | 0.07 | 0.02 | 0.09 | 0.00 | 0.00 | 0.00 | 0.02 | 0.23 | 0.02 | 0.14 | 0.23 | 0.02 | 0.05 | 0.10 |
|  | VIT_201s0011g04770 | 10.17 | 6.30 | 6.57 | 8.31 | 8.63 | 9.63 | 10.85 | 7.36 | 6.14 | 7.26 | 6.71 | 1.54 | 1.20 | 1.45 |
|  | VIT_201s0026g00190 | 2.79 | 4.47 | 3.73 | 2.72 | 4.71 | 3.78 | 3.09 | 9.96 | 10.47 | 9.55 | 13.17 | 10.32 | 11.92 | 11.93 |
|  | VIT_206s0004g06993 | 0.13 | 0.00 | 0.00 | 0.00 | 0.00 | 0.00 | 0.00 | 0.06 | 0.00 | 0.00 | 0.00 | 0.00 | 0.00 | 0.00 |
|  | VIT_206s0080g00360 | 0.88 | 0.44 | 1.39 | 0.87 | 0.77 | 1.11 | 0.94 | 4.50 | 4.14 | 3.44 | 5.63 | 2.81 | 3.03 | 3.73 |
|  | VIT_207s0031g01320 | 12.28 | 17.77 | 17.95 | 3.99 | 6.61 | 2.89 | 3.59 | 5.42 | 3.72 | 4.18 | 4.51 | 6.13 | 7.29 | 5.43 |
|  | VIT_207s0031g02670 | 0.00 | 0.00 | 0.36 | 0.07 | 0.07 | 0.07 | 0.00 | 4.67 | 0.22 | 1.23 | 4.74 | 0.14 | 1.54 | 2.23 |
|  | VIT_207s0129g00590 | 41.14 | 33.88 | 36.85 | 27.14 | 28.88 | 24.31 | 26.15 | 32.16 | 33.30 | 32.05 | 33.62 | 38.11 | 36.13 | 36.56 |
|  | VIT_208s0007g05170 | 17.00 | 20.01 | 24.27 | 13.05 | 13.51 | 14.08 | 13.56 | 17.29 | 19.30 | 18.50 | 20.74 | 22.54 | 23.56 | 22.51 |
|  | VIT_208s0007g06160 | 0.58 | 3.13 | 17.21 | 0.24 | 2.35 | 0.57 | 1.04 | 23.36 | 1.44 | 1.70 | 16.12 | 0.56 | 3.08 | 4.65 |
|  | VIT_208s0058g01100 | 7.65 | 16.85 | 12.95 | 14.14 | 16.37 | 14.20 | 14.88 | 13.19 | 6.94 | 8.78 | 11.11 | 3.07 | 2.08 | 2.76 |
|  | VIT_210s0003g01405 | 0.16 | 1.45 | 11.46 | 0.26 | 1.61 | 0.32 | 0.32 | 5.51 | 0.00 | 0.32 | 2.55 | 0.00 | 0.58 | 0.34 |
|  | VIT_210s0003g01730 | 61.06 | 42.77 | 39.21 | 89.90 | 107.62 | 72.90 | 85.58 | 80.95 | 31.43 | 54.84 | 54.35 | 16.92 | 15.30 | 20.33 |
|  | VIT_213s0019g04710 | 35.49 | 31.54 | 32.95 | 22.66 | 27.85 | 12.50 | 16.17 | 16.90 | 8.24 | 7.43 | 12.75 | 10.28 | 9.24 | 10.62 |
|  | VIT_213s0067g01156 | 0.00 | 0.00 | 0.00 | 0.00 | 0.00 | 0.00 | 0.00 | 0.53 | 0.00 | 0.09 | 0.61 | 0.00 | 0.00 | 0.27 |
|  | VIT_213s0067g01168 | 0.00 | 0.00 | 0.00 | 0.00 | 0.00 | 0.00 | 0.00 | 0.09 | 0.00 | 0.00 | 0.09 | 0.00 | 0.00 | 0.00 |
|  | VIT_213s0067g01174 | 0.00 | 0.00 | 0.09 | 0.00 | 0.00 | 0.00 | 0.00 | 0.17 | 0.00 | 0.00 | 0.08 | 0.00 | 0.00 | 0.00 |
|  | VIT_213s0067g01460 | 0.23 | 0.77 | 0.50 | 1.49 | 1.50 | 0.32 | 0.87 | 0.88 | 0.63 | 0.32 | 0.56 | 0.77 | 0.47 | 0.52 |
|  | VIT_213s0084g00660 | 41.92 | 34.69 | 44.40 | 29.65 | 32.55 | 29.45 | 29.37 | 35.38 | 38.94 | 36.25 | 40.22 | 36.62 | 39.27 | 38.18 |
|  | VIT_213s0084g00810 | 13.05 | 39.58 | 23.99 | 11.42 | 13.19 | 9.87 | 12.51 | 13.38 | 15.18 | 15.62 | 16.82 | 16.71 | 16.68 | 16.37 |
|  | VIT_214s0006g00700 | 6.34 | 2.00 | 1.73 | 2.03 | 2.76 | 0.78 | 1.01 | 0.94 | 0.23 | 0.04 | 0.18 | 0.20 | 0.04 | 0.09 |
|  | VIT_214s0006g00760 | 0.00 | 0.00 | 0.09 | 0.00 | 0.00 | 0.00 | 0.00 | 0.00 | 0.00 | 0.00 | 0.00 | 0.00 | 0.00 | 0.00 |
|  | VIT_217s0000g08080 | 4.24 | 2.57 | 1.20 | 6.82 | 8.99 | 10.82 | 13.80 | 15.25 | 3.91 | 7.76 | 11.48 | 1.37 | 1.61 | 3.67 |
|  | VIT_218s0001g04470 | 10.67 | 12.94 | 11.05 | 5.06 | 6.08 | 3.66 | 4.83 | 5.28 | 5.54 | 5.01 | 5.18 | 3.25 | 3.18 | 3.25 |
|  | VIT_219s0014g01210 | 0.46 | 0.70 | 5.78 | 0.08 | 0.45 | 0.15 | 0.45 | 4.64 | 0.16 | 0.15 | 2.37 | 0.24 | 0.63 | 1.37 |
|  | VIT_219s0014g01220 | 0.05 | 0.17 | 2.88 | 0.06 | 1.25 | 0.22 | 0.43 | 11.32 | 0.92 | 0.55 | 6.94 | 0.50 | 2.26 | 2.10 |
|  | VIT_219s0014g01450 | 0.29 | 1.84 | 10.37 | 0.00 | 0.43 | 0.00 | 0.00 | 2.84 | 0.23 | 0.15 | 2.04 | 0.66 | 0.52 | 0.84 |
|  | VIT_219s0014g01780 | 3.03 | 7.80 | 1.34 | 39.81 | 30.93 | 26.79 | 27.12 | 19.18 | 18.81 | 31.62 | 15.45 | 2.93 | 2.66 | 2.98 |
| PR-1 | VIT_200s0207g00130 | 4.14 | 3.91 | 4.34 | 0.10 | 1.12 | 0.19 | 0.93 | 0.48 | 0.00 | 0.09 | 0.29 | 0.09 | 0.10 | 0.00 |
|  | VIT_200s0207g00160 | 0.37 | 0.64 | 0.67 | 0.00 | 0.12 | 0.00 | 0.13 | 0.00 | 0.00 | 0.13 | 0.00 | 0.00 | 0.00 | 0.00 |
|  | VIT_203s0088g00690 | 3.82 | 0.58 | 0.87 | 0.23 | 0.22 | 0.00 | 1.69 | 5.17 | 0.61 | 1.50 | 0.32 | 0.74 | 0.65 | 1.83 |
|  | VIT_203s0088g00700 | 6.95 | 0.33 | 0.44 | 0.23 | 0.00 | 0.00 | 0.00 | 1.43 | 0.58 | 0.53 | 0.44 | 0.74 | 0.77 | 0.62 |
|  | VIT_203s0088g00710 | 16.84 | 1.09 | 1.49 | 4.87 | 0.00 | 0.18 | 0.31 | 2.19 | 33.22 | 1.36 | 1.54 | 11.06 | 4.00 | 5.32 |
|  | VIT_203s0088g00720 | 0.71 | 0.00 | 0.00 | 0.00 | 0.00 | 0.00 | 0.00 | 0.00 | 0.00 | 0.00 | 0.00 | 0.00 | 0.00 | 0.00 |
|  | VIT_203s0088g00750 | 0.10 | 0.10 | 0.00 | 0.00 | 0.00 | 0.00 | 0.00 | 0.00 | 0.19 | 0.00 | 0.00 | 0.00 | 0.11 | 0.00 |
|  | VIT_203s0088g00780 | 0.45 | 0.24 | 0.73 | 0.00 | 0.00 | 0.00 | 0.09 | 1.64 | 0.09 | 0.00 | 0.19 | 0.09 | 0.09 | 0.76 |
|  | VIT_203s0088g00810 | 82.18 | 130.94 | 143.68 | 23.59 | 72.33 | 6.05 | 12.72 | 116.38 | 9.37 | 9.61 | 23.54 | 1.87 | 2.83 | 16.94 |
|  | VIT_203s0088g00890 | 0.00 | 0.27 | 0.00 | 0.00 | 0.00 | 0.00 | 0.00 | 0.00 | 0.00 | 0.00 | 0.00 | 0.00 | 0.00 | 0.00 |
|  | VIT_203s0088g00900 | 1.06 | 0.40 | 0.68 | 0.13 | 0.51 | 0.53 | 0.13 | 0.54 | 0.53 | 0.78 | 1.08 | 1.55 | 1.09 | 1.38 |
|  | VIT_203s0088g00910 | 3.39 | 1.59 | 2.76 | 0.37 | 0.16 | 0.42 | 0.37 | 11.27 | 0.79 | 0.43 | 0.97 | 0.06 | 0.13 | 2.37 |
|  | VIT_203s0088g00940 | 0.41 | 0.00 | 0.00 | 0.00 | 0.00 | 0.00 | 0.00 | 0.00 | 0.00 | 0.00 | 0.00 | 0.00 | 0.00 | 0.00 |
|  | VIT_203s0088g01085 | 0.25 | 0.00 | 0.00 | 0.00 | 0.00 | 0.00 | 0.00 | 0.00 | 0.00 | 0.00 | 0.00 | 0.00 | 0.00 | 0.00 |
|  | VIT_203s0097g00700 | 1.53 | 0.10 | 1.06 | 0.10 | 0.29 | 0.00 | 0.29 | 4.48 | 0.09 | 0.19 | 0.10 | 0.28 | 0.29 | 1.87 |
|  | VIT_208s0040g02890 | 0.08 | 0.09 | 0.17 | 0.00 | 0.08 | 0.00 | 0.00 | 0.09 | 0.16 | 0.08 | 0.08 | 0.00 | 0.08 | 0.09 |
|  | VIT_211s0052g01620 | 0.64 | 0.00 | 0.07 | 0.08 | 0.00 | 0.00 | 0.07 | 0.14 | 0.14 | 0.00 | 0.00 | 0.00 | 0.00 | 0.00 |
|  | VIT_211s0052g01650 | 126.51 | 0.25 | 0.77 | 0.64 | 0.05 | 0.00 | 0.00 | 1.38 | 0.00 | 0.00 | 0.10 | 0.00 | 0.00 | 0.11 |

T1: cluster bagging from 3 WAF until harvest; T2: control group; T8: cluster bagging at E-L 35 stage and bag removal at E-L 36 stage. Red and green boxes represent genes that are significantly up and down-regulated after cluster bagging treatment, respectively.
