# Supplementary material for: Comparative physiological, metabolomic, and transcriptomic analyses reveal developmental stage-dependent effects of cluster bagging on phenolic metabolism in Cabernet Sauvignon grape berries
Source: BMC Plant Biol. 2019 Dec 26;19:583. doi: 10.1186/s12870-019-2186-z (PMC6933938; doi:10.1186/s12870-019-2186-z)
Supplement: Supplementary file 13 — Additional file 13: Table S8. Co-expression analysis of genes encoding transcription factors and key enzymes required for sunlight-regulated phenolic biosynthesis. [file 12870_2019_2186_MOESM13_ESM.docx]

**Table S8.** Co-expression analysis of genes encoding transcription factors and key enzymes required for sunlight-regulated phenolic biosynthesis.

| Key Enzymes | | Pearson Correlation Coefficient | Transcription factors | | |
| --- | --- | --- | --- | --- | --- |
| Gene ID | Family |  | Gene ID | Family | Blast nr |
| VIT_216s0039g01100 | PAL | 0.934 | VIT_211s0016g02070 | bHLH | gi\|225444893\|ref\|XP_002279486.1\|/0/PREDICTED: putative transcription factor bHLH041 [Vitis vinifera]gi\|297738655\|emb\|CBI27900.3\| unnamed protein product [*Vitis vinifera*] |
| VIT_216s0039g01100 | PAL | 0.919 | VIT_219s0014g04940 | GRAS | gi\|302143189\|emb\|CBI20484.3\|/0/unnamed protein product [*Vitis vinifera*] |
| VIT_216s0039g01100 | PAL | 0.907 | VIT_208s0007g03900 | HSF | gi\|297740065\|emb\|CBI30247.3\|/0/unnamed protein product [*Vitis vinifera*] |
| VIT_216s0039g01100 | PAL | 0.915 | VIT_206s0004g00020 | NAC | gi\|225463127\|ref\|XP_002265611.1\|/0/PREDICTED: uncharacterized protein LOC100249427 [*Vitis vinifera*]gi\|297739348\|emb\|CBI29338.3\| unnamed protein product [*Vitis vinifera*] |
| VIT_216s0039g01100 | PAL | 0.926 | VIT_216s0050g02510 | WRKY | gi\|359490533\|ref\|XP_002267793.2\|/0/PREDICTED: probable WRKY transcription factor 53-like [*Vitis vinifera*]gi\|302143686\|emb\|CBI22547.3\| unnamed protein product [*Vitis vinifera*] |
| VIT_216s0039g01100 | PAL | 0.904 | VIT_201s0011g00720 | WRKY | gi\|526117465\|ref\|NP_001268029.1\|/6.50371e-92/uncharacterized protein LOC100263794 [*Vitis vinifera*]gi\|321496082\|gb\|ADW93918.1\| copper transporter [*Vitis vinifera*] |
| VIT_210s0042g00640 | CCR | 0.926 | VIT_201s0010g02100 | C2H2 | gi\|302142947\|emb\|CBI20242.3\|/0/unnamed protein product [*Vitis vinifera*] |
| VIT_218s0122g00620 | CCR | 0.912 | VIT_207s0031g00780 | B3 | gi\|296082432\|emb\|CBI21437.3\|/0/unnamed protein product [*Vitis vinifera*] |
| VIT_218s0122g00620 | CCR | 0.906 | VIT_207s0005g05400 | B3 | gi\|297744288\|emb\|CBI37258.3\|/0/unnamed protein product [*Vitis vinifera*] |
| VIT_218s0122g00620 | CCR | 0.904 | VIT_207s0005g05100 | bHLH | gi\|297744279\|emb\|CBI37249.3\|/0/unnamed protein product [*Vitis vinifera*] |
| VIT_218s0122g00620 | CCR | 0.939 | VIT_208s0007g07870 | bHLH | gi\|297735141\|emb\|CBI17503.3\|/0/unnamed protein product [*Vitis vinifera*] |
| VIT_218s0122g00620 | CCR | 0.945 | VIT_201s0010g02100 | C2H2 | gi\|302142947\|emb\|CBI20242.3\|/0/unnamed protein product [*Vitis vinifera*] |
| VIT_218s0122g00620 | CCR | 0.900 | VIT_217s0000g04990 | MIKC_MADS | gi\|225456542\|ref\|XP_002263017.1\|/7.10011e-154/PREDICTED: agamous-like MADS-box protein AGL8 homolog [*Vitis vinifera*]gi\|297734092\|emb\|CBI15339.3\| unnamed protein product [*Vitis vinifera*] |
| VIT_200s0615g00020 | CAD | 0.911 | VIT_206s0004g03130 | ARF | gi\|359479063\|ref\|XP_002285019.2\|/0/PREDICTED: auxin response factor 4-like [*Vitis vinifera*]gi\|297746231\|emb\|CBI16287.3\| unnamed protein product [*Vitis vinifera*] |
| VIT_200s0615g00020 | CAD | 0.924 | VIT_201s0011g03070 | B3 | gi\|297742476\|emb\|CBI34625.3\|/0/unnamed protein product [*Vitis vinifera*] |
| VIT_200s0615g00020 | CAD | 0.951 | VIT_215s0048g02820 | bHLH | gi\|296088987\|emb\|CBI38690.3\|/0/unnamed protein product [*Vitis vinifera*] |
| VIT_200s0615g00020 | CAD | 0.909 | VIT_200s1314g00010 | bHLH | gi\|297735854\|emb\|CBI18608.3\|/0/unnamed protein product [*Vitis vinifera*] |
| VIT_200s0615g00020 | CAD | 0.956 | VIT_218s0001g08040 | bHLH | gi\|302142109\|emb\|CBI19312.3\|/0/unnamed protein product [*Vitis vinifera*] |
| VIT_200s0615g00020 | CAD | 0.910 | VIT_212s0028g03550 | bHLH | gi\|302143302\|emb\|CBI21863.3\|/1.13095e-134/unnamed protein product [*Vitis vinifera*] |
| VIT_200s0615g00020 | CAD | 0.944 | VIT_205s0049g00460 | bHLH | gi\|296083619\|emb\|CBI23608.3\|/7.3294e-164/unnamed protein product [*Vitis vinifera*] |
| VIT_200s0615g00020 | CAD | 0.913 | VIT_211s0052g00100 | bHLH | gi\|225445937\|ref\|XP_002263999.1\|/1.19018e-158/PREDICTED: transcription factor bHLH35 [*Vitis vinifera*]gi\|297735470\|emb\|CBI17910.3\| unnamed protein product [*Vitis vinifera*] |
| VIT_200s0615g00020 | CAD | 0.944 | VIT_218s0001g12220 | C2H2 | gi\|225458335\|ref\|XP_002281605.1\|/0/PREDICTED: uncharacterized protein LOC100260826 [*Vitis vinifera*] |
| VIT_200s0615g00020 | CAD | 0.901 | VIT_216s0013g00900 | ERF | gi\|359490063\|ref\|XP_003634024.1\|/2.83781e-180/PREDICTED: ethylene-responsive transcription factor 5-like [*Vitis vinifera*]gi\|297745013\|emb\|CBI38605.3\| unnamed protein product [*Vitis vinifera*] |
| VIT_200s0615g00020 | CAD | 0.915 | VIT_218s0001g05850 | ERF | gi\|225460135\|ref\|XP_002275853.1\|/4.47036e-117/PREDICTED: ethylene-responsive transcription factor ERF022 [*Vitis vinifera*]gi\|297741012\|emb\|CBI31324.3\| unnamed protein product [*Vitis vinifera*] |
| VIT_200s0615g00020 | CAD | 0.931 | VIT_210s0003g00580 | ERF | gi\|297740455\|emb\|CBI30637.3\|/1.01677e-111/unnamed protein product [*Vitis vinifera*] |
| VIT_200s0615g00020 | CAD | 0.944 | VIT_207s0031g01980 | ERF | gi\|225438481\|ref\|XP_002278226.1\|/0/PREDICTED: ethylene-responsive transcription factor ABR1 [*Vitis vinifera*]gi\|296082543\|emb\|CBI21548.3\| unnamed protein product [*Vitis vinifera*] |
| VIT_200s0615g00020 | CAD | 0.950 | VIT_218s0072g00260 | ERF | gi\|297739642\|emb\|CBI29824.3\|/0/unnamed protein product [*Vitis vinifera*] |
| VIT_200s0615g00020 | CAD | 0.932 | VIT_219s0014g02240 | ERF | gi\|225461524\|ref\|XP_002285146.1\|/2.98205e-111/PREDICTED: ethylene-responsive transcription factor 4 [*Vitis vinifera*]gi\|37625037\|gb\|AAQ96342.1\| putative ethylene response factor ERF3b [Vitis aestivalis]gi\|302142968\|emb\|CBI20263.3\| unnamed protein product [*Vitis vinifera*] |
| VIT_200s0615g00020 | CAD | 0.902 | VIT_208s0007g07550 | GATA | gi\|297741373\|emb\|CBI32504.3\|/0/unnamed protein product [*Vitis vinifera*] |
| VIT_200s0615g00020 | CAD | 0.902 | VIT_206s0004g04980 | GRAS | gi\|297746053\|emb\|CBI16109.3\|/0/unnamed protein product [*Vitis vinifera*] |
| VIT_200s0615g00020 | CAD | 0.906 | VIT_219s0014g04940 | GRAS | gi\|302143189\|emb\|CBI20484.3\|/0/unnamed protein product [*Vitis vinifera*] |
| VIT_200s0615g00020 | CAD | 0.911 | VIT_216s0100g00670 | HD-ZIP | gi\|297741089\|emb\|CBI31820.3\|/0/unnamed protein product [*Vitis vinifera*] |
| VIT_200s0615g00020 | CAD | 0.928 | VIT_217s0000g09080 | MYB | gi\|297733722\|emb\|CBI14969.3\|/1.79323e-134/unnamed protein product [*Vitis vinifera*] |
| VIT_200s0615g00020 | CAD | 0.949 | VIT_207s0005g03340 | MYB | gi\|297745038\|emb\|CBI38630.3\|/1.51284e-81/unnamed protein product [*Vitis vinifera*] |
| VIT_200s0615g00020 | CAD | 0.930 | VIT_207s0031g02610 | NAC | gi\|296082607\|emb\|CBI21612.3\|/0/unnamed protein product [*Vitis vinifera*] |
| VIT_200s0615g00020 | CAD | 0.920 | VIT_209s0002g01590 | NF-YA | gi\|225442180\|ref\|XP_002274458.1\|/0/PREDICTED: nuclear transcription factor Y subunit A-3 [*Vitis vinifera*]gi\|297743031\|emb\|CBI35898.3\| unnamed protein product [*Vitis vinifera*] |
| VIT_200s0615g00020 | CAD | 0.915 | VIT_204s0008g06130 | TALE | gi\|359475858\|ref\|XP_002285407.2\|/0/PREDICTED: homeobox protein knotted-1-like 3-like [*Vitis vinifera*] |
| VIT_200s0615g00020 | CAD | 0.929 | VIT_219s0090g00840 | WRKY | gi\|147860185\|emb\|CAN78720.1\|/0/hypothetical protein VITISV_035804 [*Vitis vinifera*] |
| VIT_200s0615g00020 | CAD | 0.912 | VIT_210s0003g02810 | WRKY | gi\|225463412\|ref\|XP_002272089.1\|/0/PREDICTED: probable WRKY transcription factor 28 [*Vitis vinifera*]gi\|297740645\|emb\|CBI30827.3\| unnamed protein product [*Vitis vinifera*] |
| VIT_200s0615g00020 | CAD | 0.920 | VIT_215s0046g02190 | WRKY | gi\|225454298\|ref\|XP_002276925.1\|/0/PREDICTED: WRKY transcription factor 22 [*Vitis vinifera*]gi\|297745327\|emb\|CBI40407.3\| unnamed protein product [*Vitis vinifera*] |
| VIT_200s0615g00020 | CAD | 0.921 | VIT_210s0116g01200 | WRKY | gi\|302144104\|emb\|CBI23209.3\|/0/unnamed protein product [*Vitis vinifera*] |
| VIT_200s0615g00020 | CAD | 0.910 | VIT_202s0154g00070 | YABBY | gi\|225426944\|ref\|XP_002266233.1\|/6.75169e-131/PREDICTED: axial regulator YABBY 1 [*Vitis vinifera*]gi\|297741152\|emb\|CBI31883.3\| unnamed protein product [*Vitis vinifera*] |
| VIT_200s0371g00100 | CAD | 0.950 | VIT_218s0001g08610 | AP2 | gi\|302142158\|emb\|CBI19361.3\|/1.08131e-166/unnamed protein product [*Vitis vinifera*] |
| VIT_200s0371g00100 | CAD | 0.902 | VIT_206s0004g03590 | AP2 | gi\|297746184\|emb\|CBI16240.3\|/0/unnamed protein product [*Vitis vinifera*] |
| VIT_200s0371g00100 | CAD | 0.928 | VIT_208s0007g08580 | AP2 | gi\|302142158\|emb\|CBI19361.3\|/1.08131e-166/unnamed protein product [*Vitis vinifera*] |
| VIT_200s0371g00100 | CAD | 0.959 | VIT_215s0046g00290 | ARF | gi\|297745485\|emb\|CBI40565.3\|/0/unnamed protein product [*Vitis vinifera*] |
| VIT_200s0371g00100 | CAD | 0.932 | VIT_202s0025g01740 | ARF | gi\|297742361\|emb\|CBI34510.3\|/0/unnamed protein product [*Vitis vinifera*] |
| VIT_200s0371g00100 | CAD | 0.979 | VIT_206s0004g03130 | ARF | gi\|359479063\|ref\|XP_002285019.2\|/0/PREDICTED: auxin response factor 4-like [*Vitis vinifera*]gi\|297746231\|emb\|CBI16287.3\| unnamed protein product [*Vitis vinifera*] |
| VIT_200s0371g00100 | CAD | 0.931 | VIT_218s0089g00910 | ARF | gi\|296090355\|emb\|CBI40174.3\|/1.1514e-37/unnamed protein product [*Vitis vinifera*] |
| VIT_200s0371g00100 | CAD | 0.965 | VIT_201s0011g03070 | B3 | gi\|297742476\|emb\|CBI34625.3\|/0/unnamed protein product [*Vitis vinifera*] |
| VIT_200s0371g00100 | CAD | 0.912 | VIT_201s0011g02940 | bHLH | gi\|297745500\|emb\|CBI40580.3\|/0/unnamed protein product [*Vitis vinifera*] |
| VIT_200s0371g00100 | CAD | 0.961 | VIT_200s1314g00010 | bHLH | gi\|297735854\|emb\|CBI18608.3\|/0/unnamed protein product [*Vitis vinifera*] |
| VIT_200s0371g00100 | CAD | 0.914 | VIT_213s0064g01290 | bHLH | gi\|297736219\|emb\|CBI24857.3\|/2.57926e-176/unnamed protein product [*Vitis vinifera*] |
| VIT_200s0371g00100 | CAD | 0.937 | VIT_205s0124g00240 | bHLH | gi\|297745167\|emb\|CBI39159.3\|/0/unnamed protein product [*Vitis vinifera*] |
| VIT_200s0371g00100 | CAD | 0.933 | VIT_212s0028g03550 | bHLH | gi\|302143302\|emb\|CBI21863.3\|/1.13095e-134/unnamed protein product [*Vitis vinifera*] |
| VIT_200s0371g00100 | CAD | 0.911 | VIT_205s0049g00460 | bHLH | gi\|296083619\|emb\|CBI23608.3\|/7.3294e-164/unnamed protein product [*Vitis vinifera*] |
| VIT_200s0371g00100 | CAD | 0.932 | VIT_205s0020g04780 | bHLH | gi\|302142947\|emb\|CBI20242.3\|/0/unnamed protein product [*Vitis vinifera*] |
| VIT_200s0371g00100 | CAD | 0.971 | VIT_211s0052g00100 | bHLH | gi\|225445937\|ref\|XP_002263999.1\|/1.19018e-158/PREDICTED: transcription factor bHLH35 [*Vitis vinifera*]gi\|297735470\|emb\|CBI17910.3\| unnamed protein product [*Vitis vinifera*] |
| VIT_200s0371g00100 | CAD | 0.927 | VIT_218s0001g04470 | bZIP | gi\|302143953\|emb\|CBI23058.3\|/0/unnamed protein product [*Vitis vinifera*] |
| VIT_200s0371g00100 | CAD | 0.942 | VIT_207s0031g01320 | bZIP | gi\|225438607\|ref\|XP_002280782.1\|/0/PREDICTED: transcription factor TGA1 [*Vitis vinifera*] |
| VIT_200s0371g00100 | CAD | 0.968 | VIT_219s0014g05000 | C2H2 | gi\|302143195\|emb\|CBI20490.3\|/1.65922e-55/unnamed protein product [*Vitis vinifera*] |
| VIT_200s0371g00100 | CAD | 0.969 | VIT_218s0001g14130 | C2H2 | gi\|302142648\|emb\|CBI19851.3\|/0/unnamed protein product [*Vitis vinifera*] |
| VIT_200s0371g00100 | CAD | 0.950 | VIT_206s0004g03070 | C2H2 | gi\|297746237\|emb\|CBI16293.3\|/0/unnamed protein product [*Vitis vinifera*] |
| VIT_200s0371g00100 | CAD | 0.905 | VIT_218s0001g12220 | C2H2 | gi\|225458335\|ref\|XP_002281605.1\|/0/PREDICTED: uncharacterized protein LOC100260826 [*Vitis vinifera*] |
| VIT_200s0371g00100 | CAD | 0.905 | VIT_207s0141g00020 | C2H2 | gi\|297742862\|emb\|CBI35627.3\|/0/unnamed protein product [*Vitis vinifera*] |
| VIT_200s0371g00100 | CAD | 0.928 | VIT_208s0007g06400 | C3H | gi\|297739844\|emb\|CBI30026.3\|/3.76669e-156/unnamed protein product [*Vitis vinifera*] |
| VIT_200s0371g00100 | CAD | 0.956 | VIT_205s0077g01240 | CAMTA | gi\|359477127\|ref\|XP_002272118.2\|/0/PREDICTED: calmodulin-binding transcription activator 5-like [*Vitis vinifera*]gi\|296083270\|emb\|CBI22906.3\| unnamed protein product [*Vitis vinifera*] |
| VIT_200s0371g00100 | CAD | 0.902 | VIT_207s0141g00250 | CAMTA | gi\|297742873\|emb\|CBI35638.3\|/0/unnamed protein product [*Vitis vinifera*] |
| VIT_200s0371g00100 | CAD | 0.949 | VIT_204s0008g07340 | CO-like | gi\|225430571\|ref\|XP_002263458.1\|/0/PREDICTED: zinc finger protein CONSTANS-LIKE 4-like [*Vitis vinifera*] |
| VIT_200s0371g00100 | CAD | 0.921 | VIT_205s0020g03880 | CPP | gi\|297737091\|emb\|CBI26292.3\|/0/unnamed protein product [*Vitis vinifera*] |
| VIT_200s0371g00100 | CAD | 0.901 | VIT_204s0023g03030 | DBB | gi\|359476640\|ref\|XP_003631872.1\|/3.60414e-134/PREDICTED: probable salt tolerance-like protein At1g78600-like [*Vitis vinifera*]gi\|297735043\|emb\|CBI17405.3\| unnamed protein product [*Vitis vinifera*] |
| VIT_200s0371g00100 | CAD | 0.929 | VIT_206s0009g01380 | EIL | gi\|296083982\|emb\|CBI24370.3\|/0/unnamed protein product [*Vitis vinifera*] |
| VIT_200s0371g00100 | CAD | 0.945 | VIT_211s0037g00010 | ERF | gi\|297743629\|emb\|CBI36512.3\|/4.00566e-177/unnamed protein product [*Vitis vinifera*] |
| VIT_200s0371g00100 | CAD | 0.942 | VIT_218s0001g05850 | ERF | gi\|225460135\|ref\|XP_002275853.1\|/4.47036e-117/PREDICTED: ethylene-responsive transcription factor ERF022 [*Vitis vinifera*]gi\|297741012\|emb\|CBI31324.3\| unnamed protein product [*Vitis vinifera*] |
| VIT_200s0371g00100 | CAD | 0.958 | VIT_210s0003g00580 | ERF | gi\|297740455\|emb\|CBI30637.3\|/1.01677e-111/unnamed protein product [*Vitis vinifera*] |
| VIT_200s0371g00100 | CAD | 0.936 | VIT_207s0031g01980 | ERF | gi\|225438481\|ref\|XP_002278226.1\|/0/PREDICTED: ethylene-responsive transcription factor ABR1 [*Vitis vinifera*]gi\|296082543\|emb\|CBI21548.3\| unnamed protein product [*Vitis vinifera*] |
| VIT_200s0371g00100 | CAD | 0.928 | VIT_218s0072g00260 | ERF | gi\|297739642\|emb\|CBI29824.3\|/0/unnamed protein product [*Vitis vinifera*] |
| VIT_200s0371g00100 | CAD | 0.943 | VIT_219s0014g02240 | ERF | gi\|225461524\|ref\|XP_002285146.1\|/2.98205e-111/PREDICTED: ethylene-responsive transcription factor 4 [*Vitis vinifera*]gi\|37625037\|gb\|AAQ96342.1\| putative ethylene response factor ERF3b [Vitis aestivalis]gi\|302142968\|emb\|CBI20263.3\| unnamed protein product [*Vitis vinifera*] |
| VIT_200s0371g00100 | CAD | 0.916 | VIT_216s0013g01080 | ERF | gi\|297745018\|emb\|CBI38610.3\|/0/unnamed protein product [*Vitis vinifera*] |
| VIT_200s0371g00100 | CAD | 0.943 | VIT_212s0028g03100 | G2-like | gi\|302143340\|emb\|CBI21901.3\|/0/unnamed protein product [*Vitis vinifera*] |
| VIT_200s0371g00100 | CAD | 0.914 | VIT_202s0033g00300 | G2-like | gi\|359474709\|ref\|XP_002264257.2\|/0/PREDICTED: myb family transcription factor APL-like [*Vitis vinifera*]gi\|296088397\|emb\|CBI37388.3\| unnamed protein product [*Vitis vinifera*] |
| VIT_200s0371g00100 | CAD | 0.904 | VIT_207s0197g00060 | G2-like | gi\|359480439\|ref\|XP_002264629.2\|/0/PREDICTED: uncharacterized protein LOC100243049 [*Vitis vinifera*] |
| VIT_200s0371g00100 | CAD | 0.919 | VIT_208s0007g07550 | GATA | gi\|297741373\|emb\|CBI32504.3\|/0/unnamed protein product [*Vitis vinifera*] |
| VIT_200s0371g00100 | CAD | 0.944 | VIT_203s0038g00490 | GATA | gi\|297744743\|emb\|CBI38005.3\|/0/unnamed protein product [*Vitis vinifera*] |
| VIT_200s0371g00100 | CAD | 0.910 | VIT_206s0004g04980 | GRAS | gi\|297746053\|emb\|CBI16109.3\|/0/unnamed protein product [*Vitis vinifera*] |
| VIT_200s0371g00100 | CAD | 0.937 | VIT_219s0090g01740 | GRAS | gi\|297737537\|emb\|CBI26738.3\|/0/unnamed protein product [*Vitis vinifera*] |
| VIT_200s0371g00100 | CAD | 0.913 | VIT_204s0008g05190 | HB-other | gi\|296082012\|emb\|CBI21017.3\|/3.30142e-127/unnamed protein product [*Vitis vinifera*] |
| VIT_200s0371g00100 | CAD | 0.905 | VIT_216s0100g00670 | HD-ZIP | gi\|297741089\|emb\|CBI31820.3\|/0/unnamed protein product [*Vitis vinifera*] |
| VIT_200s0371g00100 | CAD | 0.926 | VIT_208s0007g06670 | HD-ZIP | gi\|225441481\|ref\|XP_002275747.1\|/7.83705e-137/PREDICTED: homeobox-leucine zipper protein HAT14-like [*Vitis vinifera*] |
| VIT_200s0371g00100 | CAD | 0.912 | VIT_215s0021g01880 | HD-ZIP | gi\|359481257\|ref\|XP_002265561.2\|/0/PREDICTED: uncharacterized protein LOC100251663 [*Vitis vinifera*] |
| VIT_200s0371g00100 | CAD | 0.913 | VIT_200s0732g00010 | HD-ZIP | gi\|359489471\|ref\|XP_002267504.2\|/2.84738e-162/PREDICTED: uncharacterized protein LOC100257521 [*Vitis vinifera*]gi\|296089004\|emb\|CBI38707.3\| unnamed protein product [*Vitis vinifera*] |
| VIT_200s0371g00100 | CAD | 0.943 | VIT_201s0026g01950 | HD-ZIP | gi\|297741835\|emb\|CBI33148.3\|/0/unnamed protein product [*Vitis vinifera*] |
| VIT_200s0371g00100 | CAD | 0.917 | VIT_207s0191g00180 | HD-ZIP | gi\|297741835\|emb\|CBI33148.3\|/0/unnamed protein product [*Vitis vinifera*] |
| VIT_200s0371g00100 | CAD | 0.944 | VIT_211s0016g03940 | HSF | gi\|296086987\|emb\|CBI33243.3\|/8.87649e-148/unnamed protein product [*Vitis vinifera*] |
| VIT_200s0371g00100 | CAD | 0.947 | VIT_208s0007g03900 | HSF | gi\|297740065\|emb\|CBI30247.3\|/0/unnamed protein product [*Vitis vinifera*] |
| VIT_200s0371g00100 | CAD | 0.933 | VIT_201s0011g05970 | HSF | gi\|359472583\|ref\|XP_003631170.1\|/0/PREDICTED: LOW QUALITY PROTEIN: heat stress transcription factor A-8-like [*Vitis vinifera*] |
| VIT_200s0371g00100 | CAD | 0.907 | VIT_215s0046g00230 | LBD | gi\|297745491\|emb\|CBI40571.3\|/1.61778e-98/unnamed protein product [*Vitis vinifera*] |
| VIT_200s0371g00100 | CAD | 0.967 | VIT_202s0012g00990 | LSD | gi\|297742122\|emb\|CBI33909.3\|/7.87443e-116/unnamed protein product [*Vitis vinifera*] |
| VIT_200s0371g00100 | CAD | 0.961 | VIT_214s0083g01030 | MIKC_MADS | gi\|298204454\|emb\|CBI16934.3\|/4.57831e-155/unnamed protein product [*Vitis vinifera*] |
| VIT_200s0371g00100 | CAD | 0.945 | VIT_210s0003g02070 | MIKC_MADS | gi\|359485775\|ref\|XP_002267619.2\|/6.32058e-52/PREDICTED: uncharacterized protein LOC100254625 [*Vitis vinifera*]gi\|296085047\|emb\|CBI28462.3\| unnamed protein product [*Vitis vinifera*] |
| VIT_200s0371g00100 | CAD | 0.954 | VIT_217s0000g09080 | MYB | gi\|297733722\|emb\|CBI14969.3\|/1.79323e-134/unnamed protein product [*Vitis vinifera*] |
| VIT_200s0371g00100 | CAD | 0.917 | VIT_206s0080g00790 | MYB | gi\|296084883\|emb\|CBI28292.3\|/3.08946e-156/unnamed protein product [*Vitis vinifera*] |
| VIT_200s0371g00100 | CAD | 0.931 | VIT_216s0050g02530 | MYB_related | gi\|225462727\|ref\|XP_002268795.1\|/8.92238e-58/PREDICTED: transcription factor TRY [*Vitis vinifera*]gi\|158323782\|gb\|ABW34395.1\| Myb-related TRIPTYCHON-like protein [*Vitis vinifera*]gi\|302143684\|emb\|CBI22545.3\| unnamed protein product [*Vitis vinifera*] |
| VIT_200s0371g00100 | CAD | 0.915 | VIT_209s0002g03610 | MYB_related | gi\|225442479\|ref\|XP_002283785.1\|/0/PREDICTED: uncharacterized protein LOC100241227 [*Vitis vinifera*]gi\|297743200\|emb\|CBI36067.3\| unnamed protein product [*Vitis vinifera*] |
| VIT_200s0371g00100 | CAD | 0.968 | VIT_215s0048g02280 | NAC | gi\|296089035\|emb\|CBI38738.3\|/0/unnamed protein product [*Vitis vinifera*] |
| VIT_200s0371g00100 | CAD | 0.953 | VIT_206s0080g00780 | NAC | gi\|296089502\|emb\|CBI39321.3\|/6.43092e-170/unnamed protein product [*Vitis vinifera*] |
| VIT_200s0371g00100 | CAD | 0.946 | VIT_207s0031g02610 | NAC | gi\|296082607\|emb\|CBI21612.3\|/0/unnamed protein product [*Vitis vinifera*] |
| VIT_200s0371g00100 | CAD | 0.937 | VIT_218s0001g11980 | NAC | gi\|225458374\|ref\|XP_002283395.1\|/0/PREDICTED: uncharacterized protein LOC100252228 [*Vitis vinifera*] |
| VIT_200s0371g00100 | CAD | 0.911 | VIT_201s0026g02710 | NAC | gi\|296086346\|emb\|CBI31935.3\|/0/unnamed protein product [*Vitis vinifera*] |
| VIT_200s0371g00100 | CAD | 0.941 | VIT_209s0002g01590 | NF-YA | gi\|225442180\|ref\|XP_002274458.1\|/0/PREDICTED: nuclear transcription factor Y subunit A-3 [*Vitis vinifera*]gi\|297743031\|emb\|CBI35898.3\| unnamed protein product [*Vitis vinifera*] |
| VIT_200s0371g00100 | CAD | 0.911 | VIT_208s0007g08250 | NF-YA | gi\|297739684\|emb\|CBI29866.3\|/0/unnamed protein product [*Vitis vinifera*] |
| VIT_200s0371g00100 | CAD | 0.934 | VIT_212s0028g03350 | SBP | gi\|225456345\|ref\|XP_002280160.1\|/0/PREDICTED: squamosa promoter-binding-like protein 13 [*Vitis vinifera*]gi\|147837620\|emb\|CAN77059.1\| hypothetical protein VITISV_022138 [*Vitis vinifera*]gi\|297734432\|emb\|CBI15679.3\| unnamed protein product [*Vitis vinifera*] |
| VIT_200s0371g00100 | CAD | 0.926 | VIT_201s0010g03710 | SBP | gi\|297738463\|emb\|CBI27664.3\|/0/unnamed protein product [*Vitis vinifera*] |
| VIT_200s0371g00100 | CAD | 0.906 | VIT_204s0008g06130 | TALE | gi\|359475858\|ref\|XP_002285407.2\|/0/PREDICTED: homeobox protein knotted-1-like 3-like [*Vitis vinifera*] |
| VIT_200s0371g00100 | CAD | 0.945 | VIT_206s0009g00410 | TALE | gi\|296084040\|emb\|CBI24428.3\|/0/unnamed protein product [*Vitis vinifera*] |
| VIT_200s0371g00100 | CAD | 0.917 | VIT_217s0000g06020 | TCP | gi\|297733992\|emb\|CBI15239.3\|/0/unnamed protein product [*Vitis vinifera*] |
| VIT_200s0371g00100 | CAD | 0.915 | VIT_212s0035g00690 | TCP | gi\|359482656\|ref\|XP_002264851.2\|/0/PREDICTED: uncharacterized protein LOC100248501 [*Vitis vinifera*]gi\|297743015\|emb\|CBI35882.3\| unnamed protein product [*Vitis vinifera*] |
| VIT_200s0371g00100 | CAD | 0.939 | VIT_210s0003g02810 | WRKY | gi\|225463412\|ref\|XP_002272089.1\|/0/PREDICTED: probable WRKY transcription factor 28 [*Vitis vinifera*]gi\|297740645\|emb\|CBI30827.3\| unnamed protein product [*Vitis vinifera*] |
| VIT_200s0371g00100 | CAD | 0.923 | VIT_204s0069g00920 | WRKY | gi\|296083797\|emb\|CBI24014.3\|/2.44175e-175/unnamed protein product [*Vitis vinifera*] |
| VIT_200s0371g00100 | CAD | 0.952 | VIT_207s0031g00080 | WRKY | gi\|296082371\|emb\|CBI21376.3\|/8.1921e-161/unnamed protein product [*Vitis vinifera*] |
| VIT_200s0371g00100 | CAD | 0.924 | VIT_210s0003g01600 | WRKY | gi\|225443744\|ref\|XP_002269267.1\|/1.49478e-160/PREDICTED: probable WRKY transcription factor 65 [*Vitis vinifera*]gi\|297740534\|emb\|CBI30716.3\| unnamed protein product [*Vitis vinifera*] |
| VIT_200s0371g00100 | CAD | 0.952 | VIT_215s0046g02190 | WRKY | gi\|225454298\|ref\|XP_002276925.1\|/0/PREDICTED: WRKY transcription factor 22 [*Vitis vinifera*]gi\|297745327\|emb\|CBI40407.3\| unnamed protein product [*Vitis vinifera*] |
| VIT_200s0371g00100 | CAD | 0.911 | VIT_207s0141g00680 | WRKY | gi\|296087892\|emb\|CBI35175.3\|/0/unnamed protein product [*Vitis vinifera*] |
| VIT_200s0371g00100 | CAD | 0.908 | VIT_210s0116g01200 | WRKY | gi\|302144104\|emb\|CBI23209.3\|/0/unnamed protein product [*Vitis vinifera*] |
| VIT_200s0371g00100 | CAD | 0.948 | VIT_209s0018g00240 | WRKY | gi\|298204668\|emb\|CBI25166.3\|/1.04765e-177/unnamed protein product [*Vitis vinifera*] |
| VIT_200s0371g00100 | CAD | 0.941 | VIT_202s0154g00070 | YABBY | gi\|225426944\|ref\|XP_002266233.1\|/6.75169e-131/PREDICTED: axial regulator YABBY 1 [*Vitis vinifera*]gi\|297741152\|emb\|CBI31883.3\| unnamed protein product [*Vitis vinifera*] |
| VIT_200s0371g00100 | CAD | 0.903 | VIT_208s0032g01110 | YABBY | gi\|526118055\|ref\|NP_001268184.1\|/2.9443e-153/calcineurin B-like protein 08 [*Vitis vinifera*]gi\|229609893\|gb\|ACQ83562.1\| calcineurin B-like protein 08 [*Vitis vinifera*]gi\|297742351\|emb\|CBI34500.3\| unnamed protein product [*Vitis vinifera*] |
| VIT_204s0044g00210 | CAD | 0.910 | VIT_208s0007g06160 | bZIP | gi\|297739863\|emb\|CBI30045.3\|/0/unnamed protein product [*Vitis vinifera*] |
| VIT_204s0044g00210 | CAD | 0.951 | VIT_211s0016g02010 | HSF | gi\|297738649\|emb\|CBI27894.3\|/0/unnamed protein product [*Vitis vinifera*] |
| VIT_204s0044g00210 | CAD | 0.901 | VIT_218s0001g09540 | MIKC_MADS | gi\|225458762\|ref\|XP_002285097.1\|/0/PREDICTED: MADS-box transcription factor 3 [*Vitis vinifera*]gi\|302142239\|emb\|CBI19442.3\| unnamed protein product [*Vitis vinifera*] |
| VIT_202s0012g00400 | FLS | 0.908 | VIT_218s0001g08610 | AP2 | gi\|302142158\|emb\|CBI19361.3\|/1.08131e-166/unnamed protein product [*Vitis vinifera*] |
| VIT_202s0012g00400 | FLS | 0.913 | VIT_206s0004g03130 | ARF | gi\|359479063\|ref\|XP_002285019.2\|/0/PREDICTED: auxin response factor 4-like [*Vitis vinifera*]gi\|297746231\|emb\|CBI16287.3\| unnamed protein product [*Vitis vinifera*] |
| VIT_202s0012g00400 | FLS | 0.932 | VIT_201s0011g03070 | B3 | gi\|297742476\|emb\|CBI34625.3\|/0/unnamed protein product [*Vitis vinifera*] |
| VIT_202s0012g00400 | FLS | 0.932 | VIT_200s0824g00020 | bHLH | gi\|296083537\|emb\|CBI14785.3\|/2.78689e-139/unnamed protein product [*Vitis vinifera*] |
| VIT_202s0012g00400 | FLS | 0.912 | VIT_200s1314g00010 | bHLH | gi\|297735854\|emb\|CBI18608.3\|/0/unnamed protein product [*Vitis vinifera*] |
| VIT_202s0012g00400 | FLS | 0.923 | VIT_205s0124g00240 | bHLH | gi\|297745167\|emb\|CBI39159.3\|/0/unnamed protein product [*Vitis vinifera*] |
| VIT_202s0012g00400 | FLS | 0.948 | VIT_212s0028g03550 | bHLH | gi\|302143302\|emb\|CBI21863.3\|/1.13095e-134/unnamed protein product [*Vitis vinifera*] |
| VIT_202s0012g00400 | FLS | 0.911 | VIT_205s0049g00460 | bHLH | gi\|296083619\|emb\|CBI23608.3\|/7.3294e-164/unnamed protein product [*Vitis vinifera*] |
| VIT_202s0012g00400 | FLS | 0.961 | VIT_205s0020g04780 | bHLH | gi\|302142947\|emb\|CBI20242.3\|/0/unnamed protein product [*Vitis vinifera*] |
| VIT_202s0012g00400 | FLS | 0.934 | VIT_211s0052g00100 | bHLH | gi\|225445937\|ref\|XP_002263999.1\|/1.19018e-158/PREDICTED: transcription factor bHLH35 [*Vitis vinifera*]gi\|297735470\|emb\|CBI17910.3\| unnamed protein product [*Vitis vinifera*] |
| VIT_202s0012g00400 | FLS | 0.939 | VIT_218s0001g04470 | bZIP | gi\|302143953\|emb\|CBI23058.3\|/0/unnamed protein product [*Vitis vinifera*] |
| VIT_202s0012g00400 | FLS | 0.907 | VIT_207s0031g01320 | bZIP | gi\|225438607\|ref\|XP_002280782.1\|/0/PREDICTED: transcription factor TGA1 [*Vitis vinifera*] |
| VIT_202s0012g00400 | FLS | 0.920 | VIT_219s0014g05000 | C2H2 | gi\|302143195\|emb\|CBI20490.3\|/1.65922e-55/unnamed protein product [*Vitis vinifera*] |
| VIT_202s0012g00400 | FLS | 0.918 | VIT_218s0001g14130 | C2H2 | gi\|302142648\|emb\|CBI19851.3\|/0/unnamed protein product [*Vitis vinifera*] |
| VIT_202s0012g00400 | FLS | 0.940 | VIT_204s0008g07340 | CO-like | gi\|225430571\|ref\|XP_002263458.1\|/0/PREDICTED: zinc finger protein CONSTANS-LIKE 4-like [*Vitis vinifera*] |
| VIT_202s0012g00400 | FLS | 0.942 | VIT_218s0001g05850 | ERF | gi\|225460135\|ref\|XP_002275853.1\|/4.47036e-117/PREDICTED: ethylene-responsive transcription factor ERF022 [*Vitis vinifera*]gi\|297741012\|emb\|CBI31324.3\| unnamed protein product [*Vitis vinifera*] |
| VIT_202s0012g00400 | FLS | 0.919 | VIT_210s0003g00580 | ERF | gi\|297740455\|emb\|CBI30637.3\|/1.01677e-111/unnamed protein product [*Vitis vinifera*] |
| VIT_202s0012g00400 | FLS | 0.947 | VIT_207s0031g01980 | ERF | gi\|225438481\|ref\|XP_002278226.1\|/0/PREDICTED: ethylene-responsive transcription factor ABR1 [*Vitis vinifera*]gi\|296082543\|emb\|CBI21548.3\| unnamed protein product [*Vitis vinifera*] |
| VIT_202s0012g00400 | FLS | 0.926 | VIT_218s0072g00260 | ERF | gi\|297739642\|emb\|CBI29824.3\|/0/unnamed protein product [*Vitis vinifera*] |
| VIT_202s0012g00400 | FLS | 0.949 | VIT_219s0014g02240 | ERF | gi\|225461524\|ref\|XP_002285146.1\|/2.98205e-111/PREDICTED: ethylene-responsive transcription factor 4 [*Vitis vinifera*]gi\|37625037\|gb\|AAQ96342.1\| putative ethylene response factor ERF3b [Vitis aestivalis]gi\|302142968\|emb\|CBI20263.3\| unnamed protein product [*Vitis vinifera*] |
| VIT_202s0012g00400 | FLS | 0.900 | VIT_216s0013g01080 | ERF | gi\|297745018\|emb\|CBI38610.3\|/0/unnamed protein product [*Vitis vinifera*] |
| VIT_202s0012g00400 | FLS | 0.921 | VIT_212s0028g03100 | G2-like | gi\|302143340\|emb\|CBI21901.3\|/0/unnamed protein product [*Vitis vinifera*] |
| VIT_202s0012g00400 | FLS | 0.956 | VIT_203s0038g00490 | GATA | gi\|297744743\|emb\|CBI38005.3\|/0/unnamed protein product [*Vitis vinifera*] |
| VIT_202s0012g00400 | FLS | 0.901 | VIT_219s0014g04940 | GRAS | gi\|302143189\|emb\|CBI20484.3\|/0/unnamed protein product [*Vitis vinifera*] |
| VIT_202s0012g00400 | FLS | 0.911 | VIT_219s0090g01740 | GRAS | gi\|297737537\|emb\|CBI26738.3\|/0/unnamed protein product [*Vitis vinifera*] |
| VIT_202s0012g00400 | FLS | 0.908 | VIT_208s0007g06670 | HD-ZIP | gi\|225441481\|ref\|XP_002275747.1\|/7.83705e-137/PREDICTED: homeobox-leucine zipper protein HAT14-like [*Vitis vinifera*] |
| VIT_202s0012g00400 | FLS | 0.902 | VIT_200s0732g00010 | HD-ZIP | gi\|359489471\|ref\|XP_002267504.2\|/2.84738e-162/PREDICTED: uncharacterized protein LOC100257521 [*Vitis vinifera*]gi\|296089004\|emb\|CBI38707.3\| unnamed protein product [*Vitis vinifera*] |
| VIT_202s0012g00400 | FLS | 0.935 | VIT_208s0007g03900 | HSF | gi\|297740065\|emb\|CBI30247.3\|/0/unnamed protein product [*Vitis vinifera*] |
| VIT_202s0012g00400 | FLS | 0.909 | VIT_201s0011g05970 | HSF | gi\|359472583\|ref\|XP_003631170.1\|/0/PREDICTED: LOW QUALITY PROTEIN: heat stress transcription factor A-8-like [*Vitis vinifera*] |
| VIT_202s0012g00400 | FLS | 0.928 | VIT_213s0019g03810 | LBD | gi\|296086065\|emb\|CBI31506.3\|/1.51408e-92/unnamed protein product [*Vitis vinifera*] |
| VIT_202s0012g00400 | FLS | 0.906 | VIT_217s0000g03720 | LBD | gi\|297734202\|emb\|CBI15449.3\|/4.60899e-162/unnamed protein product [*Vitis vinifera*] |
| VIT_202s0012g00400 | FLS | 0.956 | VIT_214s0083g01030 | MIKC_MADS | gi\|298204454\|emb\|CBI16934.3\|/4.57831e-155/unnamed protein product [*Vitis vinifera*] |
| VIT_202s0012g00400 | FLS | 0.928 | VIT_200s0203g00170 | MYB | gi\|359495731\|ref\|XP_002264563.2\|/0/PREDICTED: transcription factor LAF1-like [*Vitis vinifera*]gi\|297745661\|emb\|CBI40872.3\| unnamed protein product [*Vitis vinifera*] |
| VIT_202s0012g00400 | FLS | 0.946 | VIT_206s0080g00790 | MYB | gi\|296084883\|emb\|CBI28292.3\|/3.08946e-156/unnamed protein product [*Vitis vinifera*] |
| VIT_202s0012g00400 | FLS | 0.908 | VIT_209s0002g03610 | MYB_related | gi\|225442479\|ref\|XP_002283785.1\|/0/PREDICTED: uncharacterized protein LOC100241227 [*Vitis vinifera*]gi\|297743200\|emb\|CBI36067.3\| unnamed protein product [*Vitis vinifera*] |
| VIT_202s0012g00400 | FLS | 0.928 | VIT_215s0048g02280 | NAC | gi\|296089035\|emb\|CBI38738.3\|/0/unnamed protein product [*Vitis vinifera*] |
| VIT_202s0012g00400 | FLS | 0.943 | VIT_206s0080g00780 | NAC | gi\|296089502\|emb\|CBI39321.3\|/6.43092e-170/unnamed protein product [*Vitis vinifera*] |
| VIT_202s0012g00400 | FLS | 0.961 | VIT_207s0031g02610 | NAC | gi\|296082607\|emb\|CBI21612.3\|/0/unnamed protein product [*Vitis vinifera*] |
| VIT_202s0012g00400 | FLS | 0.906 | VIT_218s0001g11980 | NAC | gi\|225458374\|ref\|XP_002283395.1\|/0/PREDICTED: uncharacterized protein LOC100252228 [*Vitis vinifera*] |
| VIT_202s0012g00400 | FLS | 0.946 | VIT_201s0026g02710 | NAC | gi\|296086346\|emb\|CBI31935.3\|/0/unnamed protein product [*Vitis vinifera*] |
| VIT_202s0012g00400 | FLS | 0.918 | VIT_219s0014g03290 | NAC | gi\|225461361\|ref\|XP_002284668.1\|/0/PREDICTED: NAC domain-containing protein 72 [*Vitis vinifera*]gi\|302143055\|emb\|CBI20350.3\| unnamed protein product [*Vitis vinifera*] |
| VIT_202s0012g00400 | FLS | 0.920 | VIT_209s0002g01590 | NF-YA | gi\|225442180\|ref\|XP_002274458.1\|/0/PREDICTED: nuclear transcription factor Y subunit A-3 [*Vitis vinifera*]gi\|297743031\|emb\|CBI35898.3\| unnamed protein product [*Vitis vinifera*] |
| VIT_202s0012g00400 | FLS | 0.924 | VIT_208s0007g08250 | NF-YA | gi\|297739684\|emb\|CBI29866.3\|/0/unnamed protein product [*Vitis vinifera*] |
| VIT_202s0012g00400 | FLS | 0.925 | VIT_206s0009g00410 | TALE | gi\|296084040\|emb\|CBI24428.3\|/0/unnamed protein product [*Vitis vinifera*] |
| VIT_202s0012g00400 | FLS | 0.932 | VIT_217s0000g05810 | WRKY | gi\|297734011\|emb\|CBI15258.3\|/0/unnamed protein product [*Vitis vinifera*] |
| VIT_202s0012g00400 | FLS | 0.934 | VIT_216s0050g02510 | WRKY | gi\|359490533\|ref\|XP_002267793.2\|/0/PREDICTED: probable WRKY transcription factor 53-like [*Vitis vinifera*]gi\|302143686\|emb\|CBI22547.3\| unnamed protein product [*Vitis vinifera*] |
| VIT_202s0012g00400 | FLS | 0.950 | VIT_210s0003g02810 | WRKY | gi\|225463412\|ref\|XP_002272089.1\|/0/PREDICTED: probable WRKY transcription factor 28 [*Vitis vinifera*]gi\|297740645\|emb\|CBI30827.3\| unnamed protein product [*Vitis vinifera*] |
| VIT_202s0012g00400 | FLS | 0.946 | VIT_204s0069g00920 | WRKY | gi\|296083797\|emb\|CBI24014.3\|/2.44175e-175/unnamed protein product [*Vitis vinifera*] |
| VIT_202s0012g00400 | FLS | 0.953 | VIT_207s0031g00080 | WRKY | gi\|296082371\|emb\|CBI21376.3\|/8.1921e-161/unnamed protein product [*Vitis vinifera*] |
| VIT_202s0012g00400 | FLS | 0.950 | VIT_210s0003g01600 | WRKY | gi\|225443744\|ref\|XP_002269267.1\|/1.49478e-160/PREDICTED: probable WRKY transcription factor 65 [*Vitis vinifera*]gi\|297740534\|emb\|CBI30716.3\| unnamed protein product [*Vitis vinifera*] |
| VIT_202s0012g00400 | FLS | 0.948 | VIT_215s0046g02190 | WRKY | gi\|225454298\|ref\|XP_002276925.1\|/0/PREDICTED: WRKY transcription factor 22 [*Vitis vinifera*]gi\|297745327\|emb\|CBI40407.3\| unnamed protein product [*Vitis vinifera*] |
| VIT_202s0012g00400 | FLS | 0.924 | VIT_210s0116g01200 | WRKY | gi\|302144104\|emb\|CBI23209.3\|/0/unnamed protein product [*Vitis vinifera*] |
| VIT_202s0012g00400 | FLS | 0.948 | VIT_209s0018g00240 | WRKY | gi\|298204668\|emb\|CBI25166.3\|/1.04765e-177/unnamed protein product [*Vitis vinifera*] |
| VIT_202s0012g00400 | FLS | 0.912 | VIT_206s0004g07500 | WRKY | gi\|297745809\|emb\|CBI15865.3\|/0/unnamed protein product [*Vitis vinifera*] |
| VIT_202s0012g00400 | FLS | 0.928 | VIT_202s0154g00070 | YABBY | gi\|225426944\|ref\|XP_002266233.1\|/6.75169e-131/PREDICTED: axial regulator YABBY 1 [*Vitis vinifera*]gi\|297741152\|emb\|CBI31883.3\| unnamed protein product [*Vitis vinifera*] |
| VIT_200s0361g00040 | ANR | 0.934 | VIT_200s1291g00010 | AP2 | gi\|359497499\|ref\|XP_003635541.1\|/1.15837e-19/PREDICTED: AP2-like ethylene-responsive transcription factor AIL6-like [*Vitis vinifera*] |
| VIT_200s0361g00040 | ANR | 0.945 | VIT_200s0772g00020 | AP2 | gi\|296087015\|emb\|CBI33278.3\|/0/unnamed protein product [*Vitis vinifera*] |
| VIT_200s0361g00040 | ANR | 0.983 | VIT_213s0047g00340 | AP2 | gi\|296085415\|emb\|CBI29147.3\|/0/unnamed protein product [*Vitis vinifera*] |
| VIT_200s0361g00040 | ANR | 0.930 | VIT_216s0100g00420 | ARR-B | gi\|297741112\|emb\|CBI31843.3\|/0/unnamed protein product [*Vitis vinifera*] |
| VIT_200s0361g00040 | ANR | 0.920 | VIT_214s0068g01290 | B3 | gi\|296082287\|emb\|CBI21292.3\|/0/unnamed protein product [*Vitis vinifera*] |
| VIT_200s0361g00040 | ANR | 0.967 | VIT_203s0038g02540 | bHLH | gi\|297744597\|emb\|CBI37859.3\|/0/unnamed protein product [*Vitis vinifera*] |
| VIT_200s0361g00040 | ANR | 0.967 | VIT_201s0244g00130 | bHLH | gi\|296088175\|emb\|CBI35667.3\|/0/unnamed protein product [*Vitis vinifera*] |
| VIT_200s0361g00040 | ANR | 0.947 | VIT_203s0091g00210 | bHLH | gi\|225429299\|ref\|XP_002269988.1\|/3.37576e-129/PREDICTED: transcription factor bHLH113-like [*Vitis vinifera*] |
| VIT_200s0361g00040 | ANR | 0.975 | VIT_206s0080g00340 | bZIP | gi\|296084920\|emb\|CBI28329.3\|/0/unnamed protein product [*Vitis vinifera*] |
| VIT_200s0361g00040 | ANR | 0.973 | VIT_219s0015g01020 | bZIP | gi\|225462003\|ref\|XP_002272719.1\|/2.07853e-151/PREDICTED: G-box-binding factor 4 [*Vitis vinifera*]gi\|296089968\|emb\|CBI39787.3\| unnamed protein product [*Vitis vinifera*] |
| VIT_200s0361g00040 | ANR | 0.972 | VIT_216s0013g00870 | C3H | gi\|297744995\|emb\|CBI38587.3\|/0/unnamed protein product [*Vitis vinifera*] |
| VIT_200s0361g00040 | ANR | 0.901 | VIT_210s0003g00030 | Dof | gi\|474401889\|gb\|EMS66149.1\|/0/Heat shock protein 83 [Triticum urartu] |
| VIT_200s0361g00040 | ANR | 0.974 | VIT_205s0077g01860 | ERF | gi\|225431800\|ref\|XP_002272426.1\|/1.46986e-138/PREDICTED: ethylene-responsive transcription factor RAP2-3 [*Vitis vinifera*]gi\|296083324\|emb\|CBI22960.3\| unnamed protein product [*Vitis vinifera*] |
| VIT_200s0361g00040 | ANR | 0.977 | VIT_215s0021g02510 | GATA | gi\|297734547\|emb\|CBI16598.3\|/4.01131e-148/unnamed protein product [*Vitis vinifera*] |
| VIT_200s0361g00040 | ANR | 0.936 | VIT_202s0025g04910 | GRF | gi\|297742645\|emb\|CBI34794.3\|/2.39379e-157/unnamed protein product [*Vitis vinifera*] |
| VIT_200s0361g00040 | ANR | 0.951 | VIT_200s0494g00010 | GRF | gi\|297741782\|emb\|CBI33069.3\|/0/unnamed protein product [*Vitis vinifera*] |
| VIT_200s0361g00040 | ANR | 0.909 | VIT_204s0079g00480 | HD-ZIP | gi\|225466249\|ref\|XP_002268272.1\|/0/PREDICTED: homeobox-leucine zipper protein ROC8 [*Vitis vinifera*]gi\|297738149\|emb\|CBI27350.3\| unnamed protein product [*Vitis vinifera*] |
| VIT_200s0361g00040 | ANR | 0.921 | VIT_210s0003g04670 | HD-ZIP | gi\|359483940\|ref\|XP_002281868.2\|/0/PREDICTED: homeobox-leucine zipper protein HOX32-like isoform 1 [*Vitis vinifera*]gi\|147820218\|emb\|CAN73584.1\| hypothetical protein VITISV_033098 [*Vitis vinifera*]gi\|297740817\|emb\|CBI30999.3\| unnamed protein product [*Vitis vinifera*] |
| VIT_200s0361g00040 | ANR | 0.916 | VIT_208s0056g01650 | LBD | gi\|296085914\|emb\|CBI31238.3\|/1.32103e-135/unnamed protein product [*Vitis vinifera*] |
| VIT_200s0361g00040 | ANR | 0.949 | VIT_218s0001g09250 | LBD | gi\|225458812\|ref\|XP_002285250.1\|/6.73101e-164/PREDICTED: LOB domain-containing protein 38 [*Vitis vinifera*]gi\|302142212\|emb\|CBI19415.3\| unnamed protein product [*Vitis vinifera*] |
| VIT_200s0361g00040 | ANR | 0.943 | VIT_218s0001g13460 | MIKC_MADS | gi\|125616882\|gb\|ABN46893.1\|/5.26712e-147/AP3-like MADS-box protein [Vitis labrusca x *Vitis vinifera*]gi\|302142587\|emb\|CBI19790.3\| unnamed protein product [*Vitis vinifera*] |
| VIT_200s0361g00040 | ANR | 0.953 | VIT_210s0042g00820 | MIKC_MADS | gi\|296085309\|emb\|CBI29041.3\|/3.28994e-146/unnamed protein product [*Vitis vinifera*] |
| VIT_200s0361g00040 | ANR | 0.972 | VIT_218s0041g01880 | MIKC_MADS | gi\|296089427\|emb\|CBI39246.3\|/1.6141e-160/unnamed protein product [*Vitis vinifera*] |
| VIT_200s0361g00040 | ANR | 0.921 | VIT_206s0004g06280 | MYB | gi\|297745927\|emb\|CBI15983.3\|/0/unnamed protein product [*Vitis vinifera*] |
| VIT_200s0361g00040 | ANR | 0.938 | VIT_217s0000g02660 | MYB | gi\|297734298\|emb\|CBI15545.3\|/2.13696e-155/unnamed protein product [*Vitis vinifera*] |
| VIT_200s0361g00040 | ANR | 0.963 | VIT_217s0000g08550 | MYB | gi\|225457011\|ref\|XP_002279033.1\|/0/PREDICTED: transcription factor MYB98 [*Vitis vinifera*]gi\|297733769\|emb\|CBI15016.3\| unnamed protein product [*Vitis vinifera*] |
| VIT_200s0361g00040 | ANR | 0.947 | VIT_209s0002g01410 | MYB | gi\|297743014\|emb\|CBI35881.3\|/0/unnamed protein product [*Vitis vinifera*] |
| VIT_200s0361g00040 | ANR | 0.914 | VIT_218s0117g00210 | MYB | gi\|296081016\|emb\|CBI18520.3\|/5.39304e-132/unnamed protein product [*Vitis vinifera*] |
| VIT_200s0361g00040 | ANR | 0.939 | VIT_206s0061g00470 | MYB | gi\|359487634\|ref\|XP_002283779.2\|/0/PREDICTED: F-actin-capping protein subunit alpha-like [*Vitis vinifera*] |
| VIT_200s0361g00040 | ANR | 0.940 | VIT_217s0000g02650 | MYB | gi\|297744453\|emb\|CBI37715.3\|/2.27202e-66/unnamed protein product [*Vitis vinifera*] |
| VIT_200s0361g00040 | ANR | 0.963 | VIT_201s0026g02600 | MYB | gi\|359473355\|ref\|XP_002269342.2\|/0/PREDICTED: uncharacterized protein LOC100253313 [*Vitis vinifera*] |
| VIT_200s0361g00040 | ANR | 0.943 | VIT_211s0016g01300 | MYB | gi\|297738580\|emb\|CBI27825.3\|/2.15906e-125/unnamed protein product [*Vitis vinifera*] |
| VIT_200s0361g00040 | ANR | 0.947 | VIT_206s0009g02480 | MYB | gi\|296083906\|emb\|CBI24294.3\|/0/unnamed protein product [*Vitis vinifera*] |
| VIT_200s0361g00040 | ANR | 0.984 | VIT_208s0056g01190 | MYB | gi\|296085886\|emb\|CBI31210.3\|/0/unnamed protein product [*Vitis vinifera*] |
| VIT_200s0361g00040 | ANR | 0.929 | VIT_204s0008g02710 | NAC | gi\|225429822\|ref\|XP_002280812.1\|/0/PREDICTED: NAC domain-containing protein 100 [*Vitis vinifera*]gi\|296081784\|emb\|CBI20789.3\| unnamed protein product [*Vitis vinifera*] |
| VIT_200s0361g00040 | ANR | 0.910 | VIT_212s0028g03050 | NAC | gi\|225446463\|ref\|XP_002275319.1\|/0/PREDICTED: NAC domain-containing protein 8 [*Vitis vinifera*]gi\|302143344\|emb\|CBI21905.3\| unnamed protein product [*Vitis vinifera*] |
| VIT_200s0361g00040 | ANR | 0.906 | VIT_208s0032g01190 | NF-YA | gi\|296089371\|emb\|CBI39143.3\|/0/unnamed protein product [*Vitis vinifera*] |
| VIT_200s0361g00040 | ANR | 0.948 | VIT_211s0016g01480 | NF-YA | gi\|297738597\|emb\|CBI27842.3\|/0/unnamed protein product [*Vitis vinifera*] |
| VIT_200s0361g00040 | ANR | 0.982 | VIT_219s0015g00590 | NF-YB | gi\|225461931\|ref\|XP_002268482.1\|/3.77787e-128/PREDICTED: nuclear transcription factor Y subunit B-6 [*Vitis vinifera*]gi\|296089925\|emb\|CBI39744.3\| unnamed protein product [*Vitis vinifera*] |
| VIT_200s0361g00040 | ANR | 0.985 | VIT_200s0956g00020 | NF-YB | gi\|359497402\|ref\|XP_003635503.1\|/2.6067e-152/PREDICTED: nuclear transcription factor Y subunit B-6-like [*Vitis vinifera*]gi\|296083539\|emb\|CBI23532.3\| unnamed protein product [*Vitis vinifera*] |
| VIT_200s0361g00040 | ANR | 0.956 | VIT_214s0006g02320 | NF-YC | gi\|359488151\|ref\|XP_003633710.1\|/1.81341e-92/PREDICTED: nuclear transcription factor Y subunit C-4-like [*Vitis vinifera*]gi\|296087234\|emb\|CBI33608.3\| unnamed protein product [*Vitis vinifera*] |
| VIT_200s0361g00040 | ANR | 0.924 | VIT_204s0044g00060 | Nin-like | gi\|296088659\|emb\|CBI37650.3\|/7.68766e-112/unnamed protein product [*Vitis vinifera*] |
| VIT_200s0361g00040 | ANR | 0.946 | VIT_204s0023g01020 | TALE | gi\|297735226\|emb\|CBI17588.3\|/0/unnamed protein product [*Vitis vinifera*] |
| VIT_200s0361g00040 | ANR | 0.909 | VIT_210s0042g00940 | Trihelix | gi\|296085313\|emb\|CBI29045.3\|/9.4392e-116/unnamed protein product [*Vitis vinifera*] |
| VIT_200s0361g00040 | ANR | 0.925 | VIT_217s0000g10420 | Trihelix | gi\|297733610\|emb\|CBI14857.3\|/0/unnamed protein product [*Vitis vinifera*] |
| VIT_200s0361g00040 | ANR | 0.909 | VIT_216s0050g01480 | WRKY | gi\|302143766\|emb\|CBI22627.3\|/2.6536e-116/unnamed protein product [*Vitis vinifera*] |
| VIT_216s0039g02230 | UFGT | 0.913 | VIT_202s0033g00450 | MYB | gi\|47232546\|dbj\|BAD18979.1\|/8.37869e-120/myb-related transcription factor VvMYBA3 [*Vitis vinifera*]gi\|118772095\|gb\|ABL14067.1\| R2R3 MYB transcription factor [*Vitis vinifera*]gi\|118772097\|gb\|ABL14068.1\| similar to R2R3 MYB transcription factor [*Vitis vinifera*]gi\|221271545\|dbj\|BAH15079.1\| myb-related transcription factor [*Vitis vinifera*]gi\|224549566\|gb\|ACN53920.1\| transcription factor MYBA3 [*Vitis vinifera*]gi\|224549586\|gb\|ACN53930.1\| transcription factor MYBA3 [*Vitis vinifera*]gi\|224549612\|gb\|ACN53943.1\| transcription factor MYBA3 [*Vitis vinifera*]gi\|296088409\|emb\|CBI37400.3\| unnamed protein product [*Vitis vinifera*] |

PAL, phenylalanine ammonia-lyase; CCR, cinnamoyl-CoA reductase; CAD, cinnamyl-alcohol dehydrogenase; FLS, flavonol synthase; ANR, anthocyanidin reductase; UFGT, UDP-glucose: flavonoid 3-*O*-glucosyltransferase.
