# Supplementary material for: Comparative physiological, metabolomic, and transcriptomic analyses reveal developmental stage-dependent effects of cluster bagging on phenolic metabolism in Cabernet Sauvignon grape berries
Source: BMC Plant Biol. 2019 Dec 26;19:583. doi: 10.1186/s12870-019-2186-z (PMC6933938; doi:10.1186/s12870-019-2186-z)
Supplement: Supplementary file 15 — Additional file 15: Table S9. List of the primers used in this study. [file 12870_2019_2186_MOESM15_ESM.docx]

**Table S9.** List of the primers used in this study.

| Experiment | Primer name | Sequence (5' to 3') |
| --- | --- | --- |
| qPCR | VviPAL1_qPCR_FP | CTCACACCACAACGGCAACG |
|  | VviPAL1_qPCR_RP | CGCCACCATTCTCTTCACCTC |
|  | VviPAL2_qPCR_FP | CCACCCATCAAGATTCTGCGAG |
|  | VviPAL2_qPCR_RP | CATTGTTCAGTGCGTGTTCTACCA |
|  | VviPAL3_qPCR_FP | GAAGAACCAAACAAGGCGGAG |
|  | VviPAL3_qPCR_RP | AGAGGCAAGCAAGGGGTAATG |
|  | VviPAL4_qPCR_FP | CAACTCTGTGAATGATAACCCCTT |
|  | VviPAL4_qPCR_RP | TGCTCCCTTGAAACCATAGTCC |
|  | VviCHS1_qPCR_FP | AGCCAGTGAAGCAGGTAGCC |
|  | VviCHS1_qPCR_RP | GTGATCCGGAAGTAGTAAT |
|  | VviCHS2_qPCR_FP | TCTGAGCGAGTATGGGAACA |
|  | VviCHS2_qPCR_RP | AGGGTAGCTGCGTAGGTTGG |
|  | VviCHI_qPCR_FP | CAGGCAACTCCATTCTTTTC |
|  | VviCHI_qPCR_RP | TTCTCTATCACTGCATTCCC |
|  | VviF3H1_qPCR_FP | CCAATCATAGCAGACTGTCC |
|  | VviF3H1_qPCR_RP | TCAGAGGATACACGGTTGCC |
|  | VviF3H2_qPCR_FP | CTGTGGTGAACTCCGACTGC |
|  | VviF3H2_qPCR_RP | CAAATGTTATGGGCTCCTCC |
|  | VviF3'H_qPCR_FP | ACGGCTACCACATCCCCAAAAA |
|  | VviF3'H_qPCR_RP | CCCCGAATGGAATCACTTCAAAAT |
|  | VviF3'5'H_qPCR_FP | GAAGTTCGACTGGTTATTAACAAAGAT |
|  | VviF3'5'H_qPCR_RP | AGGAGGAGTGCTTTAATGTTGGTA |
|  | VviFLS1_qPCR_FP | AATCCTCCTTCTTACAGGGA |
|  | VviFLS1_qPCR_RP | AGCCCTAACCCTACCGACAA |
|  | VviFLS2_qPCR_FP | AACCCACCTTCGTACAGGGC |
|  | VviFLS2_qPCR_RP | CCTAACCCTAATGACAGCAA |
|  | VviFLS3_qPCR_FP | AACCAAGATGACTAAGAACC |
|  | VviFLS3_qPCR_RP | CTTCTGTGACTTCCCTGTAG |
|  | VviFLS4_qPCR_FP | AAACCACCTACTTACAGAGC |
|  | VviFLS4_qPCR_RP | ACCTAACCCCAGTGACAGAC |
|  | VviMYBF1_qPCR_FP | GGAGGTTGAGGGGTTGTG |
|  | VviMYBF1_qPCR_RP | AAGTTGGGGAAGAGCAGGAG |
|  | VviMYBPA1_qPCR_FP | AGATCAACTGGTTATGCTTGCT |
|  | VviMYBPA1_qPCR_RP | AACACAAATGTACATCGCACAC |
|  | VviMYBA1_qPCR_FP | GCAAGCCTCAGGACAGAAGAA |
|  | VviMYBA1_qPCR_RP | ATCCCAGAAGCCCACATCAA |
|  | VviUbiquitin1_qPCR_FP | GTGGTATTATTGAGCCATCCTT |
|  | VviUbiquitin1_qPCR_RP | AACCTCCAATCCAGTCATCTAC |
|  | Vviβ-Actin_qPCR_FP | CTTGCATCCCTCAGCACCTT |
|  | Vviβ-Actin_qPCR_RP | TCCTGTGGACAATGGATGGA |
|  | LUC_qPCR_FP | TTCTAAAACGGATTACCAGGGAT |
|  | LUC_qPCR_RP | TCTATGAGGCAGAGCGACACC |
|  | NbUbi3_qPCR_FP | GCCGATTACAACATCCAGAAGG |
|  | NbUbi3_qPCR_RP | TGAAGTACAGCGAGCTTAACC |
|  | NbEF-1α_qPCR_FP | TGTGGAAGTTTGAGACCACC |
|  | NbEF-1α_qPCR_RP | GCAAGCAATGCGTGCTCAC |
| Gene cloning | VviFLS4_CDS_FP | ATGGAATTAAAGACAGTCCAAGCCA |
|  | VviFLS4_CDS_RP | CTACTGTGGAATCTTGTTCAGCTTA |
| Promoter cloning | VviFLS4_promoter_FP | CGACAGGTACAAGAACTGGTAT |
|  | VviFLS4_promoter_RP | CCTTGAATAGACTTGGAGTCAG |
| Prokaryotic expression vector construction | VviFLS4_protein-His_FP | TCCCCCGGGATGGAATTAAAGACAGTCCA |
|  | VviFLS4_protein-His_RP | CCGGAATTCCTACTGTGGAATCTTGTTCA |
| Transient expression vector construction | VviFLS4_promoter-LUC_FP | GGAAATTCGAGCTCGGTACCTGGAACCTTAACCTAATGCT |
|  | VviFLS4_promoter-LUC_RP | GCAGATCTCGAGCCCGGGGTCTTGGGTTTGGGCTTCTA |

The restriction enzyme sites are indicated by underlining. FP, forward primer; RP, reverse primer.
