# Supplementary material for: Comparative physiological, metabolomic, and transcriptomic analyses reveal developmental stage-dependent effects of cluster bagging on phenolic metabolism in Cabernet Sauvignon grape berries
Source: BMC Plant Biol. 2019 Dec 26;19:583. doi: 10.1186/s12870-019-2186-z (PMC6933938; doi:10.1186/s12870-019-2186-z)
Supplement: Supplementary file 16 — Additional file 16: Table S10. The promoter and cDNA sequences of VviFLS4 gene. [file 12870_2019_2186_MOESM16_ESM.docx]

**Table S10.** The promoter and cDNA sequences of *VviFLS4* gene.

>promoter sequence

CGACAGGTACAAGAACTGGTATGGCTTTTTGTATTAGAAAGTTAAAATACAACCAAAATTACAATGATACATTTTACTAAATATTAGATGATTGATCAAGATGATCATAAATGAATAGTTAGGCTTGGTGGAGCAGCAGGATCACCATGGTTGCAAGAGAAGCTAAGTGGTTAAGGAATTTATTAGTGGAAATTATTGTTTGGCCTTAGCCAATGCCTTTAATATCTATATGTTGTGACAGGTTGCTTGTTGAAGGGCATATAGCTAAATTTACAATGTATGGTCATTACAATTGACTTTGTAAAATCAAGACACAGGCTTAAGACATAGCTATGTGAGACAATTACTCACCAATGGAGTAATTACAATTGATTTTGTTAAATCATGCCCAAATTTGGCACACCCCTAGATGAAAAGCCTTGCTAGGGATTTAGTATGGAAAATATAAAGAAGAATGAGACTAAAGCCAACATCTATTATATCACCGATAATGGAACCTTAACCTAATGCTTGAAATATCACATCCTTAGGTTCAAGGAGTAAAAGTATAACATATGTTGTGATTGCAAGCGCTATAATATTAATGACATGTCTCAAAAGTAAAAGGGCTCCATACCTGTAATGTGAAGGTTAATAGTTTTACAACCTATTTATGTTGGAATATTGTGATAACTAGAGGGTCGACATTCTTGATAAGCCTACCCATGTGAGTGTGGGGAATTAGAAAGATGTCTTTCTAAAGATATATAGGACCTTACGTAAATGGGTATGTAATAACGTGATGCGACCCAAATTGAATGAGTGTGATGTCCAAAAAATGCAACCTTAGATTAAAATGTGCTTTGAAATCTTTAAAAGAGGGCCGAGGGGTGACCATTGCATTCATTCATTCAACCATCAAATTCTTCGATTCTTTCCATCATTTCACAACTTTTAGCATTATATTTTCTTCATGAATCAAAATTTTTGTATACTTTTATTGTAAAAAAACATTACAAATATTTTTGACAGGCGAGTTATATTTTGAGGATATTTGGGATCTGCTTTCATCAATGTACAATGGAGGCCTACAAACATAGCTTCATTGTATCTTGGGGGCATATTTGTTATACTCGTGCATTACAAAGTTGGTGCCCAAAGTGACTCTCTACTACACCTTGAAGCATTCTAATTCCAATTTTATTATTTACATTATCTGCTTTCGCACATCATCAATTTCAATATCATGGTACCCACAGTTTTCTCTAGTGGATGATTGCGAAGTTAGAAGTATGAGAAACGACAAAGTTAGAGAGCGACTTTTAAGGACTATTATCAAAAAAGAGAGTTGCCAGCAAGTTGTCATTTTCCCATCTTTATAGCTAGTTAGTTTCTGTTGTTCAAACAGATCTCACTTTTCTCCATCACTCCATCTCCATTTTGAATATTTATTTCAAAAGTTACCCAAAATGTGTATACTTTAATAGTCACAACCACCTCTTTAAACCAAACCTAAACTCAATTATGCGATTTCTACAGCAGATTAAGAATTGTCGAATTGGGAAATCTGTATGGCGTGGCTGTTGATGGTCCGGCTGTACCGTCCATTTCAAGAGTGATGAAGAGATGCATTAATAAGATCGCACCAATAGGAAAACTACCCTCGTGGGCAGGGTTGGTAAGAGCCATTATGCGAACATCCTCGTCACACGTCACCCTTTGGTTACCGCCTTTGAATTACTTGGCTTCCCCTTGTACGCGTGCTTTCTTTCTCGCCCTTCCATGATGACTGCTCGTCCTGTGATTCATTAATGACAGGCTCTATAAAAGTTGGATCAGACTTAGAAGCCCAAACCCAAGACAACACAGAAGAAAGAGTAAGAGAGAGAGGGAAGCAGT**ATG**

Note: The initiation codon is highlighted in bold.

>cDNA sequence

AAAGAGTAAGAGAGAGAGGGAAGCAGTATGGAATTAAAGACAGTCCAAGCCATTGCCTTTTCGTCCATGTCCGCGGGCATCATCCCTTCCGAGTTCATAAGGTCTGAGAAAGAGCAACCTGCTATCACAACCTTTCATGGGTATATCCCGCAAGTCCCCACCATTGATCTCAGTGACCCAGATGAGGAGAAGCTCACGCGTTTGATTGTCGAGGCCAGCAGCGAGTGGGGGATGTTCCAGATTGTGAACCATGGCATTCCCAGTGACGTTATCAGCAACTTGCAGAAAGTTGGCAAGGAGTTCTTTGAGCTCCCACAAGAGGAGAAGGAACTTTATGCTAAGTCTCCTGACTCCAAGTCTATTCAAGGCTATGGAAGCAAGCTTCAAAAAGAAGTGGAGGGCAAGAAAGCTTGGGTCGATCACCTCTTCCATAACATCTGGCCTCCTCCTGCCATTGACTATCAGTTCTGGCCTAAAAAACCACCTACTTACAGAGCTGCGAACGAGGAGTATGCAAAGTGGCTGCAAGGGGTGGCAGACAAGCTGTTTGGTCGTCTGTCACTGGGGTTAGGTCTGGGAGAGGGTACGTTGAAGGAAAGCGTCGGTGGGGATGAATTGTTGTATCTTCTGAAAATAAATTATTACCCACCATGTCCTCGTCCAGACCTGGCTCTTGGGGTGGTTGCCCATACTGATATGTCCGCCATTACCATTCTTATCCCAAATGAAGTTCAGGGCTTGCAGGTTTTTAGAGACGATCATTGGTTTGATGTCAAGTATATCTCCAACGCTCTTGTCATTCATGTCGGTGACCAGTTGGAGATCTTGAGCAACGGAAAATACAAGGCGGTGCTTCACAGGACGACCGTGAACAAGGAGAAGACGAGGATGTCATGGCCGGTGTTCTTGGAGCCACCGTCTGATCAGGTGGTAGGGCCTCTTCCTCAGCTTGTCAACGAAGAGAATCCTGCTAGGTACAAGACCAAGAAATACAGTGATTATGTGTACTGTAAGCTGAACAAGATTCCACAGTAGGTGAAGCCAGTGAGTGTTGAATGGATAATTGTATTGCTTTGCCACTGATATTGTTCAACACAGTAGAACAATTGCTTTATTTGTTGCTGCGTGAGAAGCTATGCTGCTTGGTGCGTGAGAATTAAGAAGATGCTTTGTGATCTGCTTTTTAGTTAGTTTTGGTGGA

Note: The full-length coding sequence is underlined.
